# Metal-Free Diastereo- and Enantioselective Dearomative Formal [3 + 2] Cycloaddition of 2-Nitrobenzofurans and Isocyanoacetate Esters

**DOI:** 10.1021/acs.orglett.2c00427

**Published:** 2022-03-16

**Authors:** Adrian Laviós, Amparo Sanz-Marco, Carlos Vila, M. Carmen Muñoz, José R. Pedro, Gonzalo Blay

**Affiliations:** † Departament de Química Orgànica, Facultat de Química, Universitat de València, Dr. Moliner 50, 46100 Burjassot, València, Spain; ‡ Departament de Física Aplicada, Universitat Politècnica de València, Camí de Vera S/N, 46022 València, Spain

## Abstract

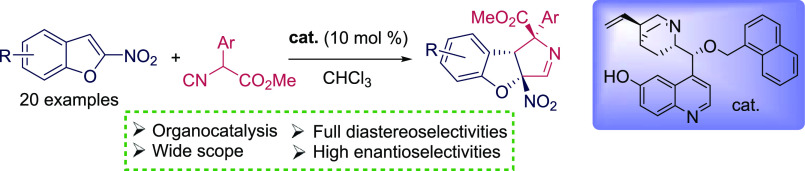

The diastereo- and enantioselective dearomative formal [3 + 2] cycloaddition of 2-nitrobenzofurans and α-aryl-α-isocyanoacetate esters provides tricyclic compounds bearing the 3*a*,8*b*-dihydro-1*H*-benzofuro[2,3-*c*]pyrrole framework with three consecutive stereogenic centers. The reaction was enabled by a cupreine-ether organocatalyst. The reaction products were obtained with almost full diastereoselectivity and with excellent enantiomeric excesses for a number of substituted 2-nitrobenzofurans and isocyanoacetates.

The asymmetric dearomatization of arenes and heteroarenes has become a powerful tool for accessing chiral structures from simple, easily available planar scaffolds.^1^ Many of these reactions involve electron-poor aromatic *aza*-heterocycles such as pyridines, pyrimidines, and their benzo-fused analogues as an approach toward the synthesis of chiral six-membered nonaromatic *aza*-heterocycles.^2^ Dearomatization of these compounds usually involves the participation of species with a positive nitrogen that are obtained through either protonation or *N*-alkylation. In contrast, the asymmetric dearomatization of five-membered heterocycles normally has exploited the nucleophilic character of these electron-rich heterocycles. Nevertheless, installing proper electron-withdrawing substituents on the heteroarenes can reverse them into electron-deficient compounds, thus serving as electrophiles for Umpolung-like reactions.^3^

Following this strategy, electron-deficient nitroheteroarenes have been successfully used as dienophiles and dipolarophiles in cycloaddition reactions, which are normally initiated by Michael addition followed by intramolecular trapping of the anion by an electrophilic group. Pioneering work by Arai^4^ and Trost^5^ showed the potential of 3-nitroindoles as dipolarophiles in catalytic asymmetric dearomative formal [3 + 2] cycloaddition reactions. Since then, 2- and 3-nitroindoles^6^ and related nitroarenes^7^ have been disclosed as dipolarophiles in cycloaddition reactions with a variety of formal dipoles to provide a great variety of polycyclic structures containing multiple stereogenic centers. Among these, dearomatization of 2-nitrobenzofurans lead to compounds containing a chiral 2,3-dihydrobenzofuran scaffold, which is an important pharmacophore present in biologically active natural products and pharmaceuticals.^8^ Despite these precedents, dearomative cycloaddition reactions with 2-nitrobenzofurans remain rare, and only a reduced number of them have been developed.^6b,6e,9^

On the other hand, α-isocyano esters stand as useful and versatile formal 1,3-dipoles. The high α-acidity together with the electrophilic ability of the isocyano group allows isocyano esters to undergo tandem/cascade reactions with electrophilic unsaturated systems, leading to a variety of five-membered *aza*-heterocycles.^10^ These formal [3 + 2] cycloaddition reactions normally involve nucleophilic addition of the α-enolate followed by intramolecular attack of the resulting anion to the empty orbital of the isocyano group. Despite some success in the application of this strategy, there is still a considerable interest in developing new cycloaddition reactions involving isocyanoacetate esters as formal dipoles to access a diversity of novel structures.

Gribble and coworkers studied the reaction of N-protected 3-nitroindoles with ethyl isocyanoacetate, which provided nonchiral pyrrolo[2,3-*b*]indoles or pyrrolo[3,4-*b*]indoles depending on the N1 protecting group via a Barton–Zard reaction involving the elimination of HNO_2_ (Scheme 1).^11^ However, the Barton–Zard reaction can be interrupted by using α-substituted α-isocyano esters.^12^ Using this modification, Yuan and You developed a catalytic asymmetric dearomatization reaction of 3-nitroindoles with α-substituted α-isocyano esters through an interrupted Barton–Zard reaction enabled by a silver/cinchona-derived aminophosphine catalyst (Scheme 1).^13^ The reaction provided the expected products with fair to good diastereoselectivity and excellent enantioselectivity for α-alkyl isocyanoacetates. A similar catalyst has been recently reported by Yuan and Zhao to catalyze the reaction of 2-nitroindoles with α-aryl α-isocyano esters. The authors also reported the reaction with 3-nitroindoles by using an Ag/BINAP complex (Scheme 1).^14^ To the best of our knowledge, a similar reaction has not been reported under metal-free conditions or with other nitroarenes. Herein we report our results on the organocatalytic enantioselective dearomative [3 + 2]-cycloaddition of 2-nitrobenzofurans and isocyanoacetate esters (Scheme 1).

**Scheme 1:**
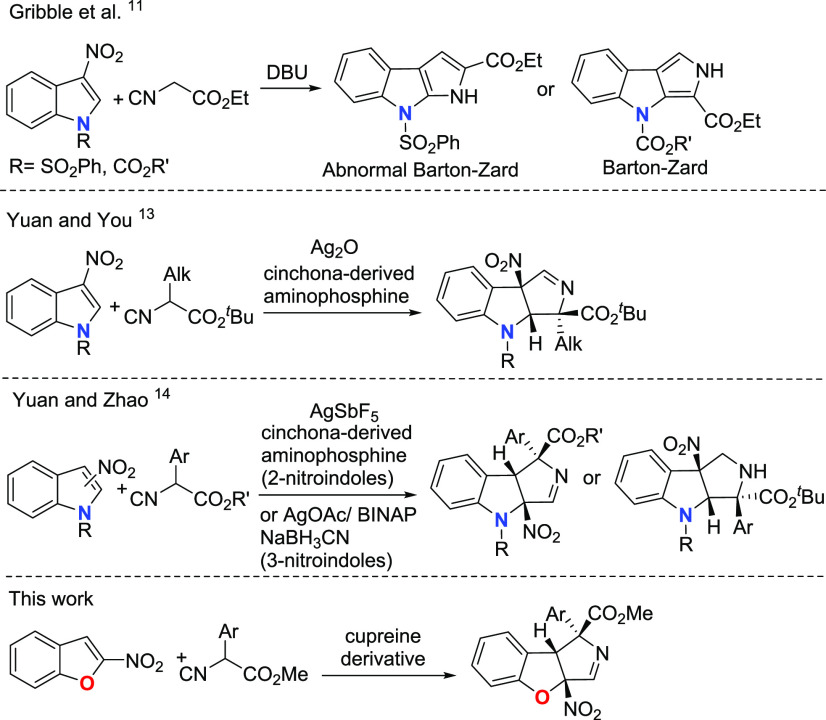
(Interrupted) Barton–Zard Reactions with Nitroarenes

To carry out the optimization process, we used the reaction between 2-nitrobenzofuran (**1a**) and methyl 2-phenyl-2-isocyanoacetate (**2a**). Following our previous research on isocyanoacetate cycloadditions,^15^ we first tested the reaction in the presence of bifunctional squaramide **I** and Ag_2_O in dichloromethane. Under these conditions, compound **3a** was obtained with fair diastereoselectivity, although with low enantiomeric excess for the major diastereomer. We also observed that silver oxide alone was able to catalyze the reaction. Accordingly, further optimization was pursued in the absence of silver salts (Table 1). In this way, squaramide **I** provided compound **3a** with null diastereoselectivity, although the *ee* increased for both diastereomers. A number of bifunctional squaramides were tested, but none of them allowed us to obtain a good enantiomeric excess for the major diasteromer. (See the SI.) In a similar way, thiourea **II** provided better diastereoselectivity but again with low *ee* for the major diastereomer. Fortunately, cupreine derivative **III** allowed us to obtain compound **3a** as just one diastereomer with a promising 73% *ee* (Table 1, entry 4). Increasing the catalyst load to 15 mol % had little effect on the stereoselectivity, whereas lowering the reaction temperature to 0 °C improved both the diastereo- and enantioselectivity, although at the expense of reducing the yield (Table 1, entry 6). Increasing the concentration permitted us to improve the yield while keeping the high stereoselectivity. Finally, several solvents were tested, with the best result being obtained in chloroform, which furnished compound **3a** in 79% yield as only one diastereomer with 93% *ee* (Table 1, entry 11).

**Table 1:**
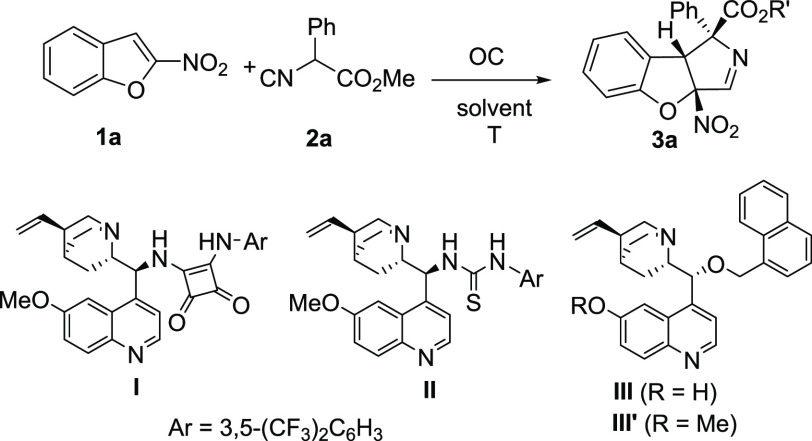
Optimization of the Reaction Conditions^a^

entry	OC (mol %)	solvent	[**1a**] (M)	*T* (°C)	yield (%)^b^	*dr*^c^	*ee* (%)^d^
1^e^	**I** (10)	CH_2_Cl_2_	0.1	rt	95	61:39	10/30
2	**I** (10)	CH_2_Cl_2_	0.1	rt	95	50:50	35/37
3	**II** (10)	CH_2_Cl_2_	0.1	rt	62	17:83	80/6
4	**III** (10)	CH_2_Cl_2_	0.1	rt	73	3:97	9/73
5	**III** (15)	CH_2_Cl_2_	0.1	rt	65	1:99	nd/76
6	**III** (15)	CH_2_Cl_2_	0.1	0	48	1:99	nd/93
7	**III** (15)	CH_2_Cl_2_	0.2	0	75	1:99	nd/90
8	**III** (10)	CH_2_Cl_2_	0.2	0	74	1:99	nd/90
9	**III** (10)	MTBE	0.2	0	46	1:99	nd/74
10	**III** (10)	toluene	0.2	0	40	1:99	nd/93
11	**III** (10)	CHCl_3_	0.2	0	79	1:99	nd/93
12^f^	**III′** (10)	CHCl_3_	0.2	0	19	1:99	nd/10

a Conditions: **1a** (0.1 mmol), **2a** (0.13 mmol), **OC**, solvent, 48 h.

b Isolated yield after column chromatography.

c Determined by ^1^H NMR.

d Determined by HPLC over chiral stationary phases.

e Ag_2_O (5 mol %) was used.

f Control experiment; see the mechanistic discussion

Under the optimized conditions, the scope of the reaction of methyl 2-phenylisocyanoacetate (**2a**) and several substituted 2-nitrobenzofurans **1** was studied (Scheme 2, **1a**–**1o**). The reaction could be successfully achieved with 2-nitrobenzofurans bearing substituents of varied electronic natures at different positions of the homoaromatic ring. Good yields, full diastereoselectivities, and high enantioselectivities were obtained with nitrobenzofurans substituted at the five (**3ba–3ga**), six (**3ha**, **3ia**), or seven (**3ja–3la**) position with either electron-donating or electron-withdrawing groups. In general, somehow better yields were obtained when this substituent was a halogen or an electron-withdrawing group. Unfortunately, substitution at position 4 of the benzofuran ring brought about a serious decrease in yield and enantioselectivity (**3ma**). We also tested some disubstituted nitrobenzofurans. 5,7-Dicloro-2-nitrobenzofuran (**1o**) reacted with **2a** to give the expected cycloaddition product **3oa** in excellent yield with excellent enantioselectivity. On the contrary, compound **1n** bearing two bulky *tert*-butyl groups reacted to give **3na** with good enantioselectivity, although in lower yield.

**Scheme 2:**
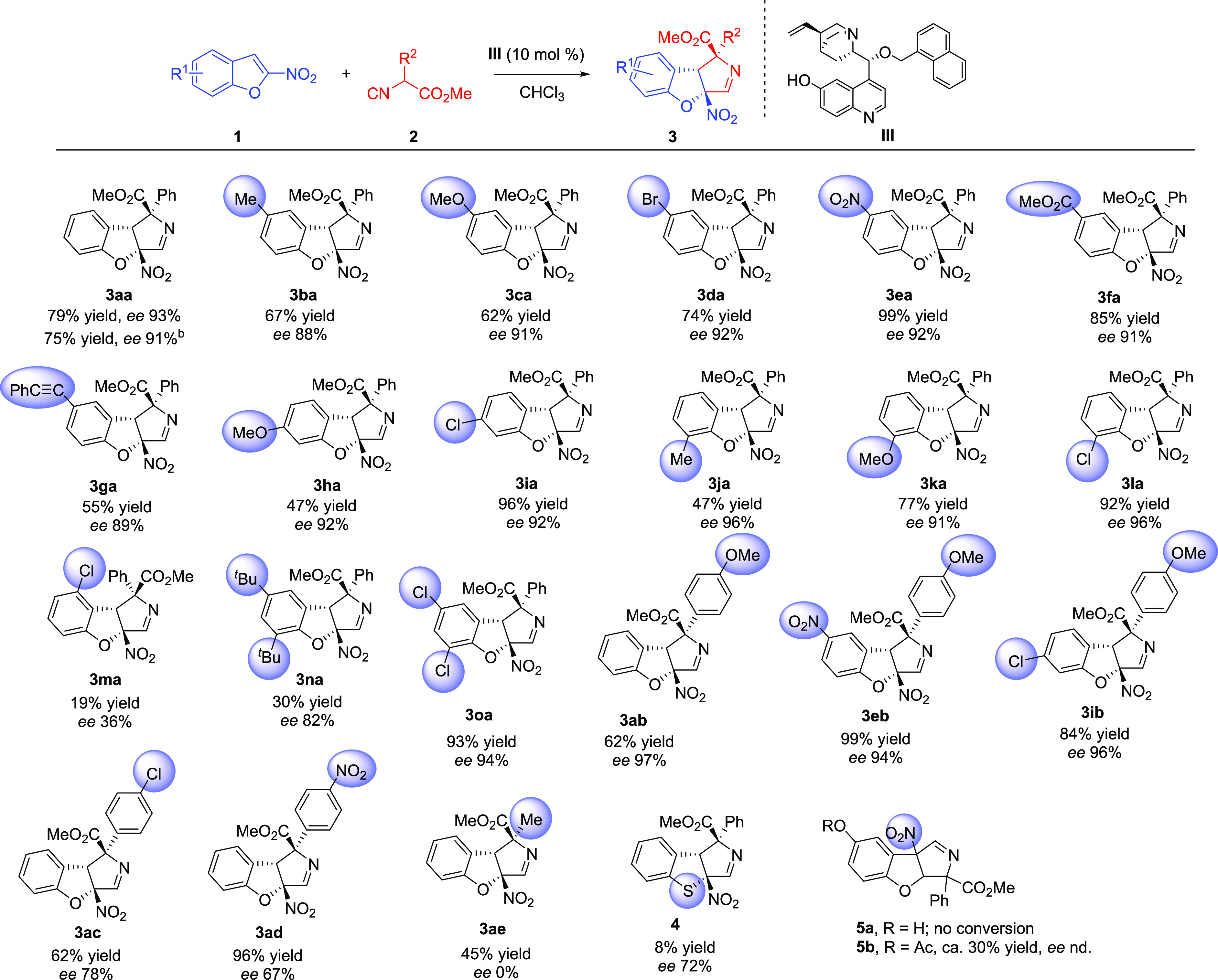
Scope of the Reaction of 2-Nitrobenzofurans **1** and Isocyanoacetates **2** Conditions: **1a** (0.15 mmol), **2a** (0.19 mmol), **III** (0.015 mmol), CHCl_3_ (0.75 mL), 0 °C. Yields after column chromatography, *dr* determined by ^1^H NMR, *ee* determined by HPLC. Reaction carried out on a 1 mmol scale.

Next, we studied the reaction with different isocyanoacetates **2**. Methyl (4-methoxyphenyl)isocyanoacetate (**2b**, R^2^ = 4-MeOC_6_H_4_) reacted with several 2-nitrobenzofurans to give the corresponding products **3ab**, **3eb**, and **3ib** in good yields with excellent enantioselectivities, higher than those obtained with isocyanoacetate **2a**. However, when an electron-withdrawing group was introduced on the aryl ring of the isocyanoacetate (**2c**, R^2^ = 4-ClC_6_H_4_; **2d**, R^2^ = 4-NO_2_C_6_H_4_) the reaction products **3ac** and **3ad** were obtained with lower enantioselectivities, although still in good yields with full diastereoselectivity. On the contrary, methyl 2-isocyanopropanoate (**2e**, R^2^ = Me) reacted with **1a** and provided a racemic product **3ae** in low yield. We also tested the reaction of **2a** with 2-nitrobenzotiophene, which was sluggish and gave compound **4** in low yield with fair enantioselectivity (72% *ee*). Finally, the reaction with the less synthetically accessible 3-nitrobenzofurans was attempted. The reaction of **2a** with commercially available 5-hydroxy-3-nitrobenzofuran did not proceed under our reaction conditions, probably due to the presence of the free OH group in the substrate (Scheme 2, **5a**). In fact, 5-acetoxy-3-nitrobenzofuran reacted with **2a** to give compound **5b** in ca. 30% yield, although unfortunately, the enantiomeric excess could not be determined.^16^

To highlight the robustness of the method, we carried out the reaction of **1a** and **2a** on a 1 mmol scale to obtain compound **3aa** in 75% yield with 91% *ee*, which was comparable to that obtained in the model reaction. Furthermore, some synthetic transformations were carried out with compound **3aa** (Scheme 3). Elimination of the nitro group upon treatment with DBU in dichloromethane gave compound **6** in 53% yield with 90% *ee*. Reduction of the C–N double bond was achieved with Et_3_SiH in the presence of BF_3_·Et_2_O to give compound **7** in quantitative yield with a slight erosion of the *ee*. Finally, aminoacetal **8** could be obtained in 99% yield with full diastereoselectivity and 98% *ee* after refluxing in MeOH. In a similar way, starting from **3da**, we could obtain compound **9**, which could be crystallized and subjected to single-crystal X-ray diffraction analysis (Figure 1). In this way, we could establish its absolute stereochemistry and hence that of compound **3da**. The stereochemistry (1*R*,3*aR*,8*bR*) of all compounds **3** was assigned by analogy, assuming a common stereochemical pathway.

**Scheme 3:**
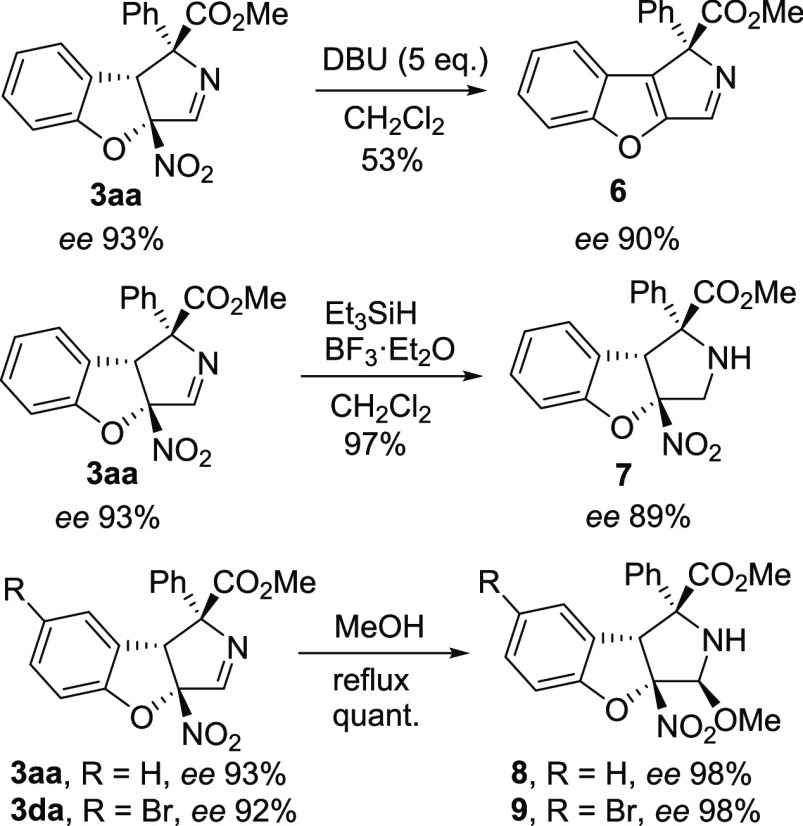
Synthetic Transformations of **3aa**

**Figure 1:**
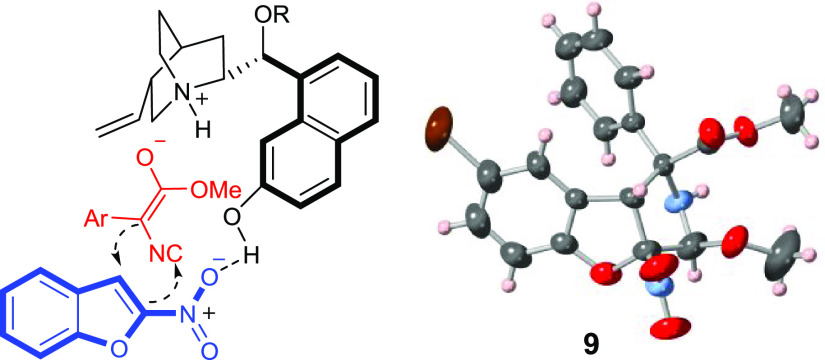
Proposed stereochemical model and ORTEP plot for the X-ray structure of compound **9** with thermal ellipsoids drawn at the 50% probability level. Flack parameter 0.005(15).

To get some insight into the reaction mechanism, we performed a control experiment running the reaction of **1a** and **2a** in the presence of compound **III′**, the methyl ether of catalyst **III** lacking a free OH phenol group. Under these conditions, the reaction took place sluggishly to give compound **3aa** in 19% yield with 10% enantiomeric excess (Table 1, entry 12). These results indicate that compound **III** most probably works as a bifunctional catalyst with the free OH phenol group and the basic amine acting synergistically. According to literature precedents and the observed stereochemistry,^18^ we propose the stereochemical model outlined in Figure 1. Catalyst **III** activates the electrophile through H bonding of the phenol and the nitro group, whereas the tertiary base deprotonates the isocyano ester. The approach of the nucleophile is guided by ion pairing with the ammonium salt facing the *Si* face of the ester enolate and the *Si* face of the furan double bond to account for the observed stereochemistry.

In summary, we have established a highly diastereo- and enantioselective organocatalytic procedure for the dearomative formal [3 + 2] cycloaddition of 2-nitrobenzofurans and 2-aryl-2-isocyanoacetate esters. Although related reactions with 2- and 3-nitroindoles have been reported, this is the first example using 2-nitrobenzofurans. The reaction is catalyzed by a cupreine derivative in the absence of metals and provides chiral tricyclic compounds in good yields with full diastereoselectivity and excellent enantioselectivity for a number of 2-nitrobenzofuran derivatives substituted at the five, six, or seven position. The arylisocyanoacetate ester allows substitution at the aryl ring, with the best results being obtained with phenyl or 4-methoxyphenyl derivatives. The potential synthetic applicability of the reaction has been demonstrated with a 1 mmol scale reaction and versatile modifications of compound **3aa**.

## Supporting Information Available

Experimental procedures, characterization data, NMR spectra, and HPLC traces (PDF)
FAIR data, including the primary NMR FID files, for compounds **3**–**8** (ZIP)

## Accession Codes

CCDC 2149627 contains the supplementary crystallographic data for this paper. These data can be obtained free of charge via www.ccdc.cam.ac.uk/data_request/cif, or by emailing data_request@ccdc.cam.ac.uk, or by contacting The Cambridge Crystallographic Data Centre, 12 Union Road, Cambridge CB2 1EZ, UK; fax: +44 1223 336033.
