# Supplementary material for: Metal-Free Diastereo- and Enantioselective Dearomative Formal [3 + 2] Cycloaddition of 2-Nitrobenzofurans and Isocyanoacetate Esters
Source: Org Lett. 2022 Mar 16;24(11):2149–54. doi: 10.1021/acs.orglett.2c00427 (PMC8961877; doi:10.1021/acs.orglett.2c00427)
Supplement: Supplementary file 1 — ol2c00427_si_001.pdf [file ol2c00427_si_001.pdf]

Metal-free diastereo- and enantioselective dearomative formal [3+2] cycloaddition of 2-nitrobenzofurans and isocyanoacetate esters

Adrian Laviós,[†] Amparo Sanz-Marco,[†] Carlos Vila,[†] M. Carmen Muñoz,[‡] José R. Pedro,[†*] and Gonzalo Blay[†]*

† Departament de Química Orgànica, Facultat de Química, Universitat de València, Dr. Moliner 50, 46100-Burjassot, València, Spain.

‡ Departament de Física Aplicada, Universitat Politècnica de València, Camí de Vera S/N, 46022-València, Spain

Experimental Procedures 2

Synthesis and characterization data for compounds **1** 3

Synthesis and characterization data of 2-nitrobenzothiophene 11

Synthesis and characterization data for 5-acetoxy-3-nitrobenzofuran 12

Synthesis and characterization data for isocyanides **2** 12

Synthesis and characterization data for catalyst **III** 16

Synthesis and characterization data for compounds **3** 18

Synthesis of **3aa** at 1 mmol scale 27

Transformations of product **3aa** 27

References 30

NMR spectra 31

HPLC spectra 97

Figure S-1. X-Ray structure for compound **89** 123

Optimization of the reaction conditions. Further experiments 125

Experimental Procedures

Unless otherwise stated, starting materials were obtained from commercial sources and used without previous purification. Reactions monitored by thin-layer chromatography using Silica Gel Merck 60 F_{254} plates (reference 5554 Merck). Eluted TLC plates were observed under 254 nm UV light and developed with cerium molybdate, potassium permanganate or *p*-anisaldehyde stain solutions. Flash column chromatography was carried out with Silica Gel Merck 60 stationary phase (0,040-0,063 mm particle size, reference 109385 Merck). NMR spectra were recorded at 300 MHz, 400 MHz or 500 MHz for ^{1}H, at 75 MHz or 101 MHz for ^{13}C. Signals of residual non-deuterated solvent were used as internal standard (7.26 ppm for ^{1}H and 77.16 ppm for ^{13}C in $CDCl_3$, and 3.31 ppm for ^{1}H and 49.0 ppm for ^{13}C in methanol-d_4). Chemical shifts are given in ppm. Carbon multiplicities were assigned through DEPT experiments. High-resolution mass spectra were recorded in a Q-TOF spectrometer equipped with an electrospray source with a capillary voltage of 3.3 kV (ESI). Specific optical rotations were measured with a polarimeter equipped with a sodium lamp (D line, 589 nm), concentrations (*c*) are given in g/100 mL. Enantiomeric excess values were measured by HPLC analysis, employing a chromatograph equipped with an UV diode array detector and columns composed of a chiral stationary phase, either of Daicel or Phenomenex brands.

Synthesis and characterization data for compounds 1

All 2-nitrobenzofuranes were synthetized via a three-step procedure comprising a nitroaldolic reaction between salicylaldehydes and nitromethane, followed by a $NaBH_4$-mediated reduction of the resulting olefin and a final oxidative cyclization.

Nitrovinylfenols

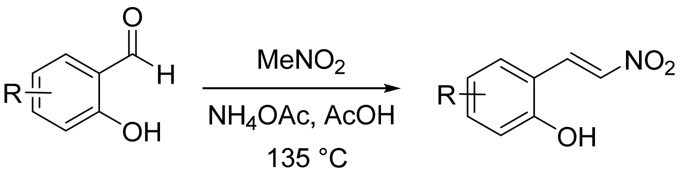

(*E*)-2-(2-Nitrovinyl)phenol *(Representative procedure):*

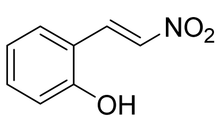

A modification of a literature procedure was carried out.[1] A round-bottom flask was charged with nitromethane (8.2 mL, 151 mmol, 18. 5 equiv), NH_4OAc (126 mg, 1.64 mmol, 0.2 equiv) and acetic acid (3.3 mL, 57.6 mmol, 7.0 equiv). The mixture was stirred in an oil bath at 90 °C for 15 min and then was added salicylaldehyde (0.87 mL, 8.19 mmol, 1.0 equiv). The temperature of the oil bath was adjusted to 135 °C and the reaction mixture stirred at this temperature overnight. After this time, the reaction mixture was left to reach room temperature, Et_2O (80 mL) was added and the organic phase was washed with brine (3×30 mL), dried over Na_2SO_4 and concentrated under reduced pressure. Purification by column chromatography (eluent: hexane:EtOAc 9:1 to 8:2) yielded 0.95 g (70%) of (*E*)-2-(2-nitrovinyl)phenol. **^{1}H NMR** (300 MHz, $CDCl_3$) δ 8.14 (d, *J* = 13.6 Hz, 1H, $CHNO_2$), 7.97 (d, *J* = 13.6 Hz, 1H, CHAr), 7.43 (dd, *J* = 7.7, 1.7 Hz, 1H, Ar), 7.35 (ddd, *J* = 8.1, 7.4, 1.7 Hz, 1H, Ar), 7.01 (td, *J* = 7.6, 1.1 Hz, 1H, Ar), 6.89 (d, *J* = 8.2 Hz, 1H, Ar), 6,06 (s, 1H, OH); **^{13}C NMR** (75 MHz, $CDCl_3$) δ 156.2 (C), 138.7 (CH), 135.8 (CH), 133.4 (CH), 132.8 (CH), 121.7 (CH), 117.8 (C), 116.6 (CH).

(*E*)-4-Methyl-2-(2-nitrovinyl)phenol

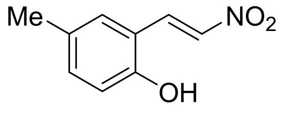

From 5-methylsalicylaldehyde (1.12 g, 8.19 mmol), 801 mg (55%) of the title compound were obtained after column chromatography eluting with hexane:EtOAc (9:1 to 8:2). **^{1}H NMR** (300 MHz, MeOH-*d*$_4$) δ 8.12 (d, *J* = 13.5 Hz, 1H, $CHNO_2$), 8.00 (d, *J* = 13.5 Hz, 1H, CHAr), 7.28 (d, *J* = 2.2 Hz, 1H, Ar), 7.13 (ddd, *J* = 8.4, 2.3, 0.7 Hz, 1H, Ar), 6.80 (d, *J* = 8.3 Hz, 1H, Ar), 2.25 (s, 3H, MeO); **^{13}C NMR** (75 MHz, MeOH-*d*$_4$) δ 157.6 (C), 138.7 (CH), 137.1 (CH), 135.2 (CH), 133.2 (CH), 130.4 (C), 118.4 (C), 117.0 (CH), 20.2 (CH_3).

(*E*)-4-Methoxy-2-(2-nitrovinyl)phenol

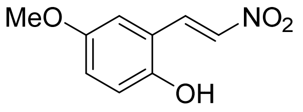

From 5-methoxysalicylaldehyde (0.82 mL, 6.57 mmol), 764 mg (60%) of the title compound were obtained after column chromatography eluting with hexane:EtOAc (8:2). **^{1}H NMR** (300 MHz, MeOH-d_4) δ 8.16 (d, *J* = 13.6 Hz, 1H, $CHNO_2$), 8.02 (d, *J* = 13.6 Hz, 1H, CHAr), 7.04 (d, *J* = 3.0 Hz, 1H, Ar), 6.93 (dd, *J* = 8.9, 3.0 Hz, 1H, Ar), 6.83 (d, *J* = 8.9 Hz, 1H, Ar), 3.76 (s, 3H, MeO); **^{13}C NMR** (75 MHz, MeOH-d_4) δ 154.4 (C), 153.9 (C), 139.0 (CH), 136.7 (CH), 121.5 (CH), 118.8 (C), 118.1 (CH), 115.8 (CH), 56.3 (CH_3).

(*E*)-4-Bromo-2-(2-nitrovinyl)phenol

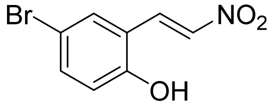

From 5-bromosalicylaldehyde (1.65 g, 8.20 mmol), 1.22 g (61%) of the title compound were obtained after column chromatography eluting with hexane:EtOAc (9:1). **^{1}H NMR** (300 MHz, MeOH-d_4) δ 8.08 (d, *J* = 13.6 Hz, 1H, $CHNO_2$), 8.02 (d, *J* = 13.6 Hz, 1H, CHAr), 7.66 (d, *J* = 2.5 Hz, 1H, Ar), 7.41 (dd, *J* = 8.8, 2.5 Hz, 1H, Ar), 6.84 (d, *J* = 8.8 Hz, 1H, Ar); **^{13}C NMR** (75 MHz, MeOH-d_4) δ 158.7 (C), 139.9 (CH), 136.6 (CH), 135.2 (CH), 135.1 (CH), 120.8 (C), 118.9 (CH), 112.5 (C).

(*E*)-4-Nitro-2-(2-nitrovinyl)phenol

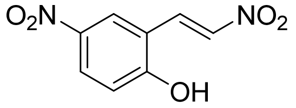

From 5-nitrosalicylaldehyde (1.20 g, 7.19 mmol), 387 mg (26%) of the title compound were obtained after column chromatography eluting with hexane:EtOAc (8:2). **^{1}H NMR** (300 MHz, MeOH-d_4) δ 8.46 (d, *J* = 2.8 Hz, 1H), 8.19 (dd, *J* = 9.1, 2.8 Hz, 1H), 8.15 (d, *J* = 13.7 Hz, 1H, $CHNO_2$), 8.09 (d, *J* = 13.6 Hz, 1H, CHAr), 7.04 (d, *J* = 9.1 Hz, 1H); **^{13}C NMR** (75 MHz, MeOH-d_4) δ 164.6 (C), 142.0 (C), 140.9 (CH), 134.5 (CH), 129.0 (CH), 129.0 (CH), 119.3 (C), 117.4 (CH).

(*E*)-4-Methoxycarbonyl-2-(2-nitrovinyl)phenol

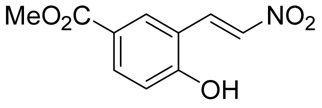

From 4-methoxycarbonylsalicylaldehyde (1.10 g, 6.1 mmol), 700 mg (51%) of the title compound were obtained after column chromatography eluting with hexane:EtOAc (95:5). **^{1}H NMR** (300 MHz, MeOH-d_4) δ 8.14 (d, J = 2.1, 1H, Ar), 8.11 (d, J = 11.4 Hz, 1H, $CHNO_2$), 8.03 (d, J = 11.4 Hz, 1H, CHAr), 7.93 (dd, J = 8.7 Hz, 2.1 Hz, 1H, Ar), 6.95 (d, J = 8.7 Hz), 3.87 (s, 3H, MeO); **^{13}C NMR** (75 MHz, MeOH-d_4) 167.63 (C), 163.42 (C), 139.9 (CH), 135.7 (CH), 135.3 (CH), 135.2 (CH), 123.1 (C), 118.8 (C), 117.1 (CH)), 52.5(CH_3).

(*E*)-5-Methoxy-2-(2-nitrovinyl)phenol

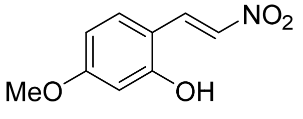

From 4-methoxysalicylaldehyde (1.0 g, 6.57 mmol), 529 mg (41%) of the title compound were obtained after column chromatography eluting with hexane:EtOAc (9:1). **^{1}H NMR** (300 MHz, MeOH-d_4) δ 8.11 (d, *J* = 13.4 Hz,

1H, $CHNO_2$), 7.93 (d, J = 13.4 Hz, 1H, CHAr), 7.40 (d, J = 8.7 Hz, 1H, Ar), 6.50 (dd, J = 8.7, 2.5 Hz, 1H, Ar), 6.44 (d, J = 2.5 Hz, 1H, Ar), 3.80 (s, 3H, MeO); **^{13}C NMR** (75 MHz, MeOH-d_4) δ 165.8 (C), 161.6 (C), 137.3 (CH), 136.4 (CH), 135.0 (CH), 112.0 (C), 108.0 (CH), 102.1 (CH), 55.9 (CH_3).

(*E*)-5-Chloro-2-(2-nitrovinyl)phenol

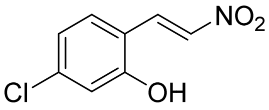

From 4-chlorosalicylaldehyde (700 mg, 4.47 mmol), 487 mg (55%) of the title compound were obtained after column chromatography eluting with hexane:EtOAc (95:5 to 9:1). **^{1}H NMR** (300 MHz, MeOH-d_4) δ 8.11 (d, J = 13.6 Hz, 1H, $CHNO_2$), 8.01 (d, J = 13.6 Hz, 1H, CHAr), 7.48 (d, J = 8.2 Hz, 1H, Ar), 6.99 – 6.86 (m, 2H, Ar); **^{13}C NMR** (75 MHz, MeOH-d_4) δ 160.2 (C), 139.6 (C), 139.2 (CH), 135.6 (CH), 134.3 (CH), 121.4 (CH), 117.8 (C), 117.1 (CH).

(*E*)-2-Methyl-6-(2-nitrovinyl)phenol

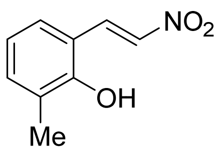

From 3-methylsalicylaldehyde (1.12 g, 8.19 mmol), 801 mg (55%) of compound of the title compound were obtained after column chromatography eluting with hexane:EtOAc (9:1). **^{1}H NMR** (300 MHz, MeOH-d_4) δ 8.25 (d, J = 13.5 Hz, 1H, $CHNO_2$), 7.97 (d, J = 13.5 Hz, 1H, CHAr), 7.43 – 7.28 (m, 1H, Ar), 7.22 (ddd, J = 7.4, 1.7, 0.8 Hz, 1H, Ar), 6.83 (t, J = 7.6 Hz, 1H, Ar), 2.25 (s, 3H, Me); **^{13}C NMR** (75 MHz, MeOH-d_4) δ 157.3 (C), 138.7 (CH), 137.0 (CH), 135.8 (CH), 130.0 (CH), 126.8 (C), 121.4 (CH), 119.4 (C), 16.6 (CH_3).

(*E*)-2-Methoxy-6-(2-nitrovinyl)phenol

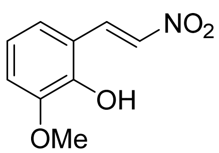

From *o*-vanillin (1.0 g, 6.57 mmol), 1.0 g (78%) of the title compound were obtained after column chromatography eluting with hexane:EtOAc (9:1). **^{1}H NMR** (300 MHz, $CDCl_3$) δ 8.10 (d, J = 13.7 Hz, 1H, $CHNO_2$), 7.95 (d, J = 13.6 Hz, 1H, CHAr), 7.08 – 6.80 (m, 3H, Ar), 6.49 (s, 1H, OH), 3.94 (s, 3H, MeO); **^{13}C NMR** (75 MHz, $CDCl_3$) δ 147.0 (C), 146.6 (C), 138.9 (CH), 135.2 (CH), 123.8 (CH), 120.4 (CH), 116.7 (C), 113.5 (CH), 56.5 (CH_3).

(*E*)-2-Chloro-6-(2-nitrovinyl)phenol

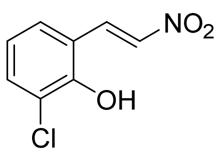

From 3-chlorosalicylaldehyde (700 mg, 4.47 mmol), 457 mg (51%) of the title compound were obtained after column chromatography eluting with hexane:EtOAc (95:5 to 9:1). **^{1}H NMR** (300 MHz, MeOH-d_4) δ 8.19 (d, J = 13.6 Hz, 1H, $CHNO_2$), 8.02 (d, J = 13.6 Hz, 1H, CHAr), 7.47 (ddd, J = 11.5, 7.9, 1.6 Hz, 2H, Ar), 6.91 (t, J = 7.9 Hz, 1H, Ar); **^{13}C NMR** (75 MHz, MeOH-d_4) δ 154.5 (C), 139.9 (CH), 135.7 (CH), 133.9 (CH), 131.4 (CH), 122.9 (C), 121.9 (CH), 121.2 (C).

(*E*)-3-Chloro-2-(2-nitrovinyl)phenol

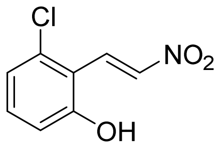

From 6-chlorosalicylaldehyde (1.0 g, 6.39 mmol), 507 mg (40%) of the title compound were obtained after column chromatography eluting with hexane:EtOAc (9:1). ^{1}**H NMR** (300 MHz, MeOH-d_4) δ 8.47 (d, *J* = 13.5 Hz, 1H, $CHNO_2$), 8.21 (d, *J* = 13.5 Hz, 1H, CHAr), 7.25 (t, *J* = 8.2 Hz, 1H, Ar), 7.01 (dd, *J* = 8.0, 1.1 Hz, 1H, Ar), 6.87 (ddd, *J* = 8.3, 1.1, 0.6 Hz, 1H, Ar); ^{13}**C NMR** (75 MHz, MeOH-d_4) δ 161.2 (C), 141.6 (CH), 138.4 (C), 134.0 (CH), 132.4 (CH), 122.2 (CH), 116.9 (C), 115.9 (CH).

(*E*)-2,4-Di-(*tert*-butyl)-6-(2-nitrovinyl)phenol

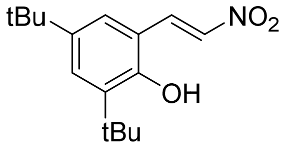

Separation by column chromatography (eluent: hexane:EtOAc 98:2) yielded a mixture containing the title compound, which was used without further purification for the next step.

(*E*)-2,4-dichloro-6-(2-nitrovinyl)phenol

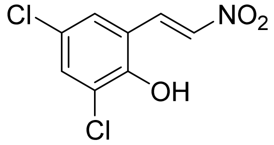

From 3,5-dichlorosalicylaldehyde (700 mg, 3.67 mmol), 337 mg (39%) of the title compound were obtained after column chromatography eluting with hexane:EtOAc (9:1). ^{1}**H NMR** (300 MHz, MeOH-d_4) δ 8.14 (d, *J* = 13.6 Hz, 1H, $CHNO_2$), 8.03 (d, *J* = 13.6 Hz, 1H, CHAr), 7.57 (d, *J* = 2.5 Hz, 1H, Ar), 7.50 (d, *J* = 2.5 Hz, 1H, Ar); ^{13}**C NMR** (75 MHz, MeOH-d_4) δ 153.4 (C), 140.9 (CH), 134.3 (CH), 133.0 (CH), 130.4 (CH), 126.0 (C), 123.8 (C), 122.3 (C).

2-Nitrobenzofurans

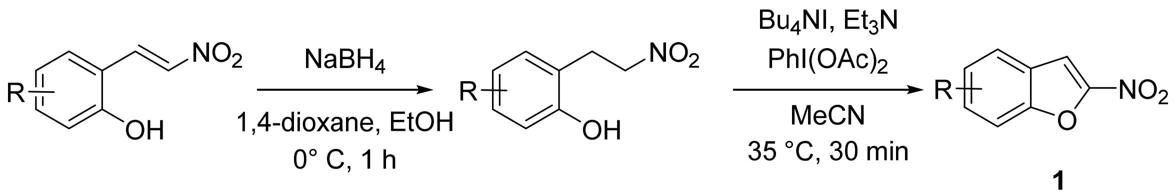

2-Nitrobenzofuran (1a) *(Representative procedure):*

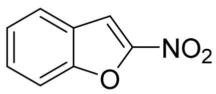
1a

A literature procedure was employed.[1] $NaBH_4$ (231 mg, 6.1 mmol, 1.2 equiv) was suspended in a mixture of 1,4-dioxane:EtOH 3:1 (20 mL), under nitrogen atmosphere. A solution of (*E*)-2-(2-nitrovinyl)phenol (840 mg, 5.08 mmol, 1.0 equiv) in 1,4-dioxane (15 mL, 0.34 M) was added dropwise at 0 °C. The reaction mixture was stirred at 0 °C for 1 h (followed by TLC analysis). The reaction was quenched with NH_4Cl (50 mL) and extracted with Et_2O (3×125 ml). Combined organic phases were washed with brine (3×80 mL) and dried over Na_2SO_4. Concentrated under reduced pressure to furnish an oily crude that was used for the next step without further purification.

The obtained crude was dissolved in MeCN (50 mL, 0.1 M) and Bu_4NI (4.7 g, 12.7 mmol, 2.5 equiv), triethylamine (1.4 mL, 10.2 mmol, 2.0 equiv), and tetrabutylammonium iodide (4.9 g, 15.2 mmol, 3.0 equiv) were added. The mixture was stirred at 35 °C (oil bath) for 30 min (followed by TLC analysis). Et_2O (500 mL) was added and the reaction was washed with brine (3×125 mL). The organic phase was dried over Na_2SO_4 and concentrated under reduced pressure. Purification by column chromatography (eluent: hexane:EtOAc 97:3) yielded 511 mg (62%, over two steps) of 2-nitrobenzofuran (**1a**).[1]

^{1}H NMR (300 MHz, $CDCl_3$) δ 7.77 (dt, *J* = 7.9, 1.1 Hz, 1H), 7.68 (d, *J* = 0.7 Hz, 1H), 7.66 – 7.56 (m, 2H), 7.42 (ddd, *J* = 8.1, 6.0, 2.3 Hz, 1H); **^{13}C NMR** (75 MHz, $CDCl_3$) δ 153.5 (C), 130.1 (CH), 126.0 (C), 125.5 (CH), 124.2 (CH), 112.9 (CH), 107.4 (CH).

5-Methyl-2-nitrobenzofuran (1b)[2]

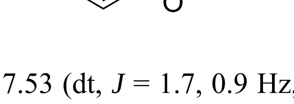

From (*E*)-4-methyl-2-(2-nitrovinyl)phenol (700 mg, 3.91 mmol), 514 mg (65%) of **1b** were obtained after column chromatography eluting with hexane:EtOAc (95:5). **^{1}H NMR** (300 MHz, $CDCl_3$) δ 7.60 (d, *J* = 0.9 Hz, 1H), 7.53 (dt, *J* = 1.7, 0.9 Hz, 1H), 7.50 (d, *J* = 8.7 Hz, 1H), 7.40 (ddd, *J* = 8.7, 1.8, 0.6 Hz, 1H), 2.48 (s, 3H); **^{13}C NMR** (75 MHz, $CDCl_3$) δ 152.0 (C), 135.4 (C), 131.8 (CH), 126.0 (C), 123.5 (CH), 112.4 (CH), 107.2 (CH), 21.5 (CH_3).

5-Methoxy-2-nitrobenzofuran (1c)[2]

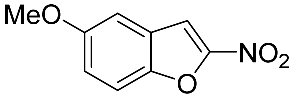

From (*E*)-4-methoxy-2-(2-nitrovinyl)phenol (620 mg, 3.18 mmol), 330 mg (54%) of **1c** were obtained after column chromatography eluting with hexane:EtOAc (95:5 to 9:1). **^{1}H NMR** (300 MHz, $CDCl_3$) δ 7.60 (d, *J* = 0.9 Hz, 1H), 7.51 (dt, *J* = 9.3, 0.7 Hz, 1H), 7.20 (dd, *J* = 9.2, 2.6 Hz, 1H), 7.12 (d, *J* = 2.6 Hz, 1H); **^{13}C NMR** (75 MHz, $CDCl_3$) δ 157.6 (C), 148.6 (C), 126.6 (C), 120.6 (CH), 113.7 (CH), 107.5 (CH), 104.4 (CH), 56.0 (CH_3).

5-Bromo-2-nitrobenzofuran (1d)[3]

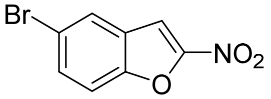

From (*E*)-4-bromo-2-(2-nitrovinyl)phenol **SI1d** (700 mg, 2.87 mmol), 474 mg (68%) of **1d** were obtained after column chromatography eluting with hexane:EtOAc (95:5 to 9:1). **^{1}H NMR** (300 MHz, $CDCl_3$) δ 7.92 (dd, *J* = 2.0, 0.6 Hz, 1H), 7.69 (dd, *J* = 8.9, 2.0 Hz, 1H), 7.61 (d, *J* = 0.9 Hz, 1H), 7.52 (dt, *J* = 9.0, 0.7 Hz, 1H); **^{13}C NMR** (75 MHz, $CDCl_3$) δ 152.0 (C), 133.2 (CH), 127.7 (C), 126.6 (CH), 118.6 (C), 114.5 (CH), 106.3 (CH).

2,5-Dinitrobenzofuran (1e)[4]

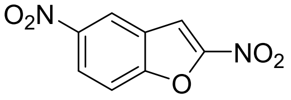

From (*E*)-4-nitrol-2-(2-nitrovinyl)phenol (340 mg, 1.62 mmol), 187 mg (77%) of **1e** were obtained after column chromatography eluting with hexane:EtOAc (9:1 to 8:2). **^{1}H NMR** (300 MHz, $CDCl_3$) δ 8.76 (d, *J* = 2.4 Hz, 1H), 8.51 (dd, *J* = 9.2, 2.4 Hz, 1H), 7.86 – 7.75 (m, 2H); **^{13}C NMR** (75 MHz, $CDCl_3$) δ 155.5 (C), 145.8 (C), 126.2 (C), 125.1 (CH), 120.8 (CH), 113.9 (CH), 107.4 (CH).

Methyl 2-nitrobenzofuran-5-carboxylate (1f)[5]

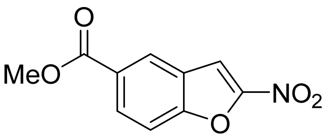

From (*E*)-4-methoxycarbonyl-2-(2-nitrovinyl)phenol (700 mg, 3.14 mmol), 329 mg (47%) of **1f** were obtained after column chromatography eluting with hexane:EtOAc (9:1 to 8:2). **^{1}H NMR** (300 MHz, $CDCl_3$) δ 8.52 (dd, J = 1.8, 0.7 Hz, 1H, Ar), 8.29 (dd, J = 8.9, 1.8 Hz, 1H, Ar), 7.73 (d, J = 1.0 Hz, 1H, Ar), 7.68 (dt, J = 8.9, 0.8 Hz, 1H, Ar), 3.97 (s, 3H, MeO); **^{13}C NMR** (75 MHz, $CDCl_3$) δ 166.1 (C, C=O), 155.5 (C), 131.2 (CH), 128.0 (C), 126.6 (CH), 125.9 (C), 112.9 (CH), 107.5 (CH), 52.7 (CH_3)

6-Methoxy-2-nitrobenzofuran (1h)[2]

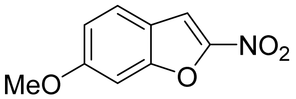

From (*E*)-5-methoxy-2-(2-nitrovinyl)phenol (420 mg, 2.15 mmol), 53 mg (13%) of **1h** were obtained after column chromatography eluting with hexane:EtOAc (95:5 to 9:1). **^{1}H NMR** (300 MHz, $CDCl_3$) δ 7.62 (dd, *J* = 8.4, 0.9 Hz, 2H), 7.07 – 7.01 (m, 2H), 3.90 (s, 3H); **^{13}C NMR** (75 MHz, $CDCl_3$) δ 162.5 (C), 155.2 (C), 124.6 (CH), 119.2 (C), 116.2 (CH), 108.3 (CH), 95.8 (CH), 56.0 (CH_3).

6-Chloro-2-nitrobenzofuran (1i)[2]

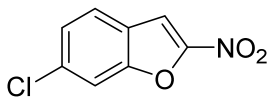

From (E)-5-chloro-2-(2-nitrovinyl)phenol (360 mg, 1.80 mmol), 281 mg (80%) of **1g** were obtained after column chromatography eluting with hexane:EtOAc (95:5). Orange solid; m. p. 104.6-105.3 °C; ^{1}**H NMR** (300 MHz, $CDCl_3$) δ 7.71 (dd, J = 8.5, 0.5 Hz, 1H), 7.67 – 7.62 (m, 2H), 7.42 (dd, J = 8.5, 1.8 Hz, 1H); ^{13}**C NMR** (75 MHz, $CDCl_3$) δ 153.3 (C), 136.2 (C), 126.7 (CH), 124.8 (CH), 124.5 (C), 113.3 (CH), 107.1 (CH).

7-Methyl-2-nitrobenzofuran (1j)[2]

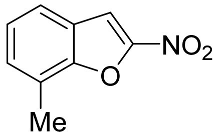

From (E)-6-methyl-2-(2-nitrovinyl)phenol (570 mg, 3.18 mmol), 428 mg (76%) of **1j** were obtained after column chromatography eluting with hexane:EtOAc (97:3). ^{1}**H NMR** (300 MHz, $CDCl_3$) δ 7.65 (s, 1H), 7.58 (ddd, J = 7.8, 1.5, 0.7 Hz, 1H), 7.39 (ddd, J = 7.4, 1.5, 0.8 Hz, 1H), 7.31 (t, J = 7.6 Hz, 1H), 2.59 (s, 3H); ^{13}**C NMR** (75 MHz, $CDCl_3$) δ 152.8 (C), 130.8 (CH), 125.6 (C), 125.5 (CH), 123.3 (C), 121.5 (CH), 107.7 (CH), 15.0 (CH_3).

7-Methoxy-2-nitrobenzofuran (1k)[2]

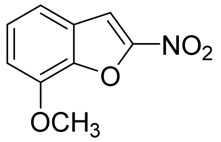

From (E)-6-methoxy-2-(2-nitrovinyl)phenol (500 mg, 2.56 mmol), 295 mg (60%) of **1k** were obtained after column chromatography eluting with hexane:EtOAc (95:5 to 9:1). ^{1}**H NMR** (300 MHz, $CDCl_3$) δ 7.64 (s, 1H), 7.39 – 7.27 (m, 2H), 7.05 (dd, J = 5.7, 3.3 Hz, 1H); ^{13}**C NMR** (75 MHz, $CDCl_3$) δ 146.1 (C), 143.2 (C), 127.5 (C), 126.2 (CH), 115.6 (CH), 111.2 (CH), 107.5 (CH), 56.4 (CH_3).

7-Chloro-2-nitrobenzofuran (1l)[2]

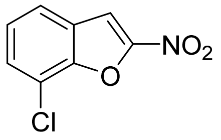

From (E)-6-chloro-2-(2-nitrovinyl)phenol (360 mg, 1.80 mmol), 265 mg (74%) of **1l** were obtained after column chromatography eluting with hexane:EtOAc (95:5 to 9:1). Pale yellow solid; m. p. 126.5-127.8 °C; ^{1}**H NMR** (300 MHz, $CDCl_3$) δ 7.72 – 7.66 (m, 2H), 7.60 (dd, J = 7.9, 1.1 Hz, 1H), 7.37 (t, J = 7.9 Hz, 1H); ^{13}**C NMR** (75 MHz, $CDCl_3$) δ 149.4 (C), 129.9 (CH), 127.4 (C), 126.3 (CH), 122.6 (CH), 118.4 (C), 107.6 (CH).

4-Chloro-2-nitrobenzofuran (1m)[2]

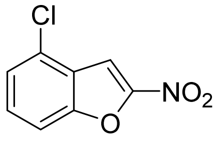

From (E)-3-chloro-2-(2-nitrovinyl)phenol (470 mg, 2.36 mmol), 293 mg (58%) of **1k** were obtained after column chromatography eluting with hexane:EtOAc (99:1). Orange solid; m. p. 110.9-111.6 °C; ^{1}**H NMR** (300 MHz, $CDCl_3$) δ 7.75 (d, J = 0.6 Hz, 1H), 7.59 – 7.48 (m, 2H), 7.48 – 7.38 (m, 1H); ^{13}**C NMR** (75 MHz, $CDCl_3$) δ 153.3 (C), 130.5 (CH), 129.3 (C), 125.9 (C), 125.3 (CH), 111.4 (CH), 105.8 (CH).

5,7-Di(*tert*-butyl)-2-nitrobenzofuran (1n)

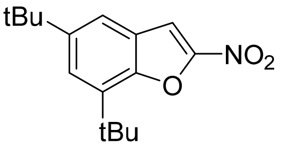

From (*E*)-4,6-di(*tert*-butyl)-2-(2-nitrovinyl)phenol (408 mg, 1.47 mmol), 171 mg (42%) of **1n** were obtained after column chromatography eluting with hexane:EtOAc (98:2). Light brown solid; m. p. 82.8-84.3 °C; **^{1}H NMR** (300 MHz, $CDCl_3$) δ 7.61 (s, 1H), 7.55 (s, 2H), 1.53 (s, 9H), 1.38 (s, 9H); **^{13}C NMR** (75 MHz, $CDCl_3$) δ 149.9 (C), 148.6 (C), 135.7 (C), 126.4 (C), 125.1 (CH), 117.5 (CH), 107.6 (CH), 35.3 (C), 34.8 (C), 31.7 (CH_3), 29.9 (CH_3).

5,7-Dichloro-2-nitrobenzofuran (1o)[2]

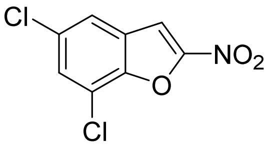

From (*E*)-4,6-dichloro-2-(2-nitrovinyl)phenol (312 mg, 1.33 mmol), 200 mg (65%) of **1o** were obtained after column chromatography eluting with hexane:EtOAc (97:3). **^{1}H NMR** (300 MHz, $CDCl_3$) δ 7.67 (d, *J* = 1.9 Hz, 1H), 7.64 (s, 1H), 7.61 (d, *J* = 1.9 Hz, 1H); **^{13}C NMR** (75 MHz, $CDCl_3$) δ 147.9 (C), 131.5 (C), 130.1 (CH), 127.9 (C), 122.0 (CH), 119.3 (C), 106.8 (CH).

5-(Phenylethynyl)-2-nitrobenzofuran (1g)[2]

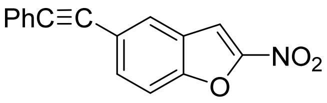

A solution of 5-bromo-2-nitrobenzofuran (**1d**, 160 mg, 0.66 mmol, 1.0 equiv) in degassed trimethylamine (2 mL) was added to a mixture of $Pd(PPh_3)_2Cl_2$ (10 mg, 0.014 mmol, 0.02 equiv), CuI (6 mg, 0.028 mmol, 0.04 equiv) and PPh_3 (8 mg, 0.028 mmol, 4 mol%) under argon. Then, degassed phenylacetylene (80 µL, 0.70 mmol, 1.1 equiv) was added. The resulting mixture was heated at 60 °C overnight under argon. A saturated aqueous NH_4Cl solution (15 mL) was added. The mixture was extracted with ethyl acetate (3 × 15 mL). The combined organic layers were washed with water (15 mL) and brine (15 mL). The organic phase was dried over anhydrous $MgSO_4$ and concentrated. Purification by column chromatography on silica gel (eluent: hexane:dicloromethane = 9:1) yielded 40.6 mg (23%) of compound **1g.** Yellow solid; **^{1}H NMR** (300 MHz, $CDCl_3$) δ 7.94 (dd, J = 1.7, 0.7 Hz, 1H), 7.74 (dd, *J* = 8.7, 1.7 Hz, 1H), 7.65 (d, *J* = 0.9 Hz, 1H), 7.60 (dt, *J* = 8.8, 0.8 Hz, 1H), 7.58 – 7.51 (m, 2H), 7.41 – 7.33 (m, 3H); **^{13}C NMR** (75 MHz, $CDCl_3$) δ 152.8 (C), 133.5 (CH), 131.8 (CH), 128.8 (CH), 128.6 (CH), 127.2 (CH), 126.1 (C), 122.8 (C), 121.1 (C), 113.1 (CH), 107.0 (CH), 90.2 (C), 88.1 (C).

Synthesis and characterization data of 2-nitrobenzothiophene

2-Nitrobenzothiophene was synthesized via a two-step procedure described in the literature.[2]

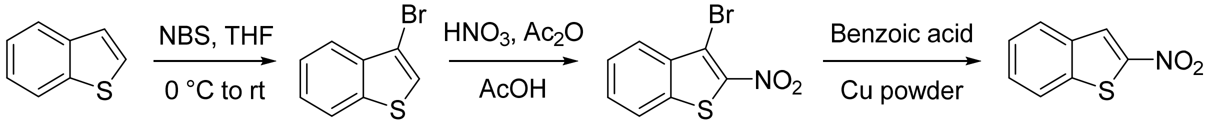

3-Bromo-2-nitrobenzo[*b*]thiophene[2]

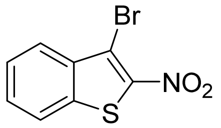

To a solution of thianaphthene (2.5 g, 18.6 mmol, 1.0 equiv) in THF (75 mL, 0.25 M) under nitrogen atmosphere was added NBS (5.0 g, 27.9 mmol, 1.5 equiv) portionwise at 0 °C. The reaction was stirred at 0 °C for 30 min and then stirred at room temperature for 48 h (followed by TLC analysis). The reaction was quenched with saturated aqueous $Na_2S_2O_3$ solution (10 mL), and the mixture changed color from orange to colorless. The phases were separated and the aqueous phase was extracted with Et_2O (3×20 mL). The combined organic phases were washed with brine (2×15 mL), dried under Na_2SO_4, filtered and concentrated under reduced pressure. A brownish oil was obtained, which was used without further purification for the next step.

To a solution of the previously obtained crude product in Ac_2O (56 mL, 0.33 M) at 0 °C under nitrogen atmosphere, was added a mixture of 60% HNO_3 (7.8 mL, 95 mmol, 5.1 equiv) and AcOH (6.3 mL, 110 mmol, 5.9 equiv). The reaction was then warmed to room temperature and stirred for 2 h. A yellow precipitate was observed. The reaction was poured into 100 mL ice. The solid was filtered and washed with cold water to obtain 2.0 g (42%, over two steps) of the title compound. Yellow solid; **^{1}H NMR** (300 MHz, $CDCl_3$) δ 8.07– 8.00 (m, 1H), 7.84 – 7.79 (m, 1H), 7.70 – 7.52 (m, 2H); **^{13}C NMR** (75 MHz, $CDCl_3$) δ 137.2 (C), 137.1 (C), 130.5 (CH), 126.9 (CH), 126.9 (CH), 123.0 (CH), 111.4 (C).

2-Nitrobenzo[*b*]thiophene[2]

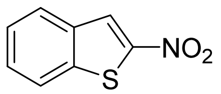

A mixture of 3-bromo-2-nitrobenzo[*b*]thiophene (0.70 g, 2.71 mmol, 1.0 equiv), benzoic acid (1.17 g, 9.60 mmol, 3.54 equiv) and Cu powder (0.80 g, 12.6 mmol, 4.65 equiv), under nitrogen atmosphere was heated at 150 °C (oil bath) and stirred for 30 min. The melted mixture was allowed to stand at room temperature until solidification. The solid was dissolved in dichloromethane (15 mL) and passed through a short-path silica gel column, eluting with dichloromethane (100 mL). The filtrate was washed with saturated aqueous $NaHCO_3$ solution (2×40 mL) and the organic phase changed color from green to yellow. The organic phase was washed with brine (2×40 mL), dried over Na_2SO_4, and concentrated by reduced pressure. 433 mg (89%) of the title compound were obtained without further purification. Yellow solid; **^{1}H NMR** (500 MHz, $CDCl_3$) δ 8.21 (s, 1H), 7.93 (d, *J* = 8.1 Hz, 1H), 7.84 (d, *J* = 8.3 Hz, 1H), 7.58 (ddd, *J* = 8.3, 7.1, 1.2 Hz, 1H), 7.49 (ddd, *J* = 8.2, 7.1, 1.1 Hz, 1H); **^{13}C NMR** (75 MHz, $CDCl_3$) δ 140.5 (C), 136.3 (C), 129.3 (CH), 127.2 (CH), 126.3 (CH), 125.8 (CH), 123.1 (CH).

5-Acetoxy-3-nitrobenzofuran[6]

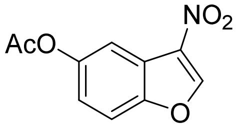

A solution of commercially available 5-hydroxy-3-nitrobenzofuran (90 mg, 0.5 mmol) in acetic anhydride (3.5 mL, 0.15 M) and concentrated H_2SO_4 (2 drops) was stirred overnight at room temperature. The reaction mixture was poured onto ice (ca. 30 g) and extracted with EtOAc (3×50 mL). The organic layer was washed with 5% aqueous $NaHCO_3$ (20 mL) and brine (20 mL), and dried over Na_2SO_4. After filtration and removal of the solvent under reduced pressure, column chromatography eluting with hexane:EtOAc (8:2) gave 60 mg (54%) of the title compound. **^{1}H NMR** (300 MHz, $CDCl_3$) δ 8.59 (s, d, J = 0.6 Hz, 1H), 7.89 (dd, J = 2.4, 0.6 Hz, 1H), 7.58 (dd, J = 9.0, 0.6 Hz, 1H), 7.20 (ddd, J = 9.0, 2.4, 0.3 Hz, 1H); **^{13}C NMR** (75 MHz, $CDCl_3$) δ 169.7 (C), 152.4 (C), 148.7 (C), 158.07 (CH), 121.5 (CH), 120.0 (C), 114.2 (CH), 113.3 (CH), 21.12 (CH_3).

Synthesis and characterization data for isocyanides 2

Methyl 2-isocyano-2-phenylacetate (2a)[7]

A two-step procedure described in the literature was carried out.[7]

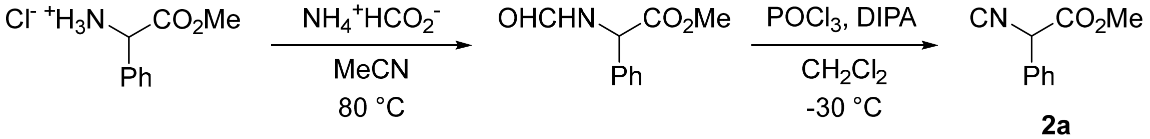

2a

To a suspension of (S)-(+)-2-phenylglycine methyl ester hydrochloride (2.0 g, 9.92 mmol) and (R)-(-)-2-phenylglycine methyl ester hydrochloride (2.0 g, 9.92 mmol) in acetonitrile (17 mL, 1.1 M) was added ammonium formate (3.2 g, 50 mmol, 2.5 equiv) and the mixture was stirred overnight at 80 °C. After reaction completion (TLC analysis), EtOAc (150 mL) and H_2O (40 mL) were added to the mixture. Phases were separated and the aqueous phase was extracted with EtOAc (3×25 mL). Combined organic phases washed with brine (2×40 mL), dried over Na_2SO_4, and concentrated under reduced pressure to afford crude formamide product as an orange oil, which was used for the next step without further purification.

To a solution of crude formamide (3.44 g, 17.8 mmol, 1 equiv) in dichloromethane (60 mL, 0.3 M) was added diisopropylamine (6.8 mL, 48.1 mmol, 2.7 equiv). The mixture was cooled to -30 °C and phosphoryl chloride was added dropwise (1.8 mL, 19.6 mmol, 1.1 equiv). The reaction was stirred at -30 °C until complete consumption of the starting material (TLC, *ca.* 2 h). The reaction was quenched with saturated aqueous Na_2CO_3 solution (37 mL). The phases were separated and the aqueous phase was extracted with dichloromethane (2×20 mL). The combined organic phases were washed with brine (2×40 mL), dried over Na_2SO_4 and concentrated under reduced pressure. Purification by column chromatography (eluent: hexane:EtOAc 8:2) furnished 2.23 g (64%, over two steps) of the title compound. Light yellow liquid; **^{1}H NMR** (300 MHz, $CDCl_3$) δ 7.52 – 7.38 (m, 5H), 5.37 (s, 1H), 3.79

(s, 3H); ^{13}**C NMR** (75 MHz, $CDCl_3$) δ 166.2 (C, C=O), 161.5 (C, NC), 131.9 (C), 129.7 (CH), 129.3 (CH), 126.8 (CH), 60.4 (CH), 53.9 (CH_3).

Synthetic procedure for methyl 2-isocyano-2-(4-methoxyphenyl)acetate

A three-step procedure was carried out.

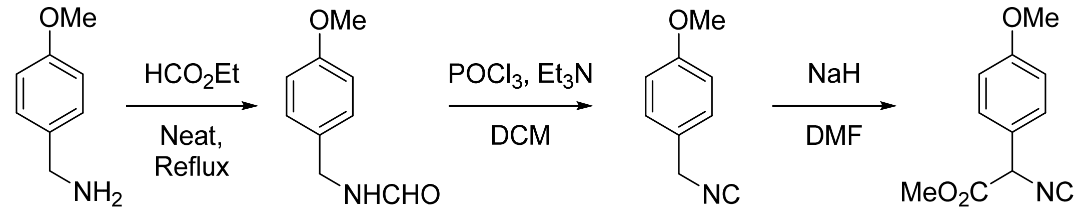

2b

N-(4-Methoxybenzyl)formamide[8]

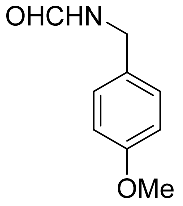

A literature procedure was carried out.[8] A mixture of 4-methoxybenxylamine (7.8 mL, 60 mmol, 1.0 equiv) and ethyl formate (25 mL, 309 mmol, 5.0 equiv) was stirred overnight at reflux (oil bath at 60 °C). Hexane (10 mL) was added at room temperature and an off-white solid precipitated, which was filtered under vacuum and washed with cold hexane to afford 6.22 g (63%) of the title compound as a 84:16 isomeric mixture; ^{1}**H NMR** (300 MHz, $CDCl_3$, for the major isomer) δ 8.19 (s, 1H, CHO), 7.19 (d, J = 8.6 Hz, 2H, Ar), 6.85 (d, J = 8.6 Hz, 2H, Ar), 6.06 (s, 1H, NH), 4.38 (d, J = 5.8 Hz, 2H, CH_2Ar), 3.78 (s, 3H, MeO); ^{13}**C NMR** (75 MHz, $CDCl_3$, for the major isomer) δ 161.1 (C, CHO), 159.2 (C), 130.2 (C), 129.3 (C), 114.2 (C), 55.4 (CH_3), 41.7 (CH_2).

1-(Isocyanomethyl)-4-methoxybenzene[8]

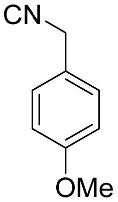

A literature procedure was carried out.[8] Triethylamine (7.9 mL, 56.4 mmol, 3.0 equiv) was added to a solution of *N*-(4-methoxymethyl)formamide (3.11 g, 18.8 mmol, 1.0 equiv) in dichloromethane (24 mL, 0.78 M), under nitrogen atmosphere. The mixture was cooled to -78 °C and phosphoryl oxychloride (2.19 mL, 23.5 mmol, 1.25 equiv) was added dropwise. The mixture was stirred at -78 °C for 5 min and then stirred at room temperature for 1 h, until complete consumption of the starting material (TLC). The reaction was quenched with saturated aqueous $NaHCO_3$ solution (24 mL). The phases were separated and the aqueous phase was extracted with dichloromethane (3×25 mL). The combined organic phases were washed with brine (25 mL), dried over Na_2SO_4 and concentrated under reduced pressure. Purification by column chromatography (eluent: hexane:EtOAc 85:15) yielded 2.66 g (96%) of the title compound as a colorless liquid. ^{1}**H NMR** (400 MHz, $CDCl_3$) δ 7.26 (d, J = 8.9 Hz, 2H), 6.92 (d, J = 8.7 Hz, 2H), 4.57 (t, J = 2.2 Hz, 2H), 3.82 (s, 3H); ^{13}**C NMR** (101 MHz, $CDCl_3$) δ 159.8 (C), 157.1 (C, NC), 128.3 (CH), 124.6 (C), 114.5 (CH), 55.5 (CH_3), 45.2 (CH_2).

Methyl 2-isocyano-2-(4-methoxyphenyl)acetate (2b)[7]

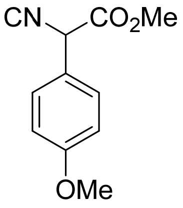

A modification of a literature procedure was employed.[7] To a solution of 1-(isocyanomethyl)-4-methoxybenzene (2.66 g, 18.1 mmol, 1.0 equiv) in DMF (60 mL, 0.3 M) under nitrogen atmosphere was added NaH (60% dispersion in mineral oil, 1.16 g, 29.0 mmol, 1.6 equiv) portionwise, and dimethylcarbonate (1.83 mL, 21.7 mmol, 1.2 equiv) was added to the resulting mixture. The mixture was stirred at room temperature for 1 h. The reaction was quenched with saturated aqueous NH_4Cl at 0 °C. Water (100 mL) was added and the mixture was extracted with Et_2O (3×150 mL). The organic phase was washed with brine (3×100 mL), dried over Na_2SO_4 and concentrated under reduced pressure. Purification by column chromatography (eluent: hexane:EtOAc 9:1) afforded 506 mg (14%) of the title compound as a light yellow liquid. **^{1}H NMR** (300 MHz, $CDCl_3$) δ 7.38 (d, J = 8.5 Hz, 2H, Ar), 6.93 (d, J = 8.8 Hz, 2H, Ar), 5.30 (s, 1H, CHAr), 3.82 (s, 3H, MeO), 3.78 (s, 3H, MeO); **^{13}C NMR** (75 MHz, $CDCl_3$) δ 166.5 (C), 161.1 (C), 160.6 (C), 128.2 (CH), 124.0 (C), 114.7 (CH), 59.8 (CH), 55.5 (CH_3), 53.8 (CH_3).

Synthetic procedure for methyl 2-(4-chlorophenyl)-2-isocyanoacetate

A three-step procedure was carried out.

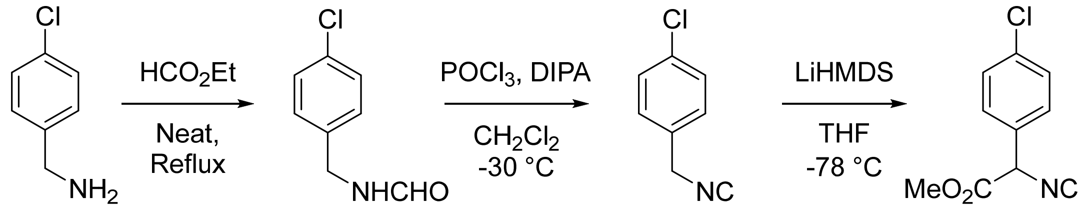

2c

***N*-(4-Chlorobenzyl)formamide[9]**

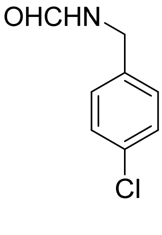

A modification of a literature procedure was carried out.[9] A mixture of 4-chlorobenzylamine (7.3 mL, 60 mmol, 1.0 equiv) and ethyl formate (25 mL, 309 mmol, 5.0 equiv) was stirred overnight at reflux (oil bath at 60 °C). The mixture was left to stand at room temperature and a white precipitate was observed. Hexane (10 mL) was added to promote total precipitation of the product, which was filtered under vacuum and washed with cold hexane to afford 8.1 g (79%) of the title compound as a 87:13 isomeric mixture. White solid; **^{1}H NMR** (300 MHz, $CDCl_3$, for the major isomer) δ 8.23 (s, 1H, CHO), 7.29 (d, J = 8.5 Hz, 2H, Ar), 7.20 (d, J = 8.7 Hz, 2H, Ar), 6.15 (s, 1H, NH), 4.42 (d, J = 6.1 Hz, 2H, CH_2Ar); **^{13}C NMR** (75 MHz, $CDCl_3$, for the major isomer) δ 161.2 (C), 136.3 (C), 133.6 (C), 129.2 (CH), 129.0 (CH), 41.6 (CH_2).

1-Chloro-4-(isocyanomethyl)benzene[10]

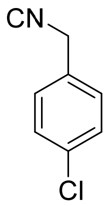

A modification of a literature procedure was employed.[7] To a solution of *N*-(4-chlorobenzyl)formamide (3.0 g, 17.7 mmol, 1.0 equiv) in CH_2Cl_2 (60 mL, 0.3 M), under nitrogen atmosphere, was added diisopropylamine (6.7 mL, 47.8 mmol, 2.7 equiv). The mixture was cooled to -30 °C and $POCl_3$ (1.82 mL, 19.5 mmol, 1.1 equiv) was added dropwise. The reaction was stirred at -30 °C for 2 h (TLC analysis) and then quenched with saturated aqueous Na_2CO_3 (30 mL). The phases were separated and the aqueous phase was extracted with dichloromethane (2×20 mL). Combined organic phases were washed with brine (20 mL), dried over Na_2SO_4 and concentrated under reduced pressure. Purification by column chromatography (eluent: hexane:EtOAc 85:15) afforded 2.22 g (83%) of the title compound. Colorless liquid; **^{1}H NMR** (300 MHz, $CDCl_3$) δ 7.38 (d, *J* = 8.5 Hz, 2H, Ar), 7.29 (d, *J* = 8.7 Hz, 2H, Ar), 4.62 (s, 2H, CH_2Ar); **^{13}C NMR** (75 MHz, $CDCl_3$) δ 158.4 (C, NC), 134.6 (C), 130.9 (C), 129.3 (CH), 128.1 (CH), 45.1 (CH_2). This compound was quite volatile and was not subjected to exhaustive drying. It contains traces of EtOAc from the eluent in the chromatographic column. It was used in this way for the next step.

Methyl 2-(4-chlorophenyl)-2-isocyanoacetate (2c)[11]

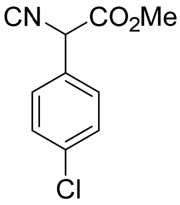

A modification of a literature procedure was performed.[7] To a solution of 1-chloro-4-(isocyanomethyl)benzene obtained in the previous step (2.22 g, 14.6 mmol, 1.0 equiv, contains EtOAc) in THF (50 mL, 0.3 M) under nitrogen atmosphere was added dropwise LiHMDS (17.5 mL, 17.5 mmol, 1.2 equiv, 1.0 M sol. in THF) at -78 °C. After 5 min of stirring, dimethylcarbonate (1.5 mL, 17.5 mmol, 1.2 equiv) was added dropwise at -78 °C, and the reaction was stirred at that temperature for 1.5 h. The reaction was quenched with saturated aqueous NH_4Cl solution and extracted with EtOAc (3×100 mL). Combined organic phases were washed with brine (40 mL), dried over Na_2SO_4 and concentrated under reduced pressure. Purification by column chromatography (eluent: hexane:EtOAc 95:5) yielded 477 mg (16%) of the title compound. Light yellow liquid; **^{1}H NMR** (300 MHz, $CDCl_3$) δ 7.45 – 7.37 (m, 4H), 5.35 (s, 1H), 3.80 (s, 3H); **^{13}C NMR** (75 MHz, $CDCl_3$) δ 165.8 (C, C=O), 162.2 (C, NC), 135.9 (C), 130.3 (C), 129.6 (CH), 128.2 (CH), 59.7 (CH), 54.1 (MeO).

Methyl 2-isocyano-2-(4-nitrophenyl)acetate (2d)[12]

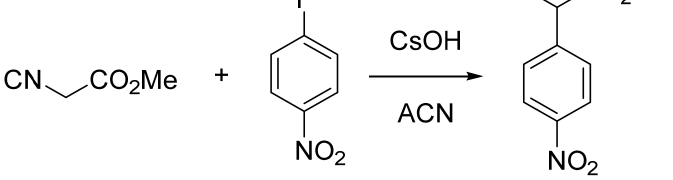

2d

A modification of a literature procedure was carried out.[12] To a solution of methyl isocyanoacetate (0.45 mL, 5.0 mmol, 1.0 equiv) in acetonitrile (5 mL, 1M) under nitrogen atmosphere was added CsOH·x H_2O (15-20% H_2O) (1.20 g, 8.0 mmol, 1.6 equiv) and 1-fluoro-4-nitrobenzene (0.58 mL, 5.5 mmol, 1.1 equiv). The reaction mixture was stirred overnight. EtOAc (150 mL) was added and the mixture was washed with 0.1 M HCl (80 mL, 1.6 equiv), the organic phase changed color from purple to orange. The phases were separated and the aqueous phase was extracted with EtOAc (3×50 mL). The combined organic phases were dried over Na_2SO_4, concentrated under reduced pressure and purified by column chromatography (eluent: hexane:EtOAc 8:2) to afford 293 g (27%) of the title compound. Light yellow solid; **^{1}H NMR** (300 MHz, $CDCl_3$) δ 8.31 (d, *J* = 8.9 Hz, 2H), 7.70 (d, *J* = 8.5 Hz, 2H), 5.51 (s, 1H), 3.83 (s, 3H); **^{13}C NMR** (75 MHz, $CDCl_3$) δ 165.0 (C, C=O), 163.7 (C, NC), 148.2 (C), 138.1 (C), 128.0 (CH), 124.5 (CH), 54.4 (CH_3), 53.5 (CH).

Synthesis and characterization data for catalyst III

A modification of a two-step procedure described in the literature was carried out.[13]

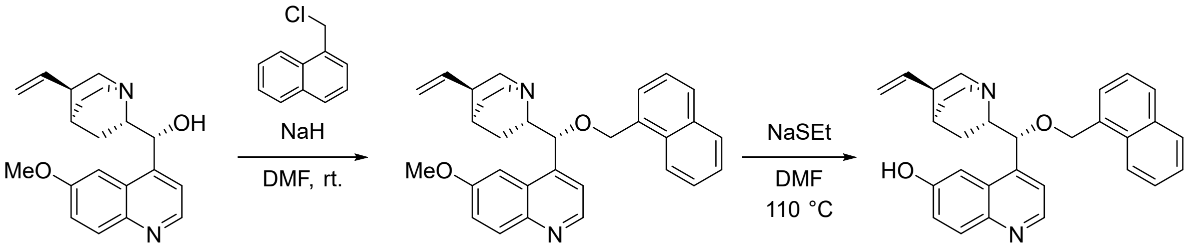

To a solution of quinine (1.0 g, 3.08 mmol, 1.0 equiv) in DMF (9 mL, 0.3 M) under nitrogen atmosphere was added NaH (60% dispersion in mineral oil, 0.32 g, 8.02 mmol, 2.6 equiv) portionwise and the mixture was stirred at room temperature for 2 h. A solution of 1-(chloromethyl)naphthalene (0.6 g, 3.38 mmol, 1.1 equiv) in DMF (1 mL) was added dropwise and the reaction was stirred for 24 h at room temperature, until complete consumption of the starting material (TLC). The reaction was quenched with brine (10 mL) and the mixture was extracted with ethyl acetate (5×30 mL). The organic phase was washed with water (5×25 mL) and brine (25 mL). Dried over Na_2SO_4 and concentrated under reduced pressure to obtain a light-yellow foam that was used without further purification for the next step.

A solution of the crude product previously obtained and sodium ethanethiolate (1.0 g, 12.5 mmol, 4.0 equiv) in DMF (19 mL, 0.16 M) under nitrogen atmosphere was stirred overnight at 110 °C (oil bath) until complete consumption of the starting material (TLC). The reaction was cooled to room temperature and quenched with saturated aqueous NH_4Cl (20 mL) and water (15 mL). The solution was acidified to pH 2 by dropwise addition of conc. HCl. The aqueous solution was washed with EtOAc (2×30 mL) and the organic phase was extracted with aq. HCl 1 M solution (2×15 mL). Combined aqueous phases were brought to pH 8 by the addition of conc. NH_3. The basified aqueous phase was extracted with EtOAc (3×100 mL). Purification by column chromatography (eluent: EtOAc:MeOH 9:1)

yielded 726 mg (52%) of **III**. Grayish solid; m. p. 141.0-141.9 °C; [α]D^{25} –15.8 (*c* 1.0, $CHCl_3$); **^{1}H NMR** (300 MHz, $CDCl_3$): 8.67 (d, *J* = 4.5 Hz, 1H, CH-N), 8.12-8.05 (br s, 2H, Ar), 8.04 (d, *J* = 9.0 Hz, 1H, Ar), 7.96 – 7.82 (m, 2H, Ar), 7.92 – 7.81 (m, 2H, Ar), 7.79 (d, *J* = 8.2 Hz, 1H, Ar), 7.63 – 7.36 (m, 5H, Ar), 7.32 (dd, *J* = 9.1, 2.4 Hz, 1H, Ar), 5.78 (bs, 1H), .58 (ddd, *J* = 17.5, 10.3, 7.5 Hz, 1H), 4.98 – 4.78 (m, 3H), 3.70 – 3.47 (m, 1H), 3.27 – 3.00 (m, 2H), 2.85 – 2.55 (m, 2H), 2.38 – 2.24 (m, 1H), 2.16 – 1.96 (m, 1H), 1.90 – 1.68 (m, 2H), 1.59 – 1.40 (m, 2H); **^{13}C NMR** (75 MHz, $CDCl_3$) δ 157.0 (C), 146.8 (CH), 144.0 (C), 143.4 (C), 140.4 (CH), 133.67 (C), 133.5 (C), 131.67 (CH), 131.3 (C), 128.8 (CH), 128.6 (CH), 127.9 (C), 126.4 (CH), 126.0 (CH), 125.8 (C), 125.5 (CH), 123.6 (CH), 123.3 (CH), 115.4 (CH_2), 106.6 (CH), 69.3 (CH_2), 59.8 (CH), 56.3 (CH_2), 43.3 (CH_2), 39.2 (CH), 27.7 (CH), 26.7 (CH_2); **HRMS** (ESI) *m/z*: 451.2369 $[M+H]^+$, $C_{30}H_{31}N_2O_2^+$ requires 451.2380.

Synthesis and characterization data for compounds 3

Enantioselective procedure

To a solution of the corresponding 2-nitrobenzofuran **1** (0.15 mmol, 1.0 equiv) and **III** (6.8 mg, 0.015 mmol, 0.1 equiv) in dry $CHCl_3$ (0.75 mL, 0.2 M) at 0 °C was added 2-phenylisocyanoacetate (**2a**, 28 µL, 0.19 mmol, 1.3 equiv). The reaction was stirred until complete consumption of compound **1** or until no more reaction advancement was observed (TLC analysis).

For the determination of the diastereomeric ratio, a short path column chromatography to separate the catalyst **III** was carried out and the resulting mixture analyzed by ^{1}H NMR. Then the product was purified by column chromatography, eluting with hexane:EtOAc mixtures.

Non-enantioselective procedure

To a solution of the corresponding 2-nitrobenzofuran **1** (0.1 mmol, 1.0 equiv) and silver oxide (1.2 mg, 0.005 mmol, 0.05 equiv) in dichloromethane (1 mL, 0.1 M) at room temperature was added 2-phenylisocyanoacetate (**2a**, 19 µL, 0.13 mmol, 1.3 equiv) The reaction was stirred at room temperature for 17 h and the product was purified by column chromatography, eluting with hexane:EtOAc mixtures.

Methyl (1*R*,3a*R*,8b*R*)-3a-nitro-1-phenyl-3a,8b-dihydro-1*H*-benzofuro[2,3-*c*]pyrrole-1-carboxylate (3aa)

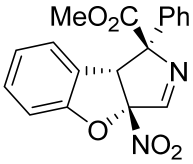

40.1 mg (79%) of **3aa** were obtained from **1a** (24.5 mg, 0.15 mmol) after column chromatography eluting with hexane:EtOAc (95:5). Enantiomeric excess (93%) was measured by HPLC (CHIRALPAK® AD-H), hexane:*i*PrOH 80:20, 1.0 mL min^{-1}, major enantiomer: t_r = 8.5 min, minor enantiomer: t_r = 24.1 min.

White foam; $[\alpha]D^{25}$ +120.5 (*c* 1.0, $CHCl_3$); **^{1}H NMR** (300 MHz, $CDCl_3$) δ 8.09 (s, 1H, CH=N), 7.23 – 7.16 (m, 3H, Ar), 7.07 (dddd, *J* = 8.2, 7.4, 1.4, 0.7 Hz, 1H, Ar), 6.96 – 6.91 (m, 2H, Ar), 6.87 (ddt, *J* = 8.3, 1.1, 0.6 Hz, 1H, Ar), 6.63 (td, *J* = 7.6, 1.0 Hz, 1H, Ar), 6.39 (dq, *J* = 7.6, 0.7 Hz, 1H, Ar), 5.37 (s, 1H), 3.91 (s, 3H, MeO); **^{13}C NMR** (75 MHz, $CDCl_3$) δ 171.1 (C, C=O), 158.7 (CH, C=N), 157.1 (C), 135.7 (C), 129.9 (CH), 128.7 (CH), 128.7 (CH), 126.5 (CH), 125.9 (CH), 124.1 (C), 123.1 (CH), 122.5 (CH), 110.3 (CH), 91.8 (C), 59.2 (CH_3), 54.1 (CH); **HRMS** (ESI) *m/z*: 339.0971 $[M+H]^+$, $C_{18}H_{15}N_2O_5^+$ requires 339.0975.

Methyl (1*R*,3a*R*,8b*R*)-7-methyl-3a-nitro-1-phenyl-3a,8b-dihydro-1*H*-benzofuro[2,3-*c*]pyrrole-1-carboxylate (3ba)

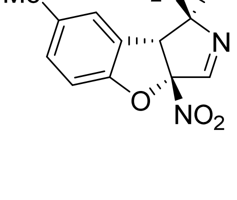

35.4 mg (67%) of **3ba** were obtained from **1b** (26.6 mg, 0.15 mmol) after column chromatography eluting with hexane:EtOAc (9:1). Enantiomeric excess (88%) was measured by HPLC (CHIRALPAK® AD-H), hexane:*i*PrOH 80:20, 1.0 mL min^{-1}, major enantiomer: t_r = 7.3 min, minor enantiomer: t_r = 27.0 min.

Colorless oil; [α]D^{25} +20.3 (*c* 0.5, $CHCl_3$); **^{1}H NMR** (300 MHz, $CDCl_3$) δ 8.07 (s, 1H, CH=N), 7.23 – 7.17 (m, 3H, Ar), 6.96 – 6.90 (m, 2H, Ar), 6.85 (ddt, *J* = 8.3, 1.8, 0.8 Hz, 1H, Ar), 6.74 (d, *J* = 8.3 Hz, 1H, Ar), 6.15 (s, 1H, Ar), 5.30 (s, 1H, CHAr), 3.91 (s, 3H, MeO), 1.96 (s, 3H, MeO); **^{13}C NMR** (75 MHz, $CDCl_3$) δ 171.1 (C, C=O), 158.8 (CH, C=N), 155.2 (C), 135.8 (C), 132.6 (C), 130.2 (CH), 128.6 (CH), 128.5 (CH), 126.9 (CH), 126.0 (CH), 124.3 (C), 122.4 (C), 109.7 (CH), 91.7 (C), 59.2 (CH_3), 54.0 (CH), 20.6 (CH_3); **HRMS** (ESI) *m/z*: 353.1128 $[M+H]^+$, $C_{19}H_{17}N_2O_5^+$ requires 353.1132.

Methyl (1*R*,3a*R*,8b*R*)-7-methoxy-3a-nitro-1-phenyl-3a,8b-dihydro-1*H*-benzofuro[2,3-*c*]pyrrole-1-carboxylate (3ca)

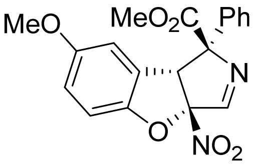

34.0 mg (62%) of **3ca** were obtained from **1c** (29.0 mg, 0.15 mmol) after column chromatography eluting with hexane:EtOAc (9:1). Enantiomeric excess (91%) was measured by HPLC (CHIRALPAK® AD-H), hexane:*i*PrOH 80:20, 1.0 mL min^{-1}, major enantiomer: t_r = 10.4 min, minor enantiomer: t_r = 23.3 min.

Orange oil; [α]D^{25} +6.7 (*c* 0.8, $CHCl_3$); **^{1}H NMR** (300 MHz, $CDCl_3$) δ 8.09 (s, 1H, CH=N), 7.28 (dd, *J* = 6.5, 3.2 Hz, 3H, Ar), 7.06 – 6.96 (m, 2H, Ar), 6.81 (d, *J* = 8.9 Hz, 1H, Ar), 6.65 (ddd, *J* = 8.9, 2.7, 0.8 Hz, 1H, Ar), 5.88 (dd, *J* = 2.7, 1.0 Hz, 1H), 5.36 (s, 1H, CHAr), 3.95 (s, 3H, MeO), 3.45 (s, 3H, MeO); **^{13}C NMR** (75 MHz, $CDCl_3$) δ 171.1 (C, C=O), 158.9 (CH, CH=N), 155.7 (C), 151.1 (C), 135.7 (C), 128.8 (CH), 128.7 (CH), 126.1 (CH), 124.6 (C), 123.2 (C), 116.6 (CH), 110.9 (CH), 110.7 (CH), 91.6 (C), 59.4 (CH_3), 55.9 (CH_3), 54.1 (CH); **HRMS** (ESI) *m/z*: 369.1079 $[M+H]^+$, $C_{19}H_{17}N_2O_6^+$ requires 369.1081.

Methyl (1*R*,3a*R*,8b*R*)-7-bromo-3a-nitro-1-phenyl-3a,8b-dihydro-1*H*-benzofuro[2,3-*c*]pyrrole-1-carboxylate (3da)

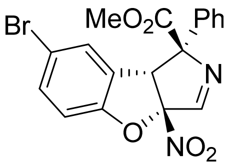

46.5 mg (74%) of **3da** were obtained from **1d** (36.5 mg, 0.15 mmol) after column chromatography eluting with hexane:EtOAc (95:5). Enantiomeric excess (92%) was measured by HPLC (CHIRALPAK® AD-H), hexane:*i*PrOH 80:20, 1.0 mL min^{-1}, major enantiomer: t_r = 8.7 min, minor enantiomer: t_r = 28.2 min.

Colorless oil; [α]D^{25} –21.2 (*c* 1.0, $CHCl_3$); **^{1}H NMR** (300 MHz, $CDCl_3$) δ 8.08 (s, 1H, CH=N), 7.28 – 7.23 (m, 3H, Ar), 7.17 (ddd, *J* = 8.7, 2.1, 0.8 Hz, 1H, Ar), 6.96 – 6.87 (m, 2H, Ar), 6.75 (d, *J* = 8.6 Hz, 1H, Ar), 6.48 (dd, *J* = 2.2, 1.0 Hz, 1H, Ar), 5.33 (s, 1H, CHAr), 3.91 (s, 3H, MeO); **^{13}C NMR** (75 MHz, $CDCl_3$) δ 170.8 (C, C=O), 158.4 (CH, CH=N), 156.1 (C), 135.3 (C), 132.7 (CH), 129.5 (CH), 129.1 (CH), 128.8 (CH), 125.7 (CH), 124.8 (C), 124.1 (C), 115.3 (C), 111.8, 91.9 (C), 58.9 (CH_3), 54.2 (CH); **HRMS** (ESI) *m/z*: 417.0079 $[M+H]^+$, $C_{18}H_{14}BrN_2O_5^+$ requires 417.0081.

Methyl (1*R*,3a*R*,8b*R*)-3a,7-dinitro-1-phenyl-3a,8b-dihydro-1*H*-benzofuro[2,3-*c*]pyrrole-1-carboxylate (3ea)

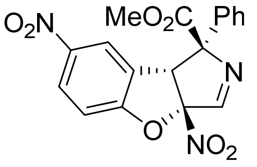

58.5 mg (99%) of **3ea** were obtained from **1e** (31.2 mg, 0.15 mmol) after column chromatography eluting with hexane:EtOAc (9:1 to 8:2). Enantiomeric excess (92%) was measured by HPLC (CHIRALPAK® AD-H), hexane:*i*PrOH 80:20, 1.0 mL min^{-1}, major enantiomer: t_r = 19.4 min, minor enantiomer: t_r = 32.7 min.

Orange oil; $[\alpha]D^{25}$ –41.6 (*c* 1.0, $CHCl_3$); **^{1}H NMR** (300 MHz, $CDCl_3$) δ 8.14 (s, 1H, CH=N), 8.03 (ddd, *J* = 8.9, 2.4, 0.7 Hz, 1H, Ar), 7.32 (dd, *J* = 2.4, 1.1 Hz, 1H, Ar), 7.25 – 7.19 (m, 3H, Ar), 6.98 (d, *J* = 9.0 Hz, 1H, Ar), 6.95 – 6.90 (m, 2H, Ar), 5.43 (s, 1H, CHAr), 3.93 (s, 3H, MeO); **^{13}C NMR** (75 MHz, $CDCl_3$) δ 170.4 (C, C=O), 161.2 (C), 157.9 (CH, C=N), 143.7 (C), 135.0 (C), 129.4 (CH), 129.1 (CH), 126.7 (CH), 125.6 (CH), 124.5 (C), 124.4 (C), 122.8 (CH), 110.6 (CH), 92.1 (C), 58.1 (CH_3), 54.3 (CH); **HRMS** (ESI) *m/z*: 384.0829 $[M+H]^+$, $C_{18}H_{14}N_3O_7^+$ requires 384.0826.

Methyl (1*R*,3a*R*,8b*R*)-7-methoxycarbonyl-3a-nitro-1-phenyl-3a,8b-dihydro-1H-benzofuro[2,3-c]pyrrole-1-carboxylate (3fa)

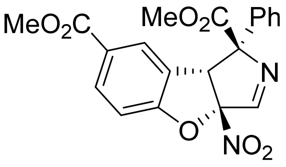

50.8 mg (85%) of **3fa** were obtained from **1f** (33.2 mg, 0.15 mmol) after column chromatography eluting with hexane:EtOAc (9:1). Enantiomeric excess (91%) was measured by HPLC (CHIRALPAK® IC), hexane:*i*PrOH 80:20, 1.0 mL min^{-1}, major enantiomer: t_r = 14.8 min, minor enantiomer: t_r = 23.5 min.

Colorless oil; $[\alpha]D^{25}$ –31.1 (*c* 1.0, $CHCl_3$); **^{1}H NMR** (300 MHz, $CDCl_3$) δ 8.11 (s, 1H, CH=N), 7.80 (ddd, *J* = 8.6, 1.8, 0.7 Hz, 1H, Ar), 7.24 – 7.10 (m, 4H, Ar), 6.97 – 6.85 (m, 3H, Ar), 5.37 (s, 1H, CHAr), 3.91 (s, 3H, MeO), 3.77 (s, 3H, MeO); **^{13}C NMR** (75 MHz, $CDCl_3$) δ 170.8 (C, C=O), 165.7 (C, C=O), 160.4 (C), 158.2 (CH, CH=N), 135.3 (C), 132.3 (CH), 128.9 (CH), 128.8 (CH), 128.5 (CH), 125.7 (CH), 125.5 (C), 124.3 (C), 123.2 (C), 110.1 (CH), 92.0 (C), 58.5 (CH), 54.1 (CH_3), 52.1 (CH_3); **HRMS** (ESI) *m/z*: 397.1023 $[M+H]^+$, $C_{20}H_{17}N_2O_7^+$ requires 397.1030.

Methyl (1*R*,3a*R*,8b*R*)-3a-nitro-1-phenyl-7-(phenylethynyl)-3a,8b-dihydro-1H-benzofuro[2,3-c]pyrrole-1-carboxylate (3ga)

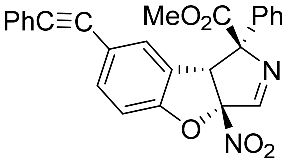

36.2 mg (55%) of **3ga** were obtained from **1g** (39.5 mg, 0.15 mmol) after column chromatography eluting with hexane:EtOAc (95:5). Enantiomeric excess (89%) was measured by HPLC (Lux® i-Amylose-1), hexane:*i*PrOH 80:20, 1.0 mL min^{-1}, major enantiomer: t_r = 12.5 min, minor enantiomer: t_r = 22.3 min.

Colorless oil; $[\alpha]D^{25}$ –88.5 (*c* 1.0, $CHCl_3$); **^{1}H NMR** (300 MHz, $CDCl_3$) δ 8.11 (s, 1H, CH=N), 7.47 – 7.38 (m, 2H, Ar), 7.37 – 7.28 (m, 3H, Ar), 7.30 – 7.24 (m, 1H, Ar), 7.24 (d, J = 2.2 Hz, 3H, Ar), 6.99 – 6.90 (m, 2H, Ar), 6.85 (d, J = 8.4 Hz, 1H, Ar), 6.60 – 6.57 (m, 1H, Ar), 5.35 (s, 1H, CHAr), 3.93 (s, 3H, MeO); **^{13}C NMR** (75 MHz, $CDCl_3$) δ 170.9 (C, C=O), 158.4 (CH, CH=N), 156.9 (C), 135.4 (C), 133.6 (CH), 131.6 (CH), 129.8 (CH), 129.0 (CH), 128.8 (CH), 128.5 (CH), 128.4 (CH), 125.8 (CH), 124.2 (C), 123.2 (C), 123.2 (C), 118.5 (C), 110.5 (CH), 91.9 (C), 88.7 (C), 88.2 (C), 59.0 (CH), 54.1 (CH_3); **HRMS** (ESI) *m/z*: 439.1283 $[M+H]^+$, $C_{26}H_{18}N_2O_5^+$ requires 439.1288.

Methyl (1*R*,3a*R*,8b*R*)-6-methoxy-3a-nitro-1-phenyl-3a,8b-dihydro-1*H*-benzofuro[2,3-*c*]pyrrole-1-carboxylate (3ha)

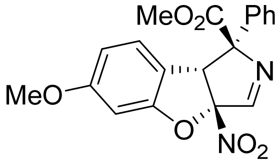

26.2 mg (47%) of **3ha** were obtained from **1h** (29.3 mg, 0.15 mmol) after column chromatography eluting with hexane:EtOAc (85:15). Enantiomeric excess (92%) was measured by HPLC (Lux® i-Amylose-1), hexane:*i*PrOH 80:20, 1.0 mL min^{-1}, major enantiomer: t_r = 10.0 min, minor enantiomer: t_r = 17.5 min.

Colorless oil; $[\alpha]D^{25}$ +94.1 (*c* 1.0, $CHCl_3$); **^{1}H NMR** (400 MHz, $CDCl_3$) δ 8.05 (s, 1H, CH=N), 7.25 – 7.20 (m, 3H, Ar), 6.99 – 6.94 (m, 2H, Ar), 6.43 (d, *J* = 2.2 Hz, 1H, Ar), 6.22 – 6.14 (m, 2H, Ar), 5.29 (s, 1H, CHAr), 3.89 (s, 3H, MeO), 3.67 (s, 3H, MeO); **^{13}C NMR** (101 MHz, $CDCl_3$) δ 171.1 (C, C=O), 161.4 (C), 158.7 (CH, CH=N), 158.3 (C), 135.9 (C), 128.7 (C), 126.6 (CH), 126.0 (CH), 124.8 (C), 114.2 (C), 109.5 (CH), 96.4 (CH), 91.5 (C), 58.8 (CH_3), 55.6 (CH_3), 54.0 (CH); **HRMS** (ESI) *m/z*: 369.1080 $[M+H]^+$, $C_{19}H_{17}N_2O_6^+$ requires 369.1081.

Methyl (1*R*,3a*R*,8b*R*)-6-chloro-3a-nitro-1-phenyl-3a,8b-dihydro-1*H*-benzofuro[2,3-*c*]pyrrole-1-carboxylate (3ia)

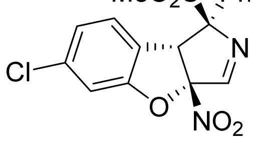

51.2 mg (92%) of **3ia** were obtained from **1i** (29.6 mg, 0.15 mmol) after column chromatography eluting with hexane:EtOAc (9:1). Enantiomeric excess (92%) was measured by HPLC (CHIRALPAK® AD-H), hexane:*i*PrOH 80:20, 1.0 mL min^{-1}, major enantiomer: t_r = 8.2 min, minor enantiomer: t_r = 14.0 min.

Colorless oil; $[\alpha]D^{25}$ +86.0 (*c* 1.0, $CHCl_3$); **^{1}H NMR** (300 MHz, $CDCl_3$) δ 8.08 (s, 1H), 7.28 – 7.20 (m, 3H), 6.96 – 6.91 (m, 2H), 6.90 (d, *J* = 1.9 Hz, 1H), 6.61 (dd, *J* = 8.2, 1.8 Hz, 1H), 6.28 (dd, *J* = 8.2, 1.1 Hz, 1H), 5.32 (s, 1H), 3.90 (s, 3H); **^{13}C NMR** (75 MHz, $CDCl_3$) δ 170.9 (C, C=O), 158.4 (CH, CH=N), 157.6 (C), 135.5 (C), 129.0 (CH), 128.9 (C), 128.9 (CH), 127.0 (CH), 125.9 (CH), 124.5 (C), 123.5, 121.3 (C), 111.3, 91.7 (C), 58.6 (CH_3), 54.1 (CH); **HRMS** (ESI) *m/z*: 373.0581 $[M+H]^+$, $C_{18}H_{14}ClN_2O_5^+$ requires 373.0586.

Methyl (1*R*,3a*R*,8b*R*)-5-methyl-3a-nitro-1-phenyl-3a,8b-dihydro-1*H*-benzofuro[2,3-*c*]pyrrole-1-carboxylate (3ja)

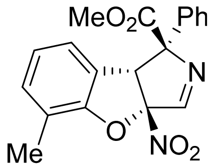

21.6 mg (41%) of **3ja** were obtained from **1j** (26.6 mg, 0.15 mmol) after column chromatography eluting with hexane:EtOAc (95:5). Enantiomeric excess (96%) was measured by HPLC (CHIRALPAK® AD-H), hexane:*i*PrOH 80:20, 1.0 mL min^{-1}, major enantiomer: t_r = 7.4 min, minor enantiomer: t_r = 19.3 min.

Colorless oil; [α]D^{25} +189.7 (*c* 1.0, $CHCl_3$); **^{1}H NMR** (300 MHz, $CDCl_3$) δ 8.09 (s, 1H, CH=N), 7.24 – 7.17 (m, 3H, Ar), 6.99 – 6.91 (m, 2H, Ar), 6.88 (ddt, *J* = 7.5, 1.4, 0.8 Hz, 1H, Ar), 6.52 (t, *J* = 7.6 Hz, 1H, Ar), 6.18 (d, *J* = 7.7 Hz, 1H, Ar), 5.35 (s, 1H, CHAr), 3.90 (s, 3H, MeO), 2.22 (s, 3H, MeO); **^{13}C NMR** (75 MHz, $CDCl_3$) δ 171.2 (C, C=O), 158.9 (CH, CH=N), 155.8 (C), 135.8 (C), 131.1 (CH), 128.7 (CH), 128.6 (CH), 126.0 (CH), 124.0 (C), 123.7 (CH), 122.9 (CH), 121.9 (C), 120.6 (CH), 91.6 (C), 59.5 (CH_3), 54.0 (CH), 15.0 (CH_3); **HRMS** (ESI) *m/z*: 353.1126 [M+H]$^+$, $C_{19}H_{17}N_2O_5^+$ requires 353.1132.

Methyl (1*R*,3a*R*,8b*R*)-5-methoxy-3a-nitro-1-phenyl-3a,8b-dihydro-1*H*-benzofuro[2,3-*c*]pyrrole-1-carboxylate (3ka)

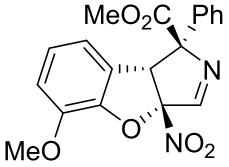

42.7 mg (77%) of **3ka** were obtained from **1k** (29.0 mg, 0.15 mmol) after column chromatography eluting with hexane:EtOAc (9:1). Enantiomeric excess (91%) was measured by HPLC (Lux® i-Amylose-1), hexane:*i*PrOH 80:20, 1.0 mL min^{-1}, major enantiomer: t_r = 11.8 min, minor enantiomer: t_r = 23.3 min.

White foam; [α]D^{25} +71.0 (*c* 1.1, $CHCl_3$); **^{1}H NMR** (300 MHz, $CDCl_3$) δ 8.09 (s, 1H, CH=N), 7.20 (ddd, *J* = 5.1, 2.6, 1.6 Hz, 3H, Ar), 6.99 – 6.90 (m, 2H, Ar), 6.66 (d, *J* = 8.1 Hz, 1H, Ar), 6.62 – 6.51 (m, 1H, Ar), 5.97 (dt, *J* = 7.6, 1.1 Hz, 1H, Ar), 5.38 (s, 1H, CHAr), 3.90 (s, 3H, MeO), 3.84 (s, 3H, MeO). **^{13}C NMR** (75 MHz, $CDCl_3$) δ 171.0 (C, C=O), 158.6 (CH, CH=N), 145.5 (C), 144.2 (C), 135.7 (C), 128.7 (CH), 128.6 (CH), 126.0 (CH), 124.3 (C), 123.8 (CH), 123.8 (C), 118.1 (CH), 112.7 (CH), 91.7 (C), 59.6 (CH_3), 56.2 (CH_3), 54.0 (CH); **HRMS** (ESI) *m/z*: 369.1080 [M+H]$^+$, $C_{19}H_{17}N_2O_6^+$ requires 369.1081.

Methyl (1*R*,3a*R*,8b*R*)-5-chloro-3a-nitro-1-phenyl-3a,8b-dihydro-1*H*-benzofuro[2,3-*c*]pyrrole-1-carboxylate (3la)

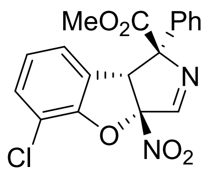

53.9 mg (96%) of **3la** were obtained from **1l** (26.9 mg, 0.15 mmol) after column chromatography eluting with hexane:EtOAc (9:1). Enantiomeric excess (96%) was measured by HPLC (CHIRALPAK® AD-H), hexane:*i*PrOH 80:20, 1.0 mL min^{-1}, major enantiomer: t_r = 8.5 min, minor enantiomer: t_r = 22.3 min.

White foam; [α]D^{25} +126.0 (*c* 1.0, $CHCl_3$); **^{1}H NMR** (300 MHz, $CDCl_3$) δ 8.14 (s, 1H, CH=N), 7.25 – 7.19 (m, 3H, Ar), 7.07 (dt, *J* = 8.1, 1.0 Hz, 1H, Ar), 7.00 – 6.87 (m, 2H, Ar), 6.57 (t, *J* = 7.9 Hz, 1H, Ar), 6.27 (dt, *J* = 7.7, 1.1 Hz, 1H, Ar), 5.42 (s, 1H), 3.91 (s, 3H, MeO); **^{13}C NMR** (75 MHz, $CDCl_3$) δ 170.9 (C, C=O), 158.3 (CH, CH=N), 153.1 (C), 135.4 (C), 130.2 (CH), 128.9 (CH), 128.8 (CH), 125.9 (CH), 124.7 (C), 124.3 (C), 124.0 (C), 123.7 (CH), 115.8 (CH), 91.8 (C), 59.6 (CH_3), 54.1 (CH); **HRMS** (ESI) *m/z*: 373.0579 $[M+H]^+$, $C_{18}H_{14}ClN_2O_5^+$ requires 373.0586.

Methyl (1*R*,3a*R*,8b*R*)-8-chloro-3a-nitro-1-phenyl-3a,8b-dihydro-1*H*-benzofuro[2,3-*c*]pyrrole-1-carboxylate (3ma)

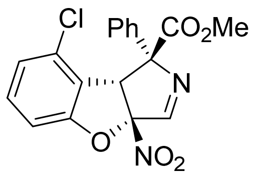

10.4 mg (19%) of **3ma** were obtained from **1m** (29.6 mg, 0.15 mmol) after column chromatography eluting with hexane:EtOAc (95:5). Enantiomeric excess (36%) was measured by HPLC (Lux® Cellulose-3), hexane:*i*PrOH 80:20, 1.0 mL min^{-1}, major enantiomer: t_r = 12.3 min, minor enantiomer: t_r = 20.4 min.

Colorless oil; [α]D^{25} –15.3 (*c* 0.9, $CHCl_3$); **^{1}H NMR** (300 MHz, $CDCl_3$) δ 8.15 (s, 1H, CH=N), 7.14 – 7.06 (m, 3H, Ar), 7.02 (td, *J* = 8.1, 0.6 Hz, 1H, Ar), 6.89 – 6.83 (m, 2H, Ar), 6.78 (dt, *J* = 8.2, 0.7 Hz, 1H, Ar), 6.68 (dd, *J* = 8.1, 0.8 Hz, 1H, Ar), 5.52 (s, 1H, CHAr), 3.98 (s, 3H, MeO); **^{13}C NMR** (75 MHz, $CDCl_3$) δ 170.5 (C, C=O), 158.2 (C), 157.5 (CH, C=N), 134.6 (C), 131.9 (C), 131.5 (CH), 128.8 (CH), 128.1 (CH), 126.1 (CH), 124.1 (CH), 123.5 (C), 122.7 (C), 108.9 (CH), 93.0 (C), 59.2 (CH_3), 54.0 (CH); **HRMS** (ESI) *m/z*: 373.0583 $[M+H]^+$, $C_{18}H_{14}ClN_2O_5^+$ requires 373.0586.

Methyl (1*R*,3a*R*,8b*R*)-5,7-di-*tert*-butyl-3a-nitro-1-phenyl-3a,8b-dihydro-1*H*-benzofuro[2,3-*c*]pyrrole-1-carboxylate (3na)

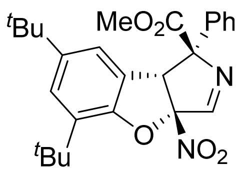

20.5 mg (30%) of **3na** were obtained from **1n** (41.3 mg, 0.15 mmol) after column chromatography eluting with hexane:CH_2Cl_2 (4:6). Enantiomeric excess (82%) was measured by HPLC (CHIRALPAK® AD-H), hexane:*i*PrOH 80:20, 1.0 mL min^{-1}, major enantiomer: t_r = 5.0 min, minor enantiomer: t_r = 8.5 min.

Colorless oil; [α]D^{25} +25.8 (*c* 1.0, $CHCl_3$); **^{1}H NMR** (300 MHz, $CDCl_3$) δ 8.12 (s, 1H), 7.16 – 7.10 (m, 3H), 6.97 (dd, *J* = 2.1, 0.7 Hz, 1H), 6.88 – 6.80 (m, 2H), 6.31 (dd, *J* = 2.0, 0.9 Hz, 1H), 5.25 (t, *J* = 0.8 Hz, 1H), 3.94 (s, 3H), 1.33 (s, 9H), 1.01 (s, 9H); **^{13}C NMR** (75 MHz, $CDCl_3$) δ 171.4 (C), 159.0 (CH, CH=N), 153.0 (C), 145.9 (C), 135.6 (C), 132.9 (C), 128.5 (CH), 125.9 (CH), 124.0 (C), 123.5 (CH), 122.5 (C), 121.0 (CH), 92.1 (C), 59.2 (CH_3), 54.0 (CH_3), 34.5 (C), 34.3 (C), 31.5 (CH_3, MeO), 29.4 (CH); **HRMS** (ESI) *m/z*: 473.2038 $[M+H]^+$, $C_{26}H_{30}ClN_2NaO_5^+$ requires 473.2047.

Methyl (1*R*,3a*R*,8b*R*)-5,7-dichloro-3a-nitro-1-phenyl-3a,8b-dihydro-1H-benzofuro[2,3-*c*]pyrrole-1-carboxylate (3oa)

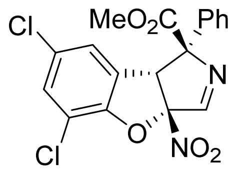

56.6 mg (93%) of **3oa** were obtained from **1o** (34.8 mg, 0.15 mmol) after column chromatography eluting with hexane:EtOAc (9:1). Enantiomeric excess (94%) was measured by HPLC (CHIRALPAK® AD-H), hexane:*i*PrOH 80:20, 1.0 mL min^{-1}, major enantiomer: t_r = 7.5 min, minor enantiomer: t_r = 39.5 min.

Colorless oil; $[\alpha]D^{25}$ +46.6 (*c* 1.0, $CHCl_3$); ^{1}**H NMR** (300 MHz, $CDCl_3$) δ 8.13 (s, 1H, CH=N), 7.29 – 7.24 (m, 3H, Ar), 7.08 (dd, *J* = 2.1, 0.8 Hz, 1H, Ar), 6.96 – 6.89 (m, 2H, Ar), 6.24 (dd, *J* = 2.1, 1.1 Hz, 1H, Ar), 5.38 (t, *J* = 0.9 Hz, 1H), 3.91 (s, 3H); ^{13}**C NMR** (75 MHz, $CDCl_3$) δ 170.6 (C), 158.1 (CH, C=N), 151.9 (C), 135.0 (C), 129.9 (CH), 129.3 (CH), 129.0 (CH), 128.6 (C), 125.7 (CH), 125.5 (C), 124.9 (CH), 123.8 (C), 116.3 (C), 91.9 (C), 59.5 (CH_3), 54.2 (CH); **HRMS** (ESI) *m/z*: 407.0189 $[M+H]^+$, $C_{18}H_{13}Cl_2N_2O_5^+$ requires 407.0196.

Methyl (1*R*,3a*R*,8b*R*)-1-(4-methoxyphenyl)-3a-nitro-3a,8b-dihydro-1*H*-benzofuro[2,3-*c*]pyrrole-1-carboxylate (3ab)

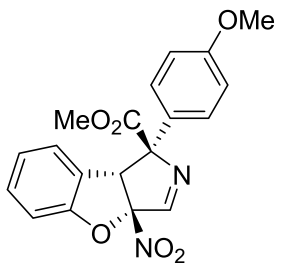

34.3 mg (92%) of **3ab** were obtained from **1a** (24.5 mg, 0.15 mmol) and methyl 2-(*p*-methoxyphenyl)isocyanoacetate (**2b**, 39 µL, 0.19 mmol) after column chromatography eluting with hexane:EtOAc (9:1). Enantiomeric excess (97%) was measured by HPLC (CHIRALPAK® AD-H), hexane:*i*PrOH 80:20, 1.0 mL min^{-1}, major enantiomer: t_r = 14.4 min, minor enantiomer: t_r = 41.4 min.

White foam; $[\alpha]D^{25}$ +87.1 (*c* 1.1, $CHCl_3$); ^{1}**H NMR** (300 MHz, $CDCl_3$) δ 8.05 (s, 1H, CH=N), 7.08 (dddd, *J* = 8.2, 7.4, 1.4, 0.8 Hz, 1H, Ar), 6.92 – 6.80 (m, 3H, Ar), 6.75 – 6.62 (m, 3H, Ar), 6.48 – 6.39 (m, 1H, Ar), 5.32 (s, 1H, CHAr), 3.90 (s, 3H, MeO), 3.72 (s, 3H, MeO); ^{13}**C NMR** (75 MHz, $CDCl_3$) δ 171.3 (C, C=O), 159.7 (C), 158.3 (CH, CH=N), 157.1 (C), 129.8, 127.8 (C), 127.2 (CH), 126.6 (CH), 124.2 (C), 123.2 (CH), 122.7 (C), 113.9 (CH), 110.3 (CH), 91.3 (C), 59.3 (CH_3), 55.4 (CH_3), 54.0 (CH); **HRMS** (ESI) *m/z*: 369.1066 $[M+H]^+$, $C_{19}H_{17}N_2O_6^+$ requires 369.1081.

Methyl (1*R*,3a*R*,8b*R*)-1-(4-methoxyphenyl)-3a,7-dinitro-3a,8b-dihydro-1*H*-benzofuro[2,3-c]pyrrole-1-carboxylate (3eb)

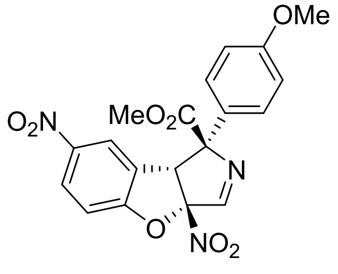

62.6 mg (99%) of **3eb** were obtained from **1e** (31.2 mg, 0.15 mmol) and methyl 2-(*p*-methoxyphenyl)isocyanoacetate (**2b**, 39 µL, 0.19 mmol) after column chromatography eluting with hexane:EtOAc (8:2). Enantiomeric excess (94%) was measured by HPLC (CHIRALPAK® AD-H), hexane:*i*PrOH 80:20, 1.0 mL min^{-1}, major enantiomer: t_r = 33.6 min, minor enantiomer: t_r = 38.5 min.

White foam; [α]D^{25} –59.4 (*c* 1.0, $CHCl_3$); **^{1}H NMR** (300 MHz, $CDCl_3$) δ 8.10 (s, 1H, CH=N), 8.04 (ddd, *J* = 8.9, 2.4, 0.7 Hz, 1H, Ar), 7.37 (dd, *J* = 2.4, 1.1 Hz, 1H, Ar), 6.98 (d, *J* = 9.0 Hz, 1H, Ar), 6.84 (d, *J* = 8.9 Hz, 2H, Ar), 6.73 (d, *J* = 9.0 Hz, 2H, Ar), 5.38 (s, 1H, CHAr), 3.92 (s, 3H, MeO), 3.70 (s, 3H, MeO); **^{13}C NMR** (75 MHz, $CDCl_3$) δ 170.7 (C, C=O), 161.2 (C), 160.1 (C), 157.5 (CH, CH=N), 143.8 (C), 127.0 (C), 126.9 (CH), 126.7 (CH), 124.6 (C), 124.5 (C), 122.9 (CH), 114.4 (CH), 110.6 (CH), 91.8 (C), 58.3 (CH_3), 55.5 (CH_3), 54.3 (CH); **HRMS** (ESI) *m/z*: 414.0915 $[M+H]^+$, $C_{19}H_{16}N_3O_8^+$ requires 414.0932.

Methyl (1*R*,3a*R*,8b*R*)-6-chloro-1-(4-methoxyphenyl)-3a-nitro-3a,8b-dihydro-1*H*-benzofuro[2,3-*c*]pyrrole-1-carboxylate (3ib)

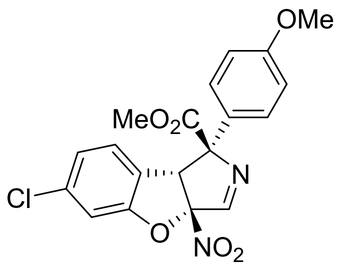

50.8 mg (84%) of **3ib** were obtained from **1i** (29.6 mg, 0.15 mmol) and methyl 2-(*p*-methoxyphenyl)isocyanoacetate (**2b**, 39 μL, 0.19 mmol) after column chromatography eluting with hexane:EtOAc (9:1). Enantiomeric excess (96%) was measured by HPLC (CHIRALPAK® AD-H), hexane:*i*PrOH 80:20, 1.0 mL min^{-1}, major enantiomer: t_r = 12.8 min, minor enantiomer: t_r = 26.2 min.

White foam; [α]D^{25} +88.9 (*c* 1.0, $CHCl_3$); **^{1}H NMR** (300 MHz, $CDCl_3$) δ 8.04 (s, 1H, CH=N), 6.92 – 6.81 (m, 3H, Ar), 6.74 (d, *J* = 9.0 Hz, 2H, Ar), 6.66 (dd, *J* = 8.2, 1.9 Hz, 1H, Ar), 6.33 (dd, *J* = 8.0, 1.0 Hz, 1H, Ar), 5.28 (s, 1H, CHAr), 3.89 (s, 3H, MeO), 3.74 (s, 3H, MeO); **^{13}C NMR** (75 MHz, $CDCl_3$) δ 171.1 (C, C=O), 159.8 (C), 158.0 (CH, CH=N), 157.5 (C), 135.4 (C), 127.5 (C), 127.2 (CH), 127.1 (CH), 124.5 (C), 123.5 (CH), 121.5 (C), 114.1 (CH), 111.2 (CH), 91.3 (C), 58.7 (CH_3), 55.4 (CH_3), 54.0 (CH); **HRMS** (ESI) *m/z*: 403.0678 $[M+H]^+$, $C_{19}H_{16}ClN_2O_6^+$ requires 406.0691.

Methyl (1*R*,3a*R*,8b*R*)-1-(4-chlorophenyl)-3a-nitro-3a,8b-dihydro-1*H*-benzofuro[2,3-*c*]pyrrole-1-carboxylate (3ac)

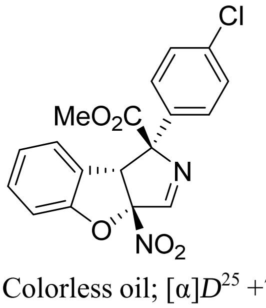

34.8 mg (92%) of **3ac** were obtained from **1a** (24.5 mg, 0.15 mmol) and methyl 2-(*p*-chlorophenyl)isocyanoacetate (**2c**, 22 μL, 0.19 mmol) after column chromatography eluting with hexane:EtOAc (9:1). Enantiomeric excess (78%) was measured by HPLC (CHIRALPAK® AD-H), hexane:*i*PrOH 80:20, 1.0 mL min^{-1}, major enantiomer: t_r = 12.1 min, minor enantiomer: t_r = 32.4 min.

Colorless oil; [α]D^{25} +71.5 (*c* 1.2, $CHCl_3$); **^{1}H NMR** (300 MHz, $CDCl_3$) δ 8.09 (s, 1H, CH=N), 7.19 (d, *J* = 8.8 Hz, 2H, Ar), 7.11 (dddd, *J* = 8.2, 7.4, 1.4, 0.7 Hz, 1H, Ar), 6.94 – 6.86 (m, 3H, Ar), 6.70 (td, *J* = 7.6, 1.0 Hz, 1H, Ar), 6.46 – 6.40 (m, 1H, Ar), 5.34 (s, 1H, CHAr), 3.91 (s, 3H, MeO); **^{13}C NMR** (75 MHz, $CDCl_3$) δ 170.8 (C, C=O), 159.1 (CH, CH=N), 157.1 (C), 134.8 (C), 134.3 (C), 130.1 (CH), 128.8 (CH), 127.5 (CH), 126.4 (CH), 124.0 (C), 123.4 (CH), 122.2 (C), 110.5 (CH), 91.2 (C), 59.1 (CH_3), 54.2 (CH); **HRMS** (ESI) *m/z*: 373.0573 $[M+H]^+$, $C_{18}H_{14}ClN_2O_5^+$ requires 373.0586.

Methyl (1*R*,3a*R*,8b*R*)-3a-nitro-1-(4-nitrophenyl)-3a,8b-dihydro-1H-benzofuro[2,3-*c*]pyrrole-1-carboxylate (3ad)

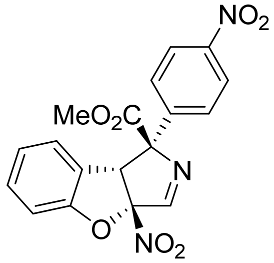

55.3 mg (96%) of **3ad** were obtained from **1a** (24.5 mg, 0.15 mmol) and methyl 2-(*p*-nitrophenyl)isocyanoacetate (**2d**, 41.8 mg, 0.19 mmol) after column chromatography eluting with hexane:Et_2O (1:1). Enantiomeric excess (67%) was measured by HPLC (CHIRALPAK® AS-H), hexane:*i*PrOH 80:20, 1.0 mL min^{-1}, major enantiomer: t_r = 52.6 min, minor enantiomer: t_r = 31.9 min.

Orange oil; $[\alpha]D^{25}$ +63.1 (*c* 0.7, $CHCl_3$); **^{1}H NMR** (300 MHz, $CDCl_3$) δ 8.16 (s, 1H, CH=N), 8.07 (d, *J* = 8.9 Hz, 2H, Ar), 7.19 (d, *J* = 8.9 Hz, 2H, Ar), 7.16 – 7.04 (m, 1H, Ar), 6.89 (d, *J* = 8.2 Hz, 1H, Ar), 6.66 (td, *J* = 7.6, 1.0 Hz, 1H, Ar), 6.43 (d, *J* = 7.7 Hz, 1H, Ar), 5.39 (s, 1H, CHAr), 3.93 (s, 3H; MeO); **^{13}C NMR** (75 MHz, $CDCl_3$) δ 170.2 (C, C=O), 159.9 (CH, CH=N), 157.2 (C), 147.9 (C), 142.6 (C), 130.5 (CH), 127.4 (CH), 126.2 (CH), 123.9 (C), 123.7 (CH), 123.5 (CH), 121.6 (C), 110.8 (CH), 91.2 (C), 59.2 (CH_3), 54.5 (CH); **HRMS** (ESI) *m/z*: 384.0822 $[M+H]^+$, $C_{18}H_{14}N_3O_7^+$ requires 384.0826.

Methyl (1*S*,3a*R*,8b*R*)-1-methyl-3a-nitro-3a,8b-dihydro-1*H*-benzofuro[2,3-*c*]pyrrole-1-carboxylate (3ae)

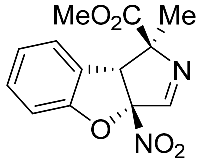

18.8 mg (45%) of **3ae** were obtained from **1a** (24.5 mg, 0.15 mmol) and 2-methylisocyanoacetate (**2e**, 17 µL, 0.19 mmol) after column chromatography eluting with hexane:EtOAc (9:1). Enantiomeric excess (0%) was measured by HPLC (CHIRALPAK® AD-H), hexane:*i*PrOH 80:20, 1.0 mL min^{-1}, major enantiomer: t_r = 11.0 min, minor enantiomer: t_r = 12.5 min.

Orange oil; **^{1}H NMR** (300 MHz, $CDCl_3$) δ 7.83 (s, 1H, CH=N), 7.31 (t, *J* = 7.5 Hz, 1H, Ar), 7.15 (d, *J* = 7.9 Hz, 1H, Ar), 7.03 (t, *J* = 7.0 Hz, 2H, Ar), 4.18 (s, 1H, CHN), 3.21 (s, 3H, MeO), 1.87 (s, 3H, Me). **^{13}C NMR** (75 MHz, $CDCl_3$) δ 169.7 (C, C=O), 157.5 (C), 156.9 (CH, C=N), 130.8 (CH), 125.4 (CH), 125.0 (C), 123.4 (CH), 122.9 (CH), 110.9 (CH), 86.6 (C), 61.5 (CH_3), 52.4 (CH), 25.3 (CH_3); **HRMS** (ESI) *m/z*: 277.0819 $[M+H]^+$, $C_{13}H_{13}N_2O_5^+$ requires 277.0819.

Methyl (1*R*,3a*R*,8b*R*)-3a-nitro-1-phenyl-3a,8b-dihydro-1*H*-benzo[4,5]thieno[2,3-*c*]pyrrole-1-carboxylate (4)

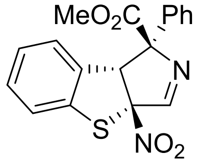

4.4 mg (8%) of **4** were obtained from **1n** (26.9 mg, 0.15 mmol) after column chromatography eluting with hexane:EtOAc (9:1). Enantiomeric excess (72%) was measured by HPLC (CHIRALPAK® AD-H), hexane:*i*PrOH 80:20, 1.0 mL min^{-1}, major enantiomer: t_r = 10.2 min, minor enantiomer: t_r = 39.1 min.

Orange oil; $[\alpha]D^{25}$ +36.7 (*c* 0.4, $CHCl_3$); **^{1}H NMR** (300 MHz, $CDCl_3$) δ 8.24 (s, 1H, CH=N), 7.20 (ddt, *J* = 7.5, 1.5, 0.7 Hz, 1H, Ar), 7.10 – 7.02 (m, 3H, Ar), 7.00 (dd, *J* = 8.0, 1.5 Hz, 1H, Ar), 6.94 (dd, *J* = 7.5, 1.3 Hz, 1H, Ar), 6.93 – 6.80 (m, 3H, Ar), 5.65 (s, 1H, CHAr), 3.92 (s, 3H, MeO); **^{13}C NMR** (75 MHz, $CDCl_3$) δ 171.8 (C, C=O), 159.7 (CH, CH=N), 137.2 (C), 134.6 (C), 133.7 (CH), 129.4 (CH), 128.5 (CH), 128.0 (CH), 127.7 (CH), 126.1 (CH), 125.6 (CH), 121.2 (CH), 112.0 (CH), 92.6 (C), 64.6 (CH_3), 53.9 (CH); **HRMS** (ESI) *m/z*: 355.0752 $[M+H]^+$, $C_{18}H_{15}N_2O_4^+S$ requires 355.0747.

Synthesis of 3aa at 1 mmol scale

To a solution of **1a** (163.1 mg, 1.0 mmol) and **III** (45.1 mg, 0.1 mol) in dry $CHCl_3$ (5 mL) was added 2-phenylisocyanoacetate (**2a**, 190 µL, 1.3 mmol). The reaction was stirred for 6 days at 0 °C and passed through a short-path column. The resulting mixture was used for the determination of the diastereomeric ratio by ^{1}H-NMR analysis. The product was then purified by column chromatography (eluent: hexane:EtOAc 9:1) to furnish 254 mg (75%) of **3aa** as a yellowish foam. Enantiomeric excess (91%) was measured by HPLC (CHIRALPAK® AD-H), hexane:*i*PrOH 80:20, 1.0 mL min^{-1}, major enantiomer: t_r = 8.5 min, minor enantiomer: t_r = 24.1 min.

Transformations of product 3aa

Methyl (*S*)-1-phenyl-1*H*-benzofuro[2,3-*c*]pyrrole-1-carboxylate (6)

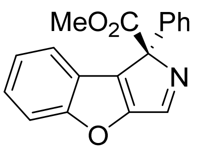

A modification of a literature procedure was employed.[14] To a solution of **3aa** (31.9 mg, 0.094 mmol, 1.0 equiv, *ee* 93%) in dry dichloromethane (2 mL, 0.05 M) was added DBU (70 µL, 0.470 mmol, 5 equiv). The mixture was stirred at room temperature for 17 h. Purification by column chromatography (hexane:EtOAc 8:2) furnished 14.4 mg (53%) of the title compound. Enantiomeric excess (90%) was measured by HPLC (CHIRALPAK® AD-H), hexane:*i*PrOH 80:20, 1.0 mL min^{-1}, major enantiomer: t_r = 11.9 min, minor enantiomer: t_r = 8.2 min.

Red oil; $[\alpha]D^{25}$ +11.0 (*c* 1.0, $CHCl_3$); **^{1}H NMR** (300 MHz, $CDCl_3$) δ 8.52 (s, 1H, CH=N), 7.94 – 7.87 (m, 1H, Ar), 7.73 – 7.67 (m, 2H, Ar), 7.65 – 7.59 (m, 1H, Ar), 7.49 – 7.41 (m, 2H, Ar), 7.40 – 7.32 (m, 3H, Ar), 3.76 (s, 3H, MeO); **^{13}C NMR** (75 MHz, $CDCl_3$) δ 168.9 (C, C=O), 161.6 (C), 160.8 (C), 153.2 (CH, CH=N), 140.5 (C), 135.8 (C), 128.9 (CH), 128.8 (CH), 127.9 (CH), 126.5 (CH), 124.6 (C), 124.6 (CH), 121.4 (CH), 113.7 (CH), 82.8 (C), 53.6 (CH_3); **HRMS** (ESI) *m/z*: 292.0968 $[M+H]^+$, $C_{18}H_{14}NO_3^+$ requires 292.0968.

Methyl (1*R*,3a*R*,8b*R*)-3a-nitro-1-phenyl-2,3,3a,8b-tetrahydro-1*H*-benzofuro[2,3-*c*]pyrrole-1-carboxylate (7)

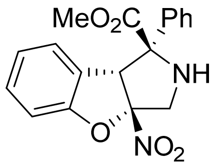

A modification of a literature procedure was employed.[15] To a solution of **3aa** (21.1 mg, 0.062 mmol, 1.0 equiv, *ee* 89%) in dry dichloromethane (1.8 mL, 0.034 M) and triethylsilane (30 µL, 0.187 mmol, 3.0 equiv), under nitrogen atmosphere, was added $BF_3 \cdot Et_2O$ (25 µL, 0.205 mmol, 3.3 equiv). The mixture was stirred at room temperature until complete consumption of the starting material (TLC, *ca.* 17 h). The mixture was quenched with saturated aqueous $NaHCO_3$ (2 mL). CH_2Cl_2 (10 mL) were added and the mixture was transferred to a separatory funnel. The phases were separated and the aqueous phase was extracted with CH_2Cl_2 (3×5 mL). The combined organic phases were washed with brine (5 mL), dried over Na_2SO_4, filtered and concentrated under reduced pressure. Purification by column chromatography (hexane:EtOAc 8:2) furnished 20.4 mg (97%) of the title compound. Enantiomeric excess (89%) was measured by HPLC (CHIRALPAK® AD-H), hexane:*i*PrOH 80:20, 1.0 mL min^{-1}, major enantiomer: t_r = 7.2 min, minor enantiomer: t_r = 17.2 min.

White solid; $[\alpha]D^{25}$ +180.5 (*c* 1.0, $CHCl_3$); **^{1}H NMR** (300 MHz, $CDCl_3$) δ 7.37 – 7.28 (m, 2H, Ar), 7.26 (tdd, *J* = 4.5, 3.1, 1.2 Hz, 3H, Ar), 7.10 (dddd, *J* = 8.2, 7.5, 1.4, 0.7 Hz, 1H, Ar), 6.93 (d, *J* = 7.7 Hz, 1H, Ar), 6.55 (td, *J* = 7.5, 1.1 Hz, 1H, Ar), 6.04 (d, *J* = 7.7 Hz, 1H, Ar), 5.19 (s, 1H, CHAr), 3.93 (s, 3H, MeO), 3.84 (d, 1H, *J* = 16.2 Hz), 3.83 (d, *J* = 15.7 Hz, 1H), 2.87 (s, 1H, NH); **^{13}C NMR** (75 MHz, $CDCl_3$) δ 174.0 (C, C=O), 159.2 (C), 135.8 (C), 129.4 (CH), 128.8 (CH), 128.5 (CH), 126.4 (CH), 126.3 (CH), 122.9 (C), 122.2 (CH), 121.6 (C), 109.6 (CH), 77.3 (C), 60.1 (CH_3), 54.9 (CH_2), 53.7 (CH); **HRMS** (ESI) *m/z*: 341.1128 $[M+H]^+$, $C_{18}H_{17}N_2O_5^+$ requires 341.1132.

Methyl (1*R*,3*S*,3a*R*,8b*R*)-3-methoxy-3a-nitro-1-phenyl-2,3,3a,8b-tetrahydro-1*H*-benzofuro[2,3-*c*]pyrrole-1-carboxylate (8)

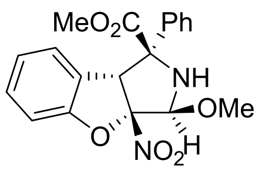

A solution of **3aa** (25.2 mg, 0.075 mmol) dissolved in MeOH (2 mL, 0.04 M) was stirred at reflux for 17 h (oil bath at 70 °C). The solvent was then evaporated to obtain 27.6 mg of **7** (99%). Enantiomeric excess (98%) was measured by HPLC (CHIRALPAK® AD-H), hexane:*i*PrOH 80:20, 1.0 mL min^{-1}, major enantiomer: t_r = 8.8 min, minor enantiomer: t_r = 16.8 min. White foam; $[\alpha]D^{25}$ +208.4 (*c* 1.1, $CHCl_3$); **^{1}H NMR** (300 MHz, $CDCl_3$) δ 7.24 – 7.15 (m, 5H, Ar), 7.03 (t, *J* = 7.7 Hz, 1H, Ar), 6.85 (d, *J* = 8.1 Hz, 1H, Ar), 6.51 (td, *J* = 7.5, 1.0 Hz, 1H, Ar), 6.13 (d, *J* = 7.6 Hz, 1H, Ar), 5.77 (s, 1H, CHNH), 5.03 (s, 1H, CHAr), 3.85 (s, 3H, MeO), 3.72 (s, 1H, NH), 3.36 (s, 3H, MeO); **^{13}C NMR** (75 MHz, $CDCl_3$) δ 174.9 (C, C=O), 157.7 (C), 136.7 (C), 129.1 (CH), 128.6 (CH), 128.5 (CH), 126.5 (CH), 125.8 (CH), 123.8 (C), 123.4 (C), 122.3 (CH), 109.5 (CH), 92.2 (CH), 75.0 (C), 55.6 (CH), 53.4 (CH_3), 53.2 (CH_3); **HRMS** (ESI) *m/z*: 393.1048 $[M+Na]^+$, $C_{19}H_{18}N_2NaO_6^+$ requires 393.1057.

Methyl (1*R*,3S,3a*R*,8b*R*)-7-bromo-3-methoxy-3a-nitro-1-phenyl-2,3,3a,8b-tetrahydro-1*H*-benzofuro[2,3-c]pyrrole-1-carboxylate (9)

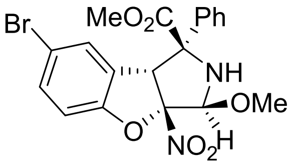

Following the same procedure for the synthesis of **7**, from **3da** (45.1 mg, 0.11 mmol), 48.5 mg of **9** (99%) were obtained. Enantiomeric excess (98%) was measured by HPLC (CHIRALPAK® AD-H), hexane:*i*PrOH 80:20, 1.0 mL min^{-1}, major enantiomer: t_r = 8.5 min, minor enantiomer: t_r = 12.3 min. White solid, m.p. 201.7-203.0; $[\alpha]D^{25}$ +158.1 (*c* 1.0, $CHCl_3$); **^{1}H NMR** (300 MHz, $CDCl_3$) δ 7.30 – 7.21 (m, 3H, Ar), 7.21 – 7.12 (m, 3H, Ar), 6.73 (d, *J* = 8.6 Hz, 1H, Ar), 6.19 (dd, *J* = 2.1, 1.0 Hz, 1H, Ar), 5.73 (s, 1H, CHN), 5.01 (s, 1H, CHAr), 3.86 (s, 3H, MeO), 3.72 (s, 1H, NH), 3.36 (s, 3H, MeO); **^{13}C NMR** (75 MHz, $CDCl_3$) δ 174.5 (C, C=O), 156.7 (C), 136.4 (C), 132.0 (CH), 129.6 (CH), 129.0 (CH), 128.7 (CH), 126.2 (C), 125.6 (CH), 123.6 (C), 114.6 (C), 111.0 (CH), 92.2 (CH), 75.0 (C), 55.7 (CH_3), 53.6 (CH), 53.3 (CH_3); **HRMS** (ESI) *m/z*: 471.0153 $[M+Na]^+$, $C_{19}H_{17}BrN_2NaO_6^+$ requires 471.0162. Compound **9** was re-crystallized from hexane-MeOH to give suitable crystals for X-ray analysis.

References

(1) Yang, X.-H.; Li, J.-P.; Wang, D.-C.; Xie, M.-S.; Qu, G.-R.; Guo, H.-M. *Chem. Commun.* **2019**, *55*, 9144–9147.
(2) Cheng, Q.; Zhang, H.-J.; Yue, W.-J.; You, S.-L. *Chem* **2017**, *3*, 428–436.
(3) Ling, J.; Laugeois, M.; Michelet, V.; Ratovelomanana-Vidal, V.; Vitale, M. R. *Synlett* **2018**, *29*, 928–932.
(4) Ohishi, Y.; Doi, Y.; Nakanishi, T. *Chem. Pharm. Bull.* **1984**, *32*, 4260–4270.
(5) Tromelin, A.; Demerseman, P.; Royer, R. *Synthesis* **1985**, *1985*, 1074–1076.
(6) Osipov, D. V.; Korzhenko, K. S.; Rashchepkina, D. A.; Artemenko, A. A.; Demidov, O. P.; Shiryaeva, V. A.; Osyanin, V. A. *Org. Biomol. Chem.* **2021**, *19*, 10156–10168.
(7) Buyck, T.; Wang, Q.; Zhu, J. *Angew. Chem. Int. Ed.* **2013**, *52*, 12714–12718.
(8) Madej, A.; Paprocki, D.; Koszelewski, D.; Żądło-Dobrowolska, A.; Brzozowska, A.; Walde, P.; Ostaszewski, R. *RSC Adv.* **2017**, *7*, 33344–33354.
(9) Nammalwar, B.; Muddala, N. P.; Watts, F. M.; Bunce, R. A. *Tetrahedron* **2015**, *71*, 9101–9111.
(10) Kitano, Y.; Manoda, T.; Miura, T.; Chiba, K.; Tada, M. *Synthesis* **2006**, *2006*, 405–410.
(11) Elders, N.; Ruijter, E.; de Kanter, F. J. J.; Groen, M. B.; Orru, R. V. A. *Chem. Eur. J.* **2008**, *14*, 4961–4973.
(12) Bonne, D.; Dekhane, M.; Zhu, J. *Angew. Chem. Int. Ed.* **2007**, *46*, 2485–2488.
(13) Liu, Y.; Sun, B.; Wang, B.; Wakem, M.; Deng, L. *J. Am. Chem. Soc.* **2009**, *131*, 418–419.
(14) Zhao, J.-Q.; Zhou, X.-J.; Zhou, Y.; Xu, X.-Y.; Zhang, X.-M.; Yuan, W.-C. *Org. Lett.* **2018**, *20*, 909–912.
(15) Han, B.; Li, J.-L.; Ma, C.; Zhang, S.-J.; Chen, Y.-C. *Angew. Chem. Int. Ed.* **2008**, *47*, 9971–9974.

NMR spectra

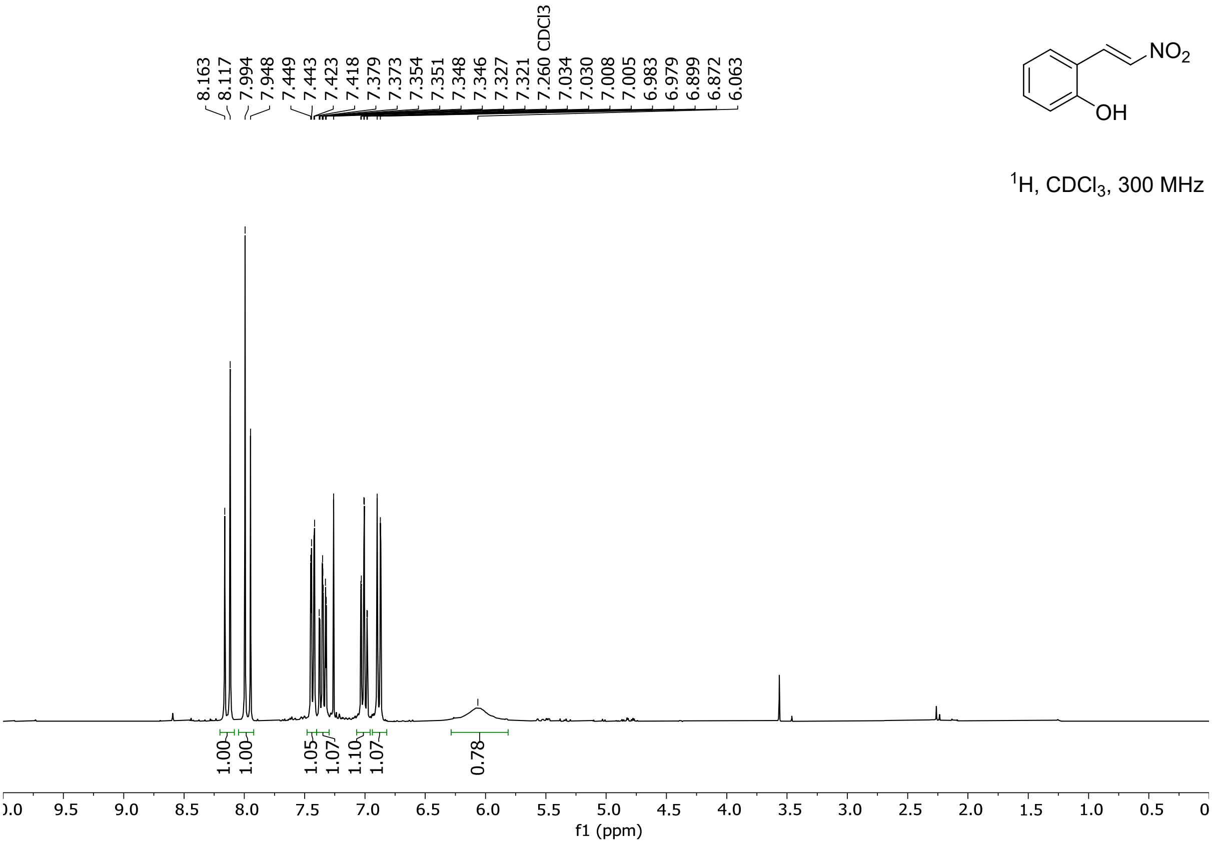

8.163
8.117
7.994
7.948
7.449
7.443
7.423
7.418
7.379
7.373
7.354
7.351
7.348
7.346
7.327
7.321
7.260 CDCl3
7.034
7.030
7.008
7.005
6.983
6.979
6.899
6.872
6.063
NO2
OH
1H, CDCl3, 300 MHz
1.00
1.00
1.05
1.07
1.10
1.07
0.78
f1 (ppm)

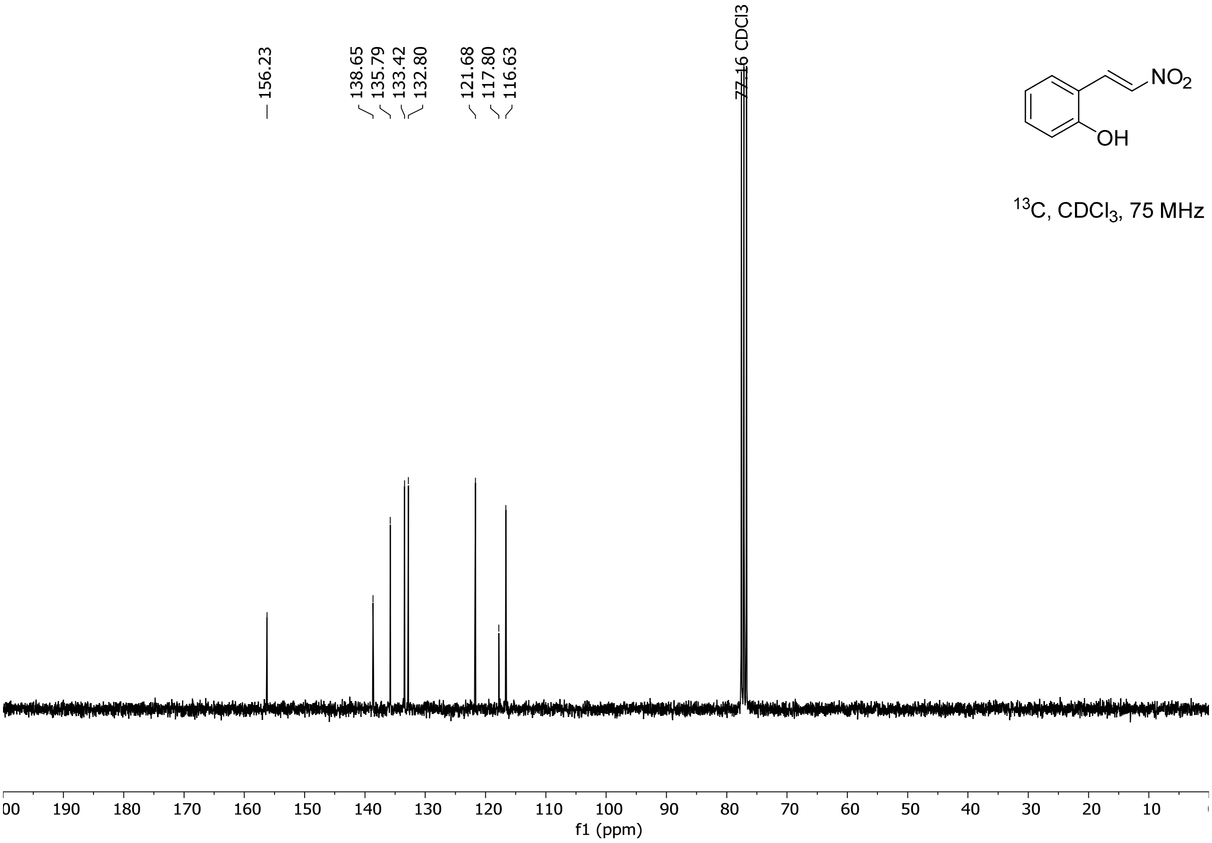

156.23
138.65
135.79
133.42
132.80
121.68
117.80
116.63
77.16 CDCl3
NO2
OH
13C, CDCl3, 75 MHz
f1 (ppm)

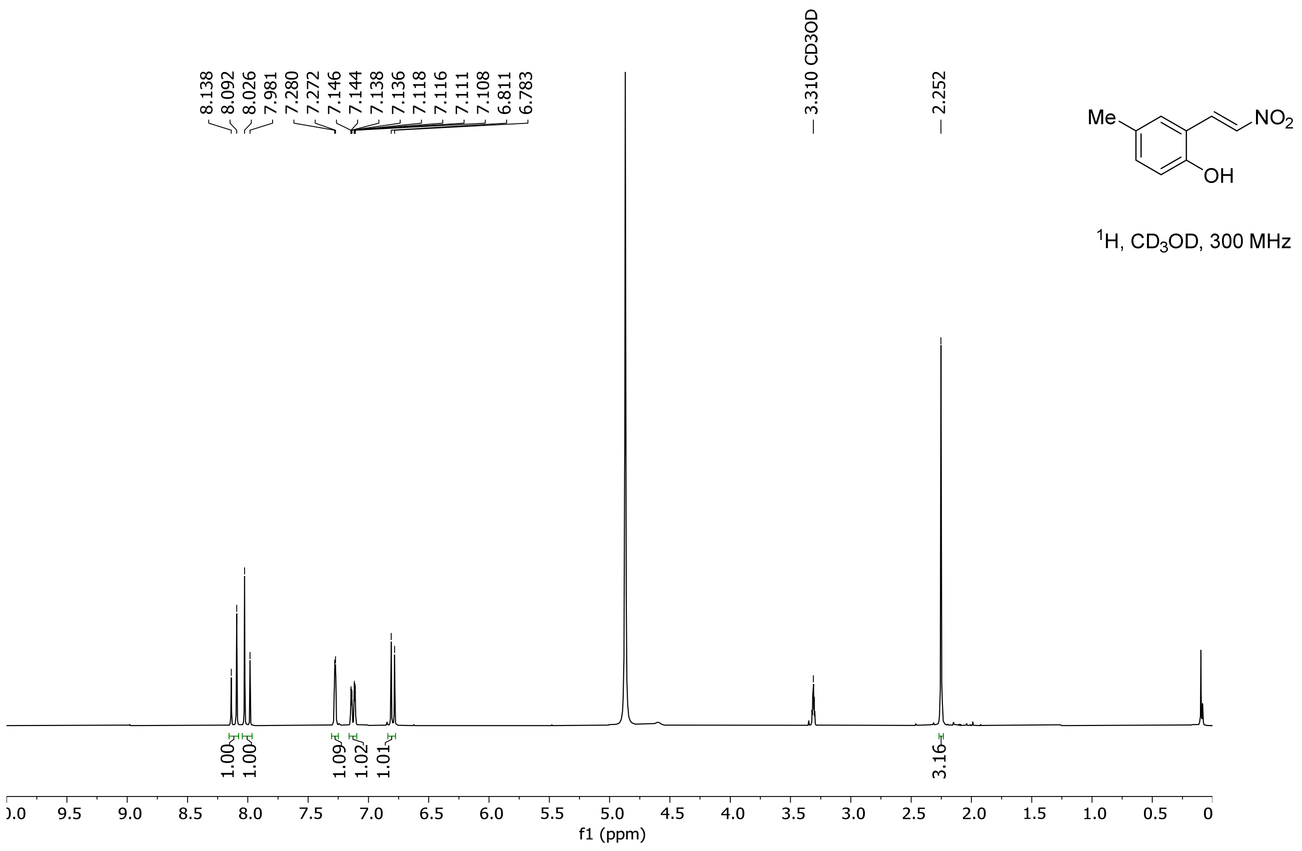
8.138
8.092
8.026
7.981
7.280
7.272
7.146
7.144
7.138
7.136
7.118
7.116
7.111
7.108
6.811
6.783
3.310 CD3OD
2.252
Me
NO2
OH
1H, CD3OD, 300 MHz
1.00
1.00
1.09
1.02
1.01
3.16
f1 (ppm)

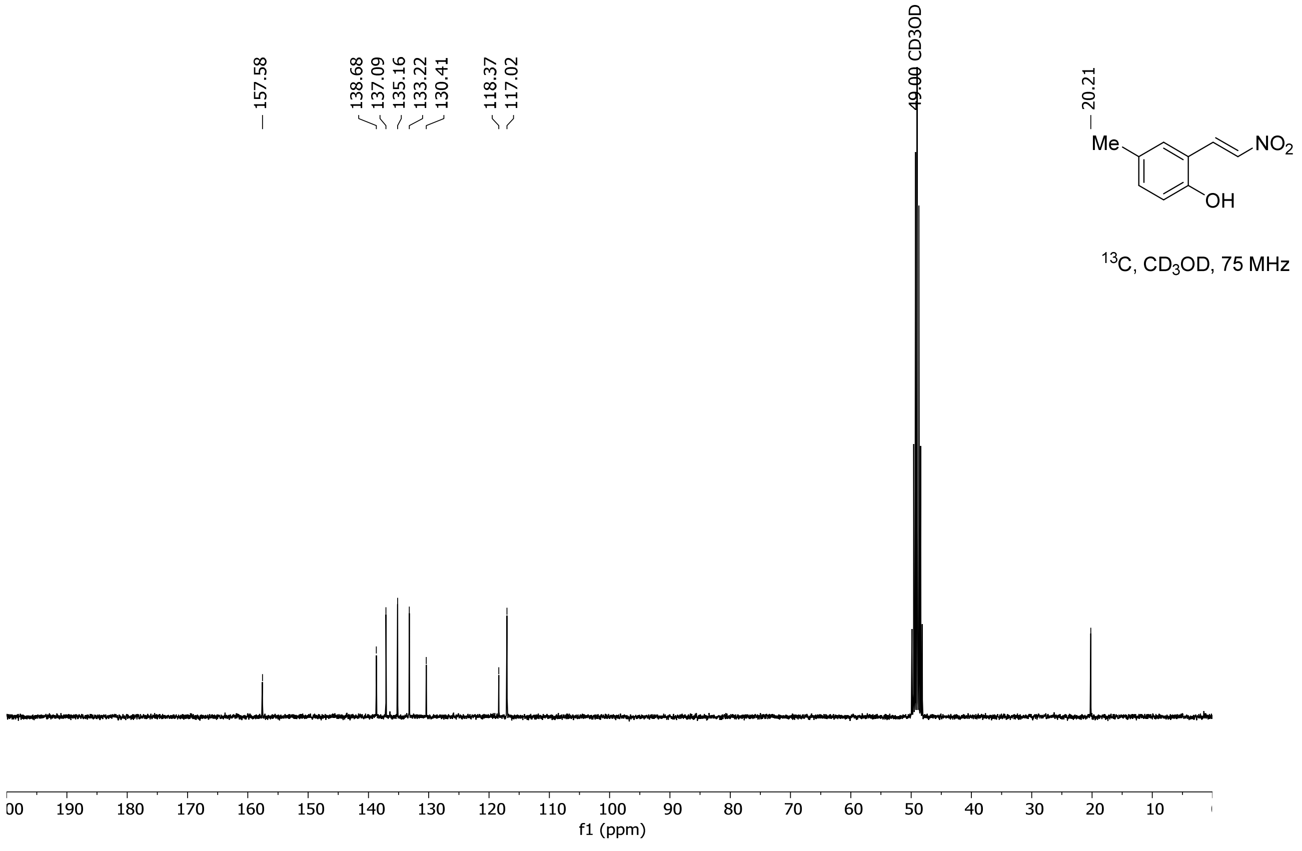
157.58
138.68
137.09
135.16
133.22
130.41
118.37
117.02
49.00 CD3OD
20.21
Me
NO2
OH
13C, CD3OD, 75 MHz
f1 (ppm)

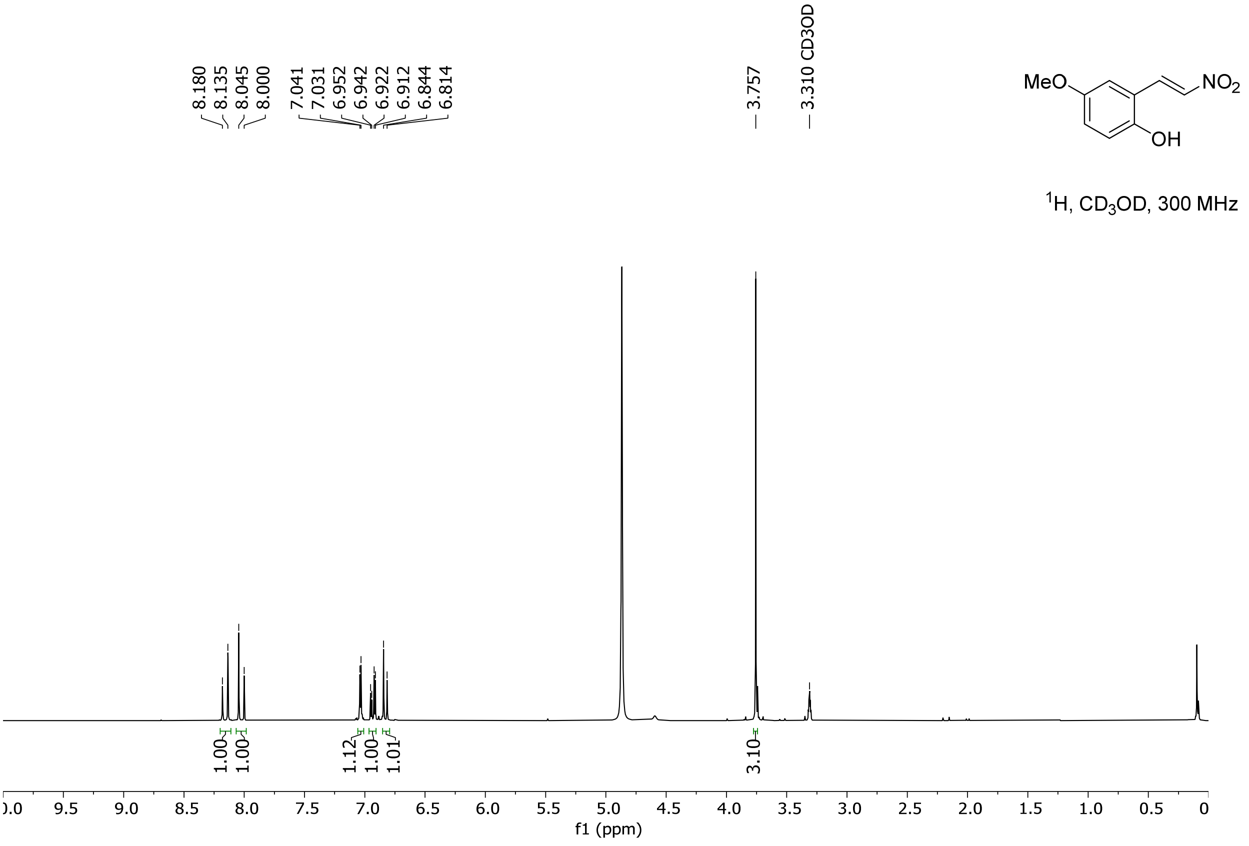

8.180
8.135
8.045
8.000
7.041
7.031
6.952
6.942
6.922
6.912
6.844
6.814
3.757
3.310 CD3OD
MeO
NO2
OH
1H, CD3OD, 300 MHz
1.00
1.00
1.12
1.00
1.01
3.10
f1 (ppm)

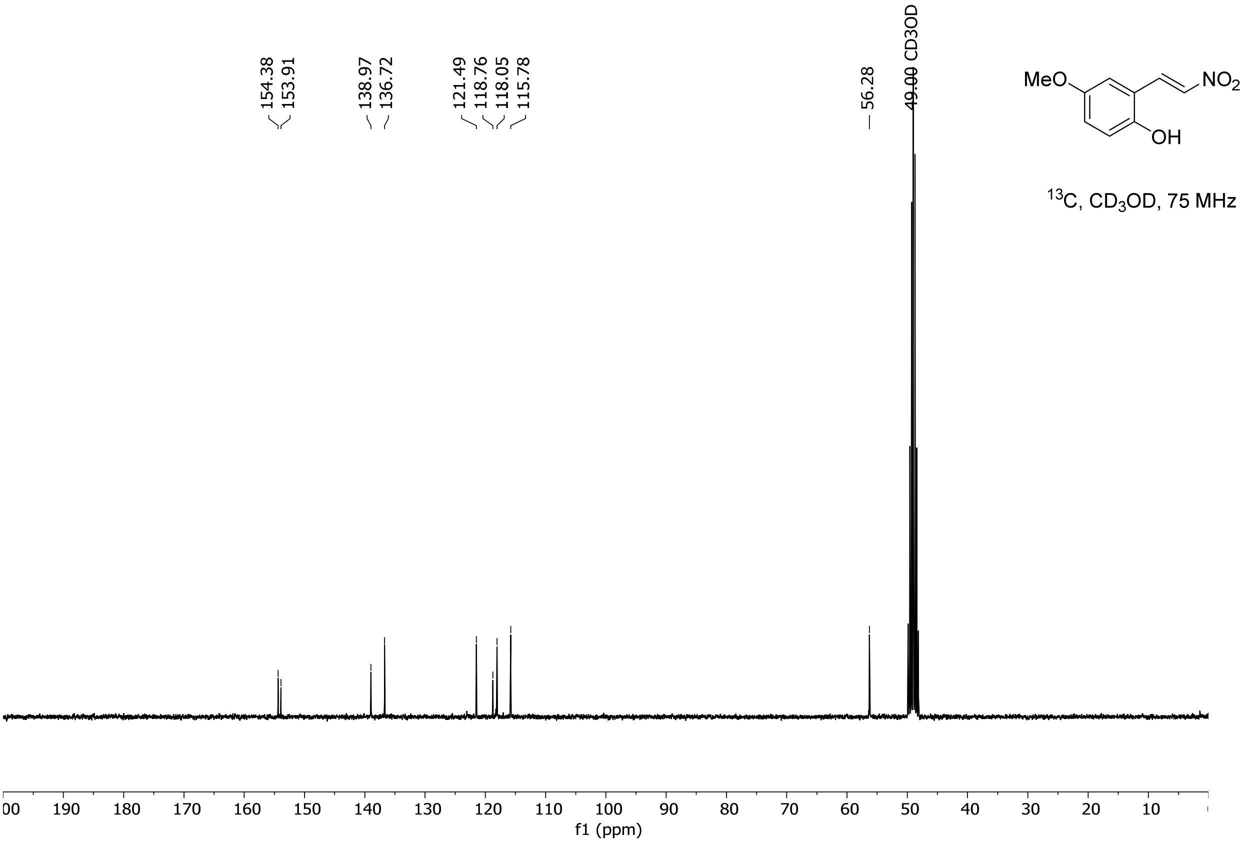

154.38
153.91
138.97
136.72
121.49
118.76
118.05
115.78
56.28
49.00 CD3OD
MeO
NO2
OH
13C, CD3OD, 75 MHz
f1 (ppm)

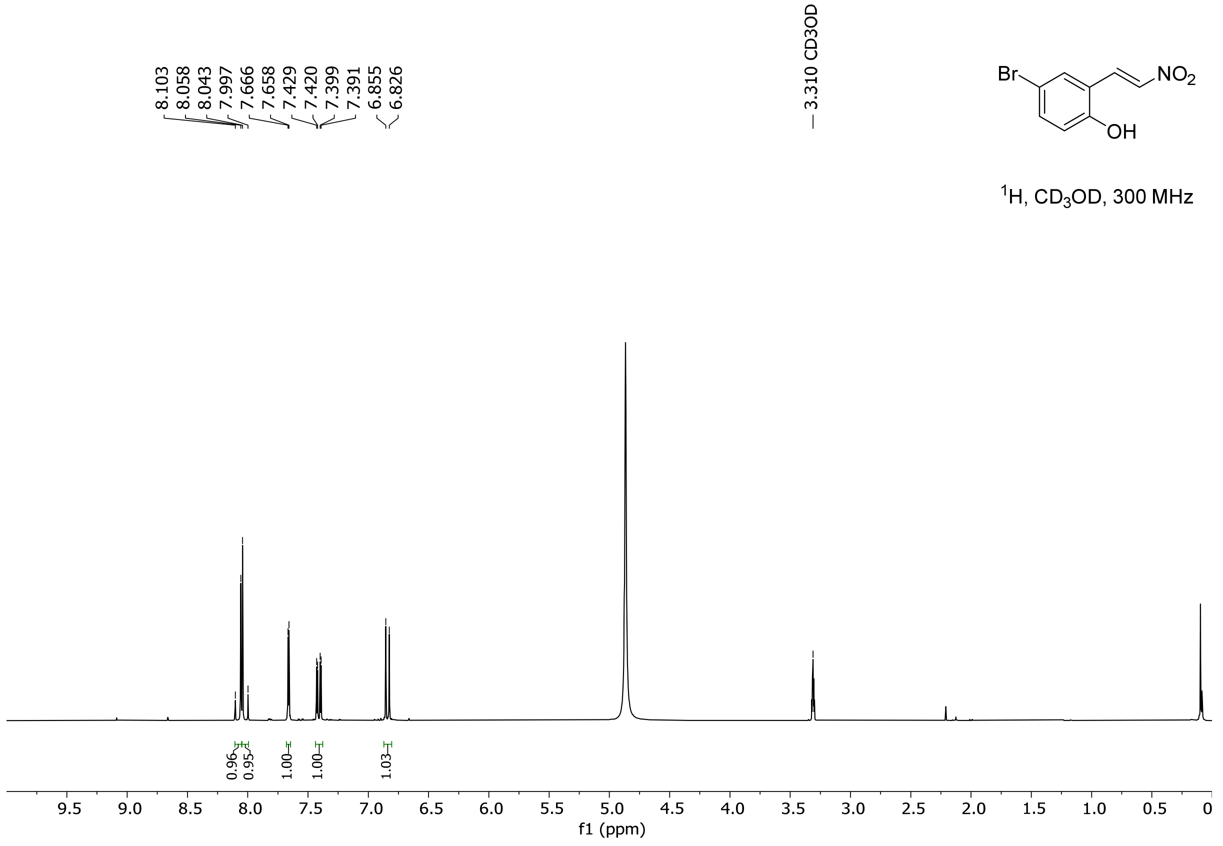
8.103
8.058
8.043
7.997
7.666
7.658
7.429
7.420
7.399
7.391
6.855
6.826
3.310 CD3OD
Br
NO_2
OH
^{1}H, CD_3OD, 300 MHz
0.96
0.95
1.00
1.00
1.03
9.5 9.0 8.5 8.0 7.5 7.0 6.5 6.0 5.5 5.0 4.5 4.0 3.5 3.0 2.5 2.0 1.5 1.0 0.5 0
f1 (ppm)

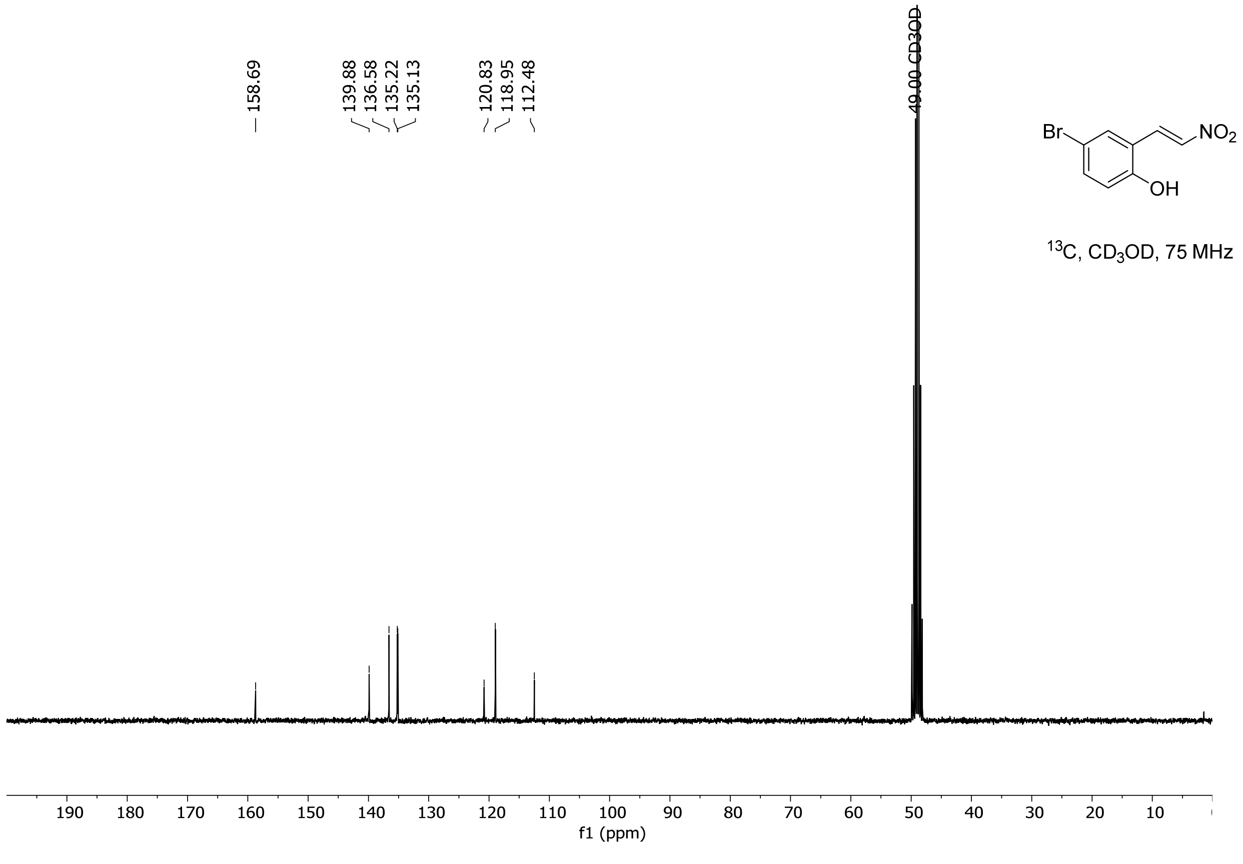
158.69
139.88
136.58
135.22
135.13
120.83
118.95
112.48
49.00 CD3OD
Br
NO_2
OH
^{13}C, CD_3OD, 75 MHz
190 180 170 160 150 140 130 120 110 100 90 80 70 60 50 40 30 20 10
f1 (ppm)

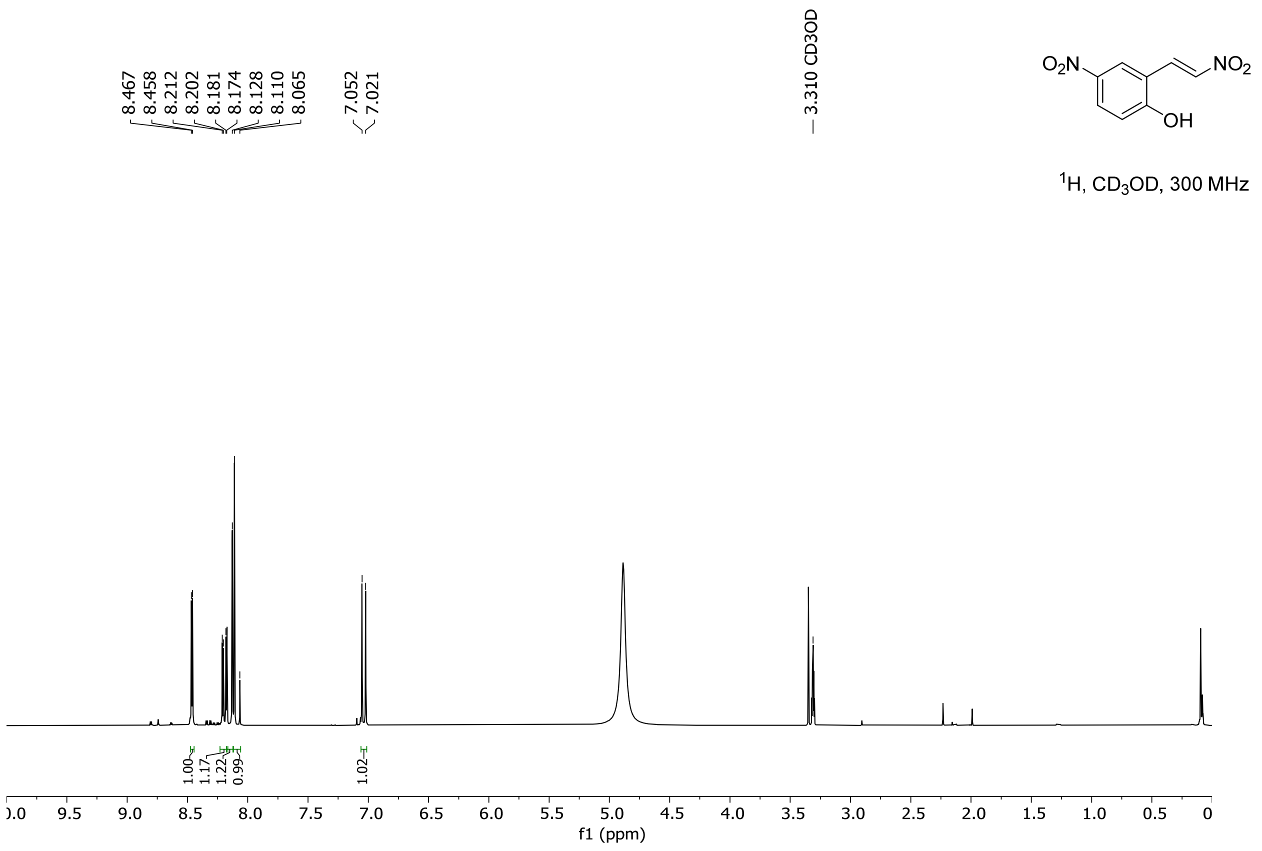

^1H, CD_3OD, 300 MHz

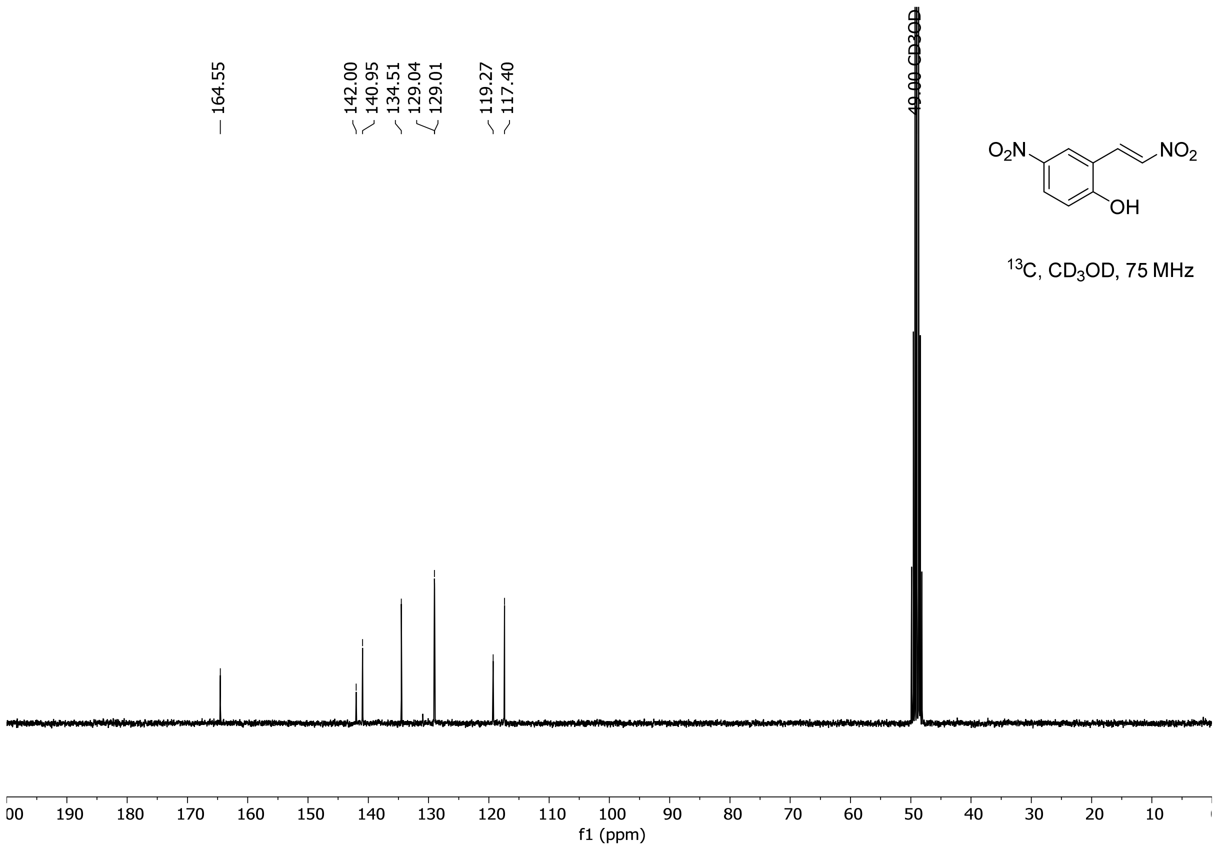

^{13}C, CD_3OD, 75 MHz

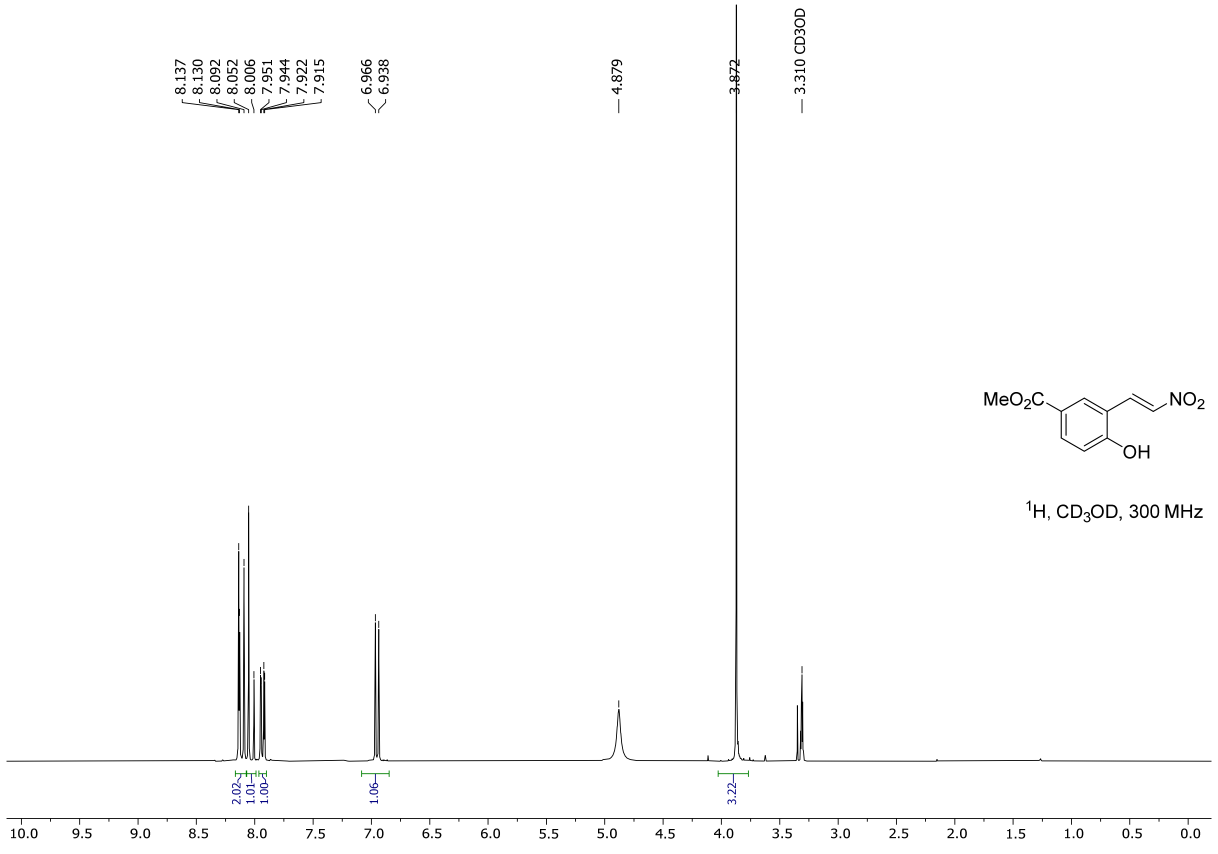
8.137
8.130
8.092
8.052
8.006
7.951
7.944
7.922
7.915
6.966
6.938
4.879
3.872
3.310 CD3OD
MeO2C
NO2
OH
1H, CD3OD, 300 MHz
2.02
1.01
1.00
1.06
3.22
10.0 9.5 9.0 8.5 8.0 7.5 7.0 6.5 6.0 5.5 5.0 4.5 4.0 3.5 3.0 2.5 2.0 1.5 1.0 0.5 0.0

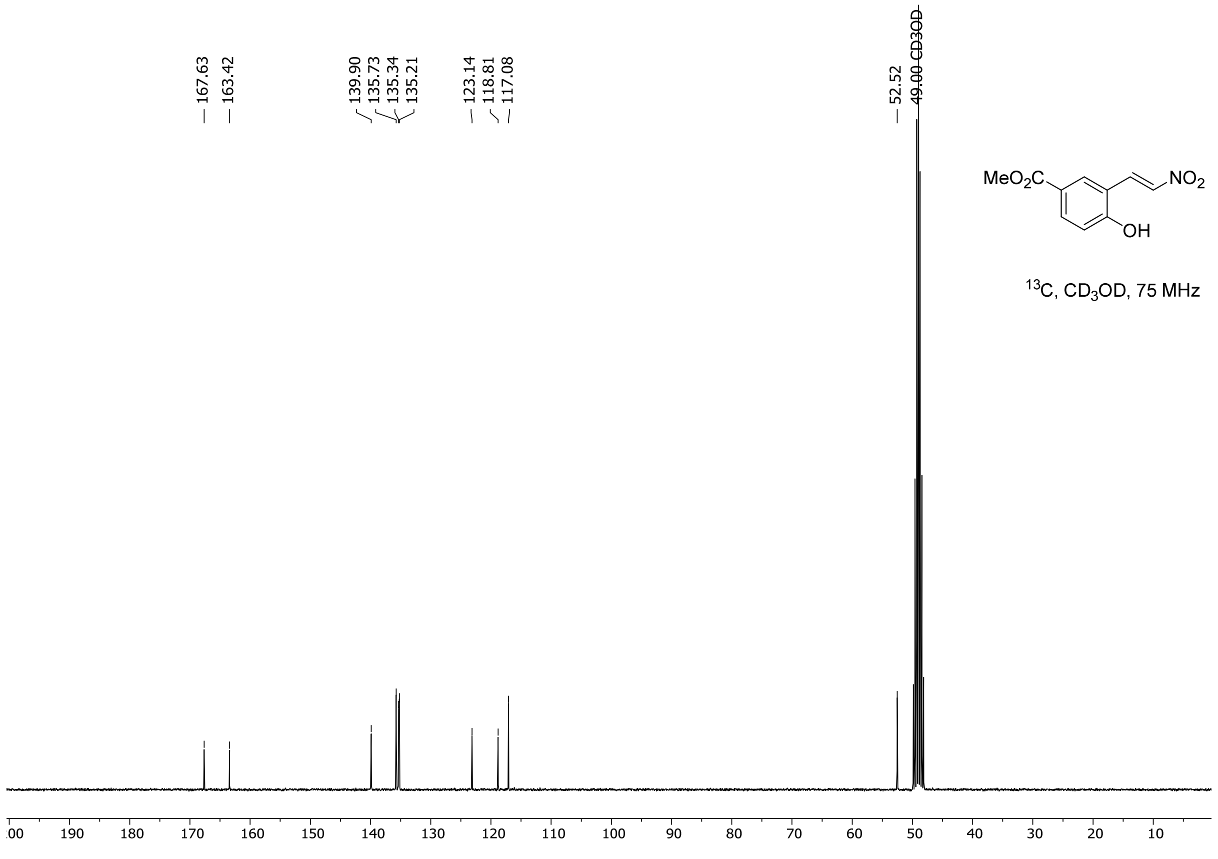
167.63
163.42
139.90
135.73
135.34
135.21
123.14
118.81
117.08
52.52
49.00 CD3OD
MeO2C
NO2
OH
13C, CD3OD, 75 MHz
00 190 180 170 160 150 140 130 120 110 100 90 80 70 60 50 40 30 20 10

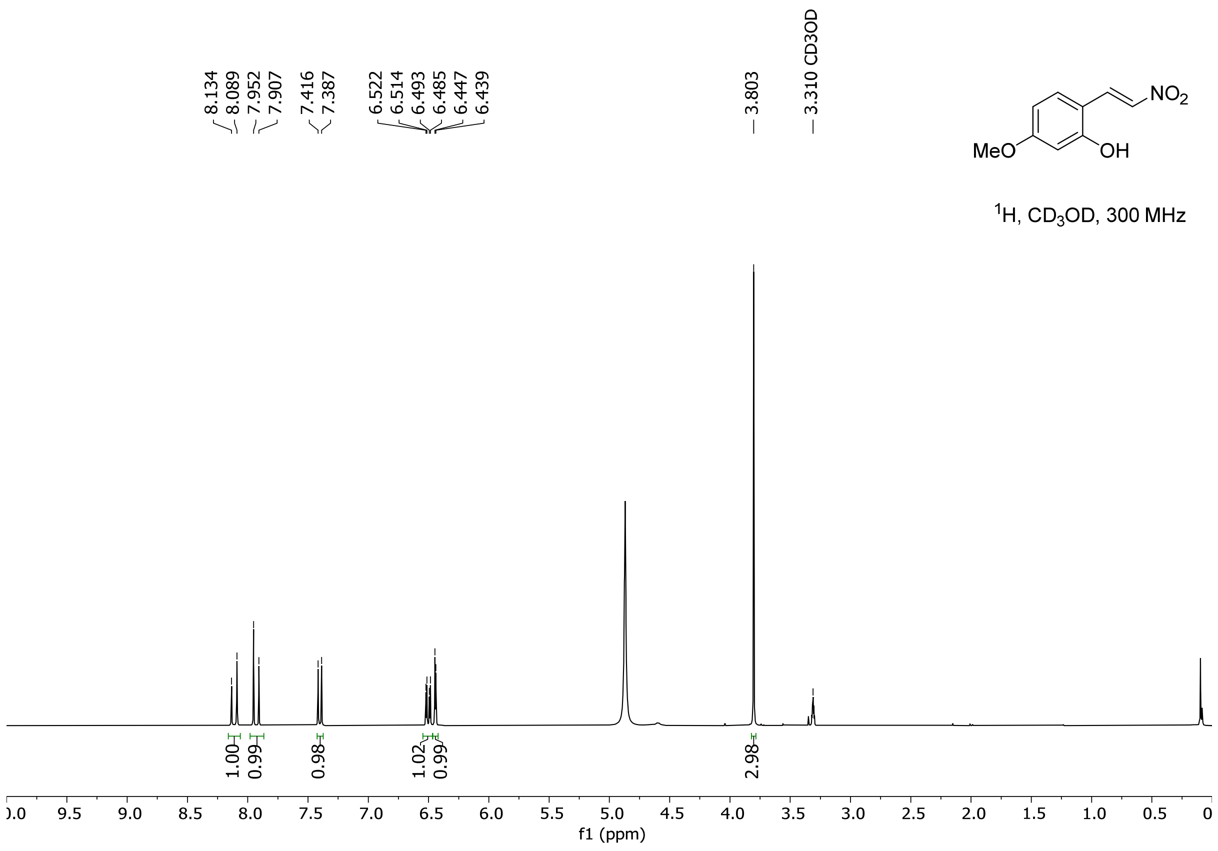
8.134
8.089
7.952
7.907
7.416
7.387
6.522
6.514
6.493
6.485
6.447
6.439
3.803
3.310 CD3OD
NO2
MeO
OH
1H, CD3OD, 300 MHz
1.00
0.99
0.98
1.02
0.99
2.98
f1 (ppm)

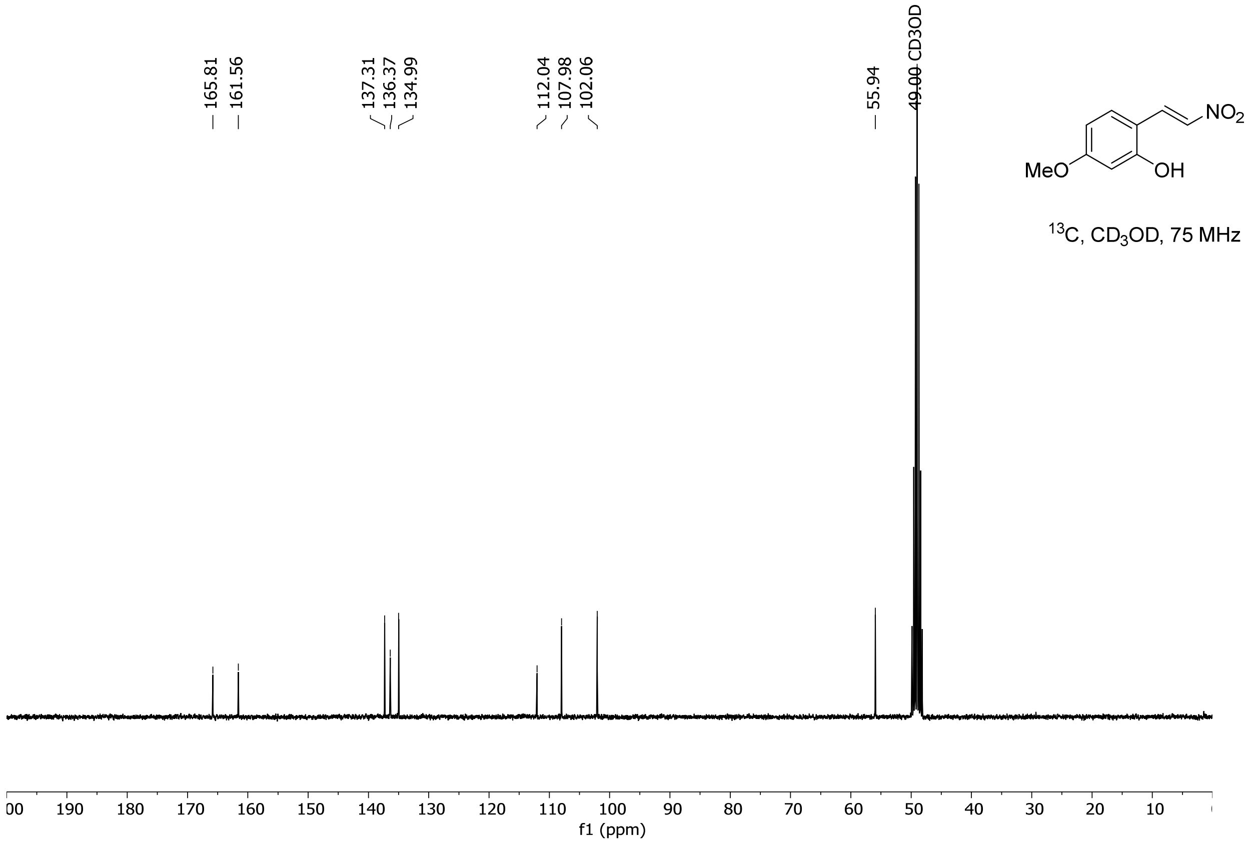
165.81
161.56
137.31
136.37
134.99
112.04
107.98
102.06
55.94
49.00 CD3OD
NO2
MeO
OH
13C, CD3OD, 75 MHz
f1 (ppm)

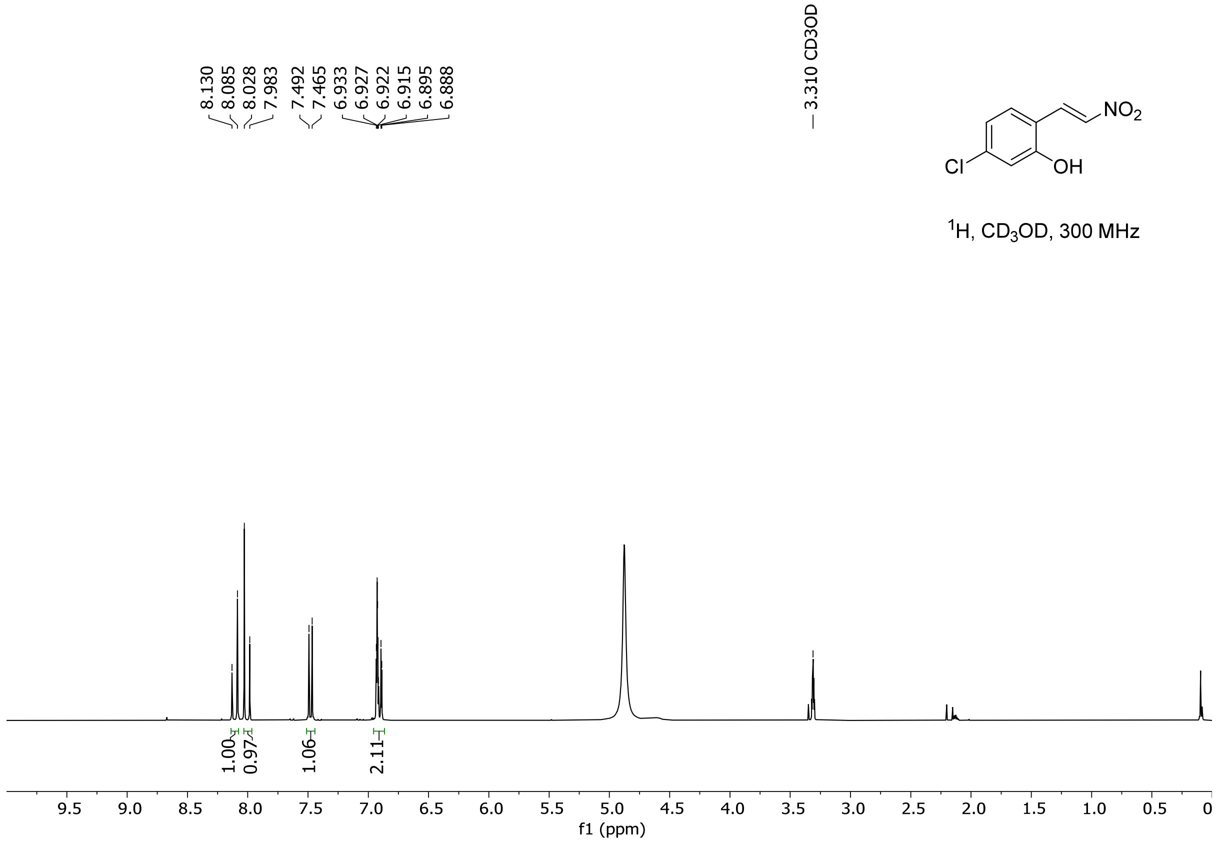
8.130
8.085
8.028
7.983
7.492
7.465
6.933
6.927
6.922
6.915
6.895
6.888
3.310 CD3OD
NO2
Cl
OH
1H, CD3OD, 300 MHz
1.00
0.97
1.06
2.11
9.5 9.0 8.5 8.0 7.5 7.0 6.5 6.0 5.5 5.0 4.5 4.0 3.5 3.0 2.5 2.0 1.5 1.0 0.5 0
f1 (ppm)

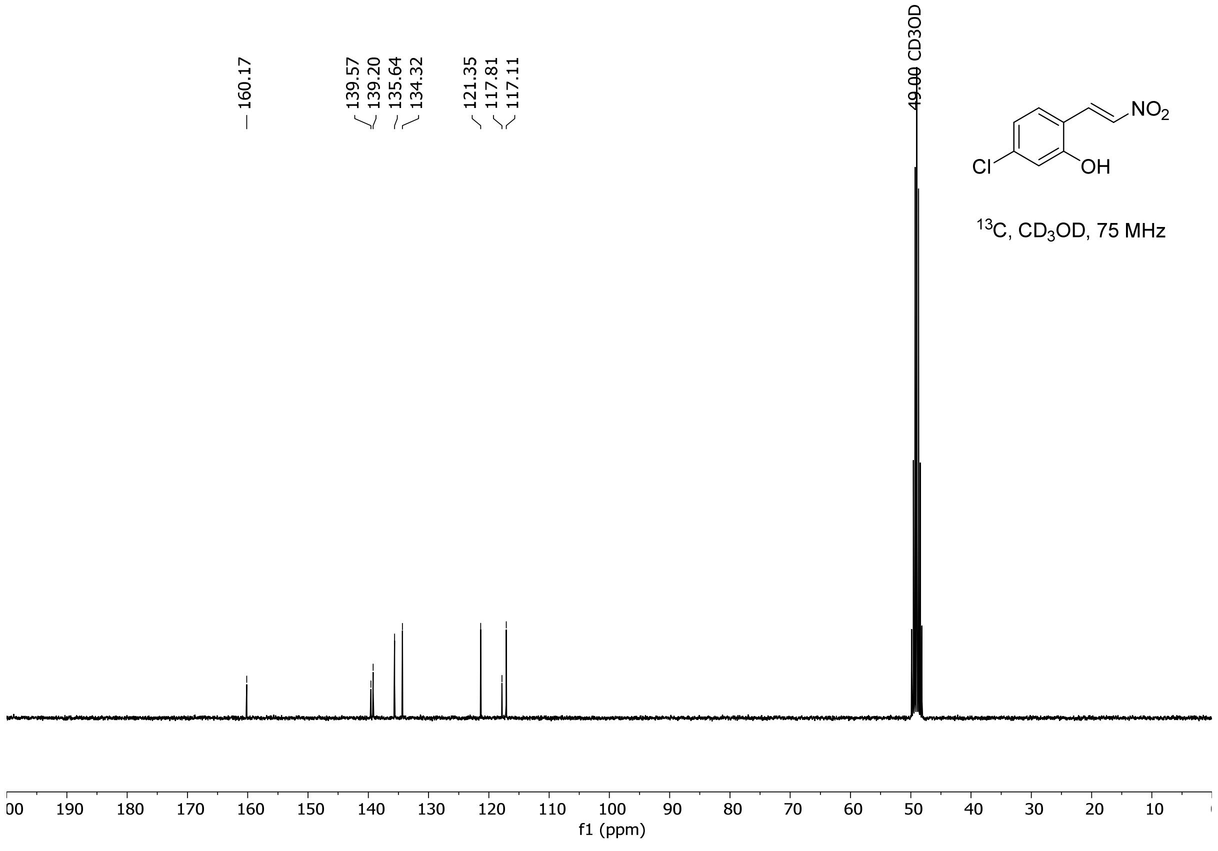
160.17
139.57
139.20
135.64
134.32
121.35
117.81
117.11
49.00 CD3OD
NO2
Cl
OH
13C, CD3OD, 75 MHz
190 180 170 160 150 140 130 120 110 100 90 80 70 60 50 40 30 20 10
f1 (ppm)

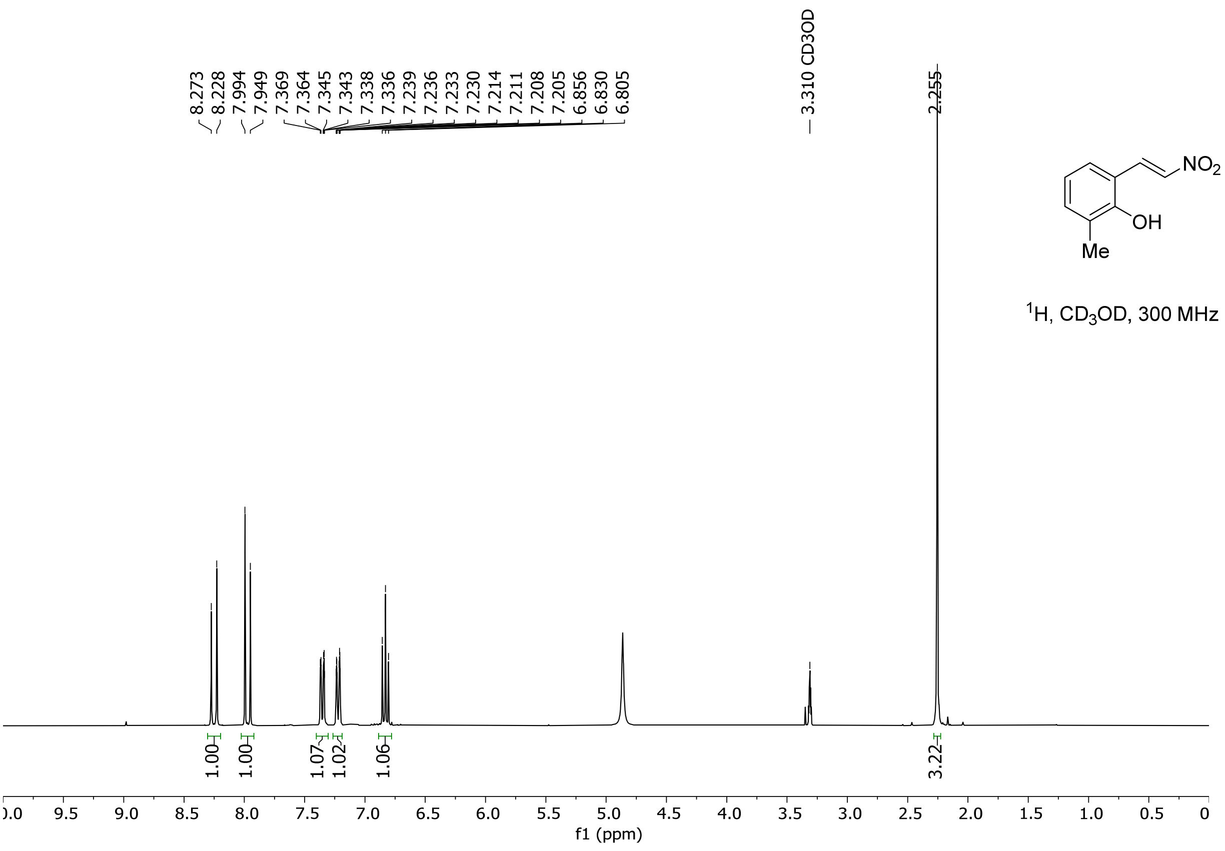

8.273
8.228
7.994
7.949
7.369
7.364
7.345
7.343
7.338
7.336
7.239
7.236
7.233
7.230
7.214
7.211
7.208
7.205
6.856
6.830
6.805
3.310 CD3OD
2.255
NO2
OH
Me
1H, CD3OD, 300 MHz
1.00
1.00
1.07
1.02
1.06
3.22
f1 (ppm)

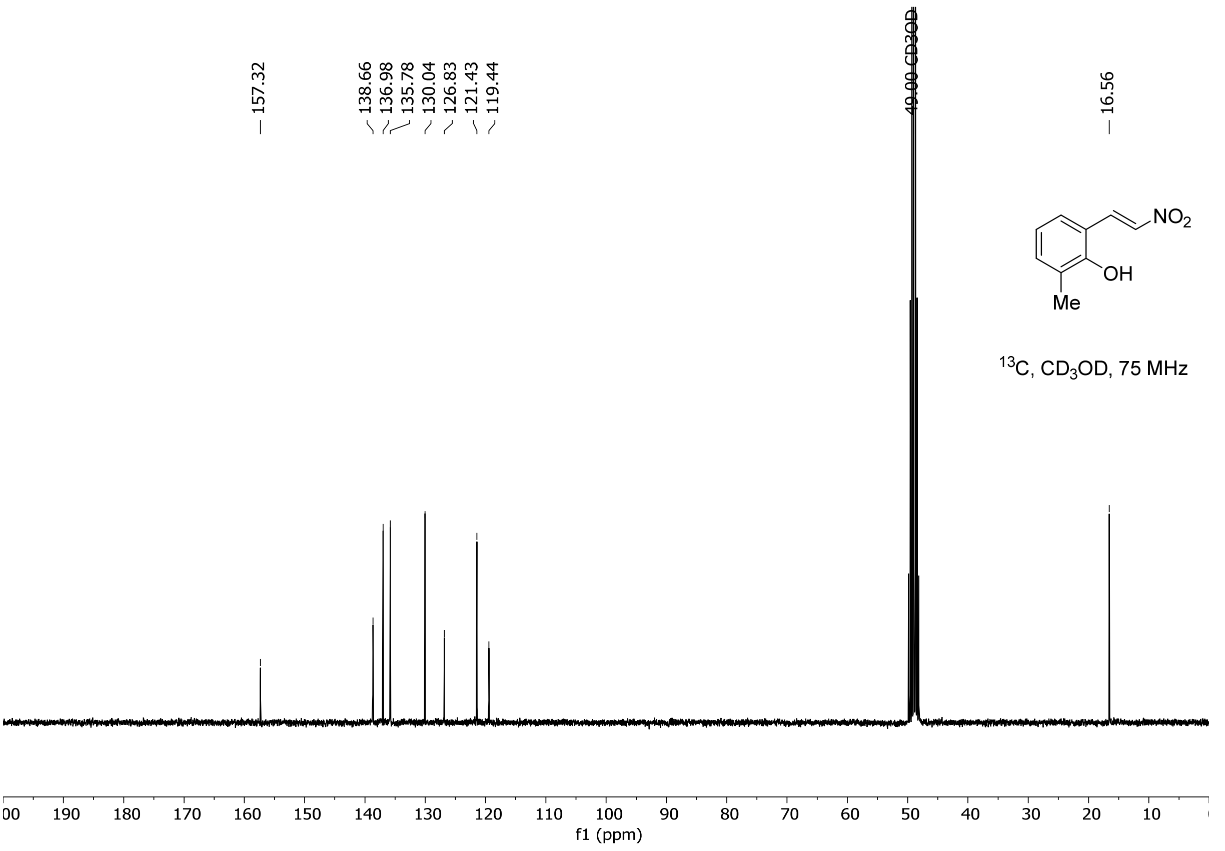

157.32
138.66
136.98
135.78
130.04
126.83
121.43
119.44
49.00 CD3OD
16.56
NO2
OH
Me
13C, CD3OD, 75 MHz
f1 (ppm)

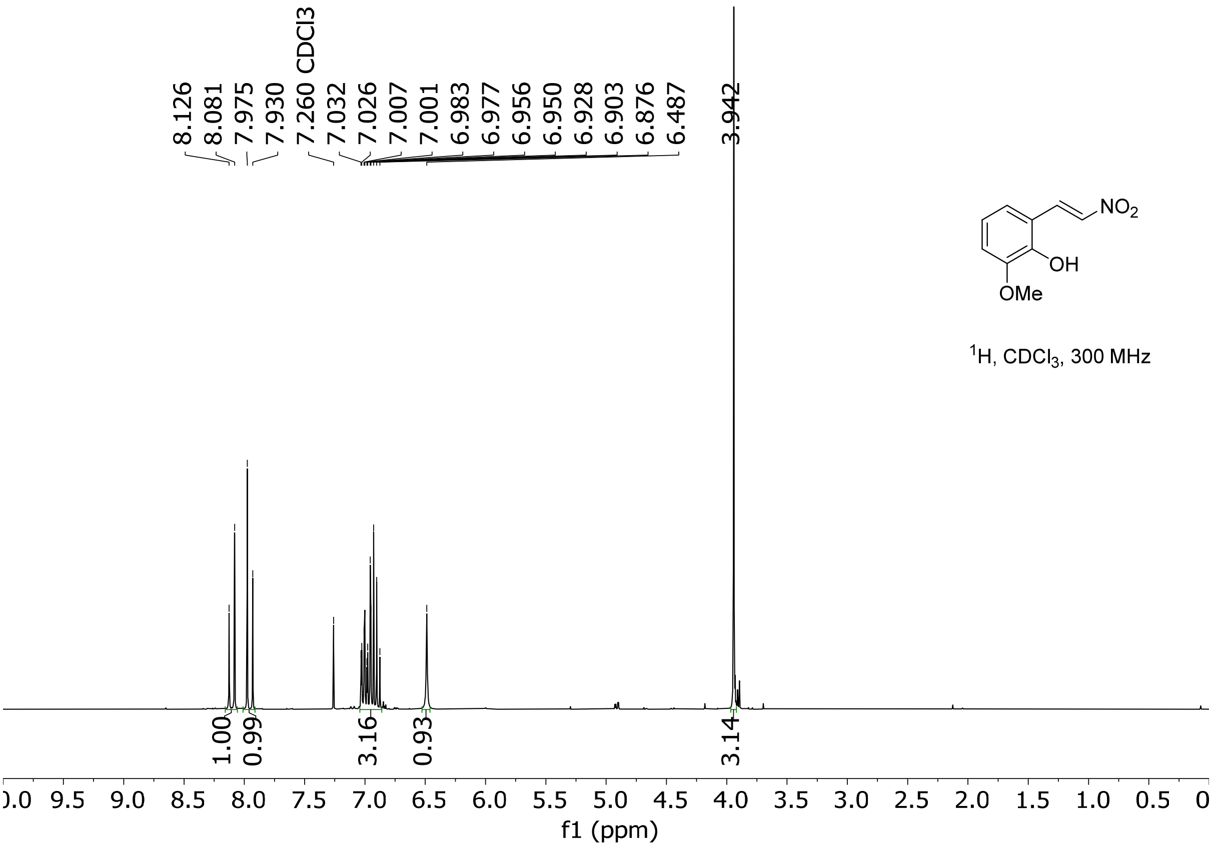
8.126
8.081
7.975
7.930
7.260 CDCl3
7.032
7.026
7.007
7.001
6.983
6.977
6.956
6.950
6.928
6.903
6.876
6.487
3.942
NO2
OH
OMe
1H, CDCl3, 300 MHz
1.00
0.99
3.16
0.93
3.14
f1 (ppm)

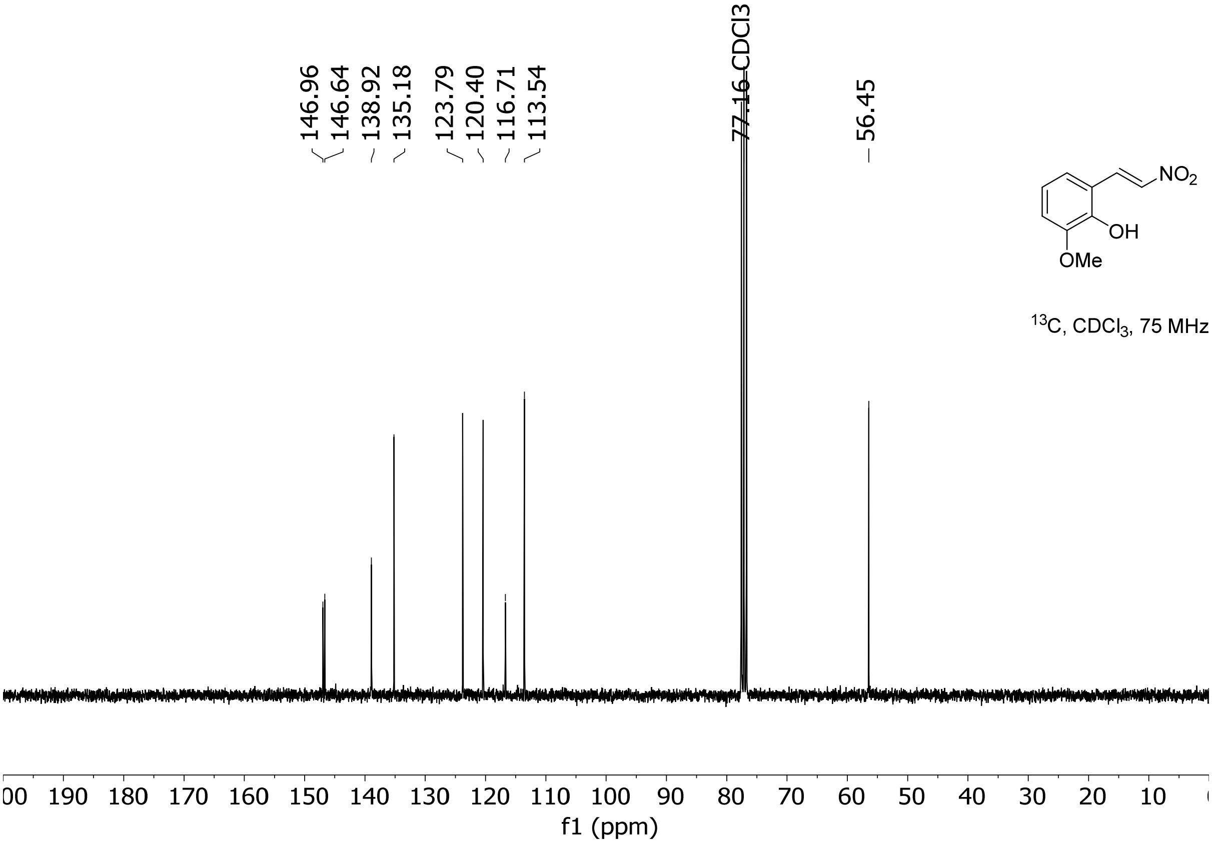
146.96
146.64
138.92
135.18
123.79
120.40
116.71
113.54
77.16 CDCl3
56.45
NO2
OH
OMe
13C, CDCl3, 75 MHz
f1 (ppm)

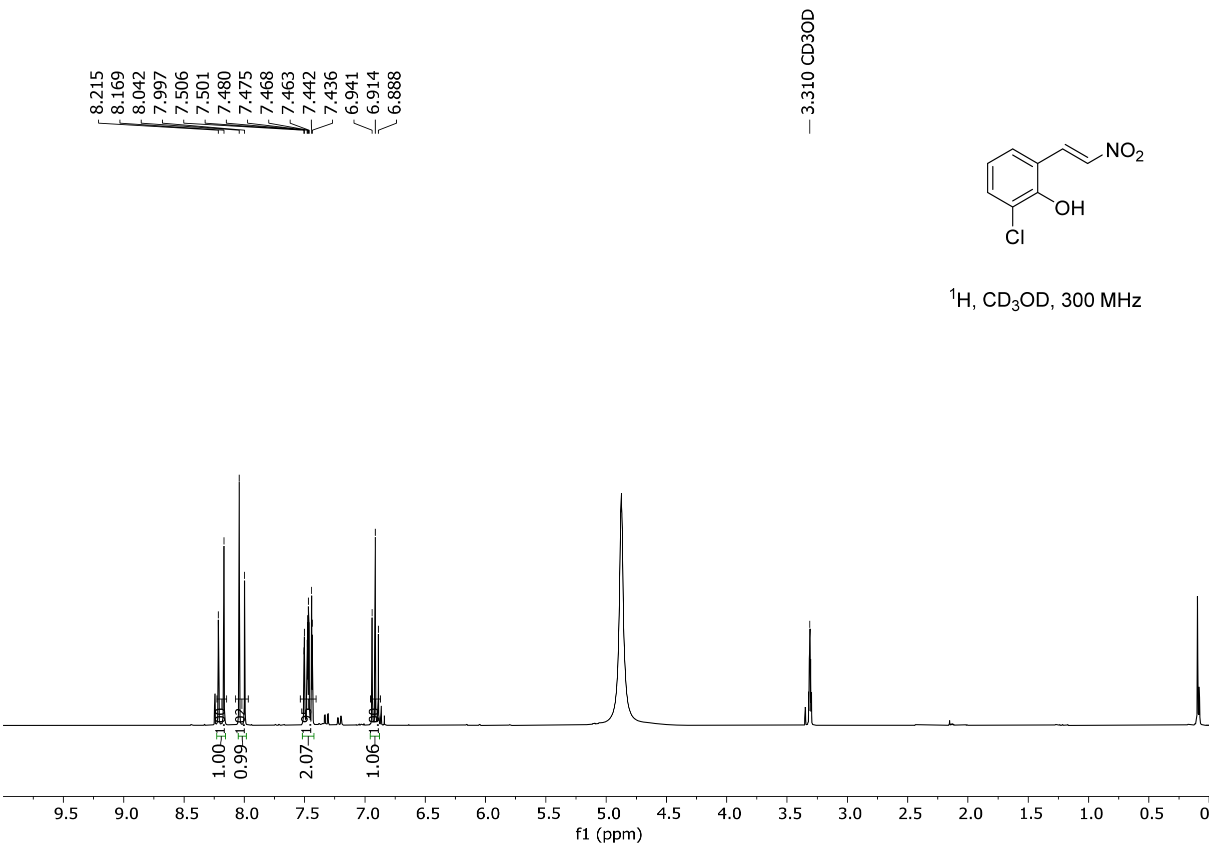
8.215
8.169
8.042
7.997
7.506
7.501
7.480
7.475
7.468
7.463
7.442
7.436
6.941
6.914
6.888
3.310 CD3OD
NO2
OH
Cl
1H, CD3OD, 300 MHz
1.00
0.99
2.07
1.06
9.5 9.0 8.5 8.0 7.5 7.0 6.5 6.0 5.5 5.0 4.5 4.0 3.5 3.0 2.5 2.0 1.5 1.0 0.5 0
f1 (ppm)

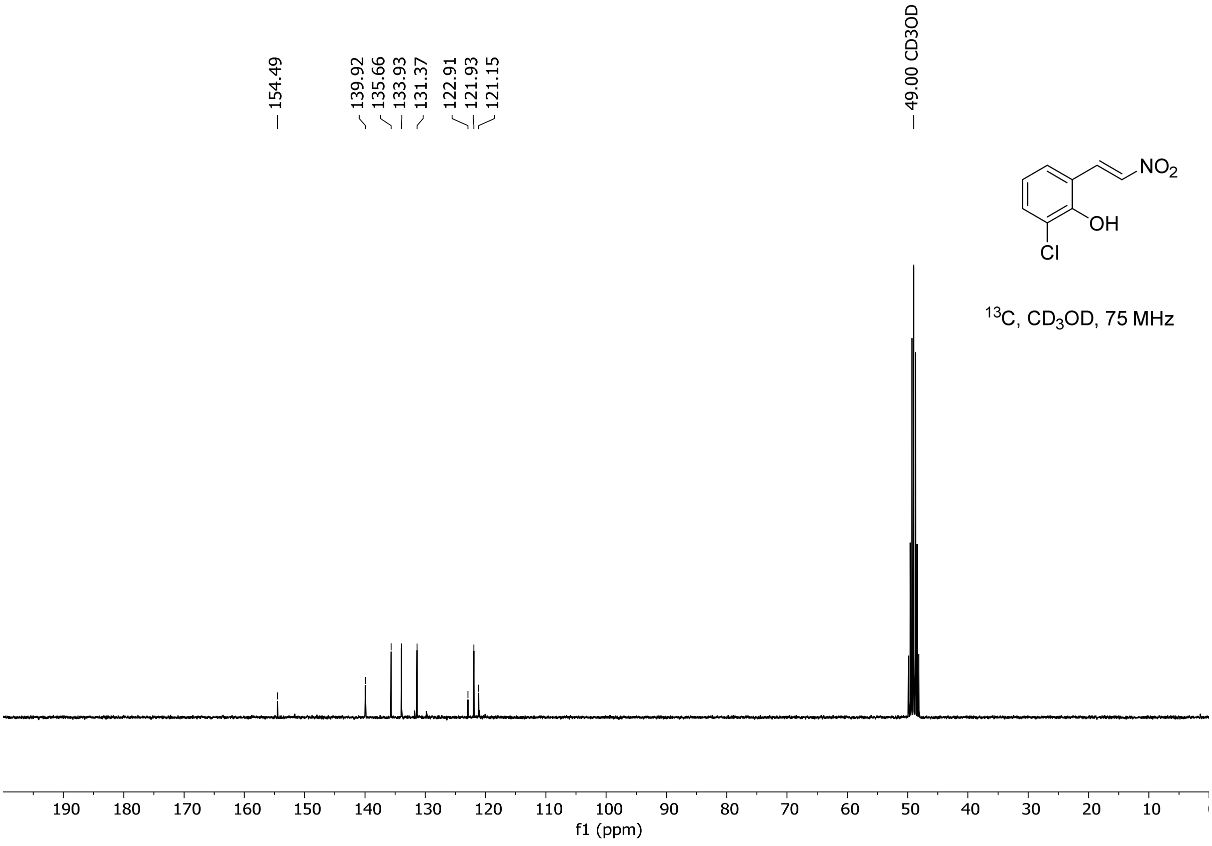
154.49
139.92
135.66
133.93
131.37
122.91
121.93
121.15
49.00 CD3OD
NO2
OH
Cl
13C, CD3OD, 75 MHz
190 180 170 160 150 140 130 120 110 100 90 80 70 60 50 40 30 20 10
f1 (ppm)

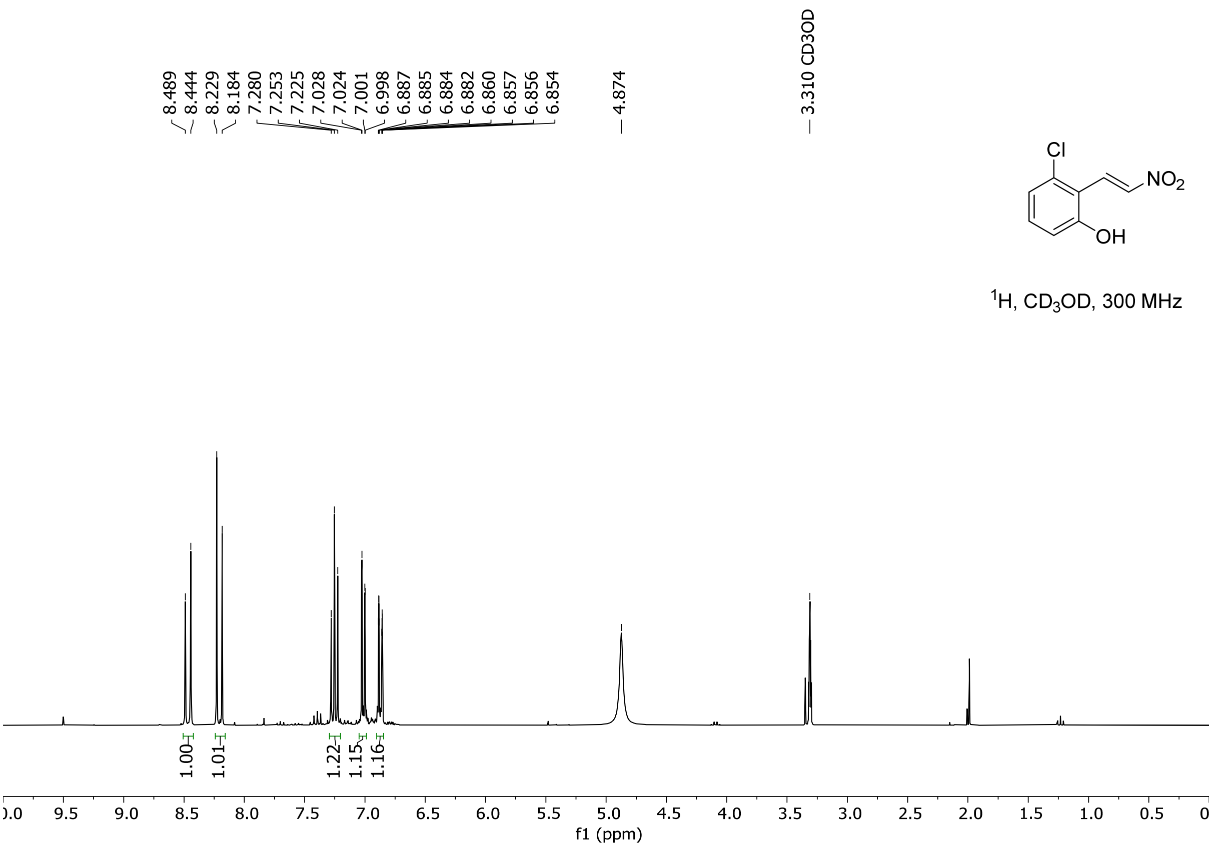
8.489
8.444
8.229
8.184
7.280
7.253
7.225
7.028
7.024
7.001
6.998
6.887
6.885
6.884
6.882
6.860
6.857
6.856
6.854
4.874
3.310 CD3OD
Cl
NO2
OH
1H, CD3OD, 300 MHz
1.00
1.01
1.22
1.15
1.16
f1 (ppm)

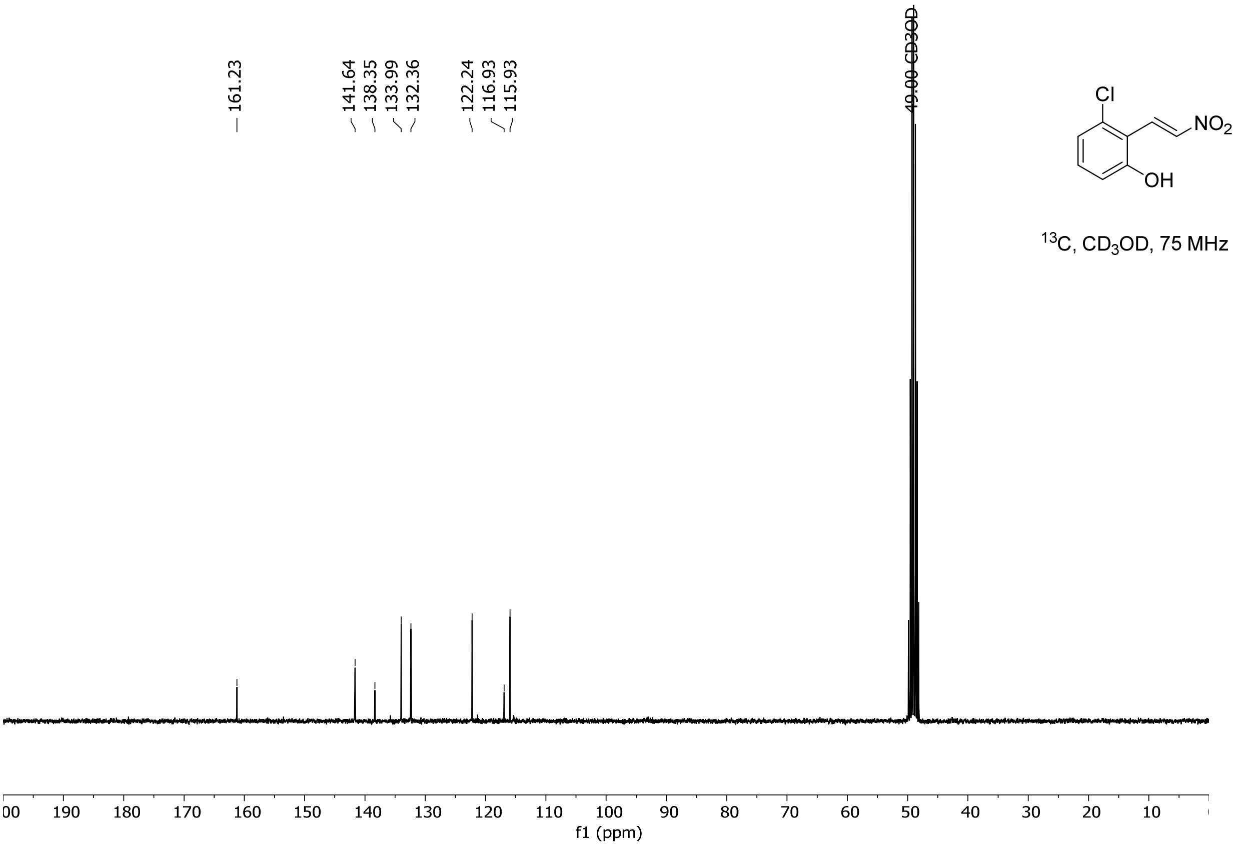
161.23
141.64
138.35
133.99
132.36
122.24
116.93
115.93
49.00 CD3OD
Cl
NO2
OH
13C, CD3OD, 75 MHz
f1 (ppm)

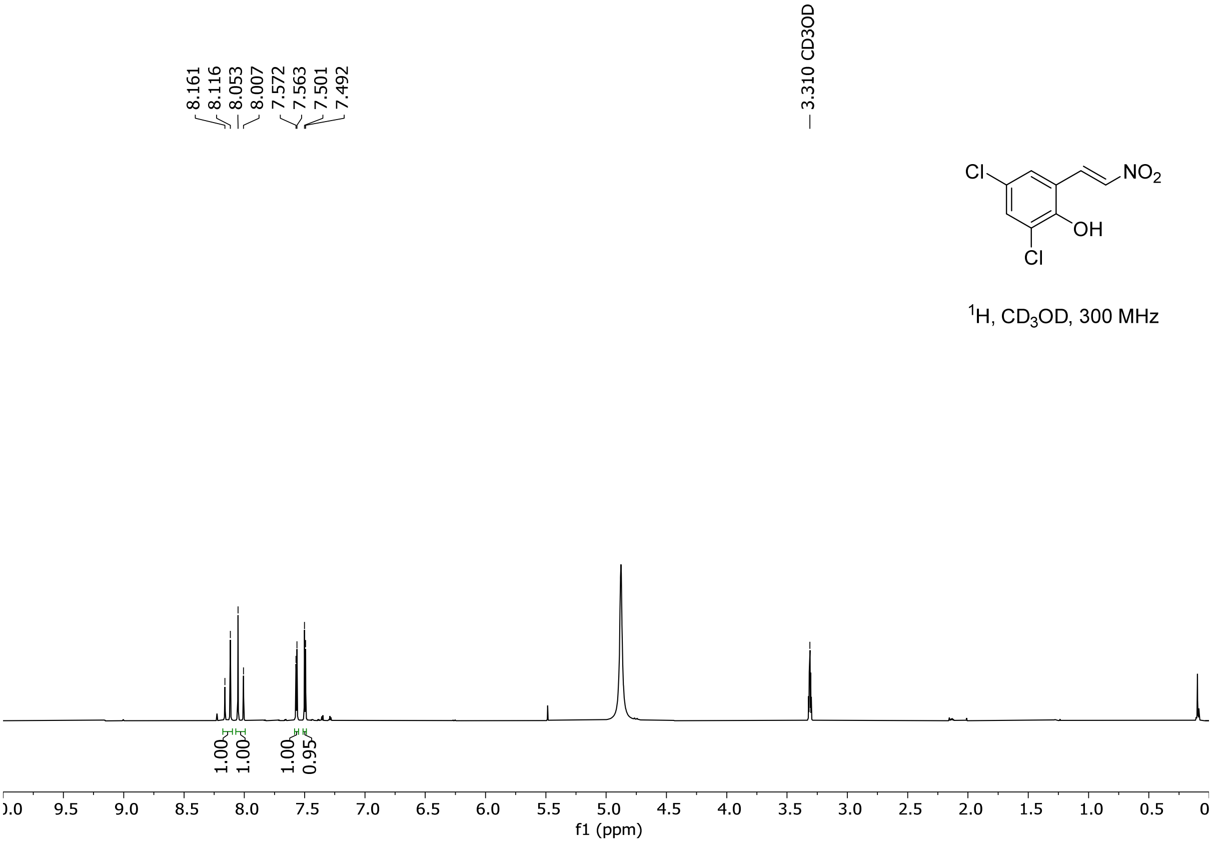
8.161
8.116
8.053
8.007
7.572
7.563
7.501
7.492
3.310 CD3OD
Cl
NO_2
OH
Cl
^{1}H, CD_3OD, 300 MHz
1.00
1.00
1.00
0.95
f1 (ppm)

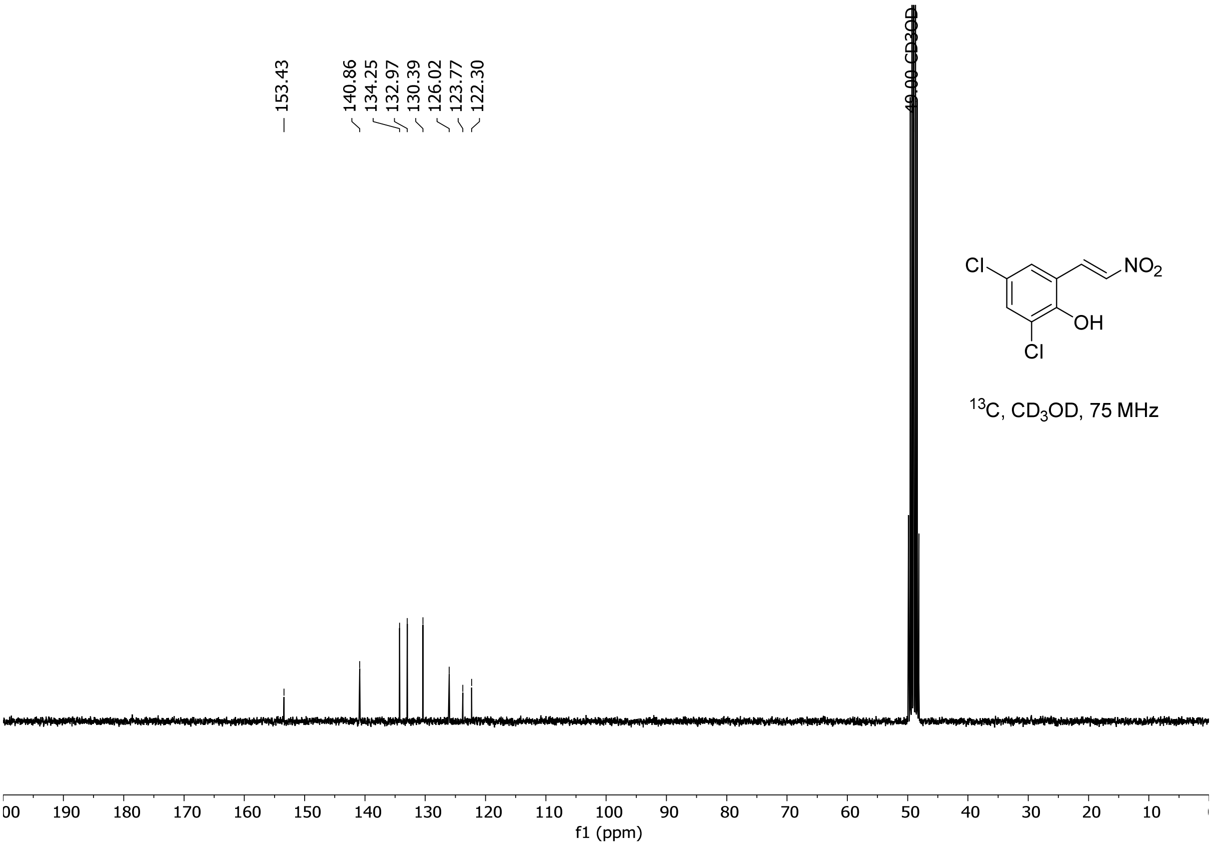
153.43
140.86
134.25
132.97
130.39
126.02
123.77
122.30
49.00 CD3OD
Cl
NO_2
OH
Cl
^{13}C, CD_3OD, 75 MHz
f1 (ppm)

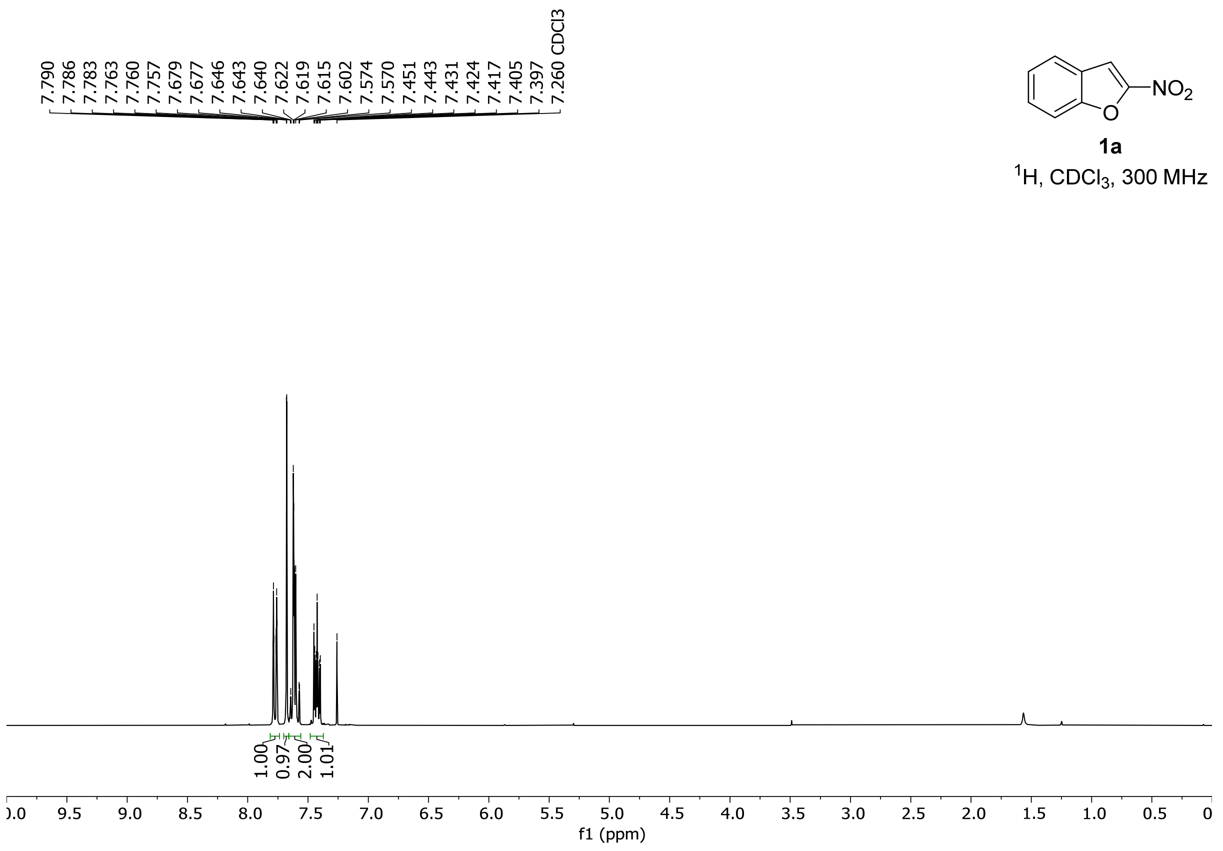
7.790
7.786
7.783
7.763
7.760
7.757
7.679
7.677
7.646
7.643
7.640
7.622
7.619
7.615
7.602
7.574
7.570
7.451
7.443
7.431
7.424
7.417
7.405
7.397
7.260 CDCl3
NO2
O
1a
1H, CDCl3, 300 MHz
1.00
0.97
2.00
1.01
f1 (ppm)

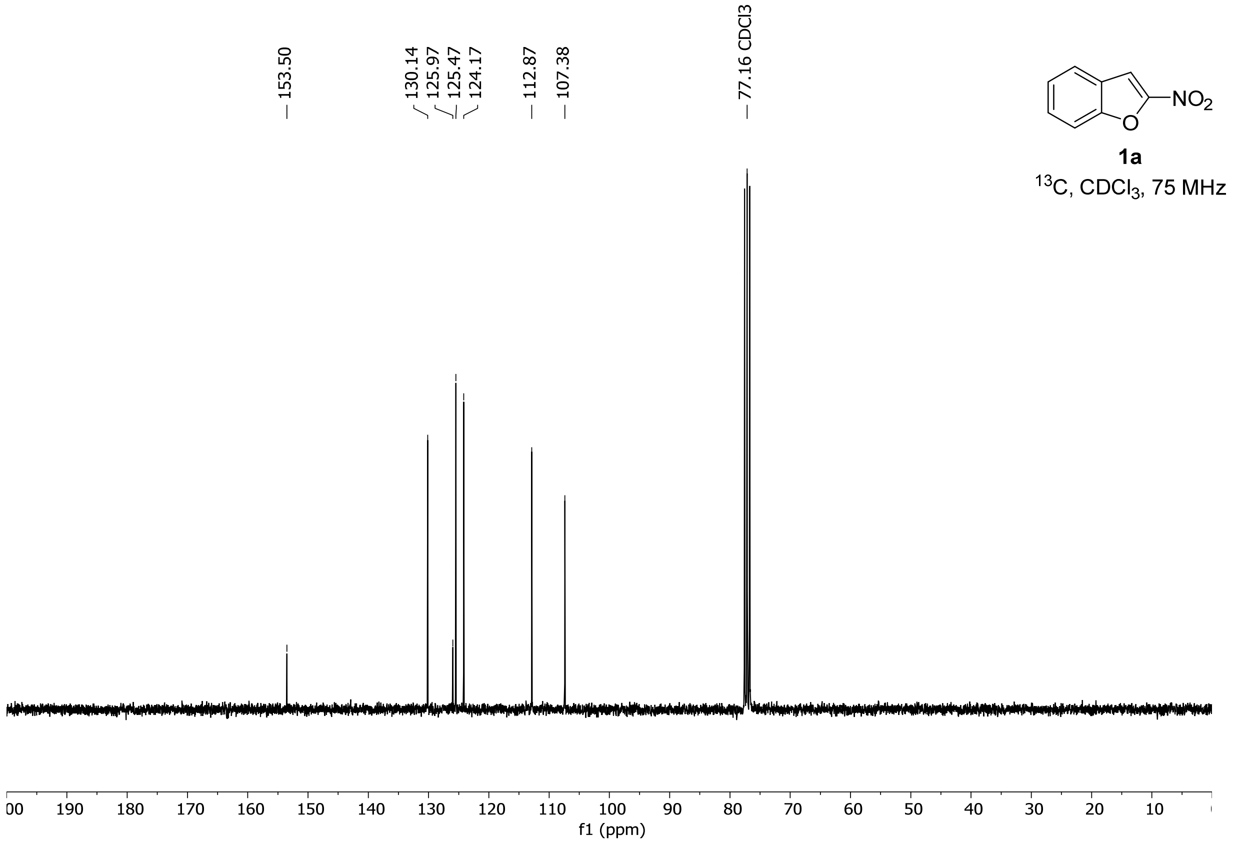
153.50
130.14
125.97
125.47
124.17
112.87
107.38
77.16 CDCl3
NO2
O
1a
13C, CDCl3, 75 MHz
f1 (ppm)

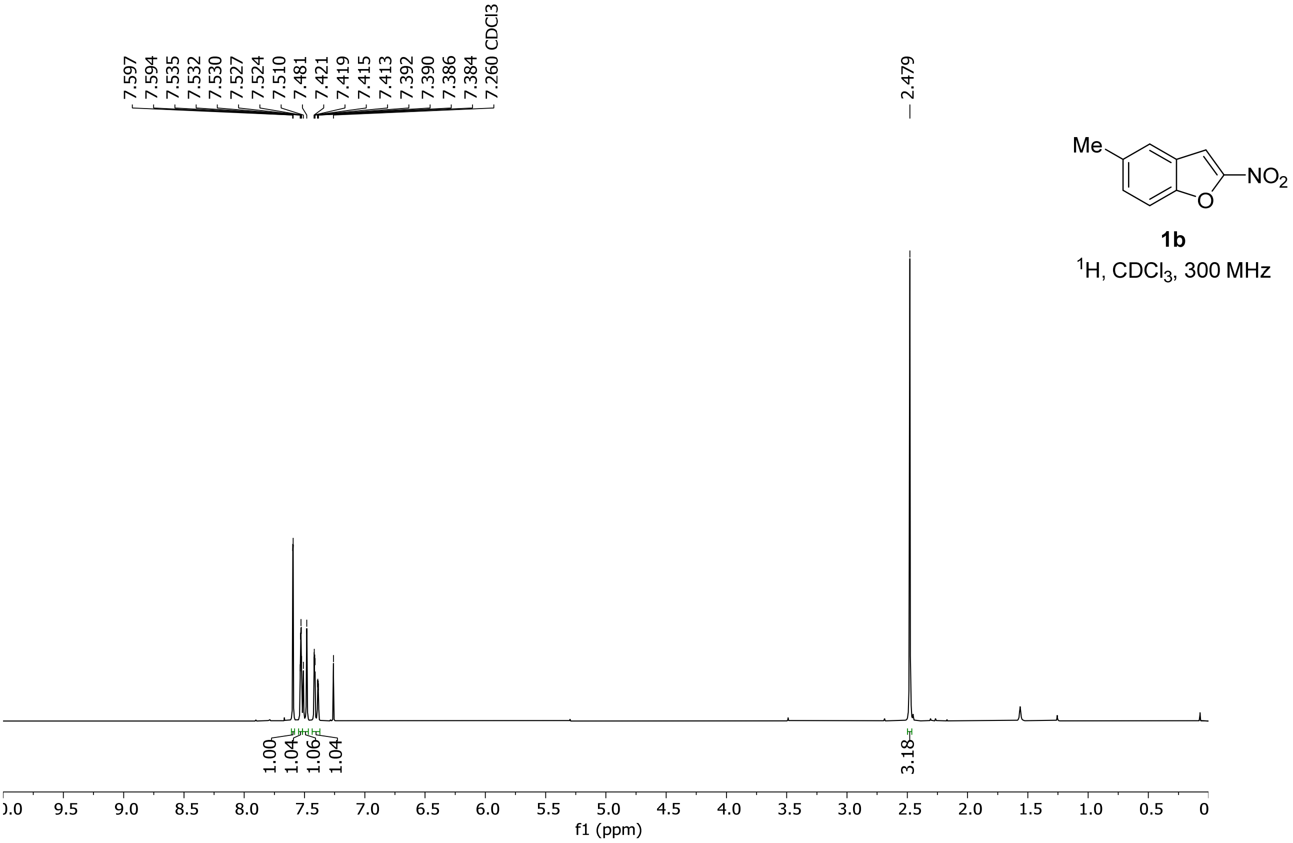
7.597
7.594
7.535
7.532
7.530
7.527
7.524
7.510
7.481
7.421
7.419
7.415
7.413
7.392
7.390
7.386
7.384
7.260 CDCl3
2.479
Me
NO2
O
1b
1H, CDCl3, 300 MHz
1.00
1.04
1.06
1.04
3.18
f1 (ppm)

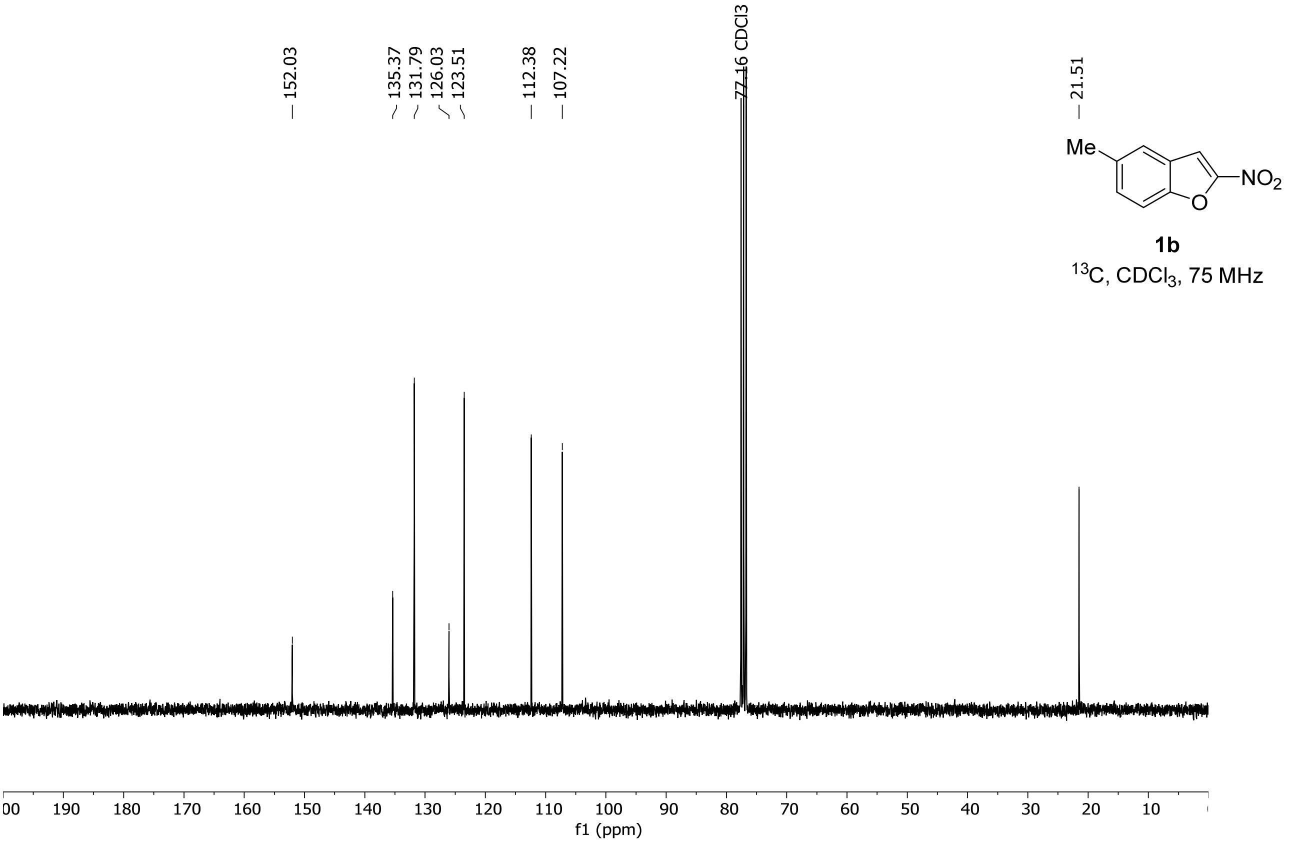
152.03
135.37
131.79
126.03
123.51
112.38
107.22
77.16 CDCl3
21.51
Me
NO2
O
1b
13C, CDCl3, 75 MHz
f1 (ppm)

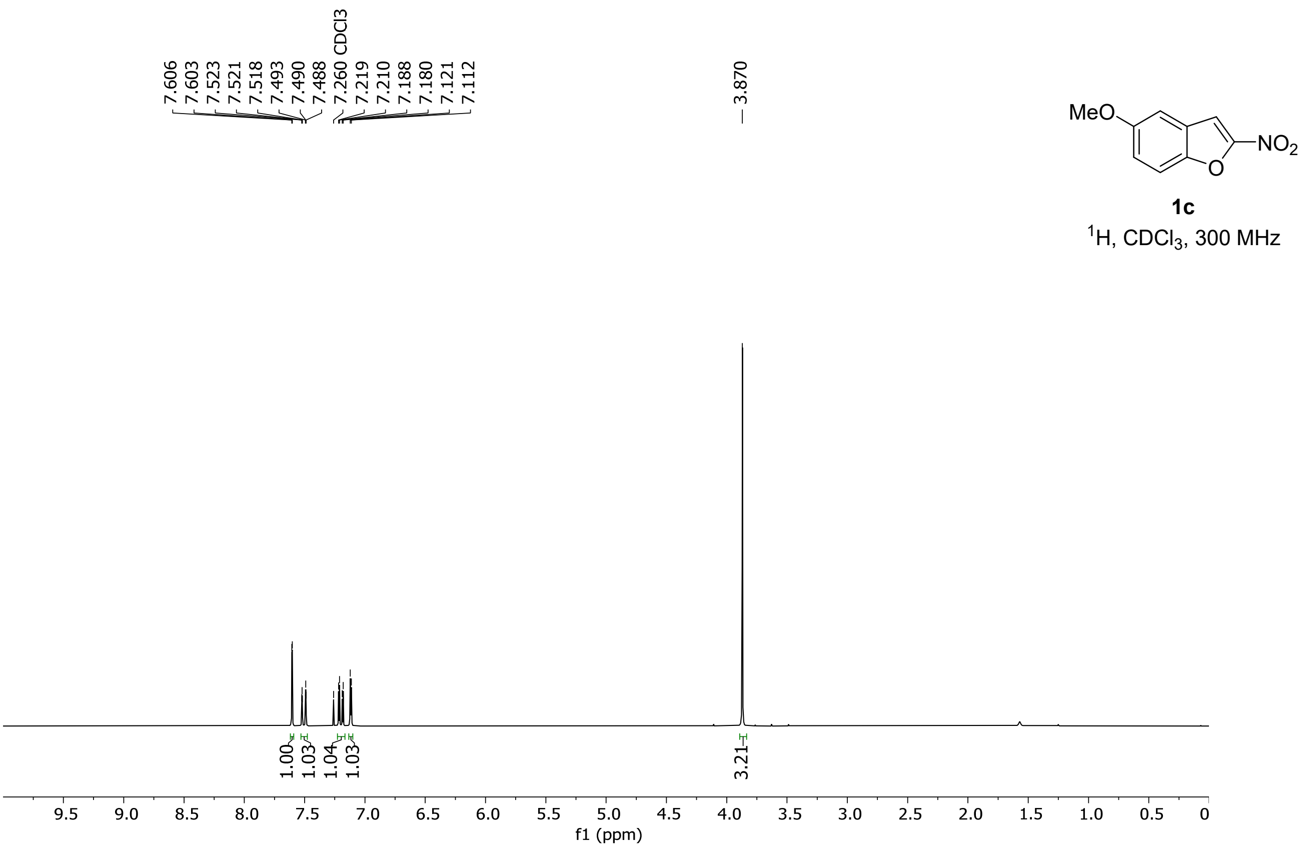

1c

^{1}H, $CDCl_3$, 300 MHz

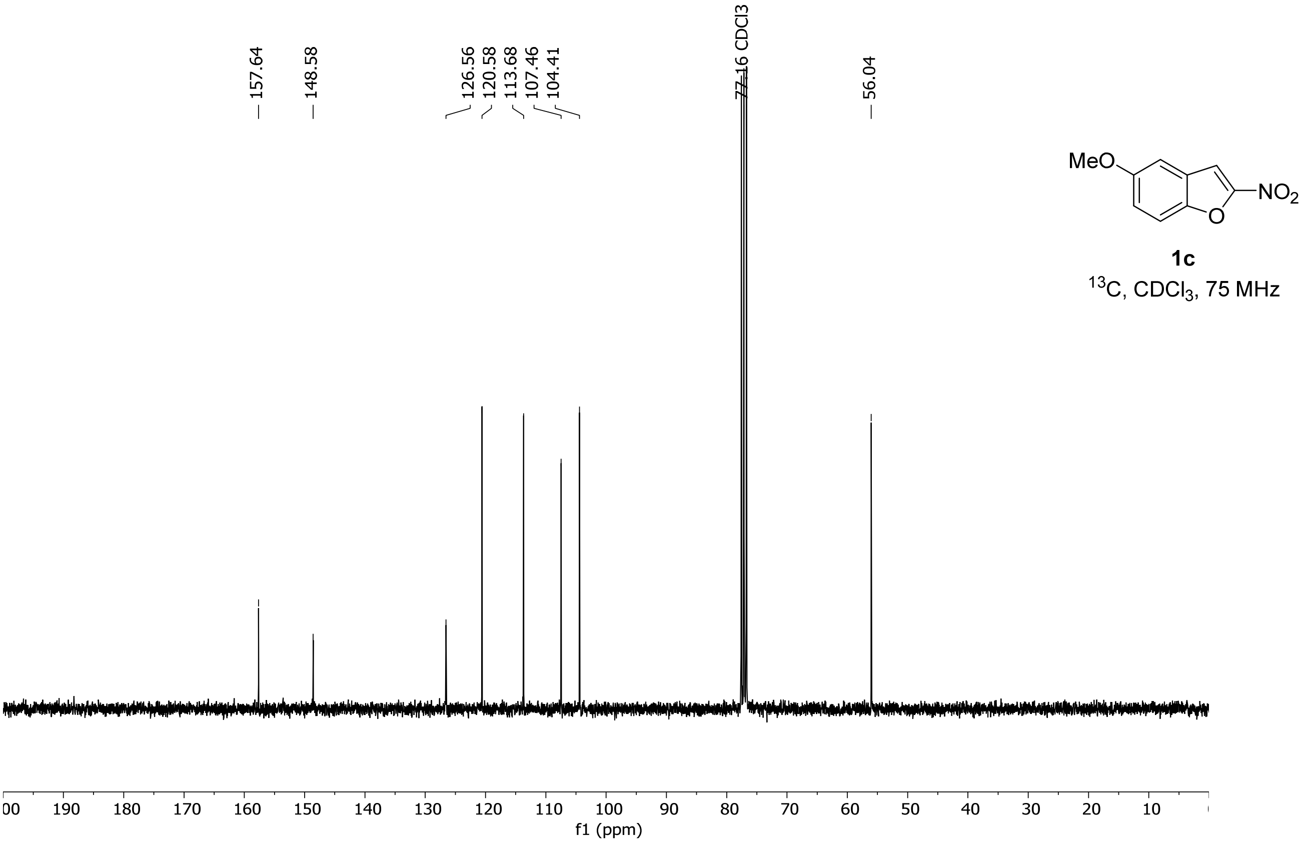

1c

^{13}C, $CDCl_3$, 75 MHz

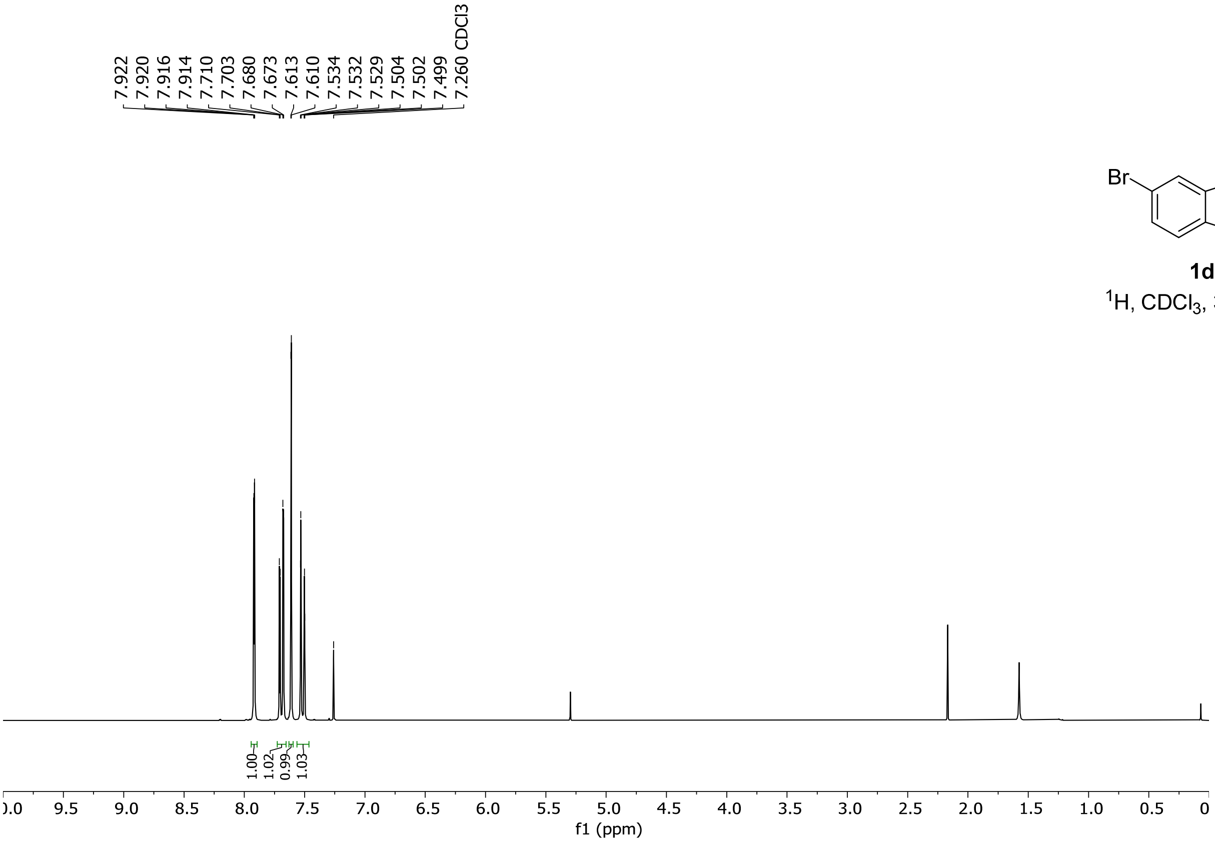

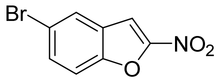

1d

^{1}H, $CDCl_3$, 300 MHz

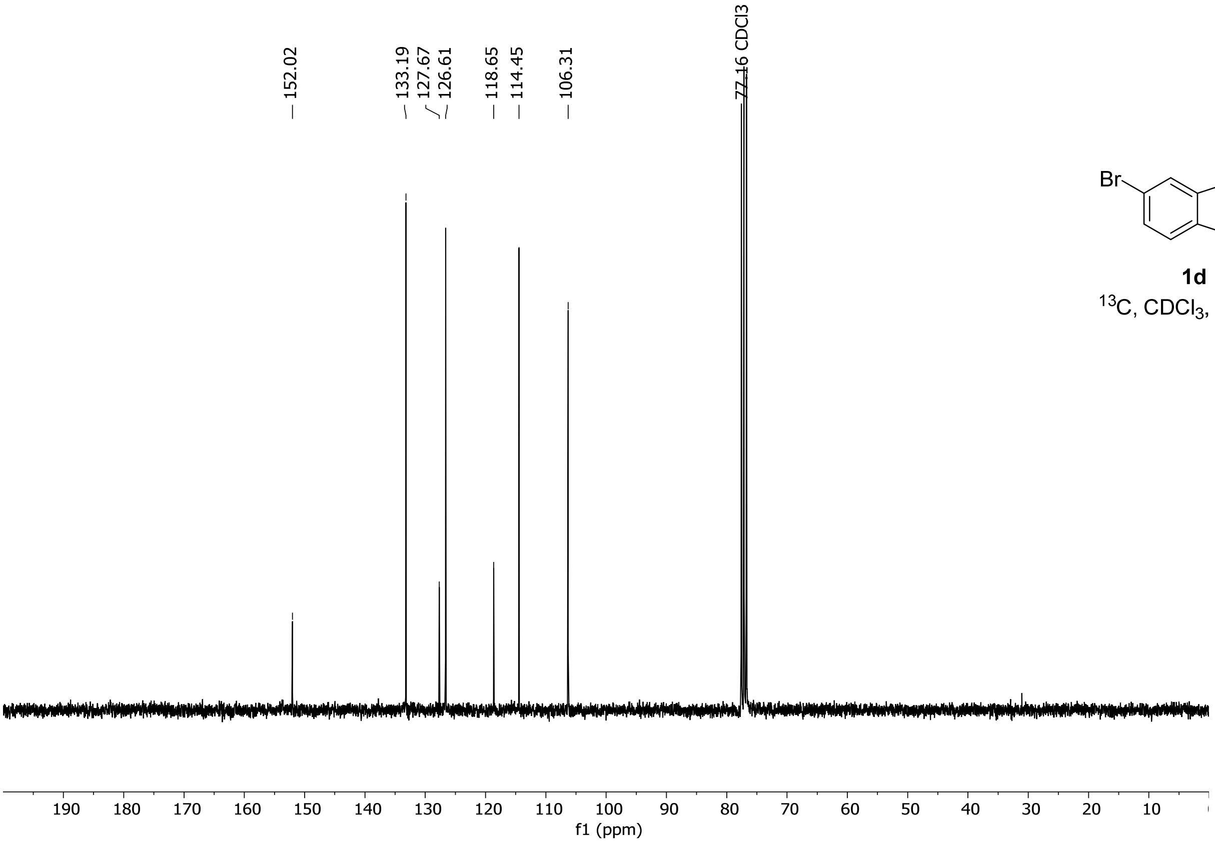

1d

^{13}C, $CDCl_3$, 75 MHz

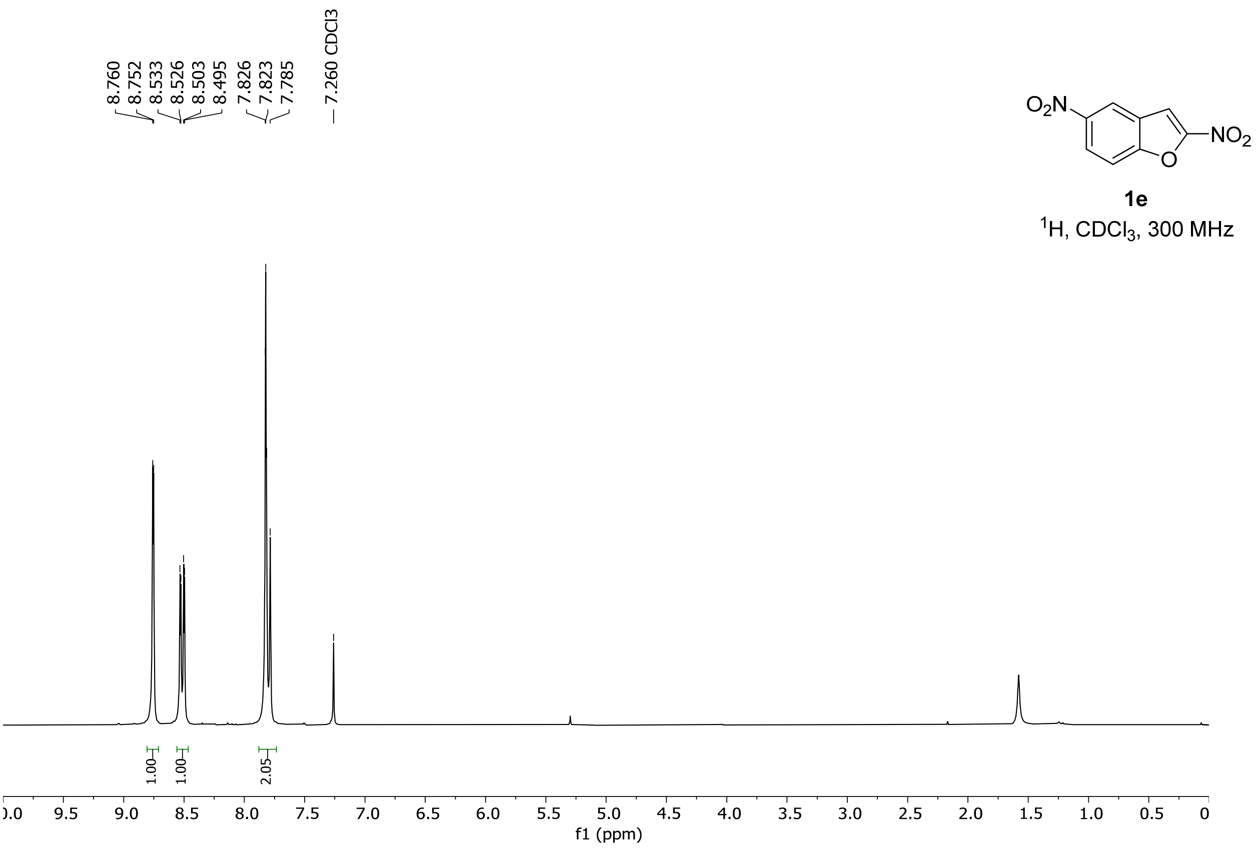
8.760
8.752
8.533
8.526
8.503
8.495
7.826
7.823
7.785
7.260 CDCl3
O2N
NO2
O
1e
1H, CDCl3, 300 MHz
1.00
1.00
2.05
f1 (ppm)

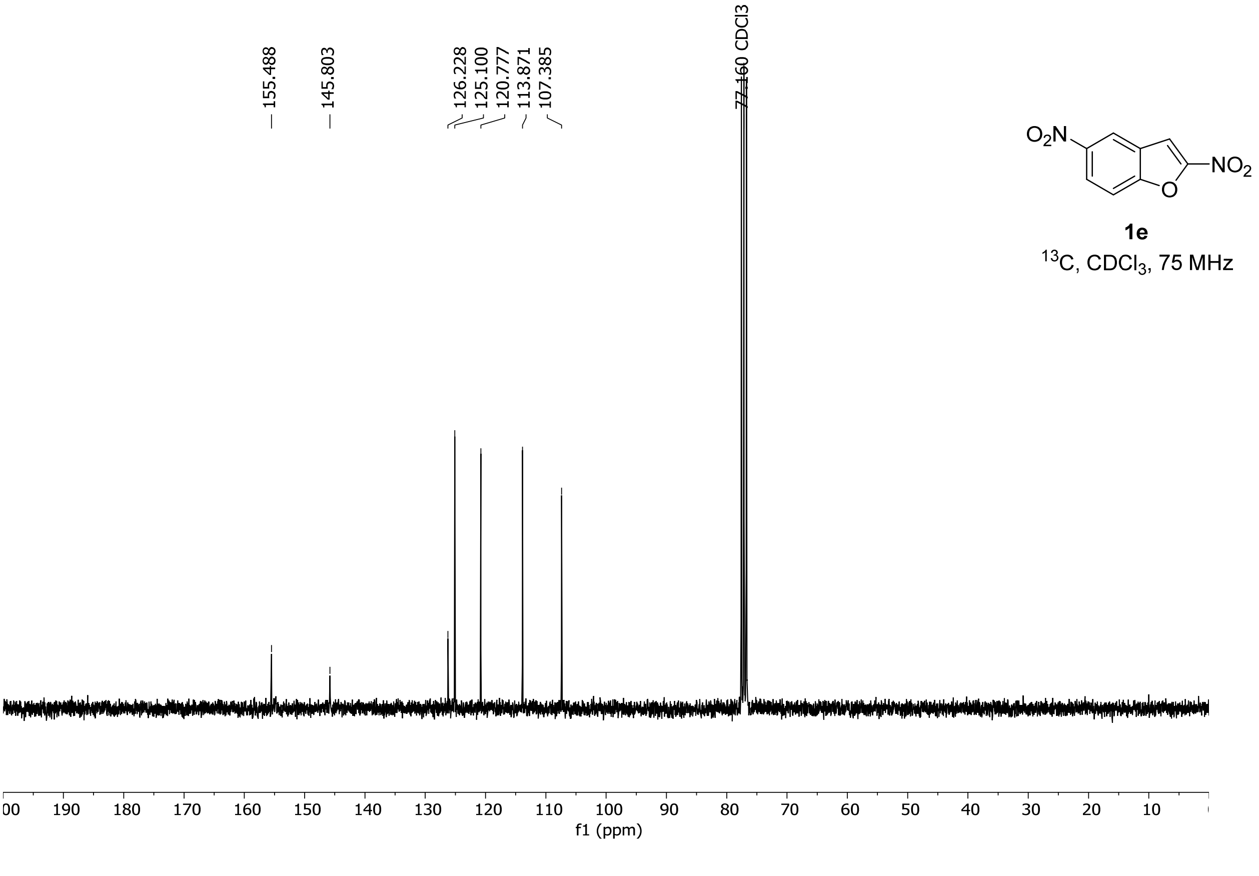
155.488
145.803
126.228
125.100
120.777
113.871
107.385
77.160 CDCl3
O2N
NO2
O
1e
13C, CDCl3, 75 MHz
f1 (ppm)

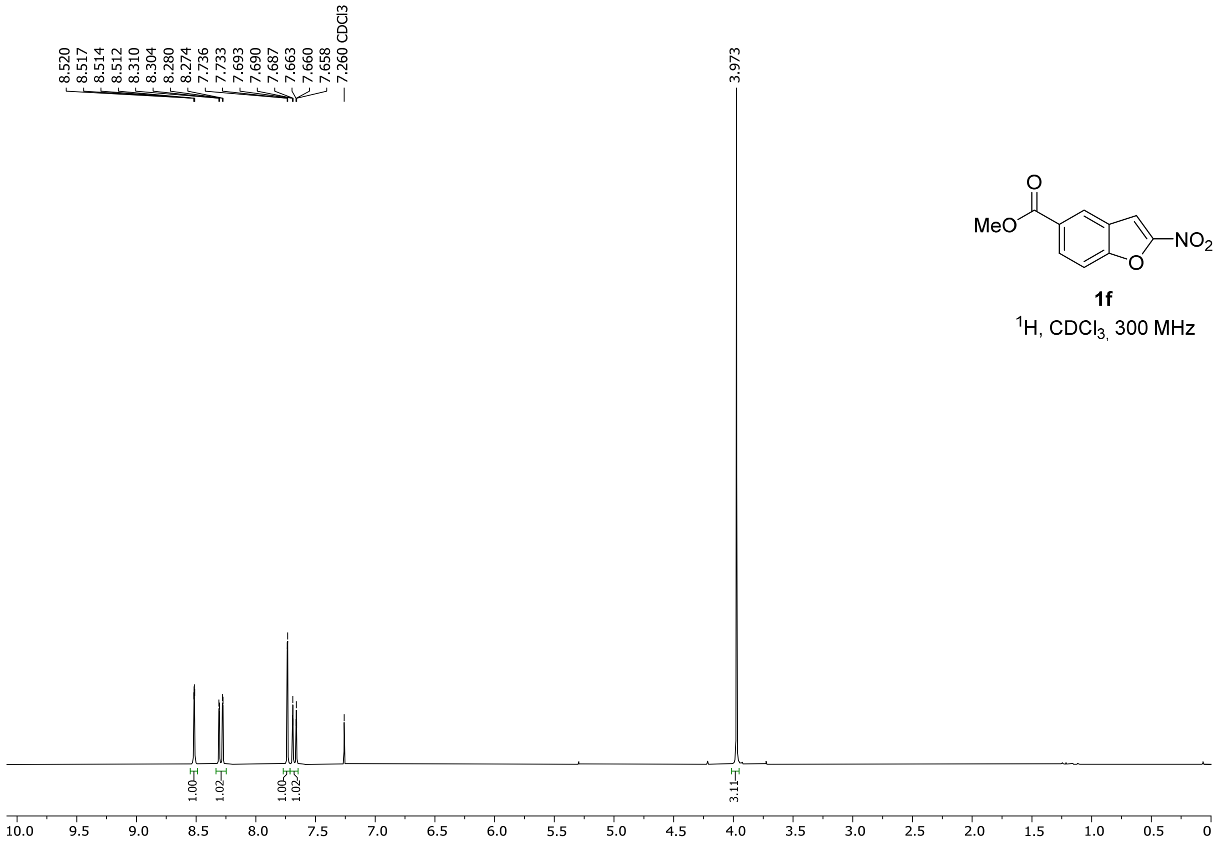
8.520
8.517
8.514
8.512
8.310
8.304
8.280
8.274
7.736
7.733
7.693
7.690
7.687
7.663
7.660
7.658
7.260 CDCl3
3.973
MeO
O
NO2
1f
1H, CDCl3, 300 MHz
1.00
1.02
1.00
1.02
3.11
10.0
9.5
9.0
8.5
8.0
7.5
7.0
6.5
6.0
5.5
5.0
4.5
4.0
3.5
3.0
2.5
2.0
1.5
1.0
0.5
0

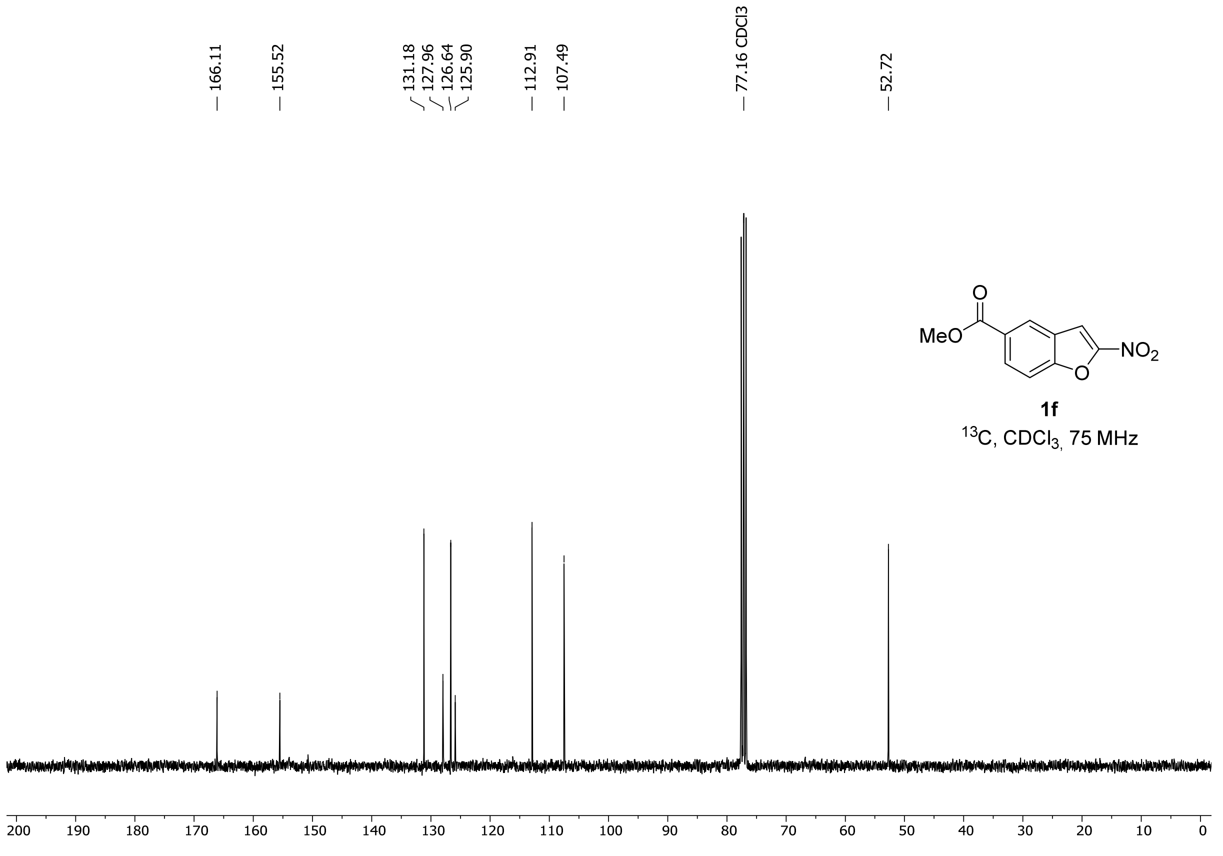
166.11
155.52
131.18
127.96
126.64
125.90
112.91
107.49
77.16 CDCl3
52.72
MeO
O
NO2
1f
13C, CDCl3, 75 MHz
200
190
180
170
160
150
140
130
120
110
100
90
80
70
60
50
40
30
20
10
0

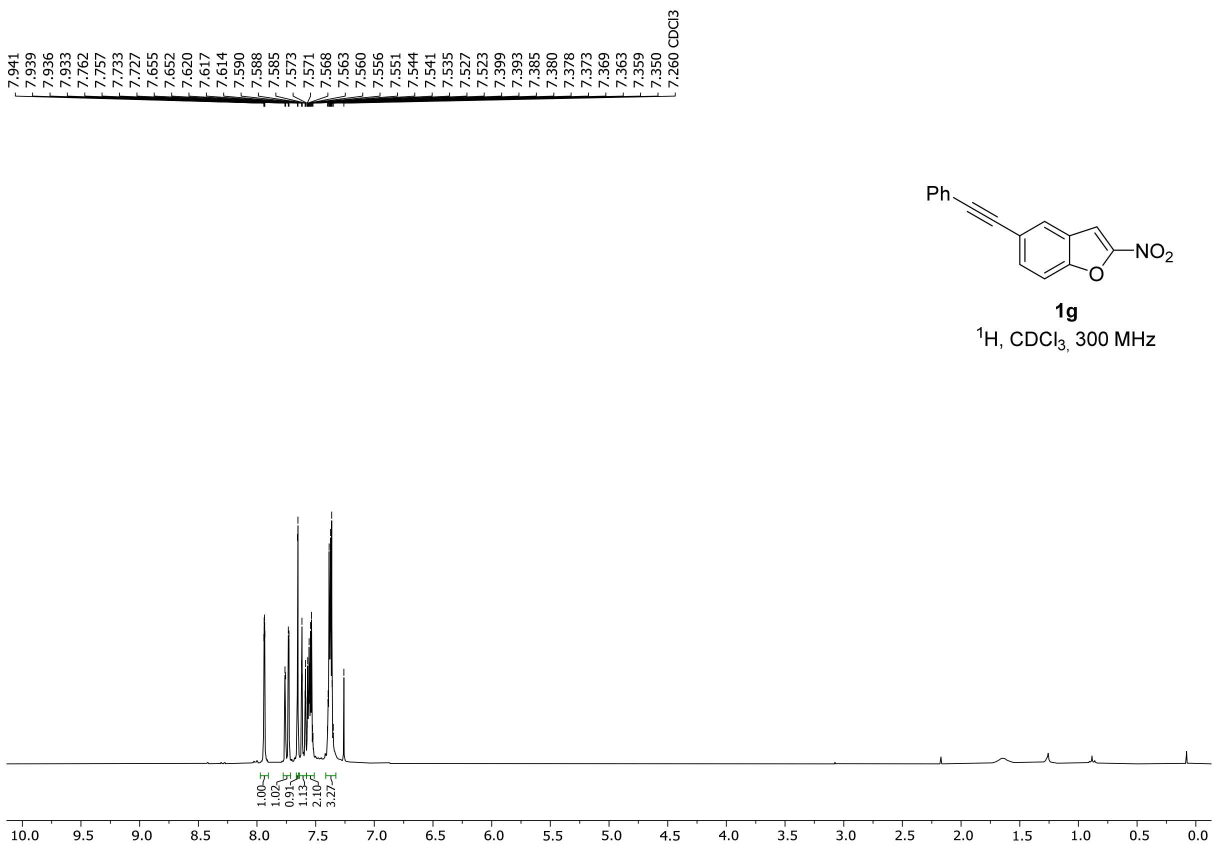

1g

^{1}H, $CDCl_3$, 300 MHz

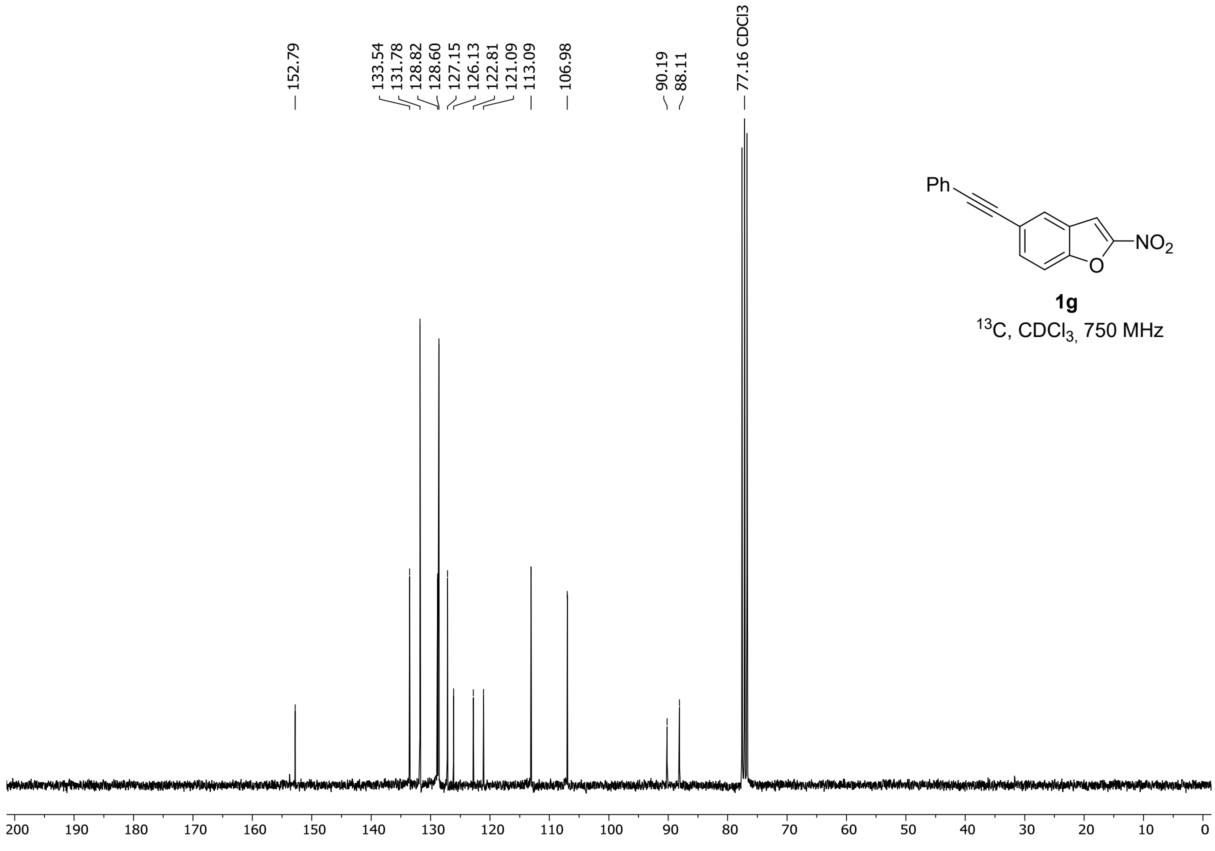

1g

^{13}C, $CDCl_3$, 750 MHz

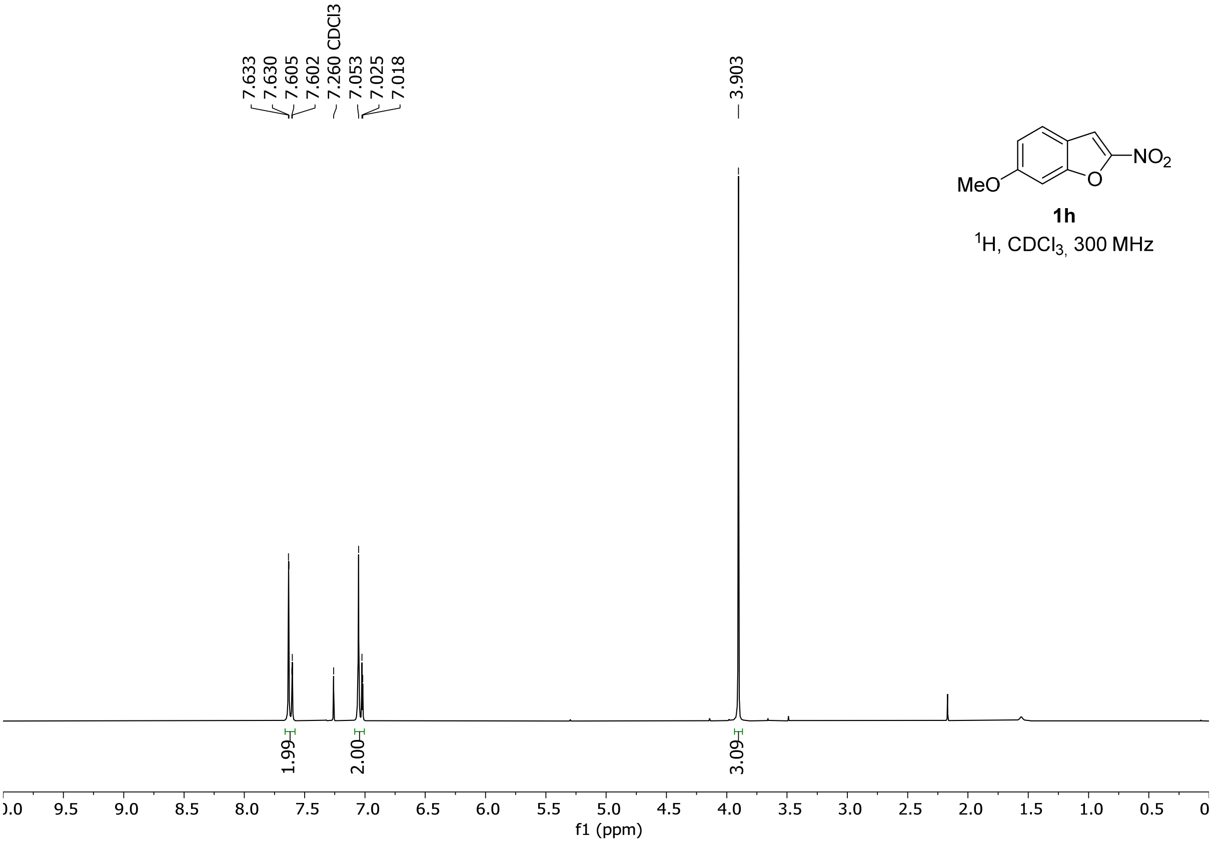

7.633
7.630
7.605
7.602
7.260 CDCl3
7.053
7.025
7.018
3.903
MeO
O
NO2
1h
1H, CDCl3, 300 MHz
1.99
2.00
3.09
f1 (ppm)

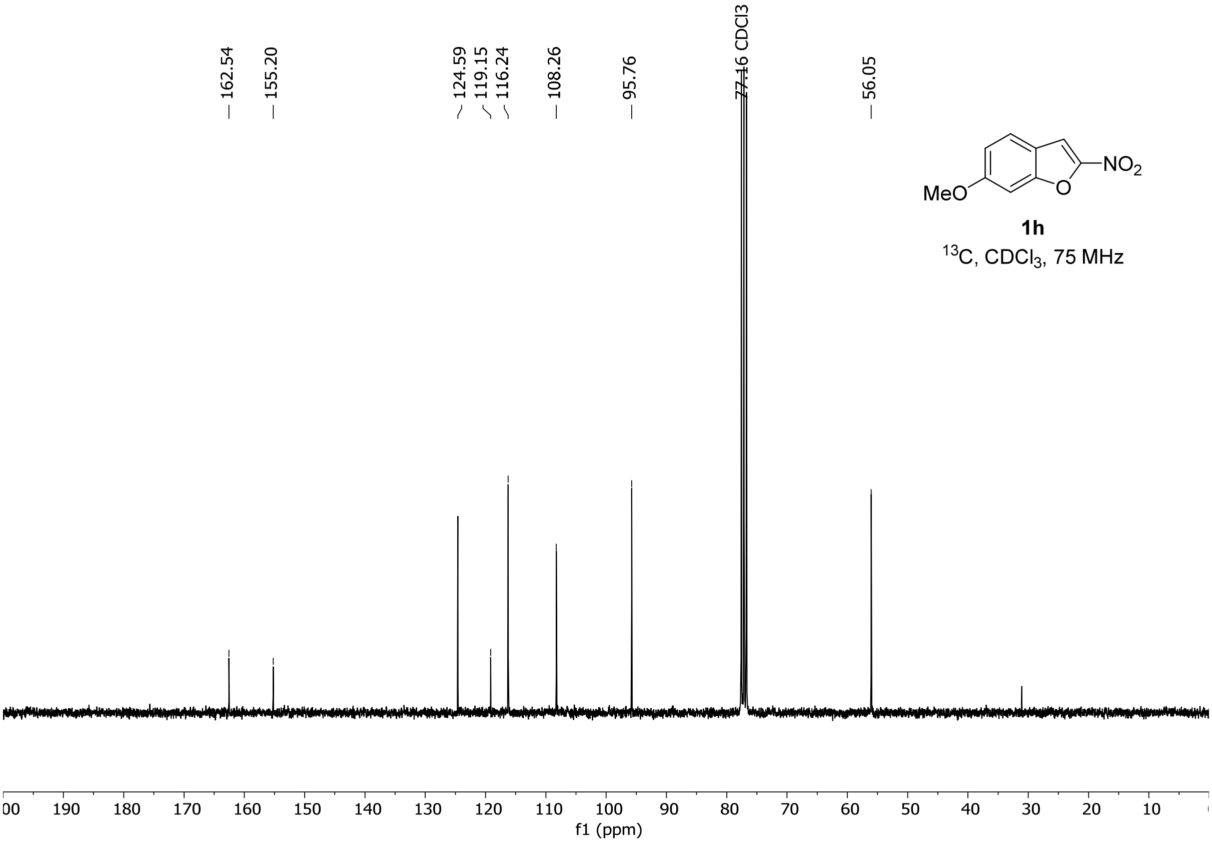

162.54
155.20
124.59
119.15
116.24
108.26
95.76
77.16 CDCl3
56.05
MeO
O
NO2
1h
13C, CDCl3, 75 MHz
f1 (ppm)

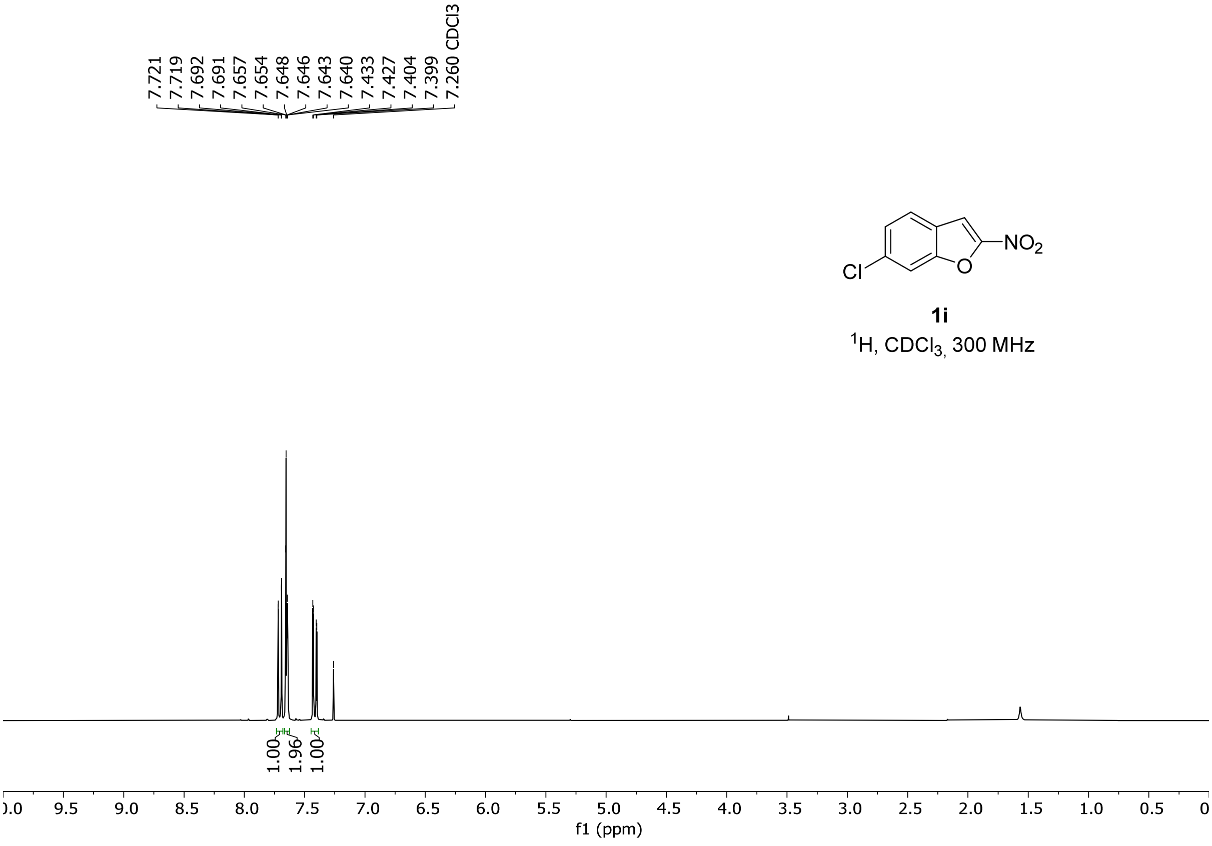

1i

^{1}H, $CDCl_3$, 300 MHz

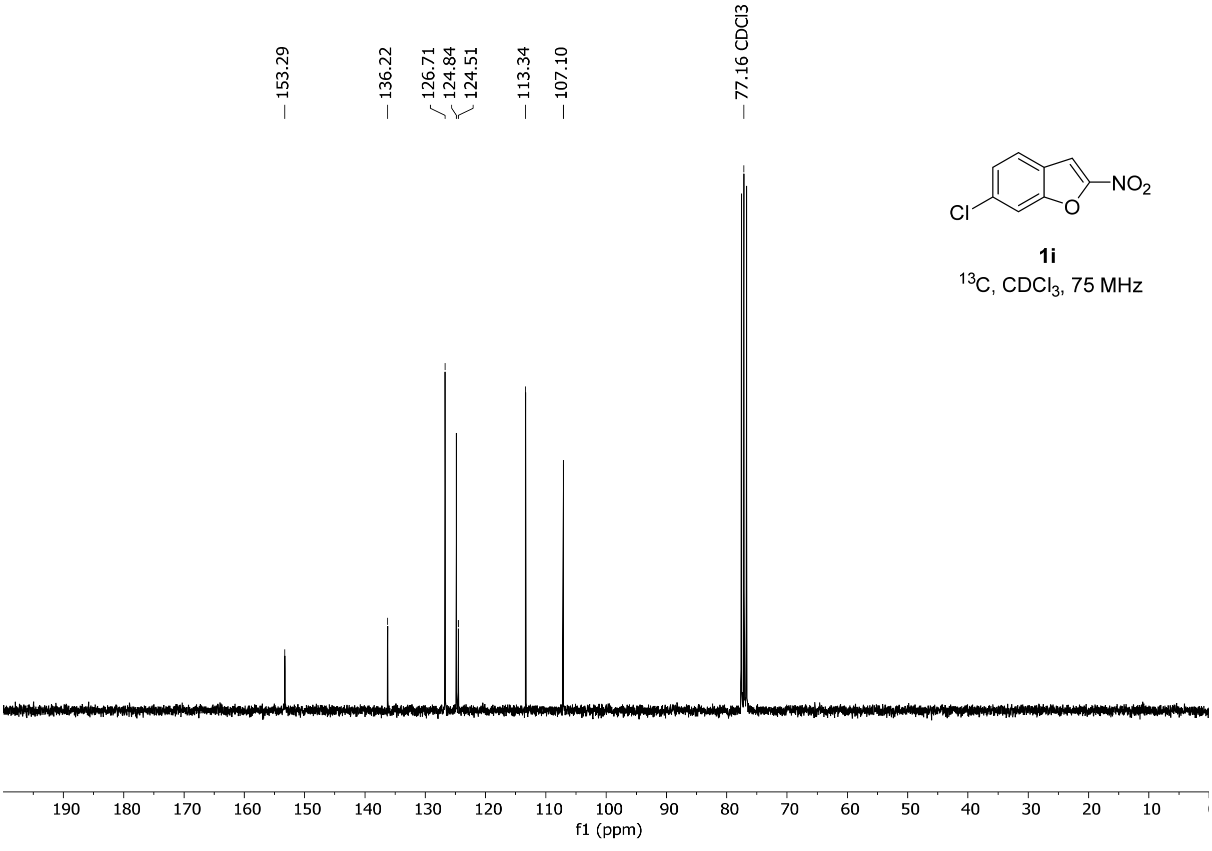

1i

^{13}C, $CDCl_3$, 75 MHz

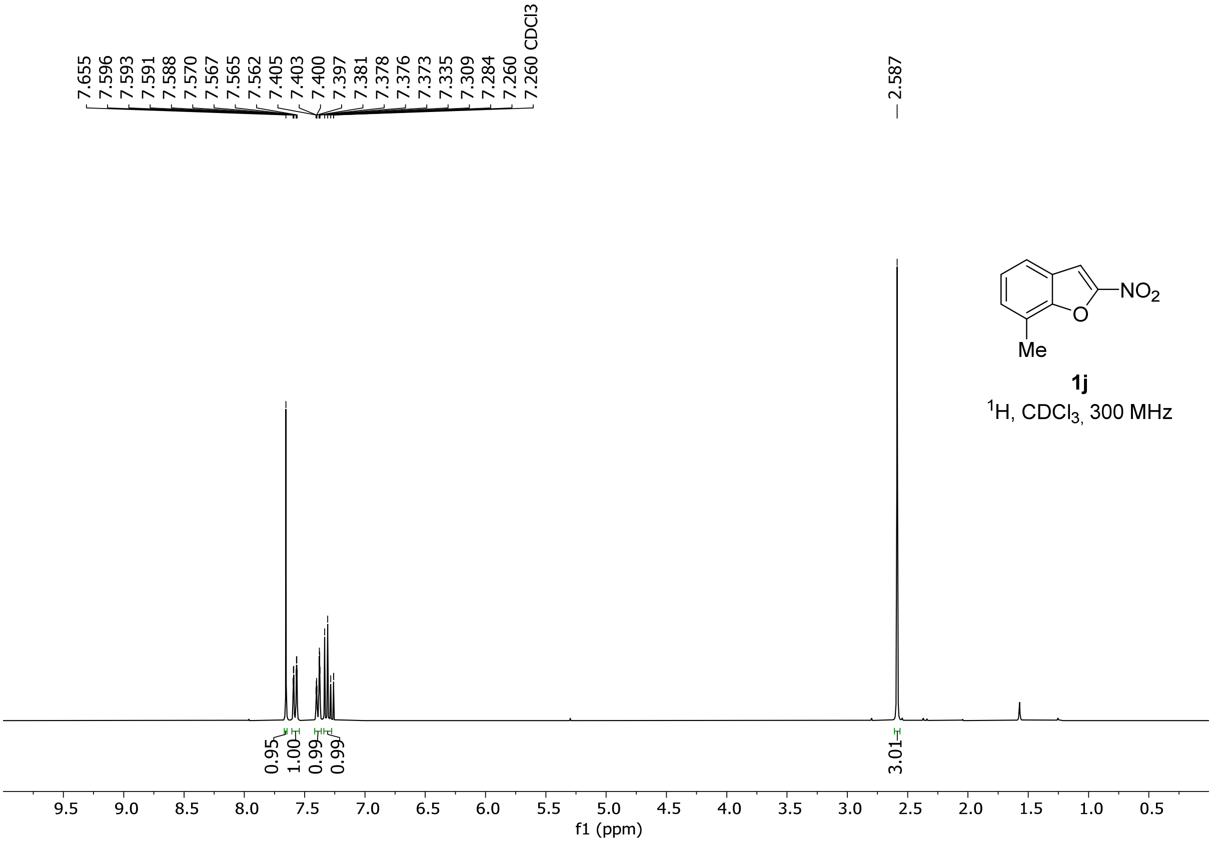

1j

^{1}H, $CDCl_3$, 300 MHz

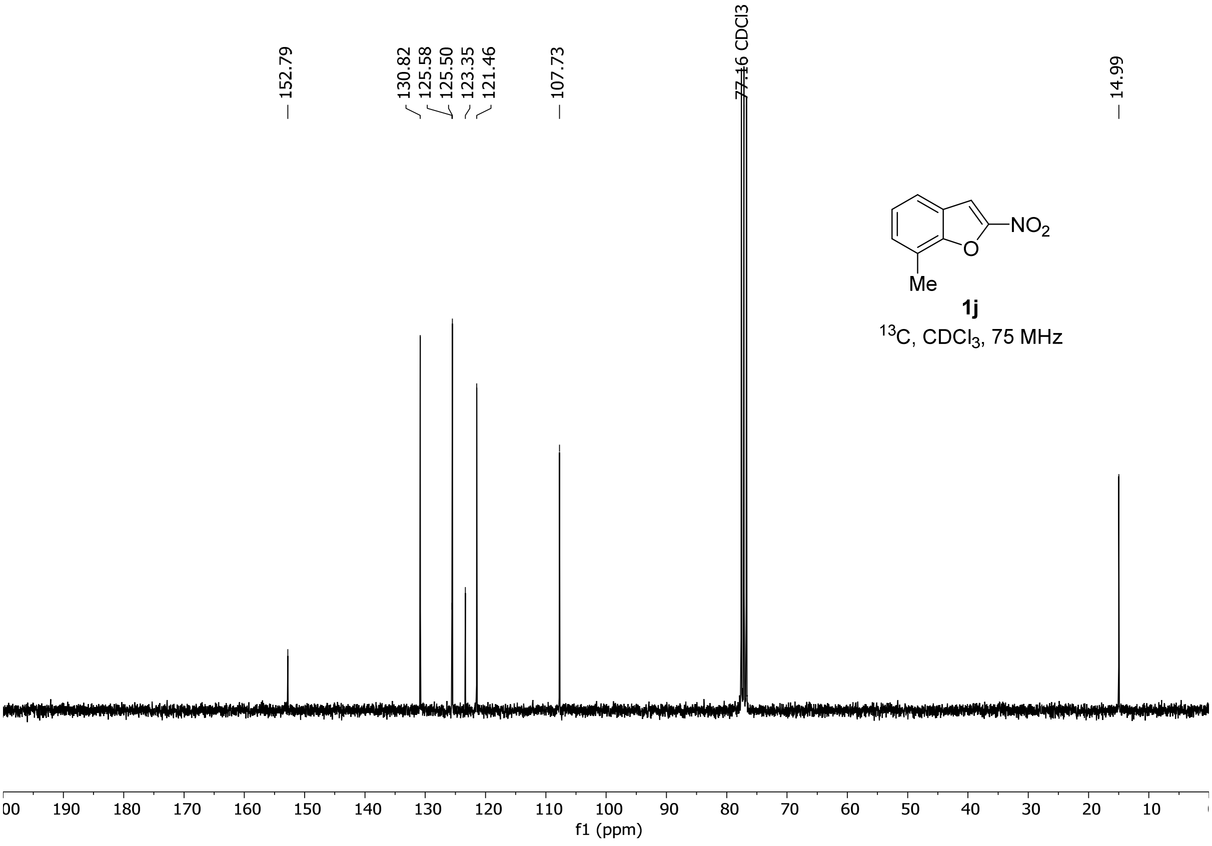

1j

^{13}C, $CDCl_3$, 75 MHz

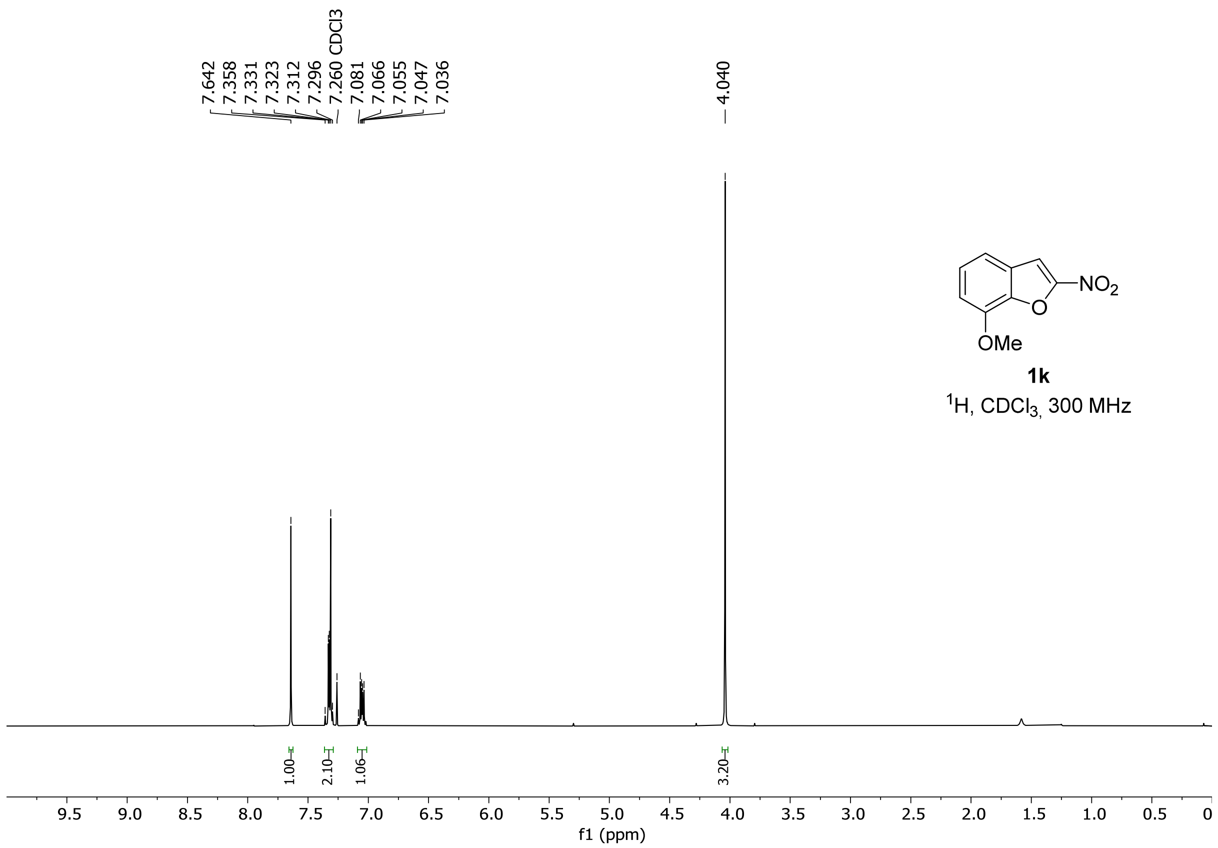
7.642
7.358
7.331
7.323
7.312
7.296
7.260 CDCl3
7.081
7.066
7.055
7.047
7.036
4.040
NO2
O
OMe
1k
1H, CDCl3, 300 MHz
1.00
2.10
1.06
3.20
9.5 9.0 8.5 8.0 7.5 7.0 6.5 6.0 5.5 5.0 4.5 4.0 3.5 3.0 2.5 2.0 1.5 1.0 0.5 0
f1 (ppm)

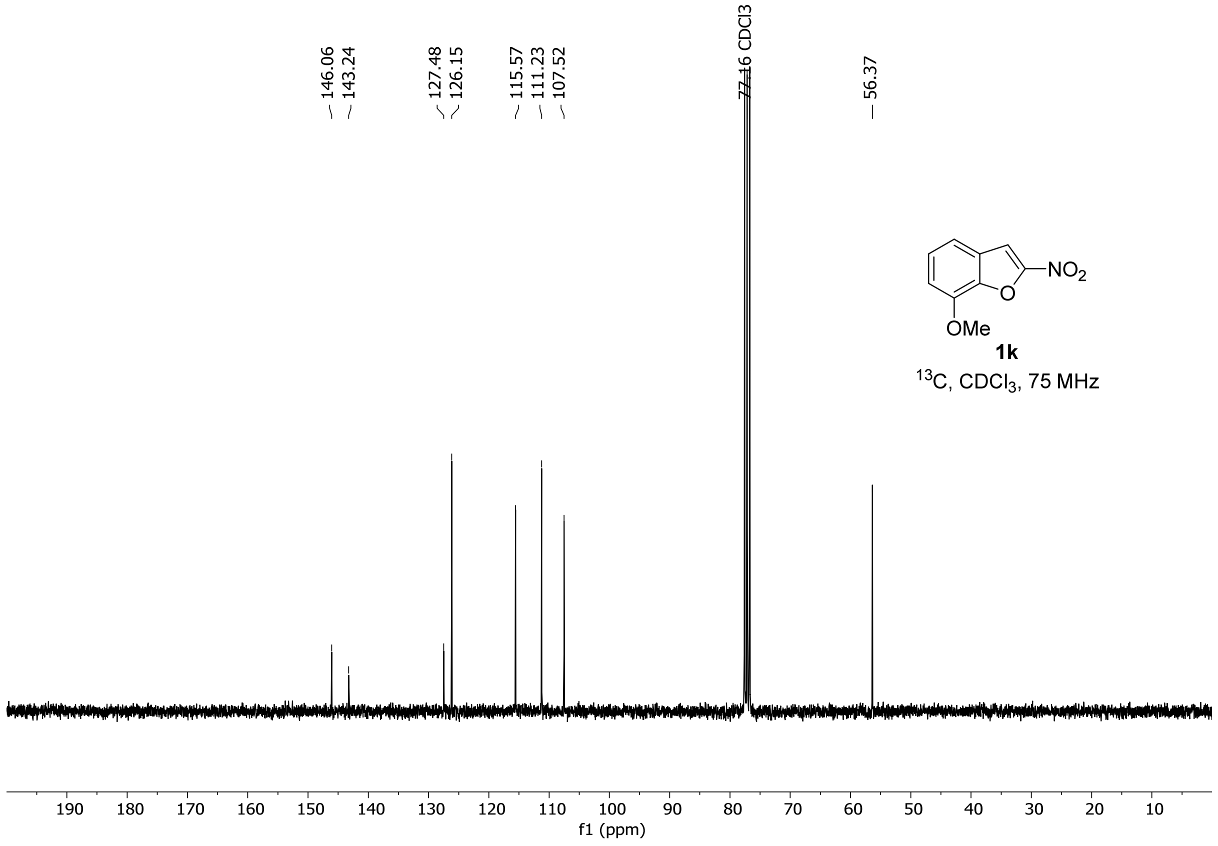
146.06
143.24
127.48
126.15
115.57
111.23
107.52
77.16 CDCl3
56.37
NO2
O
OMe
1k
13C, CDCl3, 75 MHz
190 180 170 160 150 140 130 120 110 100 90 80 70 60 50 40 30 20 10
f1 (ppm)

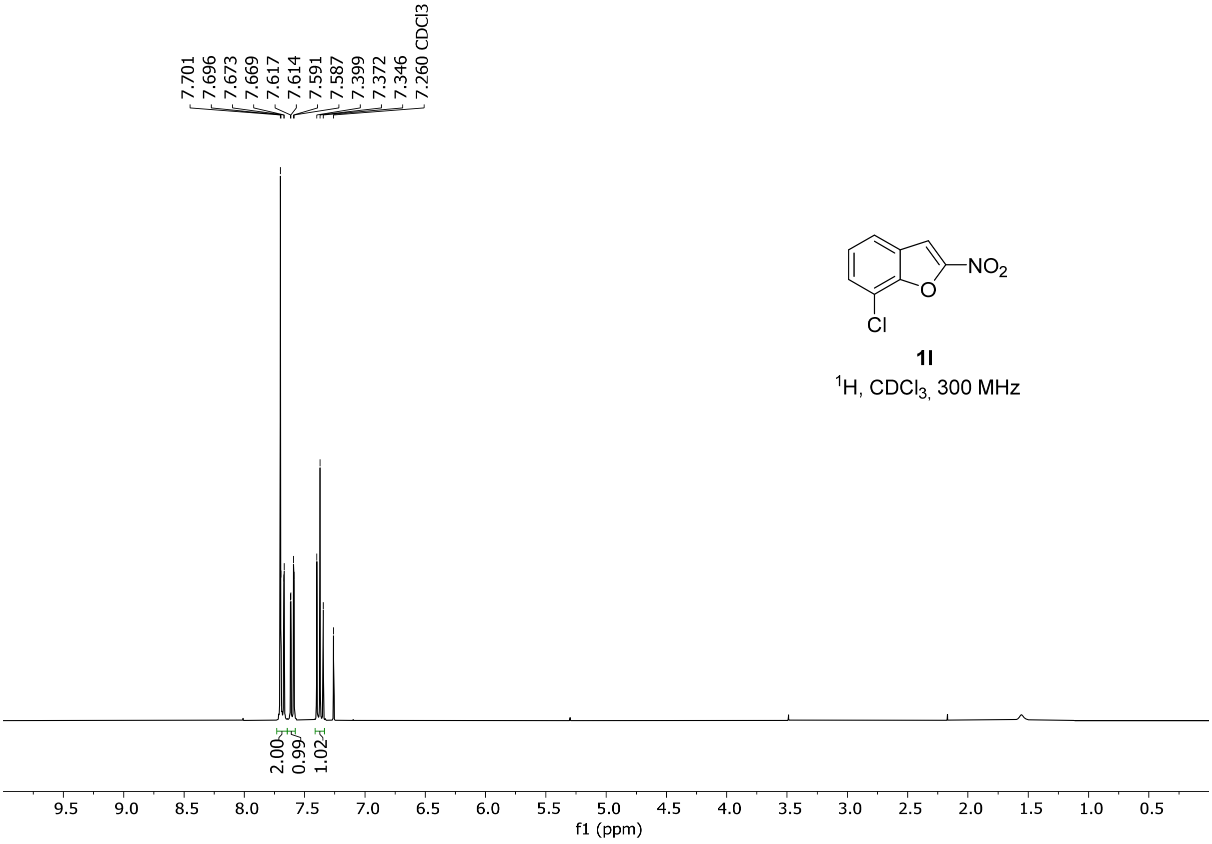

1l

^{1}H, $CDCl_3$, 300 MHz

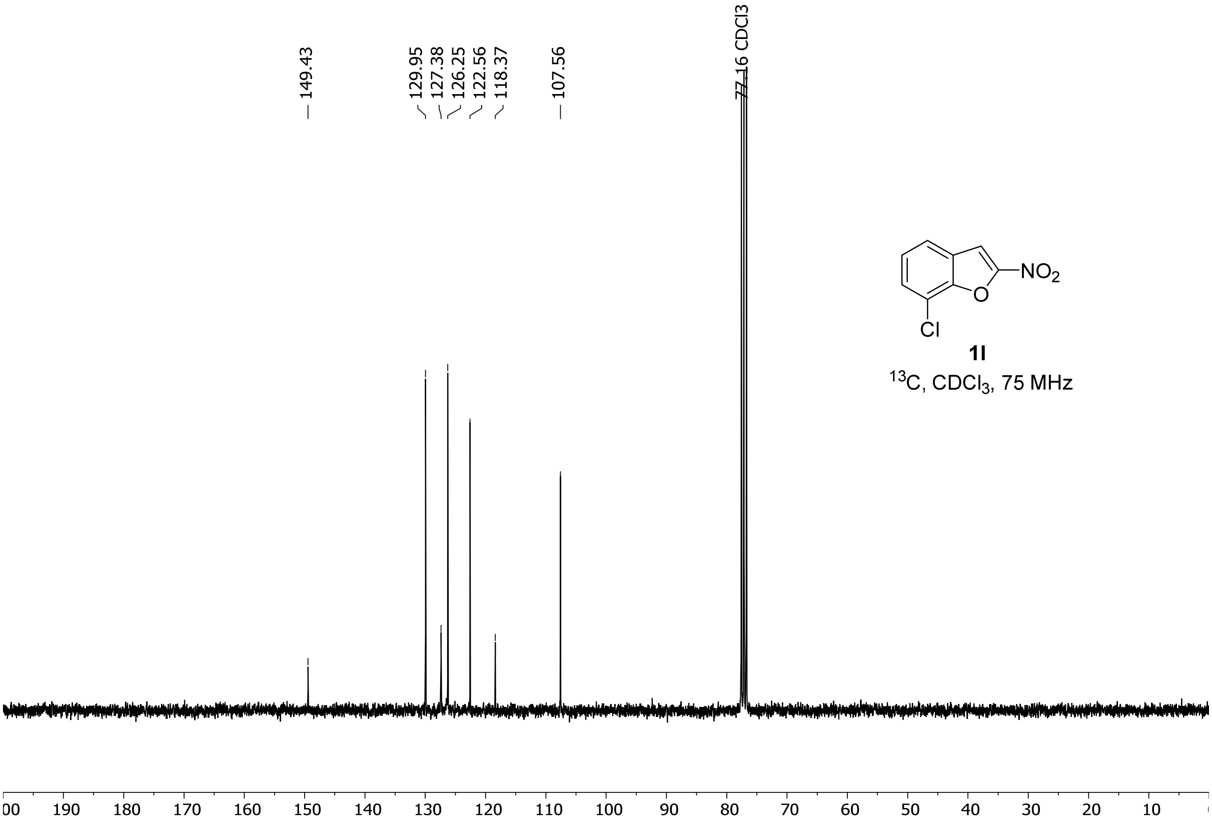

1l

^{13}C, $CDCl_3$, 75 MHz

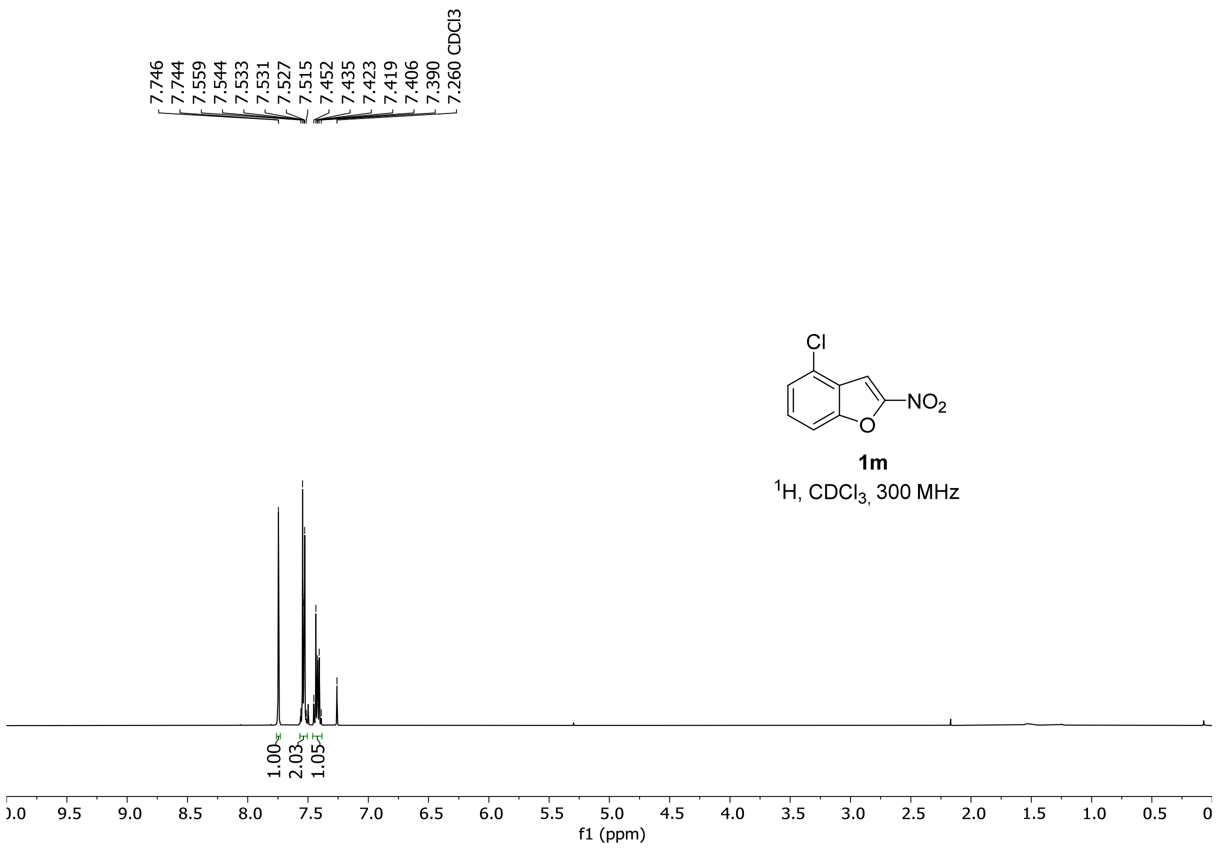
7.746
7.744
7.559
7.544
7.533
7.531
7.527
7.515
7.452
7.435
7.423
7.419
7.406
7.390
7.260 CDCl3
Cl
NO2
O
1m
1H, CDCl3, 300 MHz
1.00
2.03
1.05
f1 (ppm)

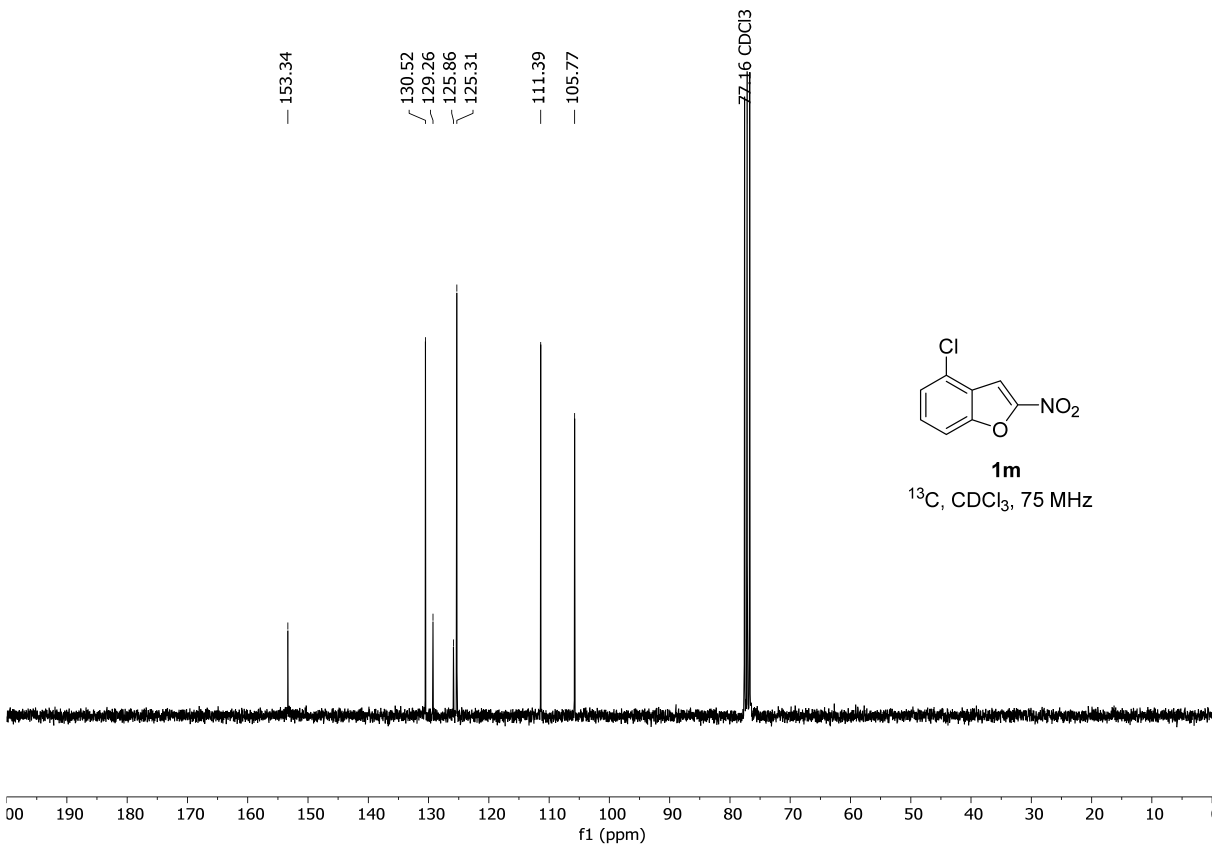
153.34
130.52
129.26
125.86
125.31
111.39
105.77
77.16 CDCl3
Cl
NO2
O
1m
13C, CDCl3, 75 MHz
f1 (ppm)

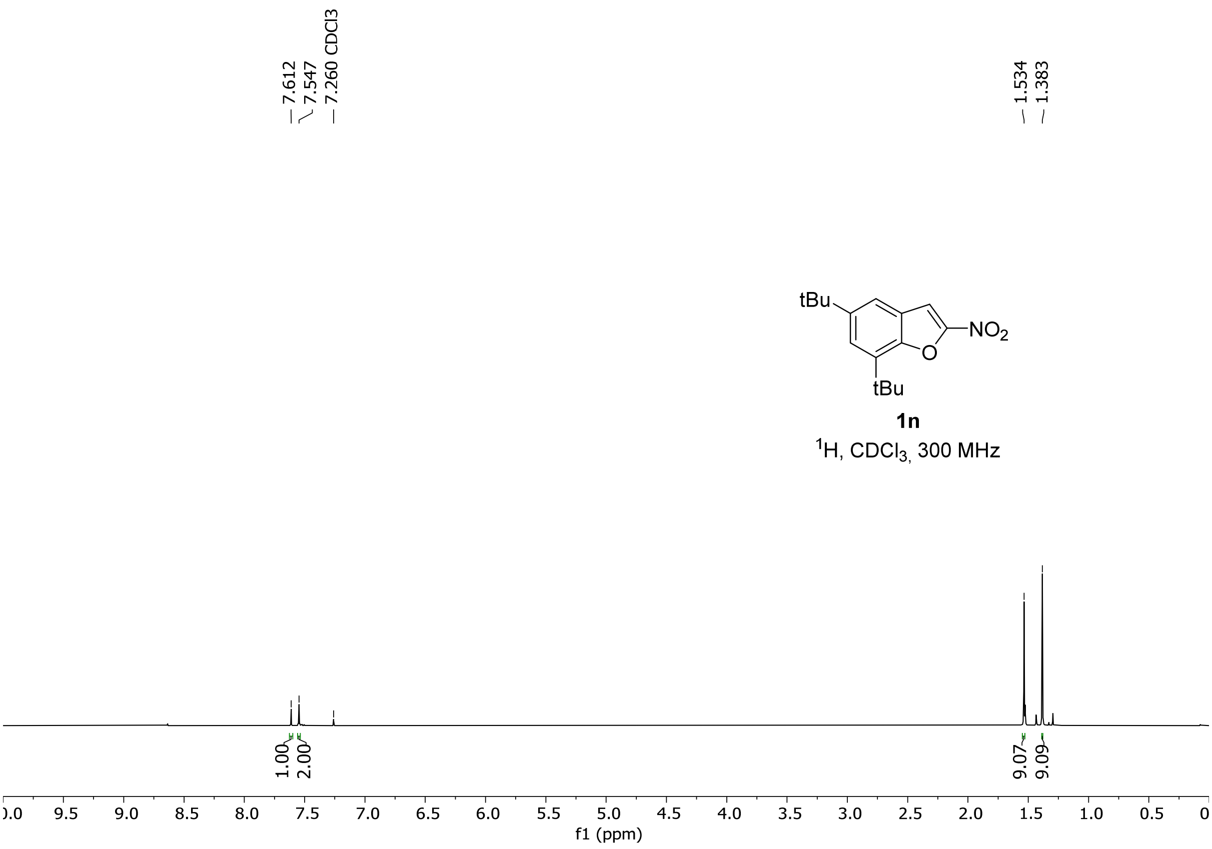
7.612
7.547
7.260 CDCl3
1.534
1.383
tBu
NO2
O
tBu
1n
1H, CDCl3, 300 MHz
1.00
2.00
9.07
9.09
f1 (ppm)

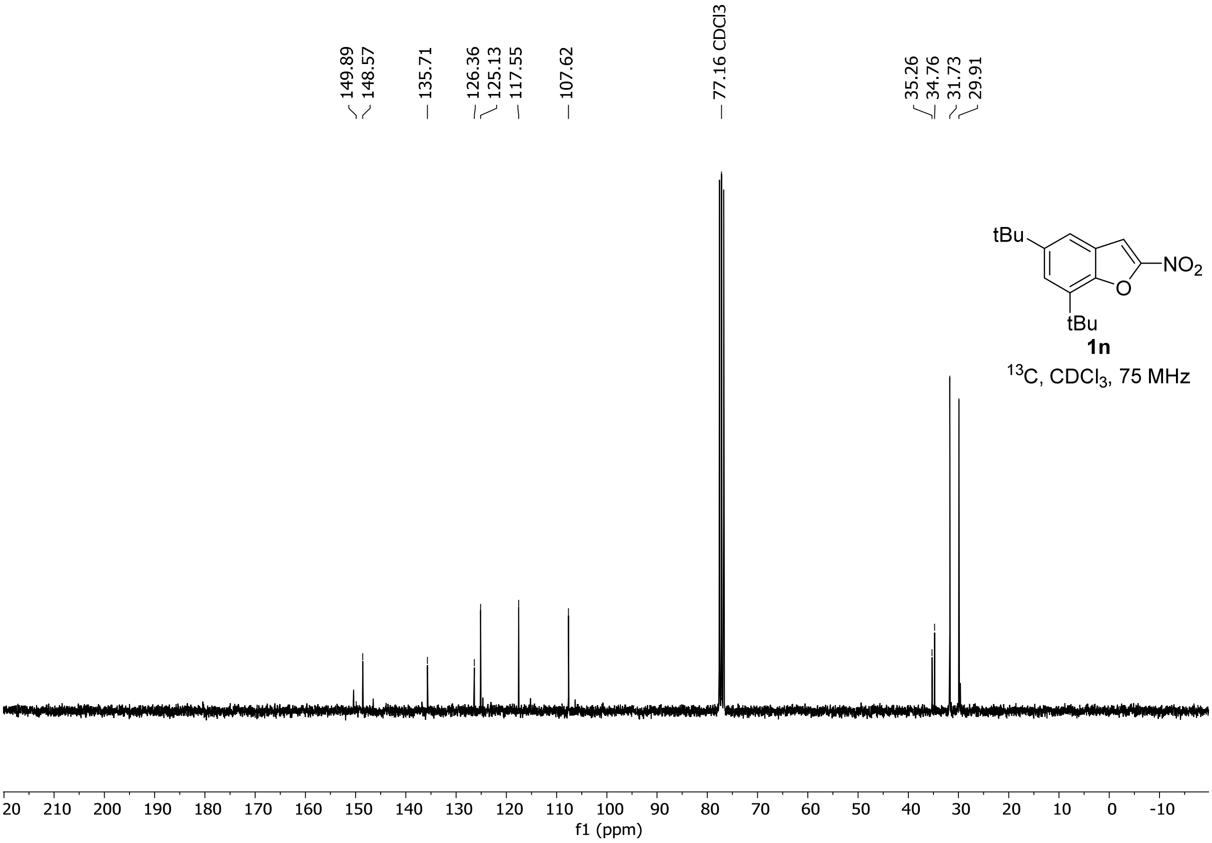
149.89
148.57
135.71
126.36
125.13
117.55
107.62
77.16 CDCl3
35.26
34.76
31.73
29.91
tBu
NO2
O
tBu
1n
13C, CDCl3, 75 MHz
f1 (ppm)

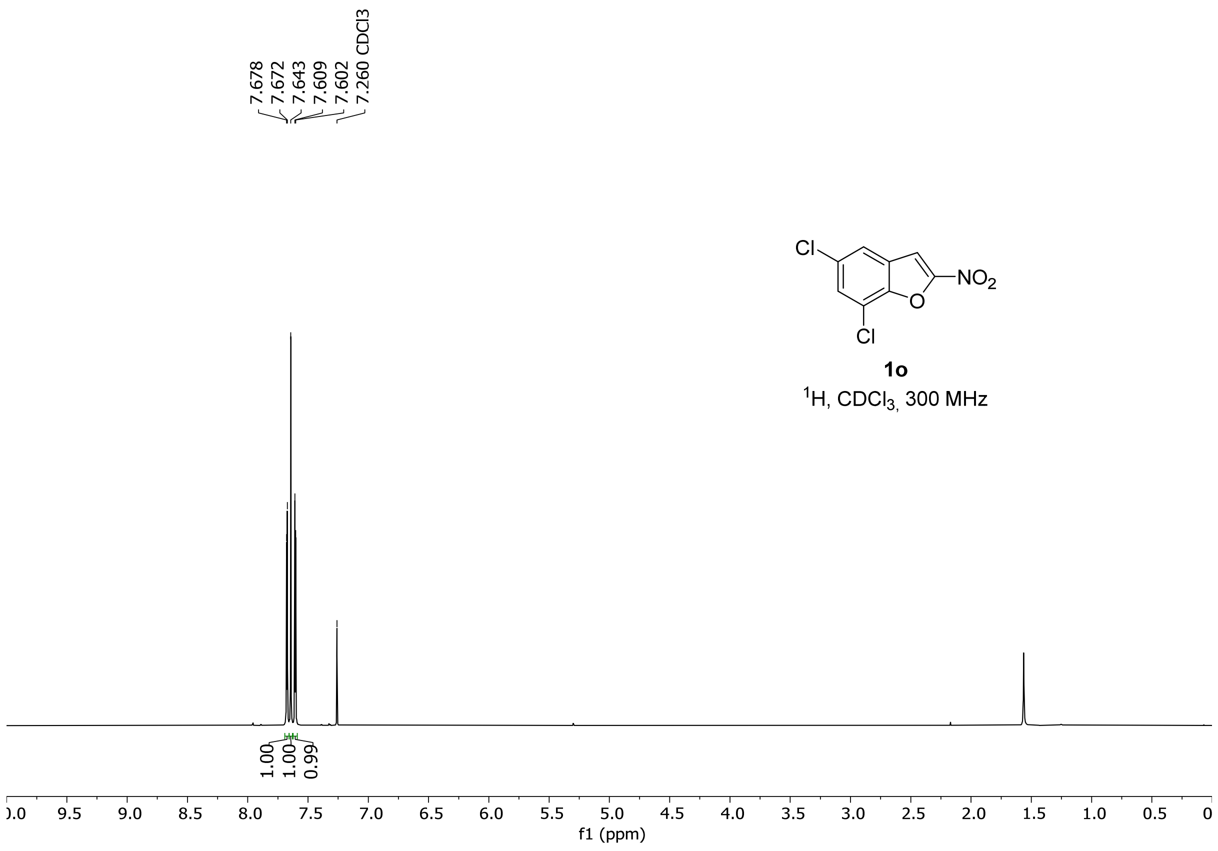
7.678
7.672
7.643
7.609
7.602
7.260 CDCl3
Cl
NO2
O
Cl
1o
1H, CDCl3, 300 MHz
1.00
1.00
0.99
f1 (ppm)

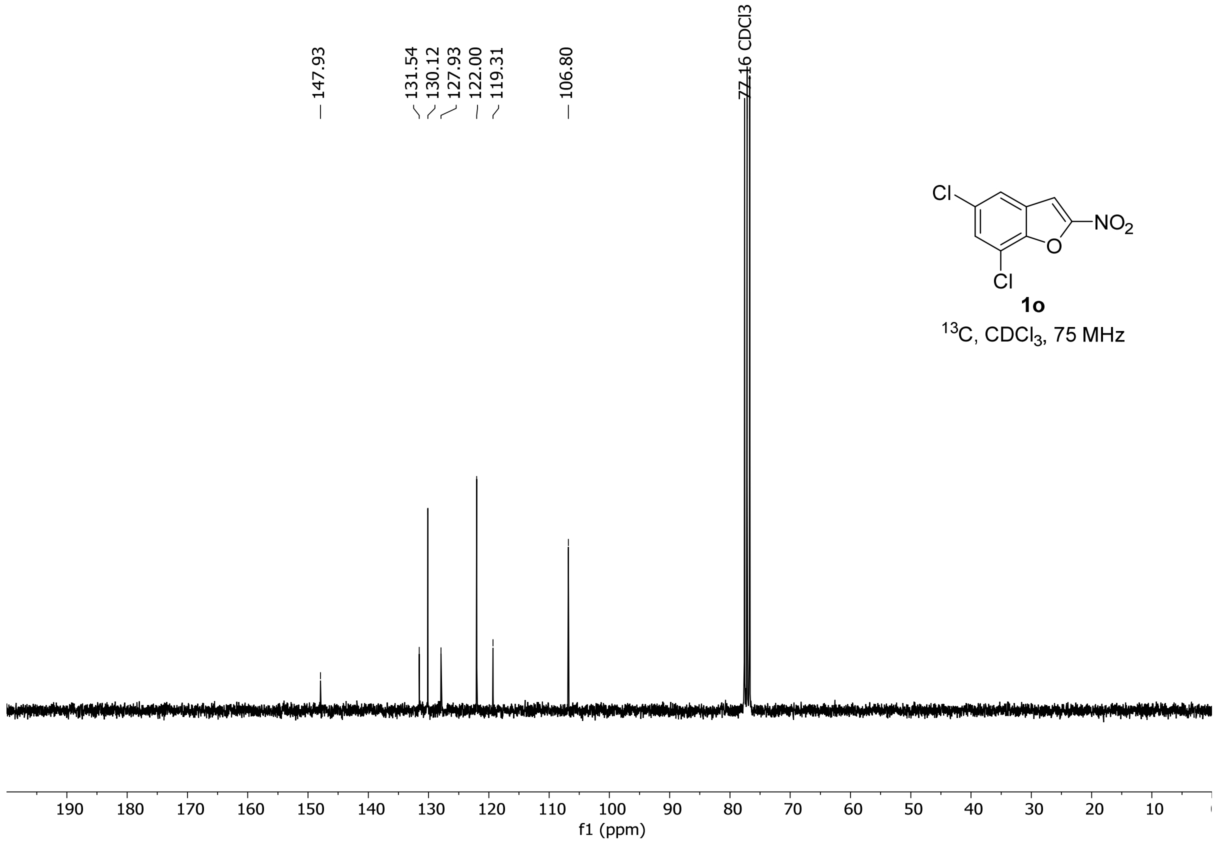
147.93
131.54
130.12
127.93
122.00
119.31
106.80
77.16 CDCl3
Cl
NO2
O
Cl
1o
13C, CDCl3, 75 MHz
f1 (ppm)

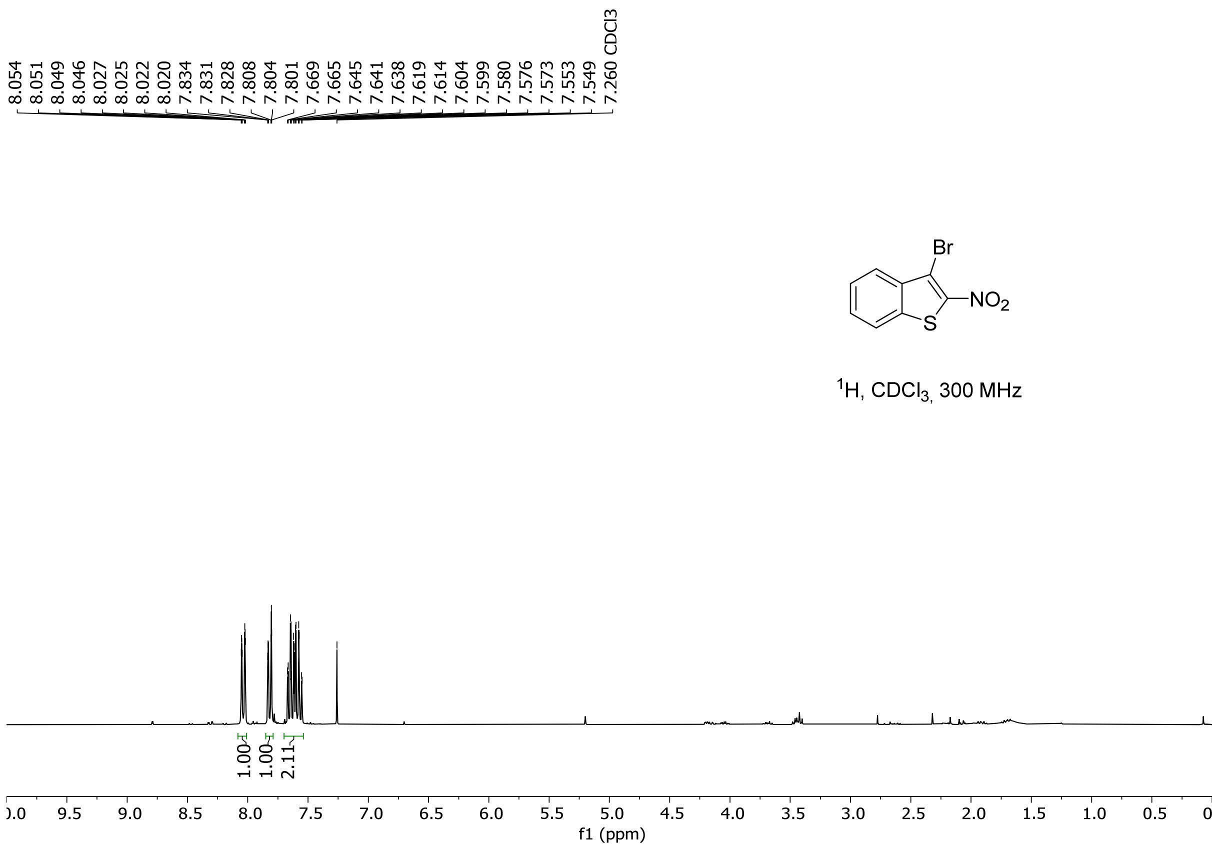
8.054
8.051
8.049
8.046
8.027
8.025
8.022
8.020
7.834
7.831
7.828
7.808
7.804
7.801
7.669
7.665
7.645
7.641
7.638
7.619
7.614
7.604
7.599
7.580
7.576
7.573
7.553
7.549
7.260 CDCl3
Br
NO2
S
1H, CDCl3, 300 MHz
1.00
1.00
2.11
9.5 9.0 8.5 8.0 7.5 7.0 6.5 6.0 5.5 5.0 4.5 4.0 3.5 3.0 2.5 2.0 1.5 1.0 0.5 0
f1 (ppm)

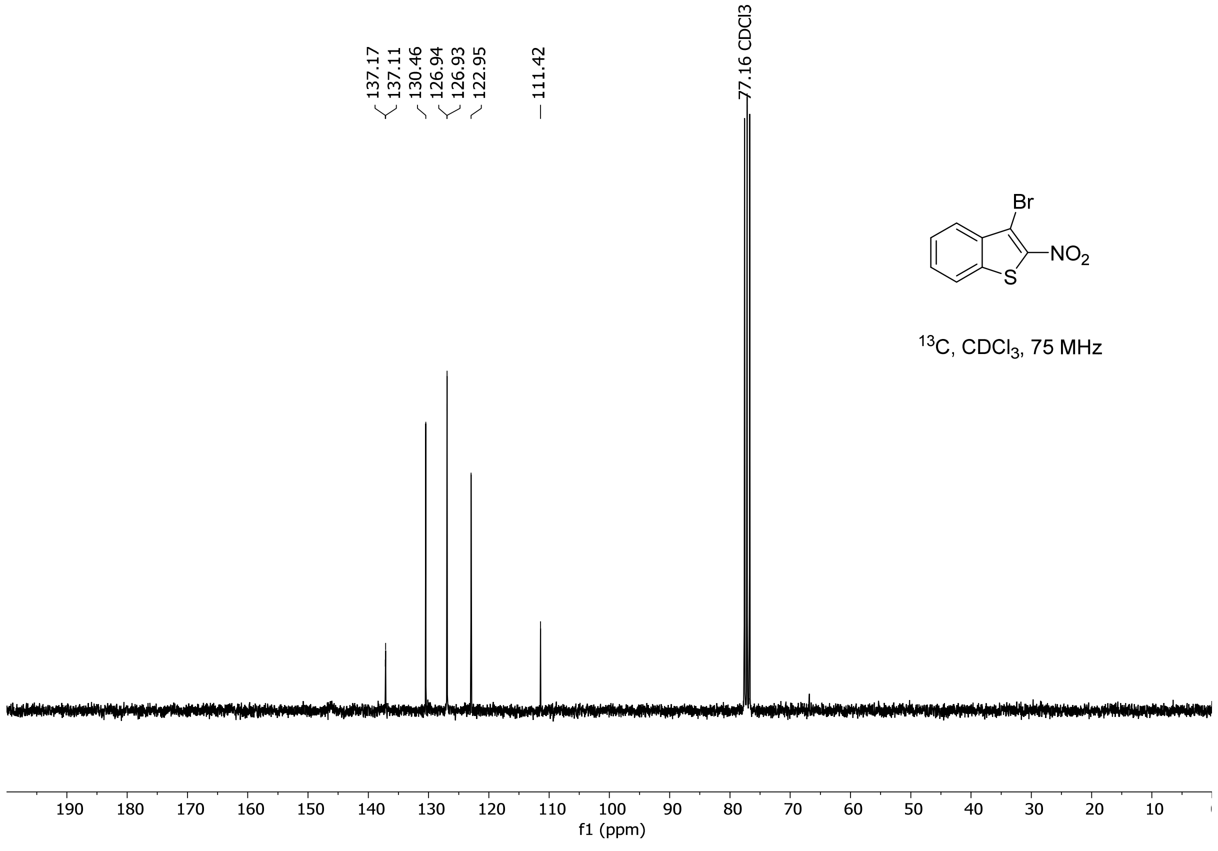
137.17
137.11
130.46
126.94
126.93
122.95
111.42
77.16 CDCl3
Br
NO2
S
13C, CDCl3, 75 MHz
190 180 170 160 150 140 130 120 110 100 90 80 70 60 50 40 30 20 10
f1 (ppm)

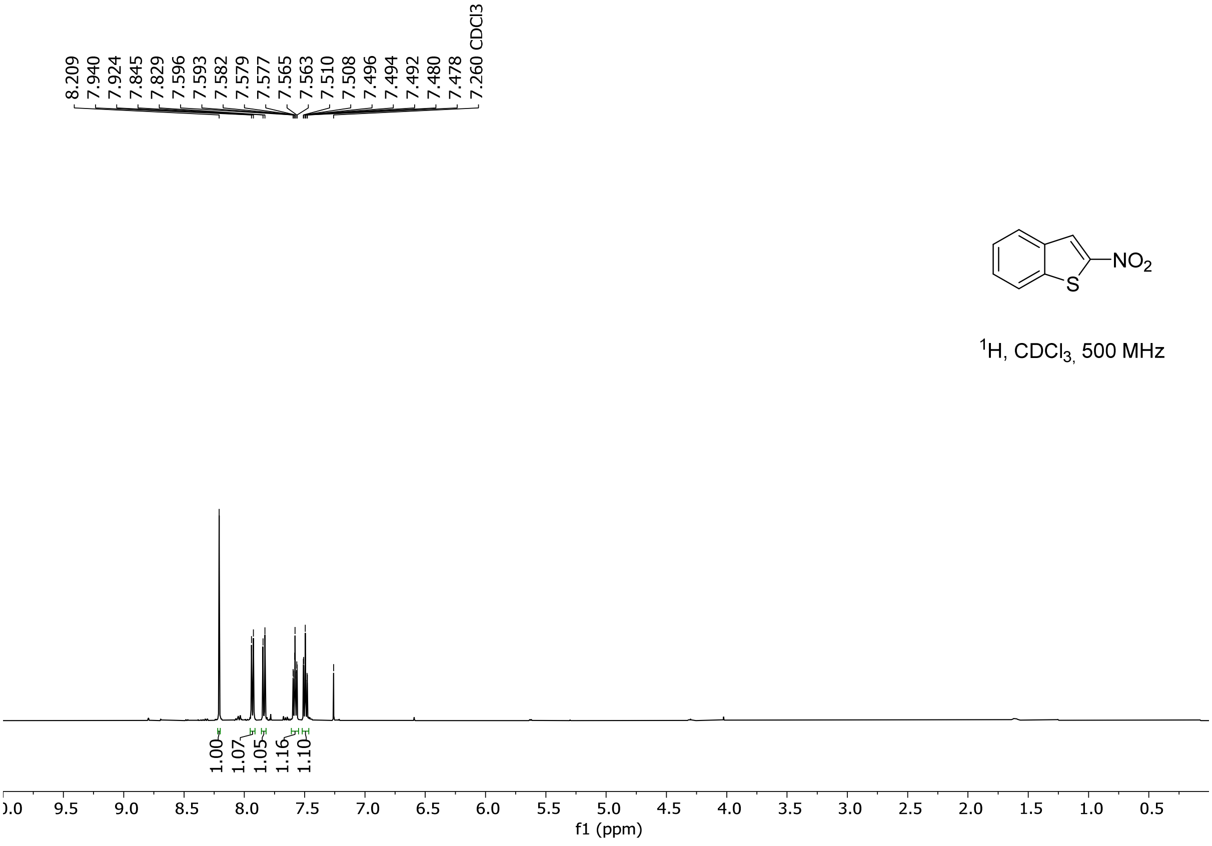

8.209
7.940
7.924
7.845
7.829
7.596
7.593
7.582
7.579
7.577
7.565
7.563
7.510
7.508
7.496
7.494
7.492
7.480
7.478
7.260 CDCl3
NO2
S
1H, CDCl3, 500 MHz
1.00
1.07
1.05
1.16
1.10
f1 (ppm)

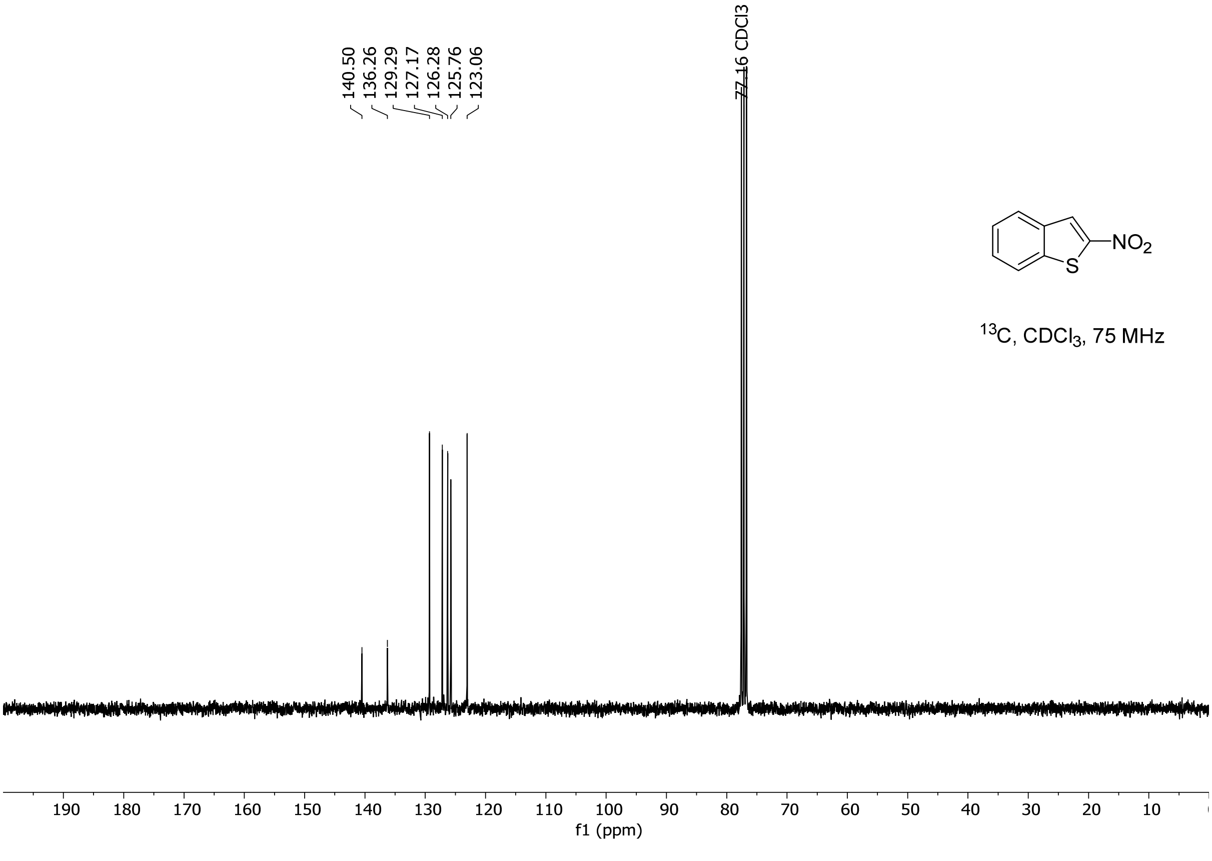

140.50
136.26
129.29
127.17
126.28
125.76
123.06
77.16 CDCl3
NO2
S
13C, CDCl3, 75 MHz
f1 (ppm)

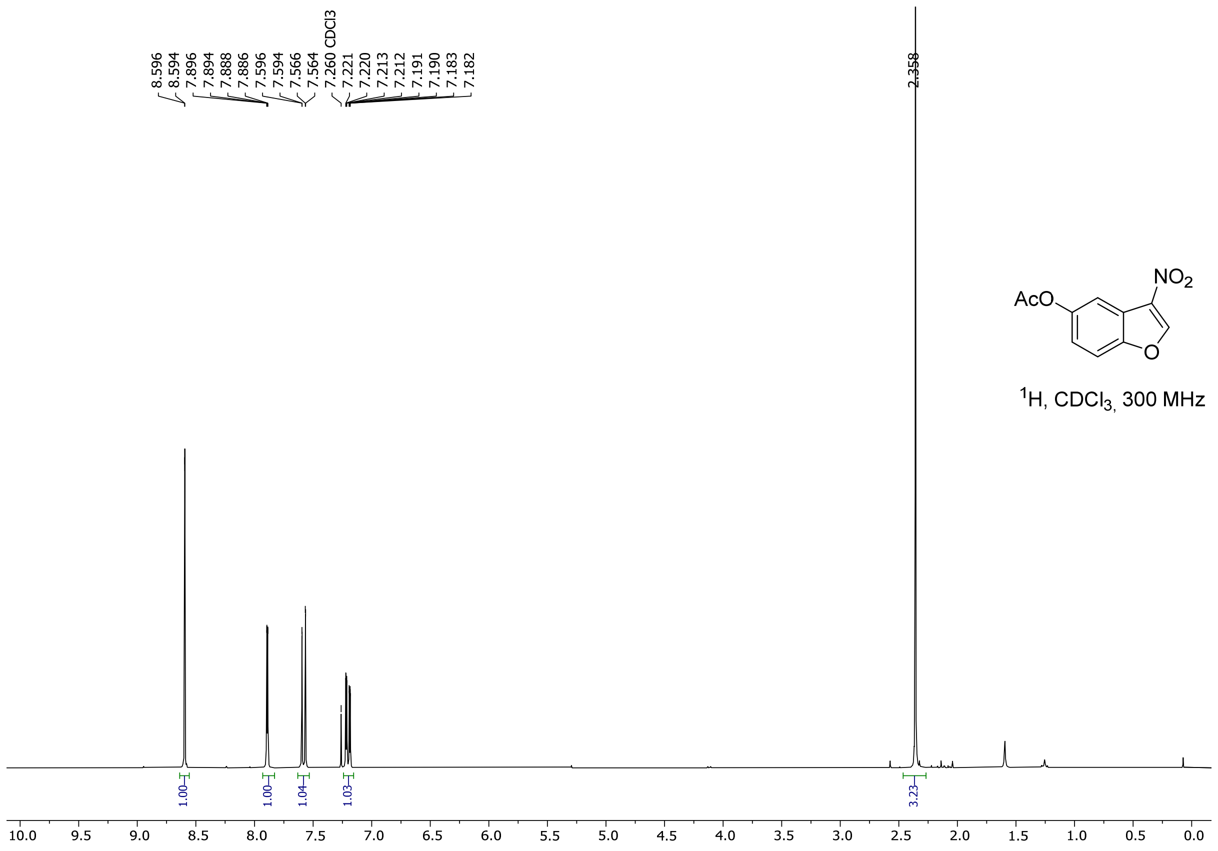
8.596
8.594
7.896
7.894
7.888
7.886
7.596
7.594
7.566
7.564
7.260 CDCl3
7.221
7.220
7.213
7.212
7.191
7.190
7.183
7.182
2.358
AcO
NO2
O
1H, CDCl3, 300 MHz
1.00
1.00
1.04
1.03
3.23
10.0
9.5
9.0
8.5
8.0
7.5
7.0
6.5
6.0
5.5
5.0
4.5
4.0
3.5
3.0
2.5
2.0
1.5
1.0
0.5
0.0

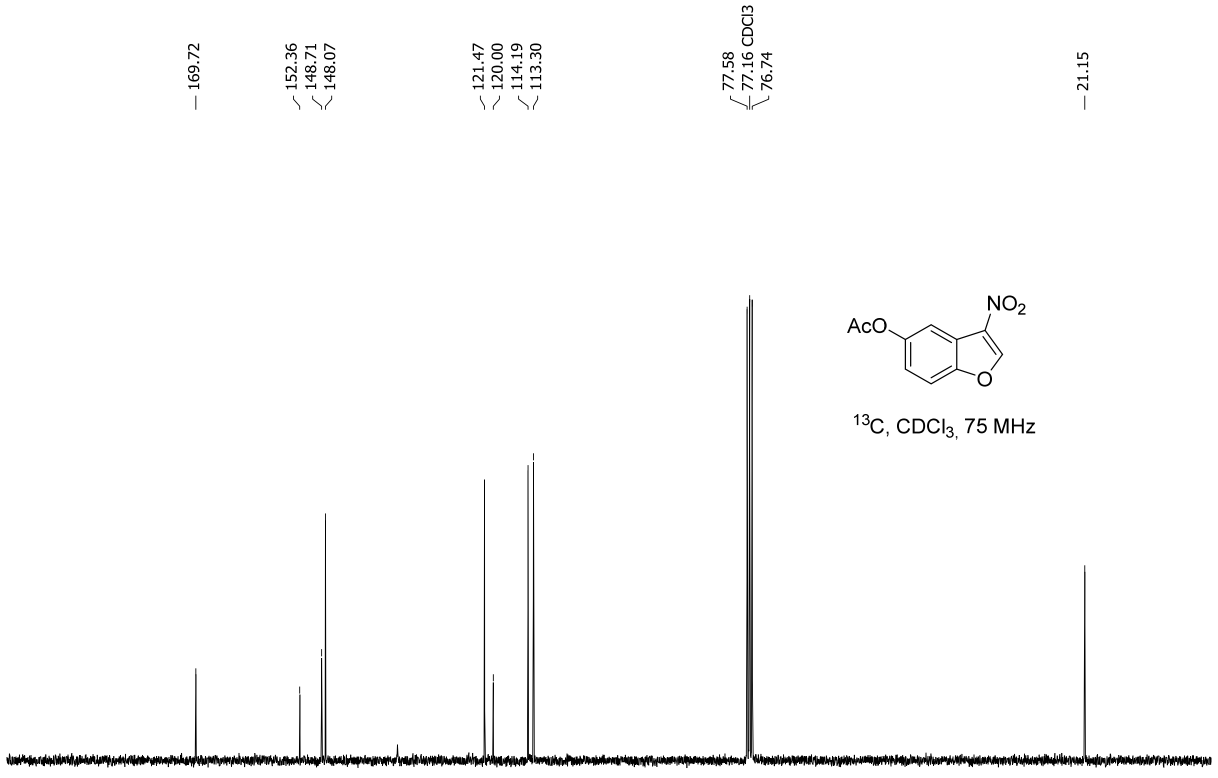
169.72
152.36
148.71
148.07
121.47
120.00
114.19
113.30
77.58
77.16 CDCl3
76.74
21.15
AcO
NO2
O
13C, CDCl3, 75 MHz
200
190
180
170
160
150
140
130
120
110
100
90
80
70
60
50
40
30
20
10

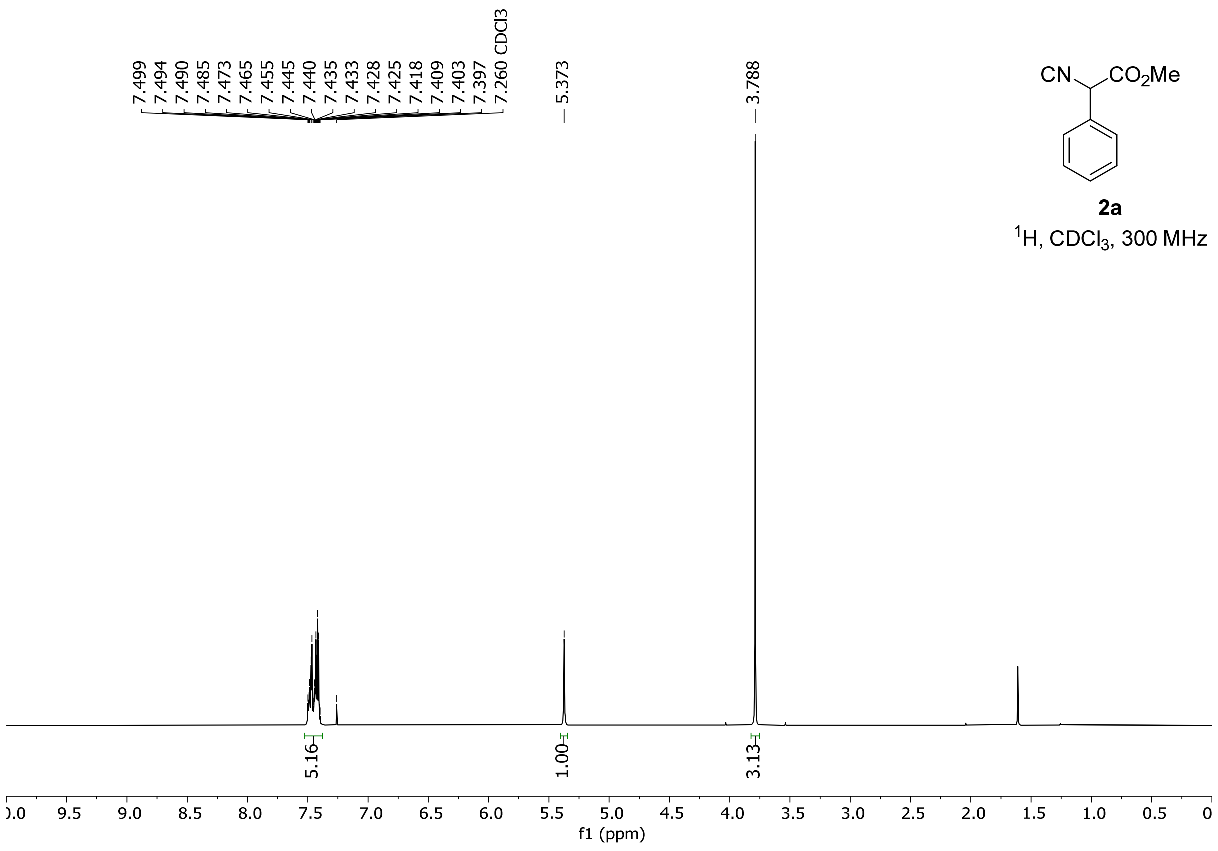
7.499
7.494
7.490
7.485
7.473
7.465
7.455
7.445
7.440
7.435
7.433
7.428
7.425
7.418
7.409
7.403
7.397
7.260 CDCl3
5.373
3.788
CN
CO2Me
2a
1H, CDCl3, 300 MHz
5.16
1.00
3.13
f1 (ppm)

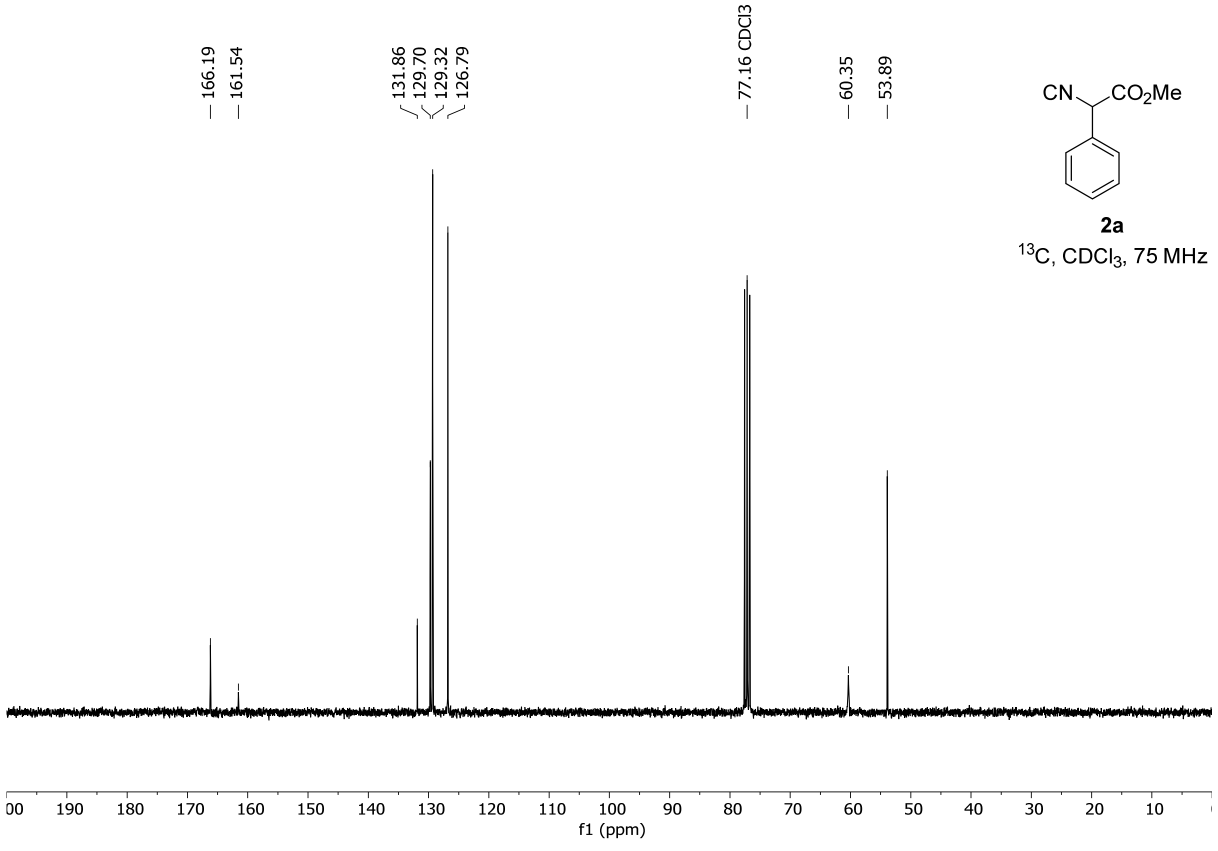
166.19
161.54
131.86
129.70
129.32
126.79
77.16 CDCl3
60.35
53.89
CN
CO2Me
2a
13C, CDCl3, 75 MHz
f1 (ppm)

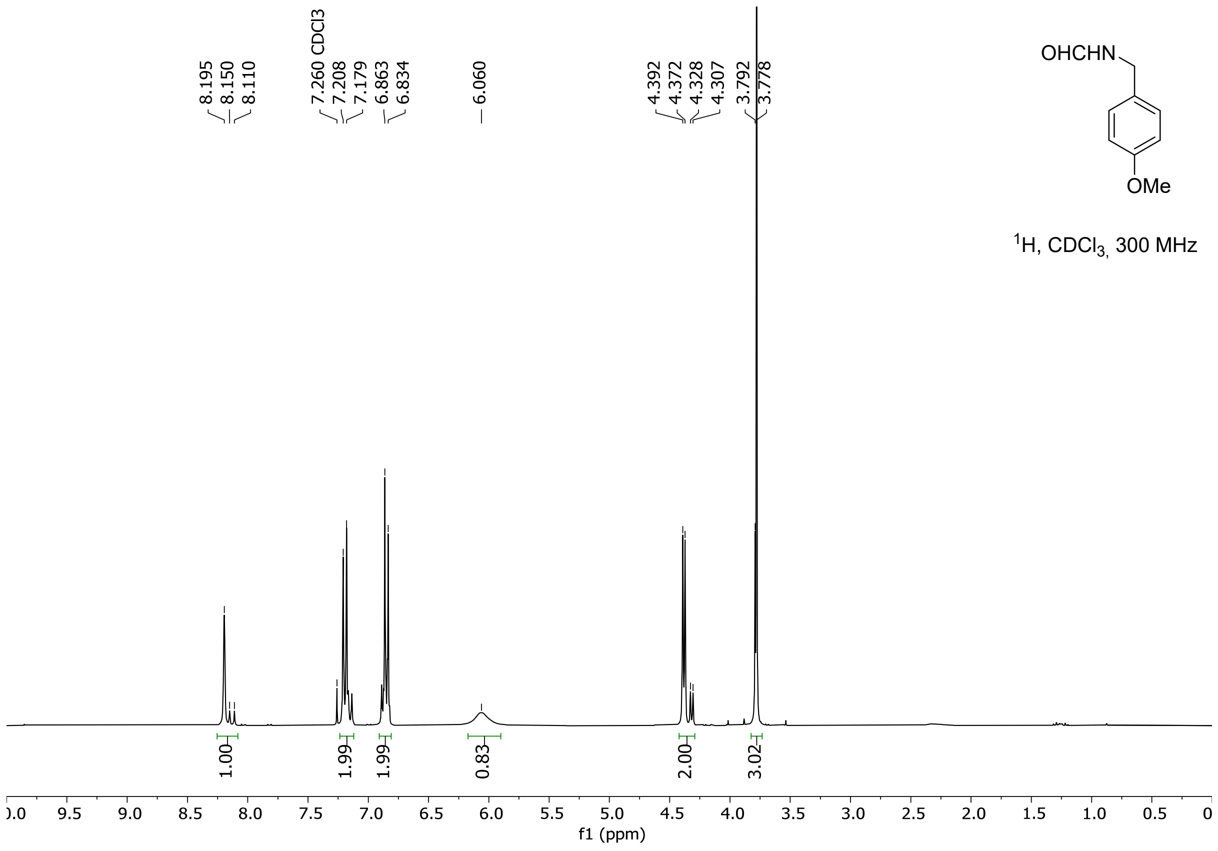

8.195
8.150
8.110
7.260 CDCl3
7.208
7.179
6.863
6.834
6.060
4.392
4.372
4.328
4.307
3.792
3.778
OHCHN
OMe
1H, CDCl3, 300 MHz
1.00
1.99
1.99
0.83
2.00
3.02
f1 (ppm)

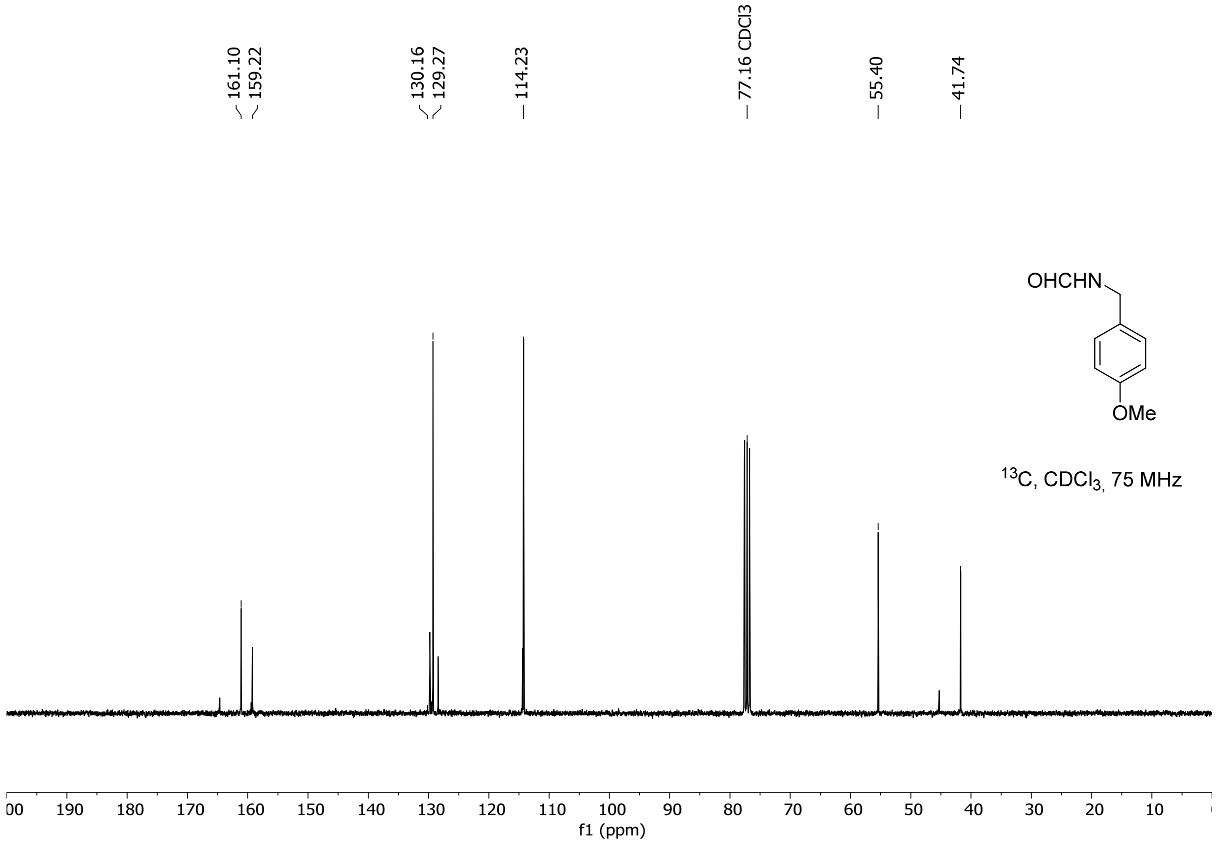

161.10
159.22
130.16
129.27
114.23
77.16 CDCl3
55.40
41.74
OHCHN
OMe
13C, CDCl3, 75 MHz
f1 (ppm)

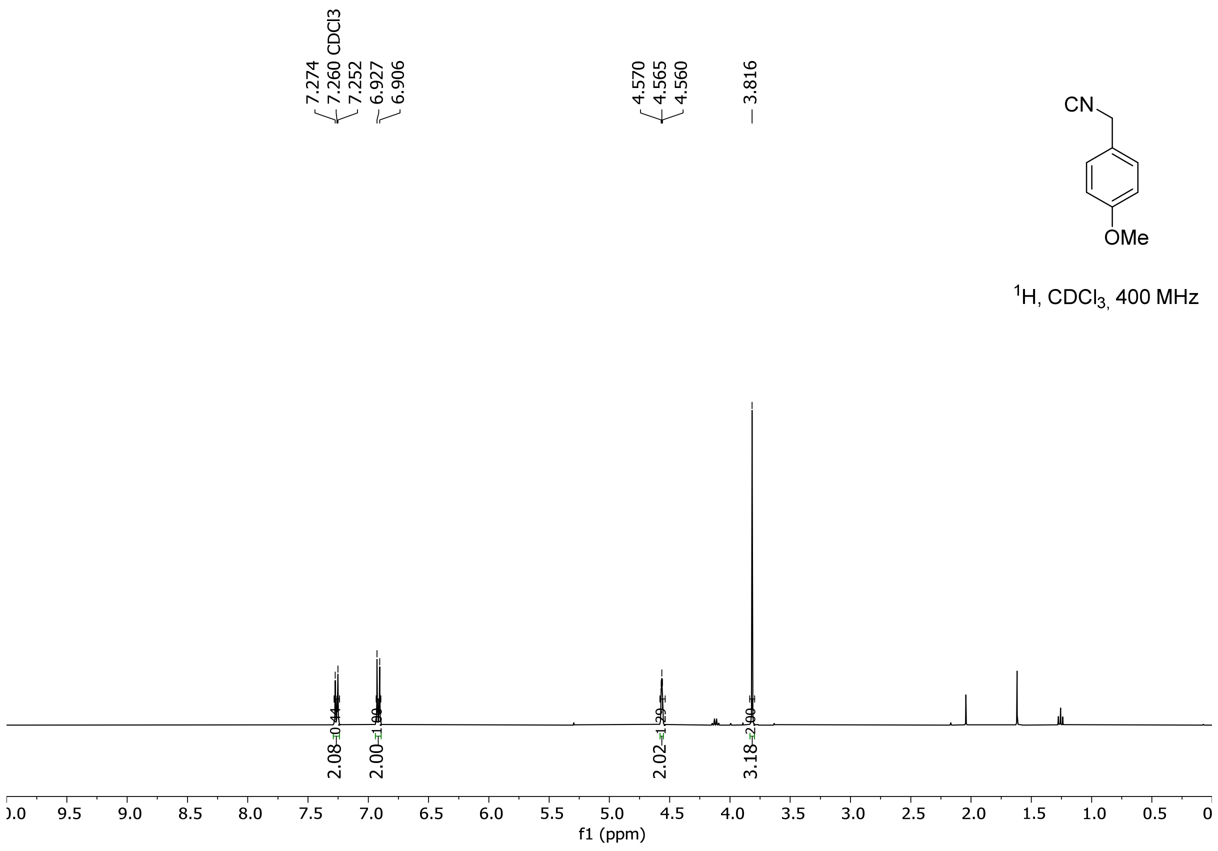
7.274
7.260 CDCl3
7.252
6.927
6.906
4.570
4.565
4.560
3.816
CN
OMe
^{1}H, $CDCl_3$, 400 MHz
2.08
2.00
2.02
3.18
f1 (ppm)

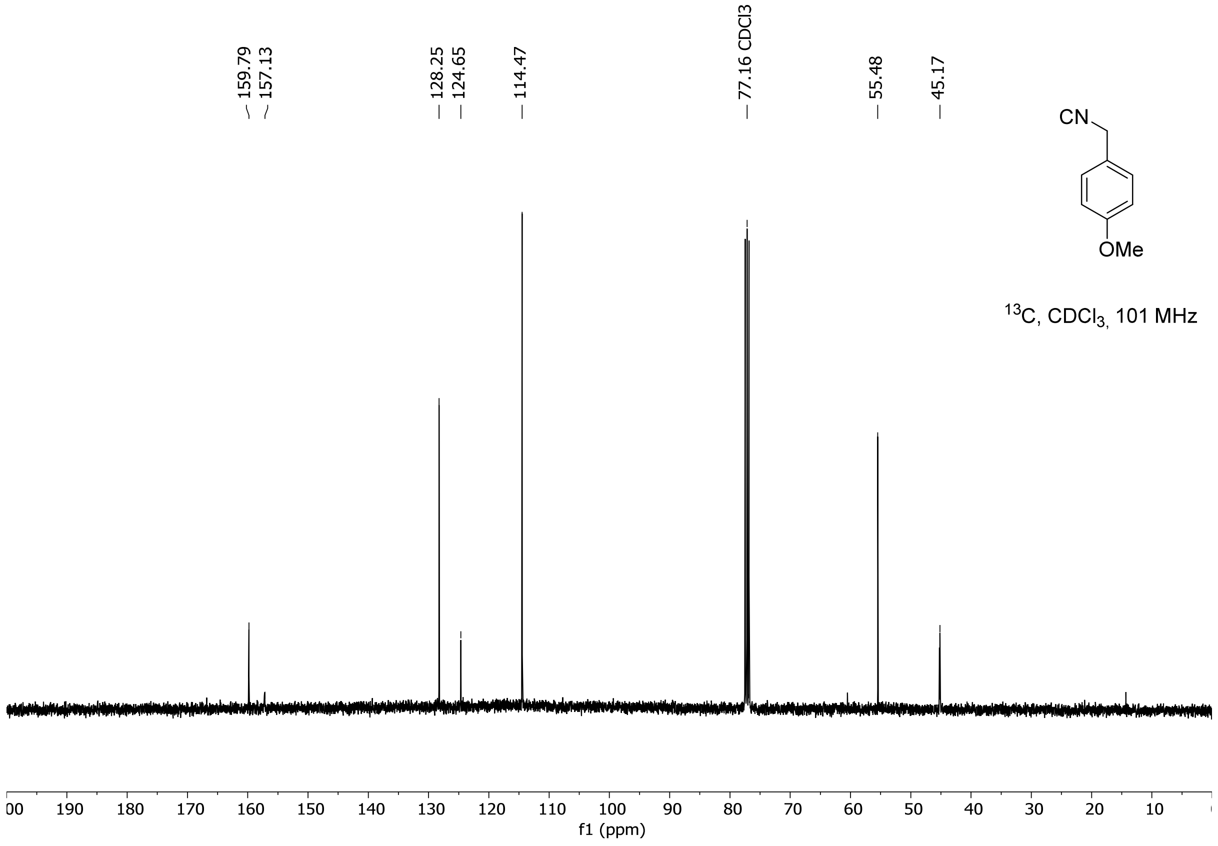
159.79
157.13
128.25
124.65
114.47
77.16 CDCl3
55.48
45.17
CN
OMe
^{13}C, $CDCl_3$, 101 MHz
f1 (ppm)

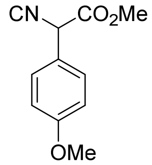

2b

^{1}H, $CDCl_3$, 300 MHz

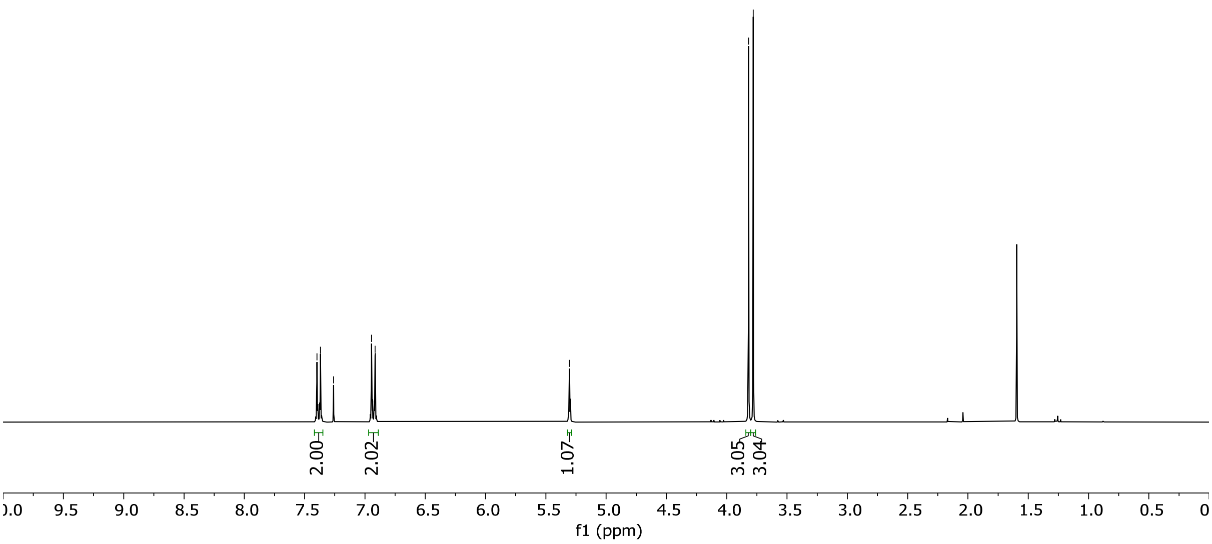

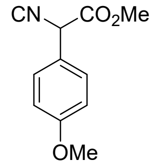

2b

^{13}C, $CDCl_3$, 75 MHz

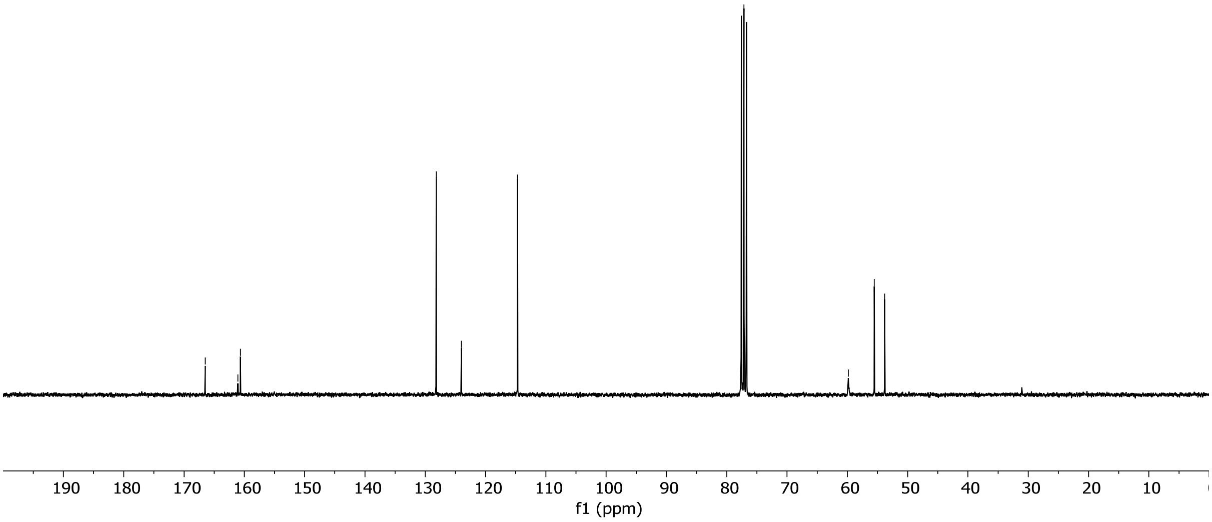

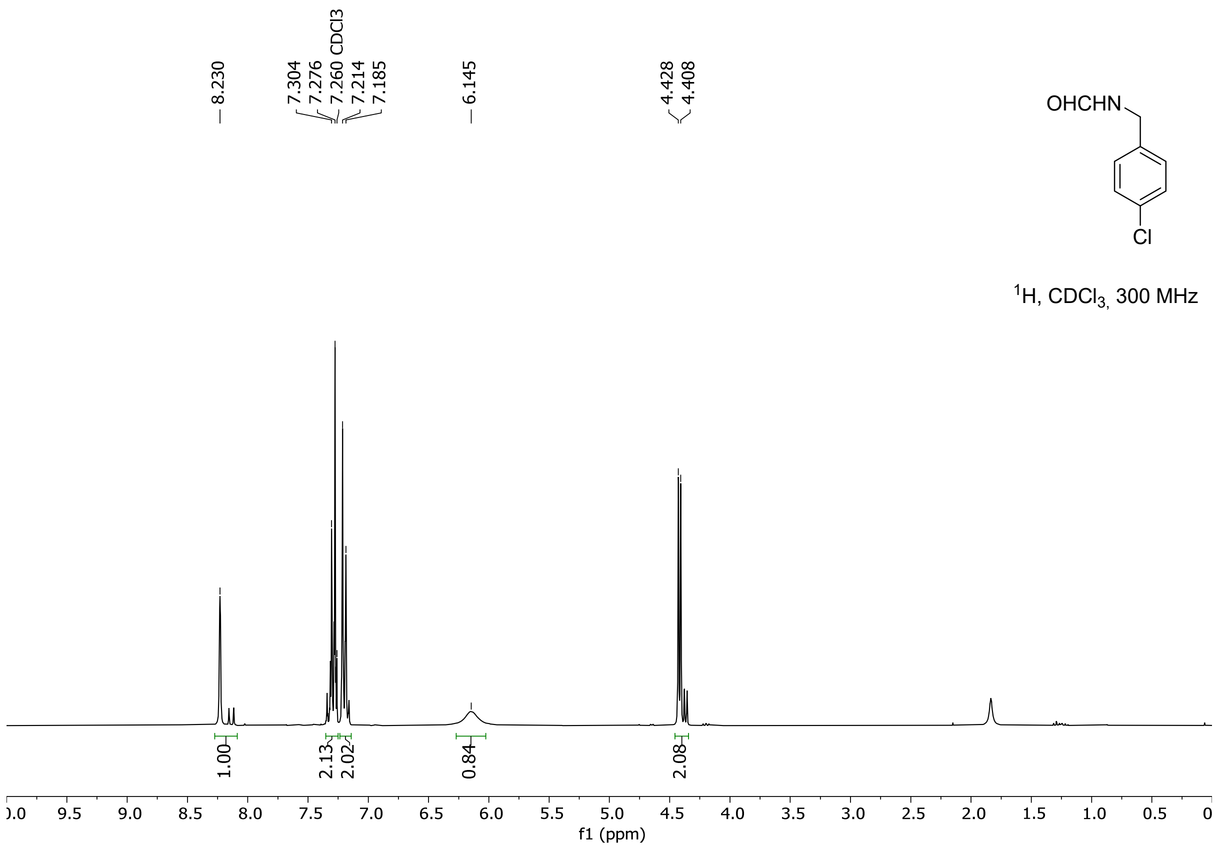
8.230
7.304
7.276
7.260 CDCl3
7.214
7.185
6.145
4.428
4.408
OHCHN
Cl
1H, CDCl3, 300 MHz
1.00
2.13
2.02
0.84
2.08
9.5 9.0 8.5 8.0 7.5 7.0 6.5 6.0 5.5 5.0 4.5 4.0 3.5 3.0 2.5 2.0 1.5 1.0 0.5
f1 (ppm)

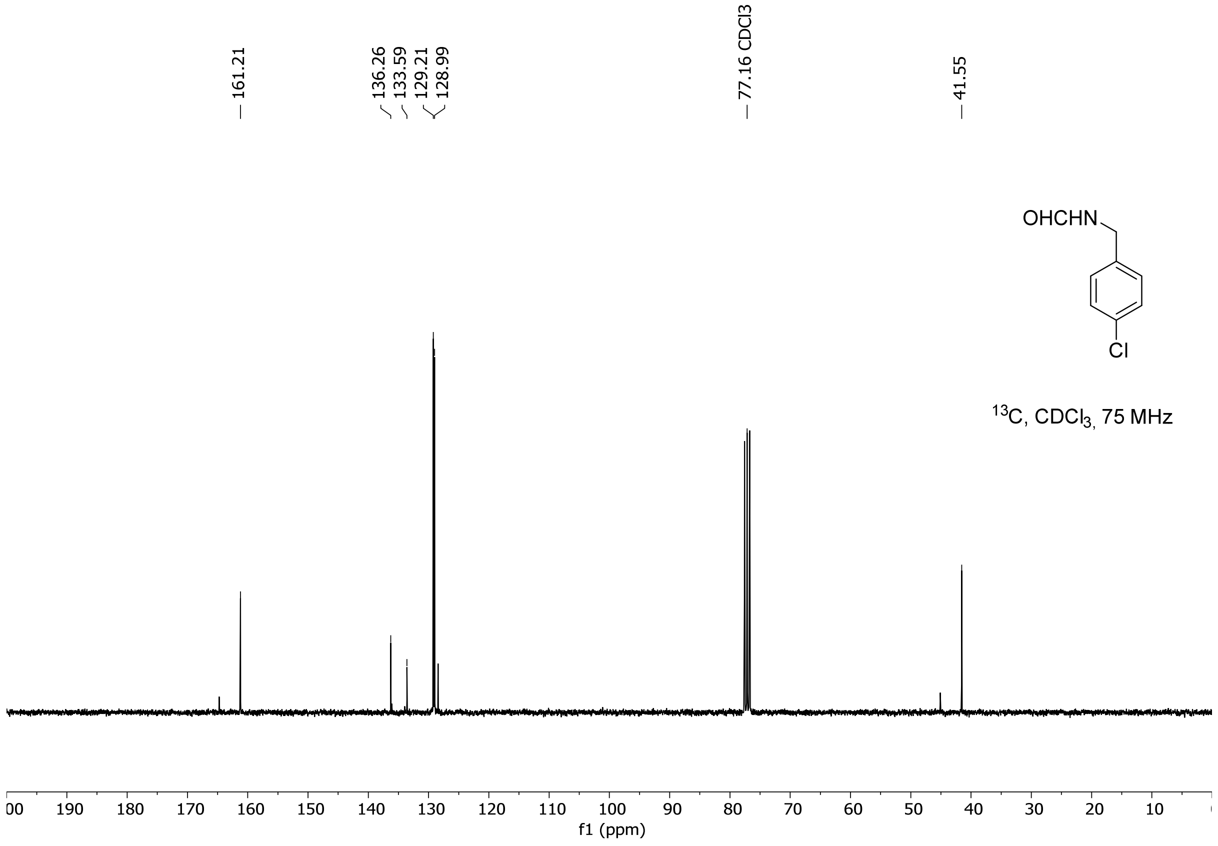
161.21
136.26
133.59
129.21
128.99
77.16 CDCl3
41.55
OHCHN
Cl
13C, CDCl3, 75 MHz
190 180 170 160 150 140 130 120 110 100 90 80 70 60 50 40 30 20 10
f1 (ppm)

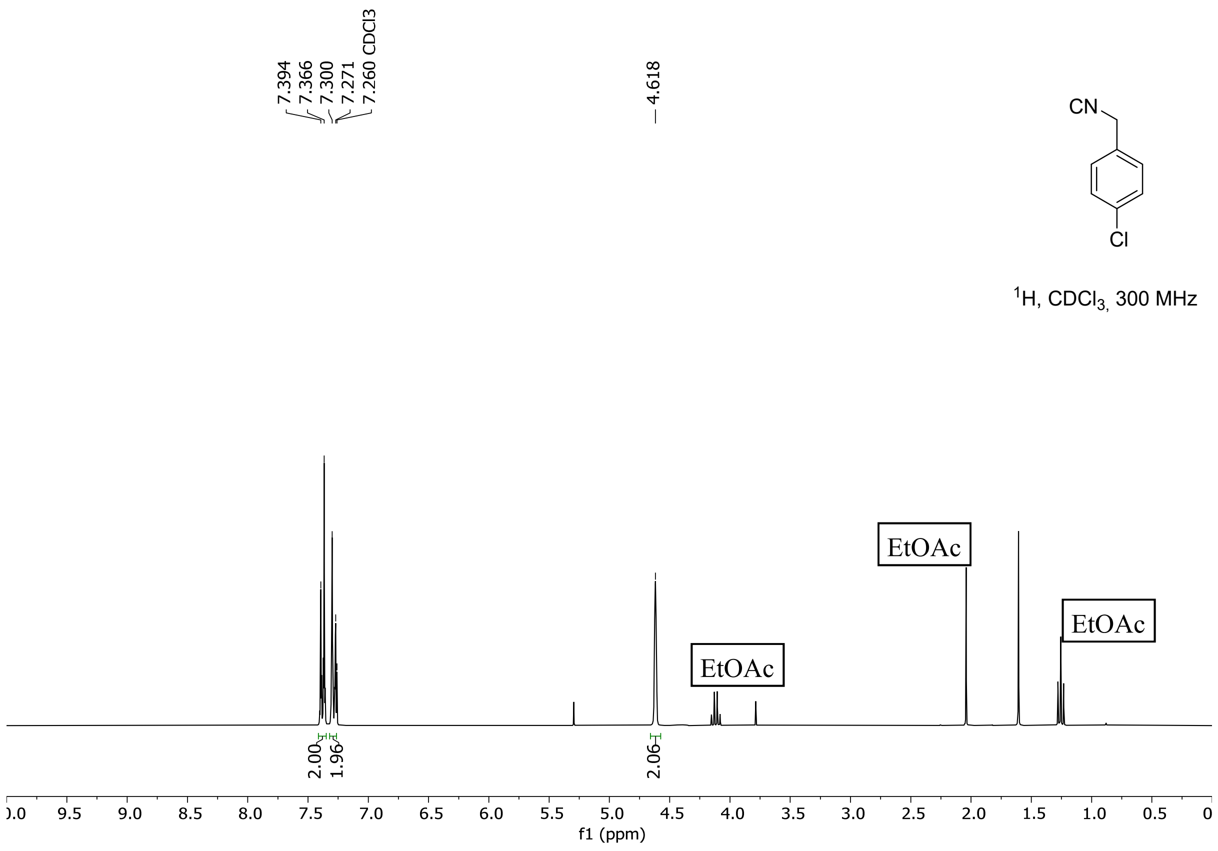
7.394
7.366
7.300
7.271
7.260 CDCl3
4.618
CN
Cl
1H, CDCl3, 300 MHz
EtOAc
EtOAc
EtOAc
2.00
1.96
2.06
f1 (ppm)

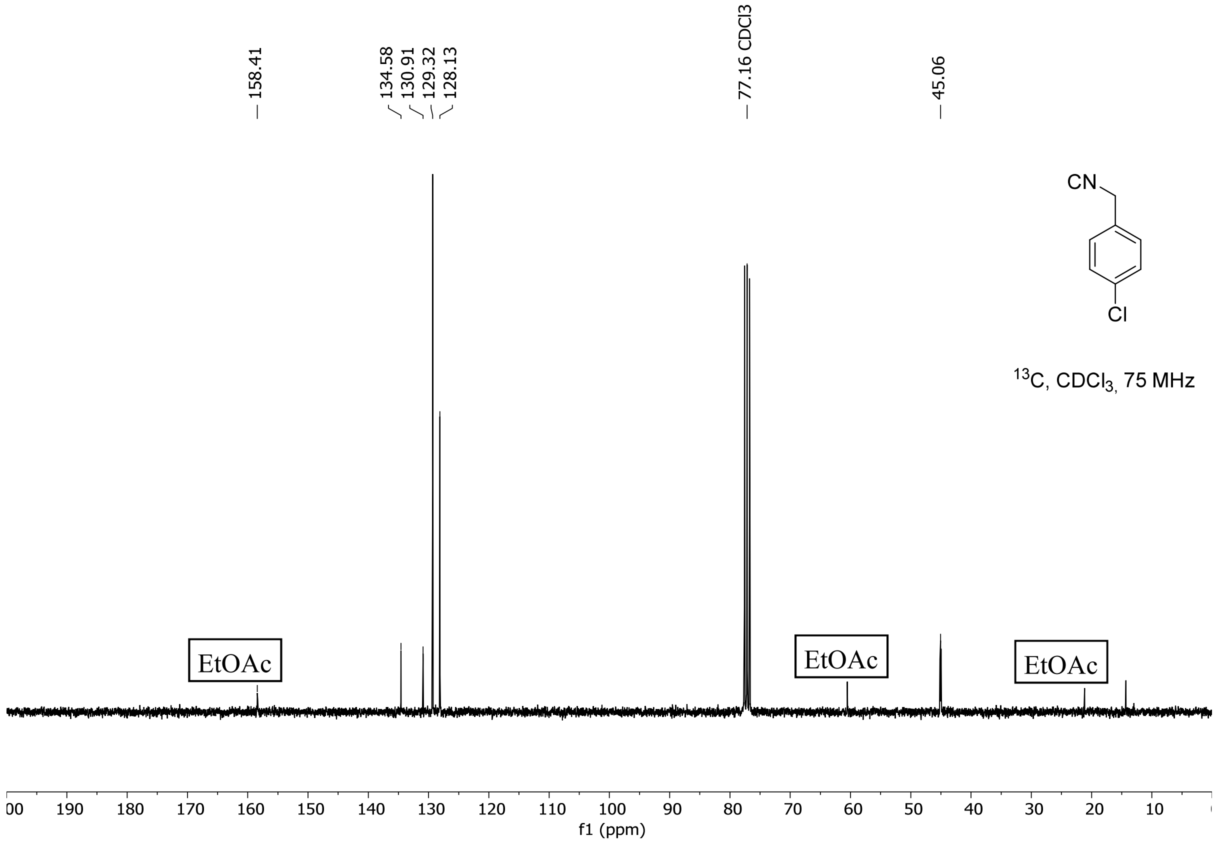
158.41
134.58
130.91
129.32
128.13
77.16 CDCl3
45.06
CN
Cl
13C, CDCl3, 75 MHz
EtOAc
EtOAc
EtOAc
f1 (ppm)

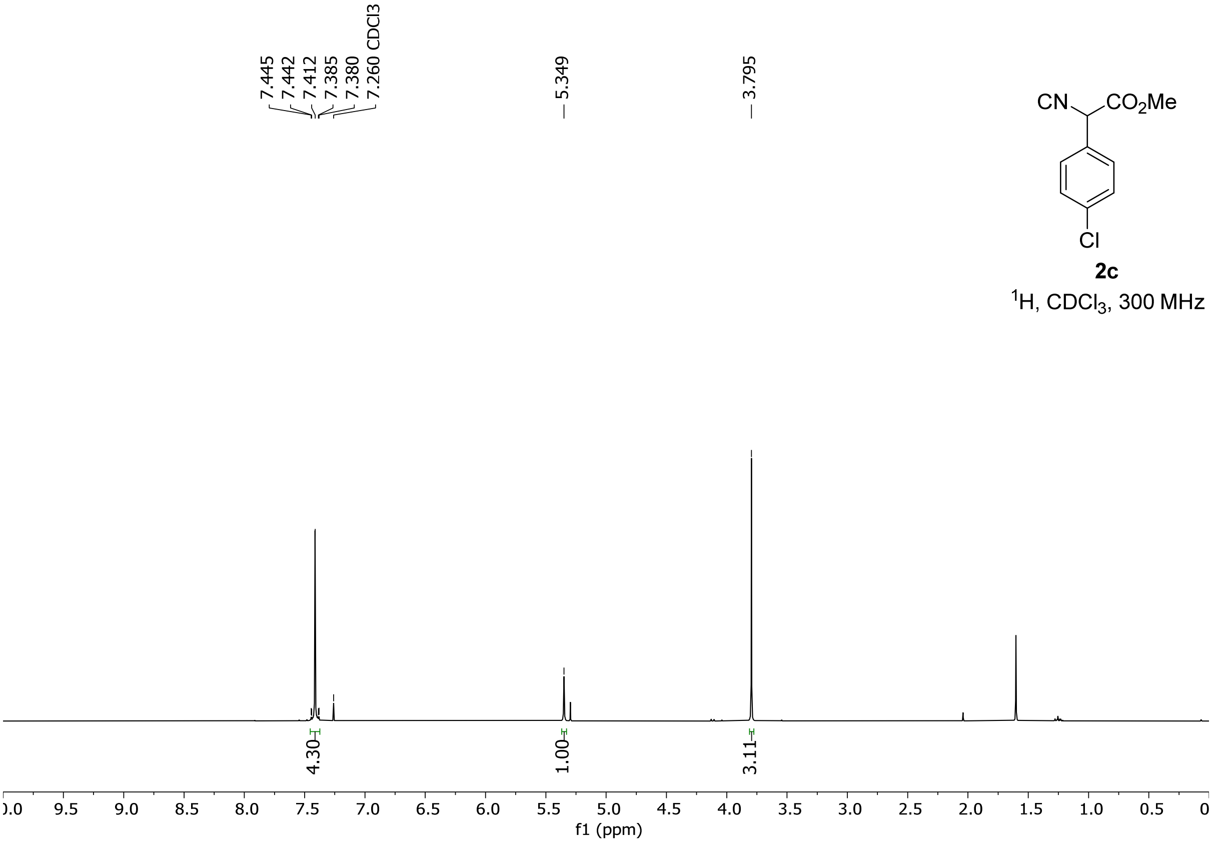

2c

^{1}H, $CDCl_3$, 300 MHz

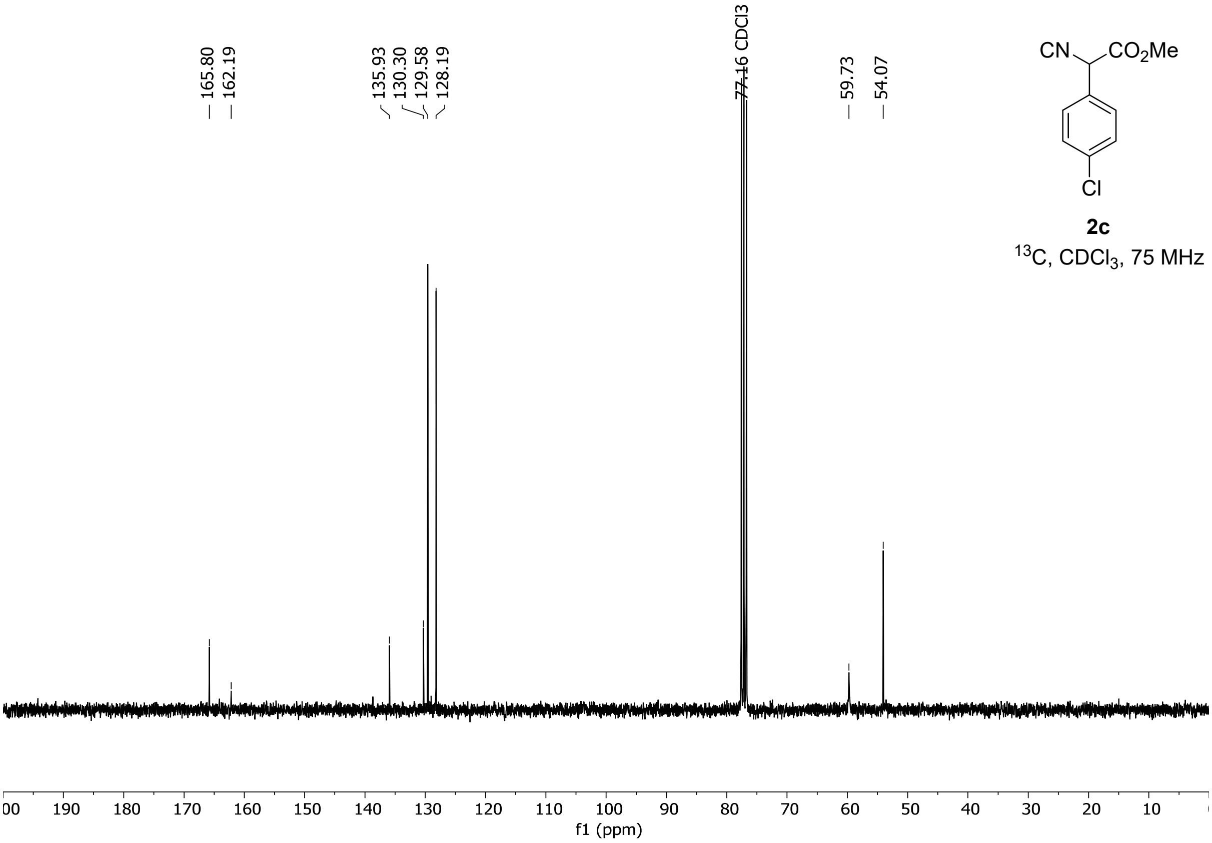

2c

^{13}C, $CDCl_3$, 75 MHz

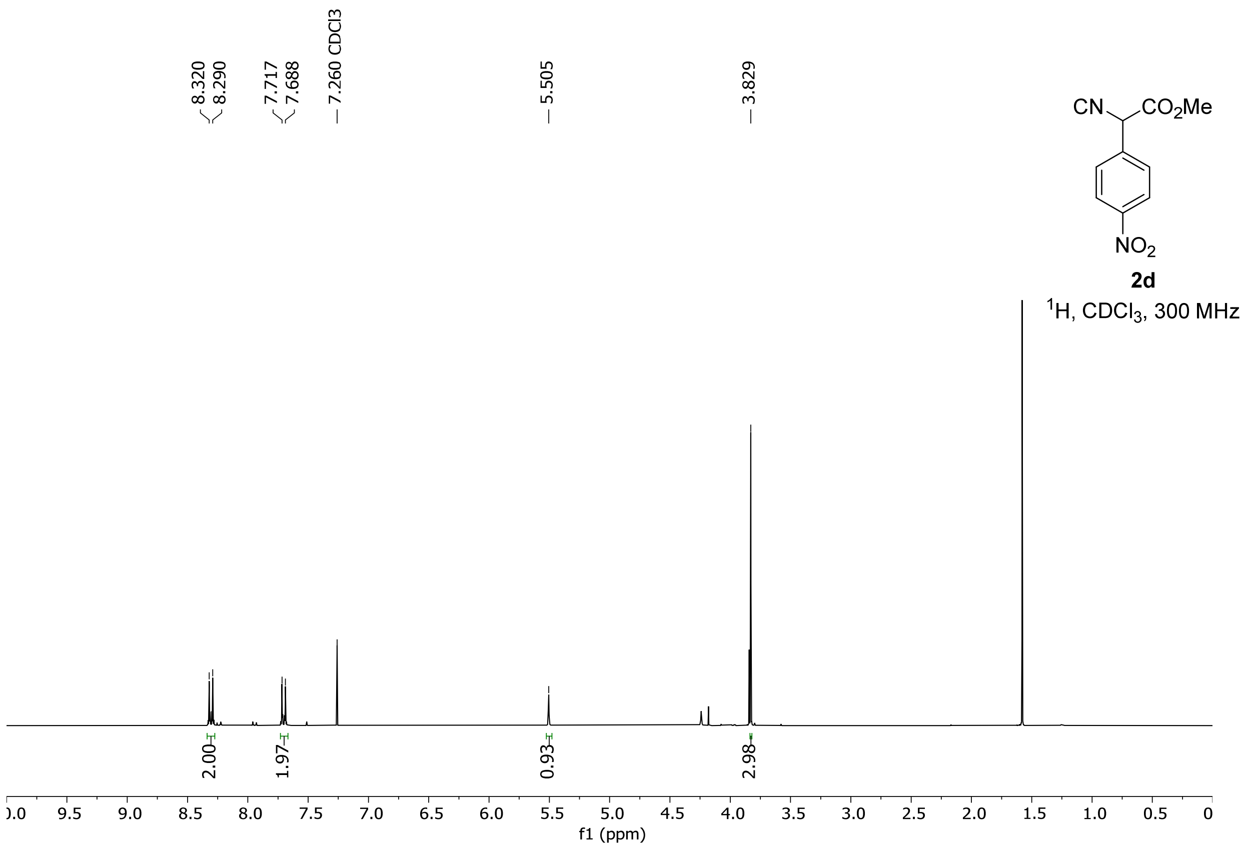
8.320
8.290
7.717
7.688
7.260 CDCl3
5.505
3.829
CN
CO2Me
NO2
2d
1H, CDCl3, 300 MHz
2.00
1.97
0.93
2.98
f1 (ppm)

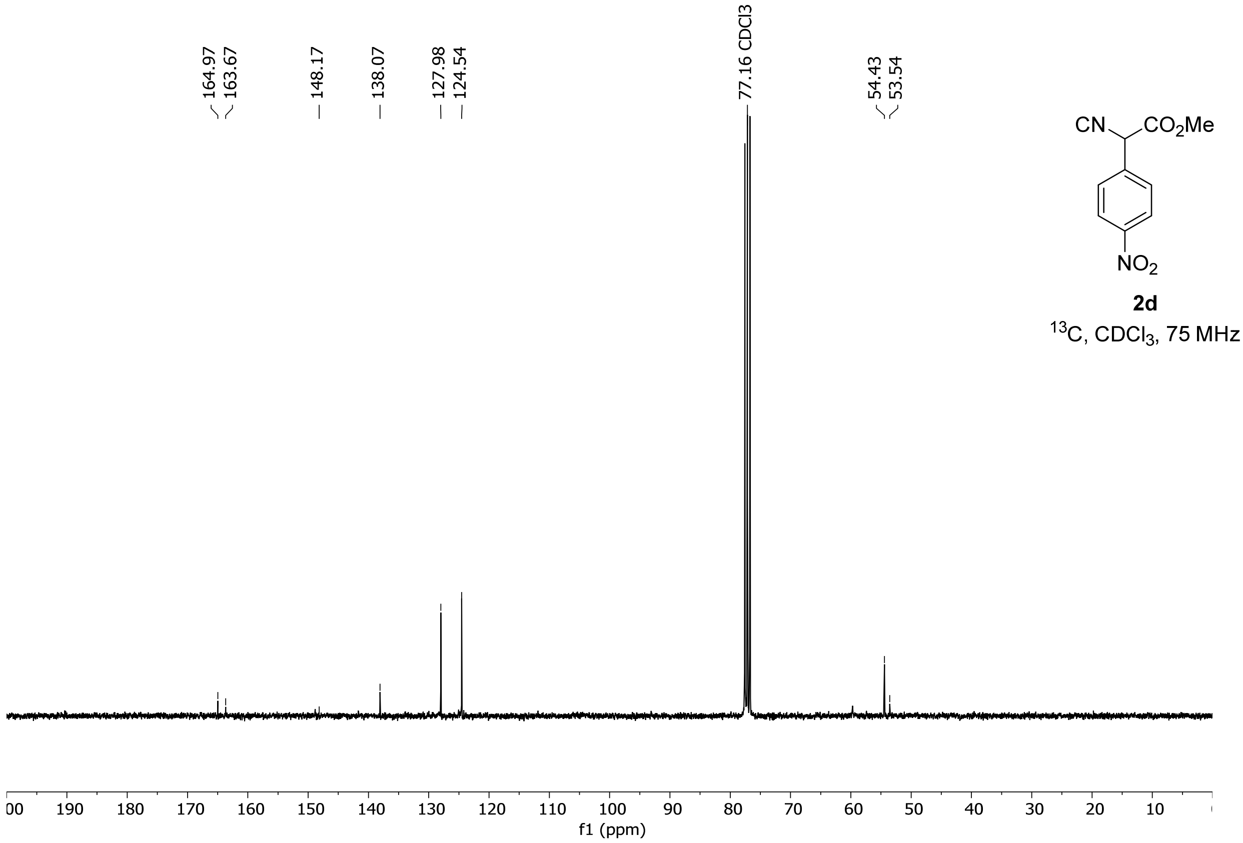
164.97
163.67
148.17
138.07
127.98
124.54
77.16 CDCl3
54.43
53.54
CN
CO2Me
NO2
2d
13C, CDCl3, 75 MHz
f1 (ppm)

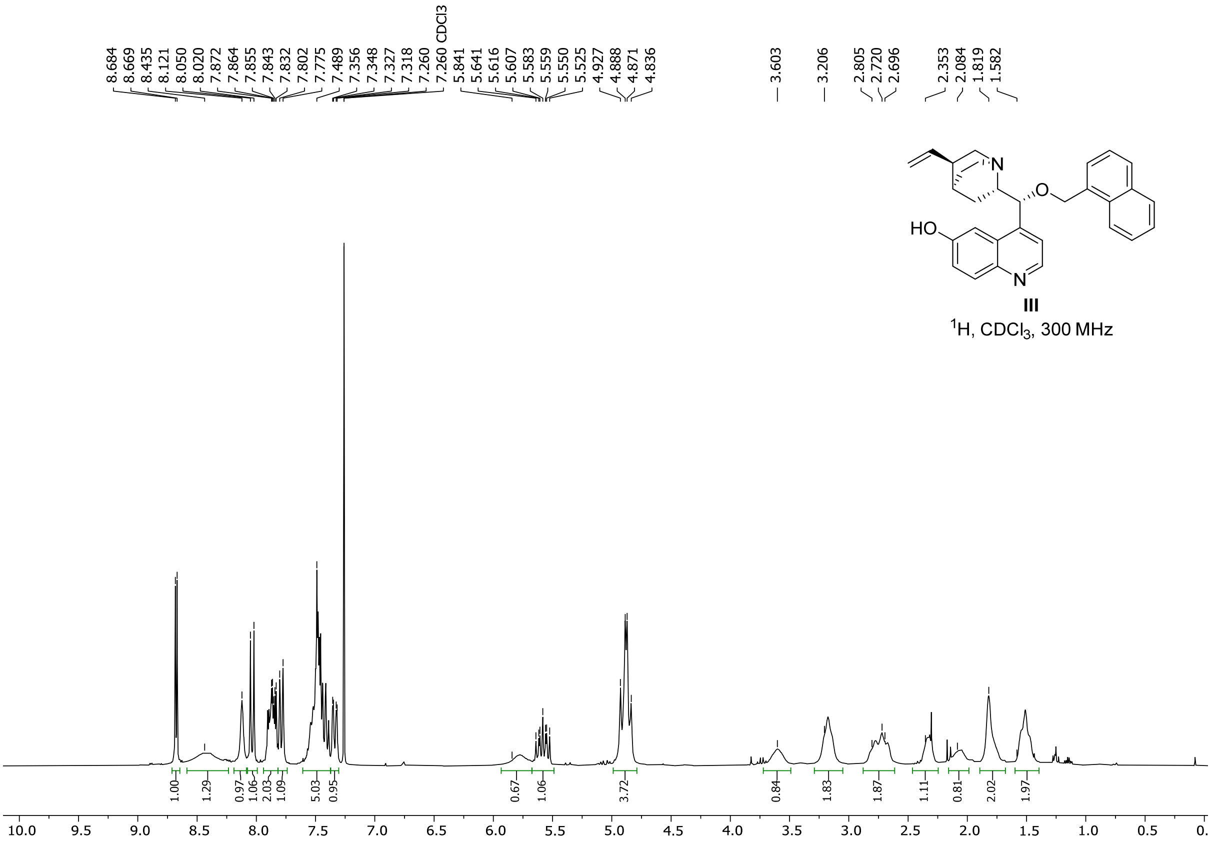
8.684 8.669 8.435 8.121 8.050 8.020 7.872 7.864 7.855 7.843 7.832 7.802 7.775 7.489 7.356 7.348 7.327 7.318 7.260 7.260 CDCl3 5.841 5.641 5.616 5.607 5.583 5.559 5.550 5.525 4.927 4.888 4.871 4.836
3.603 3.206 2.805 2.720 2.696 2.353 2.084 1.819 1.582
HO
N
O
III
^{1}H, $CDCl_3$, 300 MHz
1.00 1.29 0.97 1.06 2.03 1.09 5.03 0.95 0.67 1.06 3.72 0.84 1.83 1.87 1.11 0.81 2.02 1.97
10.0 9.5 9.0 8.5 8.0 7.5 7.0 6.5 6.0 5.5 5.0 4.5 4.0 3.5 3.0 2.5 2.0 1.5 1.0 0.5 0.

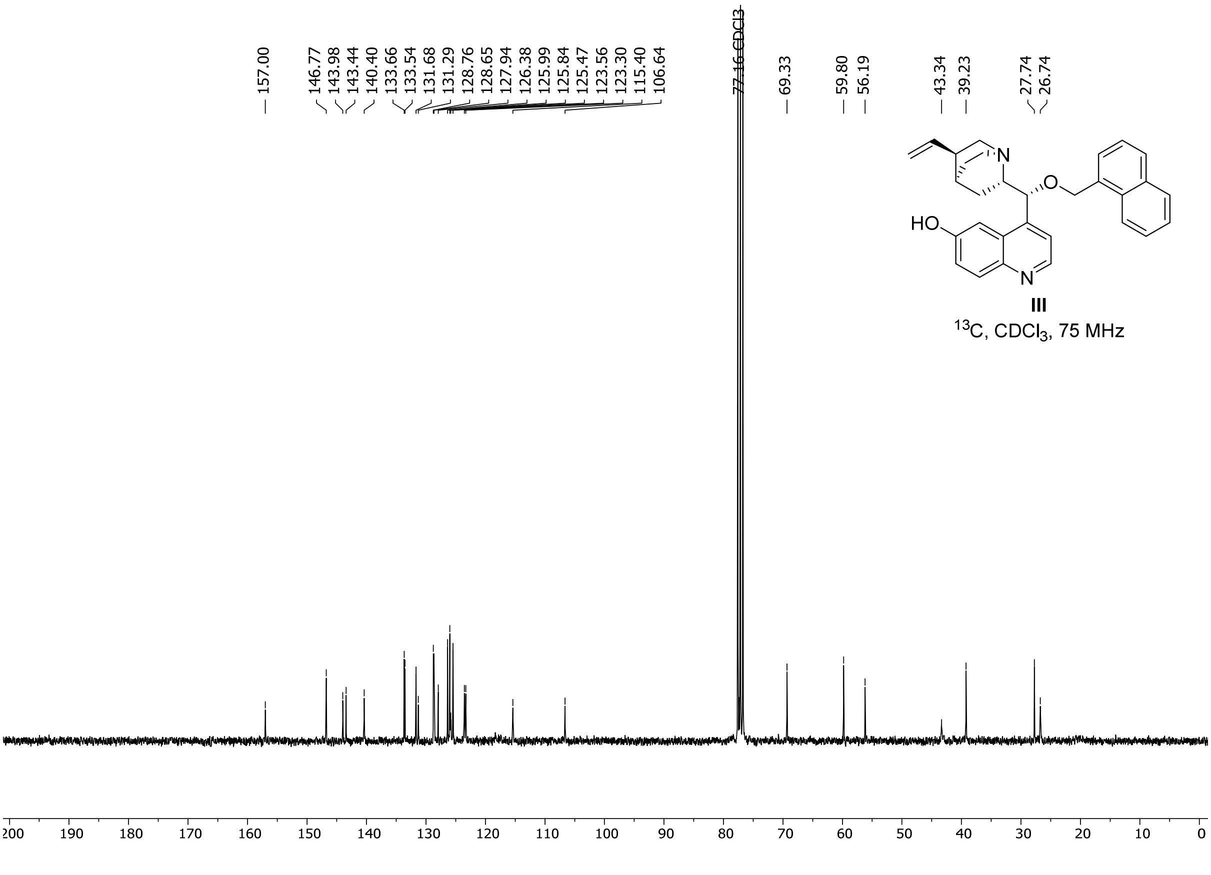
157.00
146.77 143.98 143.44 140.40 133.66 133.54 131.68 131.29 128.76 128.65 127.94 126.38 125.99 125.84 125.47 123.56 123.30 115.40 106.64
77.16 CDCl3
69.33 59.80 56.19 43.34 39.23 27.74 26.74
HO
N
O
III
^{13}C, $CDCl_3$, 75 MHz
200 190 180 170 160 150 140 130 120 110 100 90 80 70 60 50 40 30 20 10 0

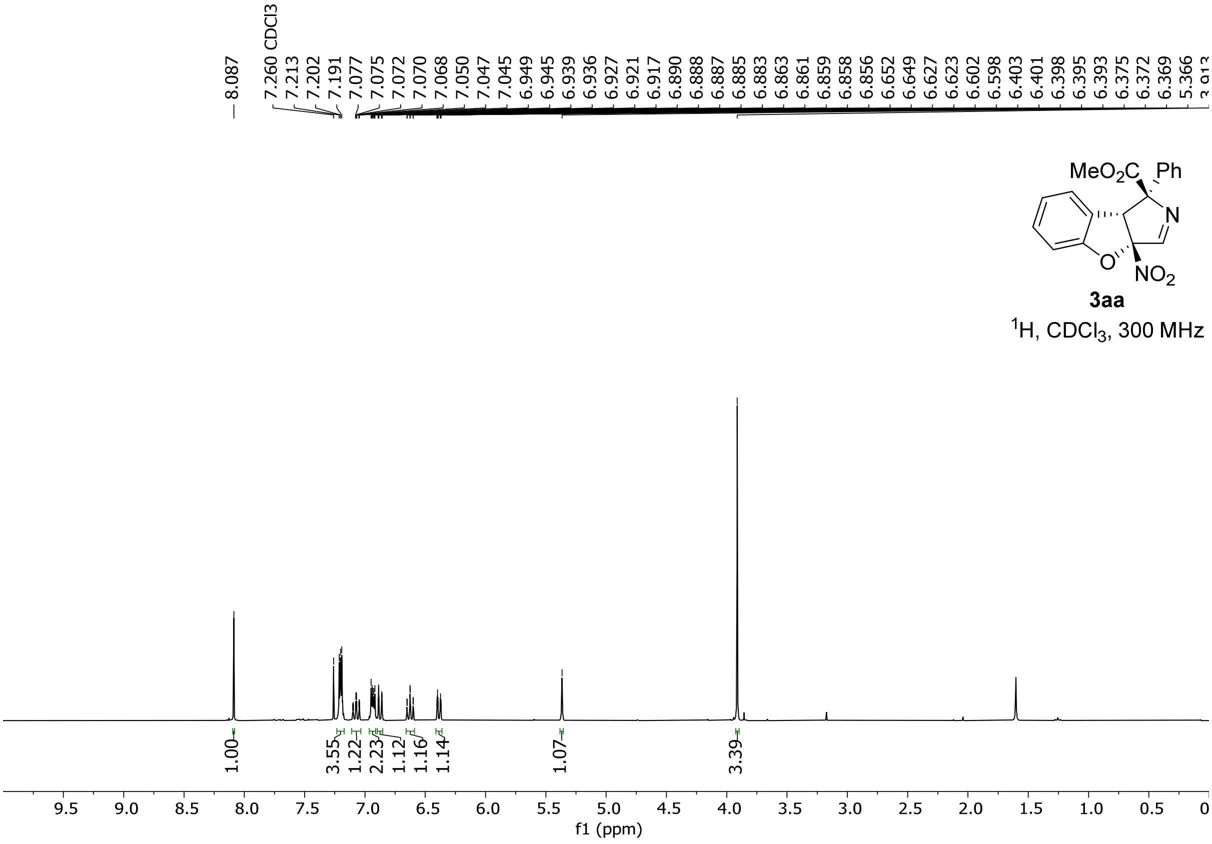
8.087
7.260 CDCl3
7.213
7.202
7.191
7.077
7.075
7.072
7.070
7.068
7.050
7.047
7.045
6.949
6.945
6.939
6.936
6.927
6.921
6.917
6.890
6.888
6.887
6.885
6.883
6.863
6.861
6.859
6.858
6.856
6.652
6.649
6.627
6.623
6.602
6.598
6.403
6.401
6.398
6.395
6.393
6.375
6.372
6.369
5.366
MeO2C
Ph
N
O
NO2
3aa
1H, CDCl3, 300 MHz
1.00
3.55
1.22
2.23
1.12
1.16
1.14
1.07
3.39
9.5 9.0 8.5 8.0 7.5 7.0 6.5 6.0 5.5 5.0 4.5 4.0 3.5 3.0 2.5 2.0 1.5 1.0 0.5 0
f1 (ppm)

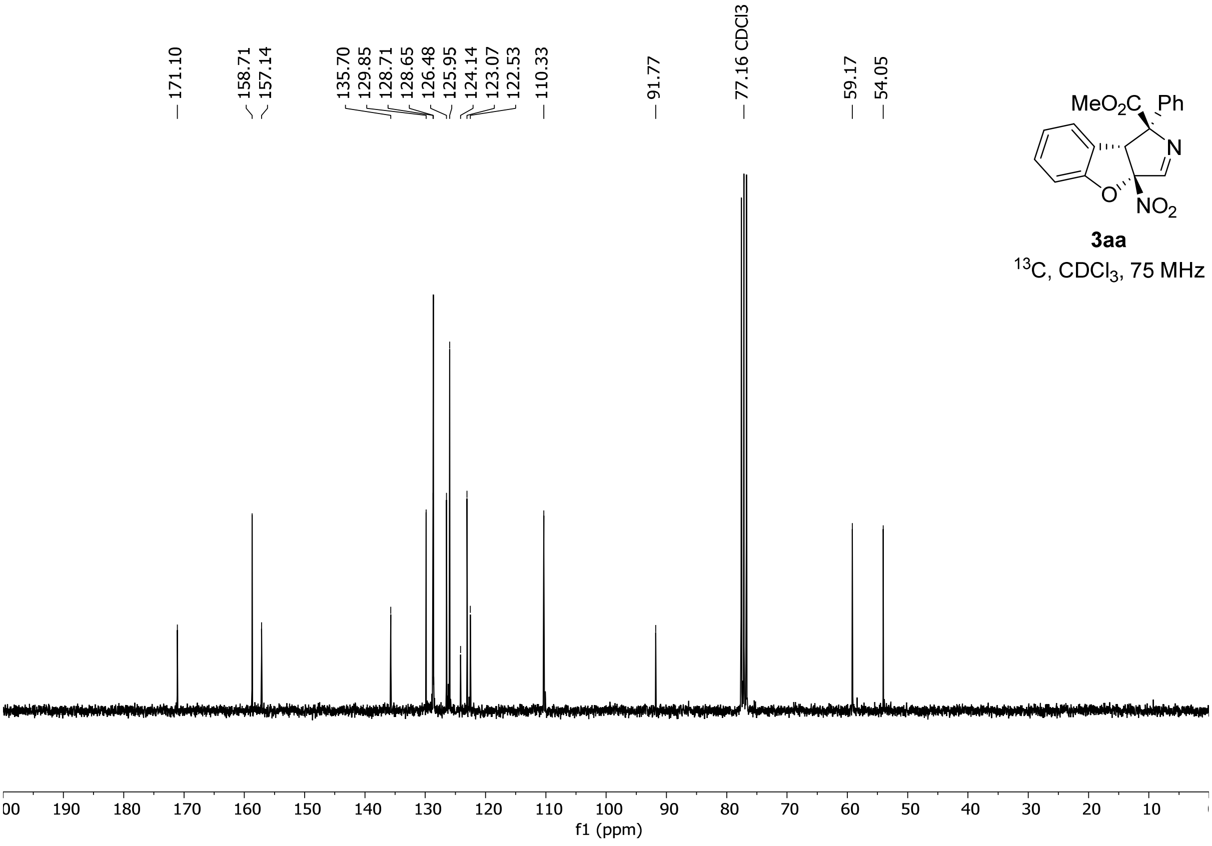
171.10
158.71
157.14
135.70
129.85
128.71
128.65
126.48
125.95
124.14
123.07
122.53
110.33
91.77
77.16 CDCl3
59.17
54.05
MeO2C
Ph
N
O
NO2
3aa
13C, CDCl3, 75 MHz
190 180 170 160 150 140 130 120 110 100 90 80 70 60 50 40 30 20 10
f1 (ppm)

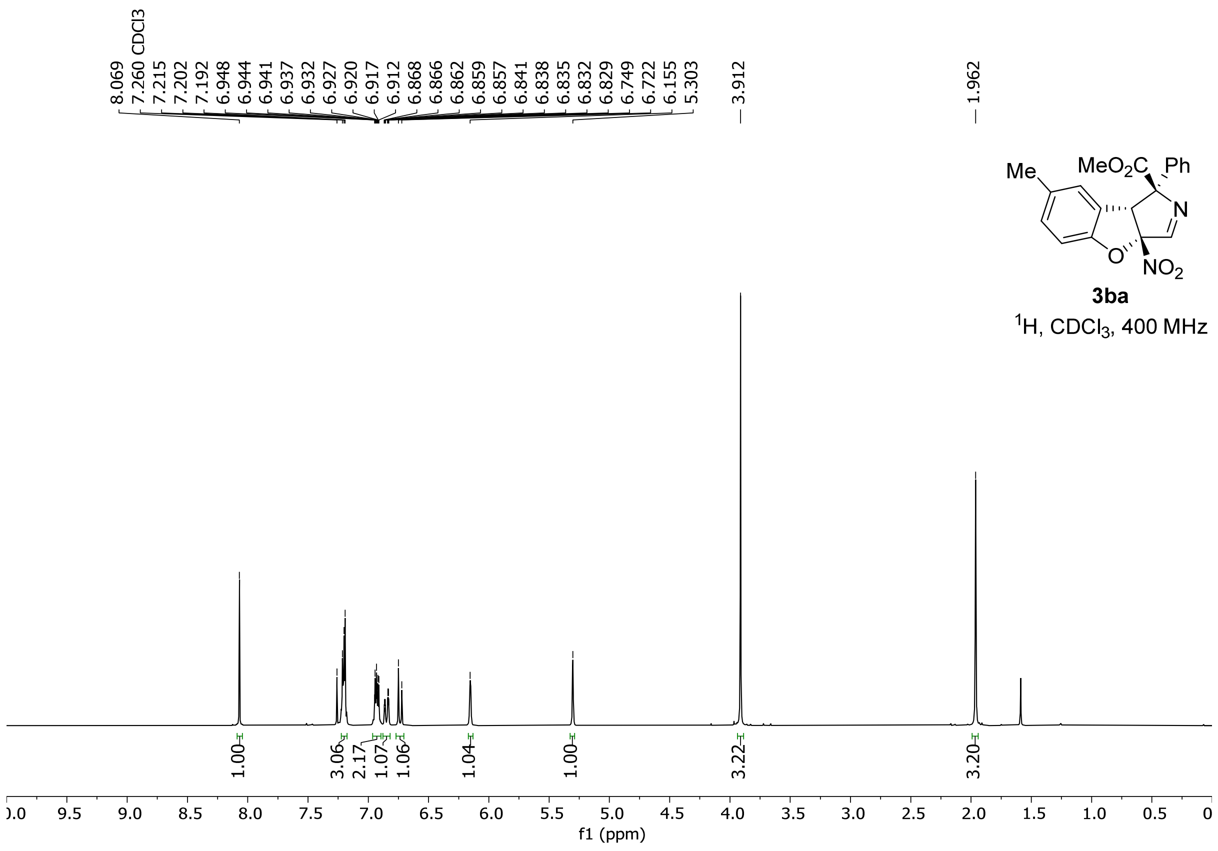
8.069
7.260 CDCl3
7.215
7.202
7.192
6.948
6.944
6.941
6.937
6.932
6.927
6.920
6.917
6.912
6.868
6.866
6.862
6.859
6.857
6.841
6.838
6.835
6.832
6.829
6.749
6.722
6.155
5.303
3.912
1.962
Me
MeO2C
Ph
N
O
NO2
3ba
1H, CDCl3, 400 MHz
1.00
3.06
2.17
1.07
1.06
1.04
1.00
3.22
3.20
f1 (ppm)

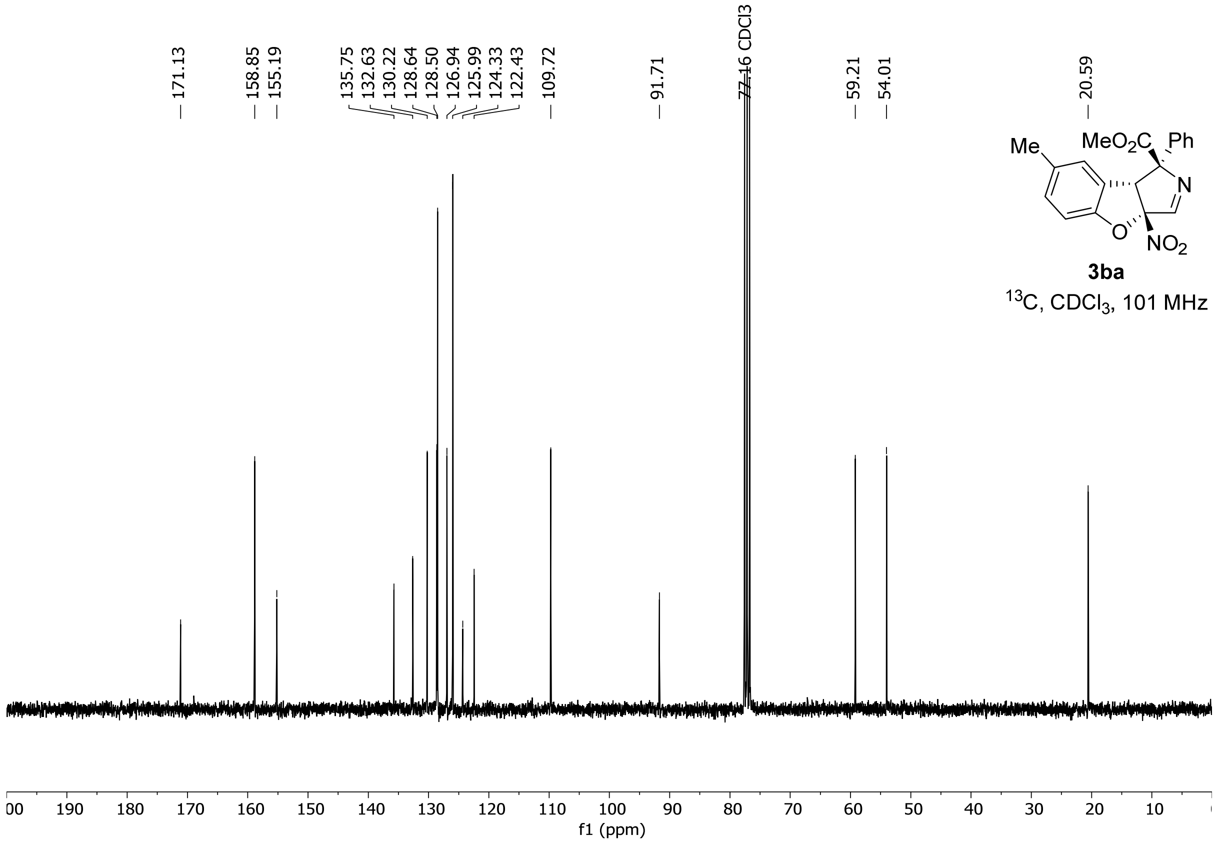
171.13
158.85
155.19
135.75
132.63
130.22
128.64
128.50
126.94
125.99
124.33
122.43
109.72
91.71
77.16 CDCl3
59.21
54.01
20.59
Me
MeO2C
Ph
N
O
NO2
3ba
13C, CDCl3, 101 MHz
f1 (ppm)

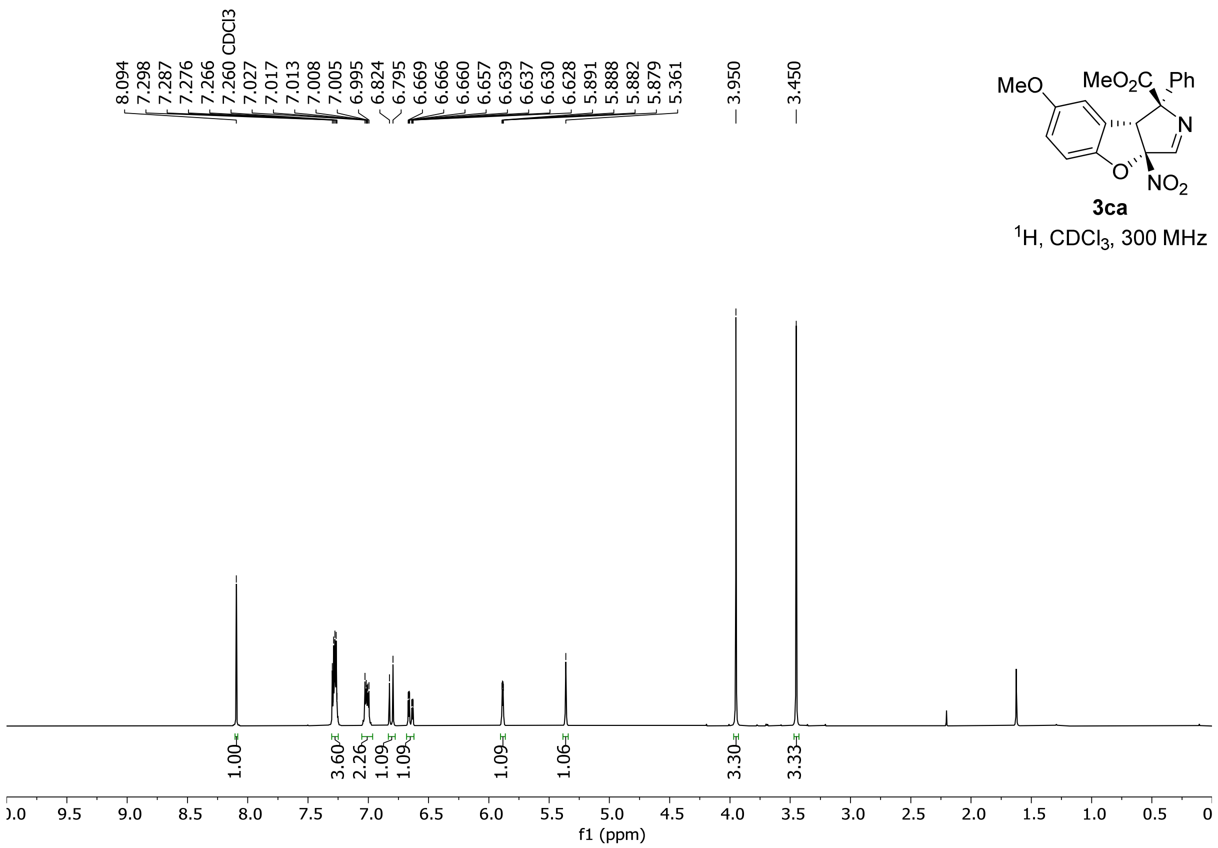

MeO
MeO2C
Ph
N
O
NO2
3ca
1H, CDCl3, 300 MHz
f1 (ppm)

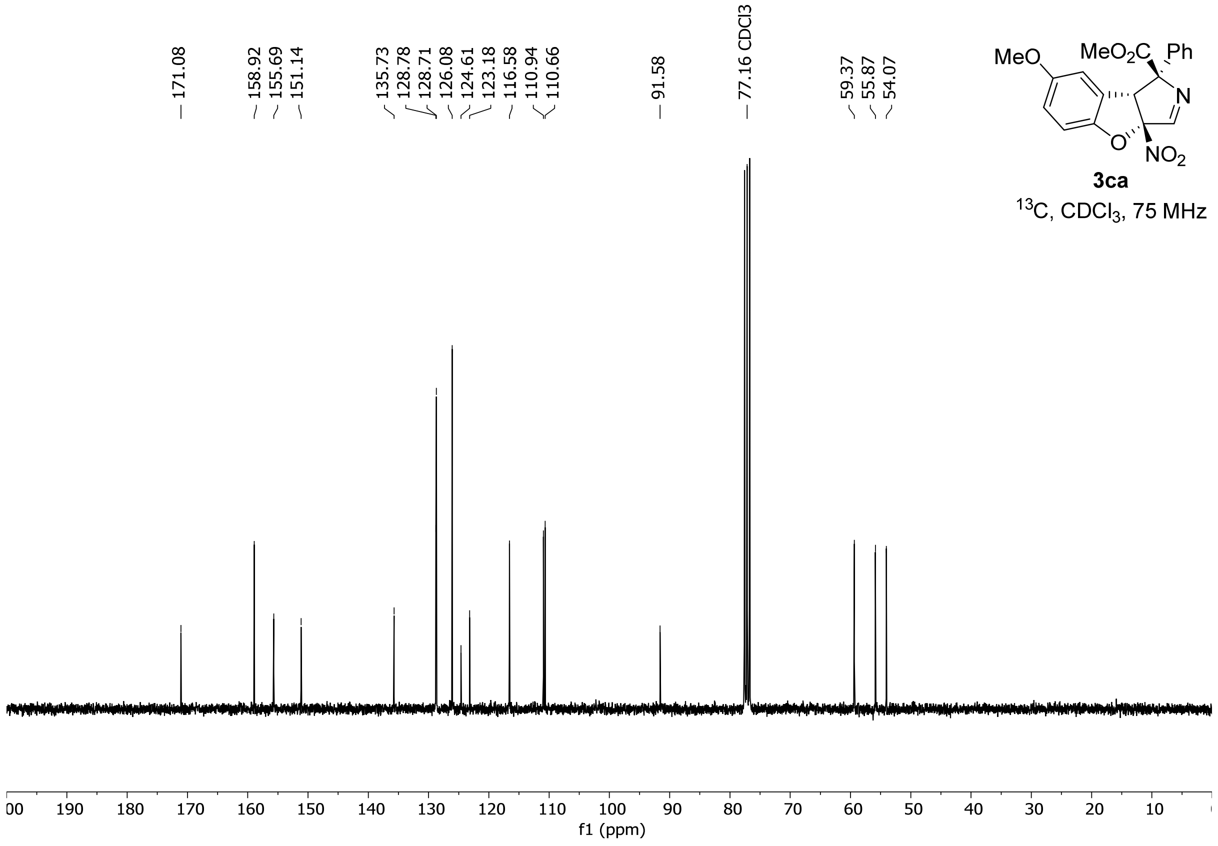

MeO
MeO2C
Ph
N
O
NO2
3ca
13C, CDCl3, 75 MHz
f1 (ppm)

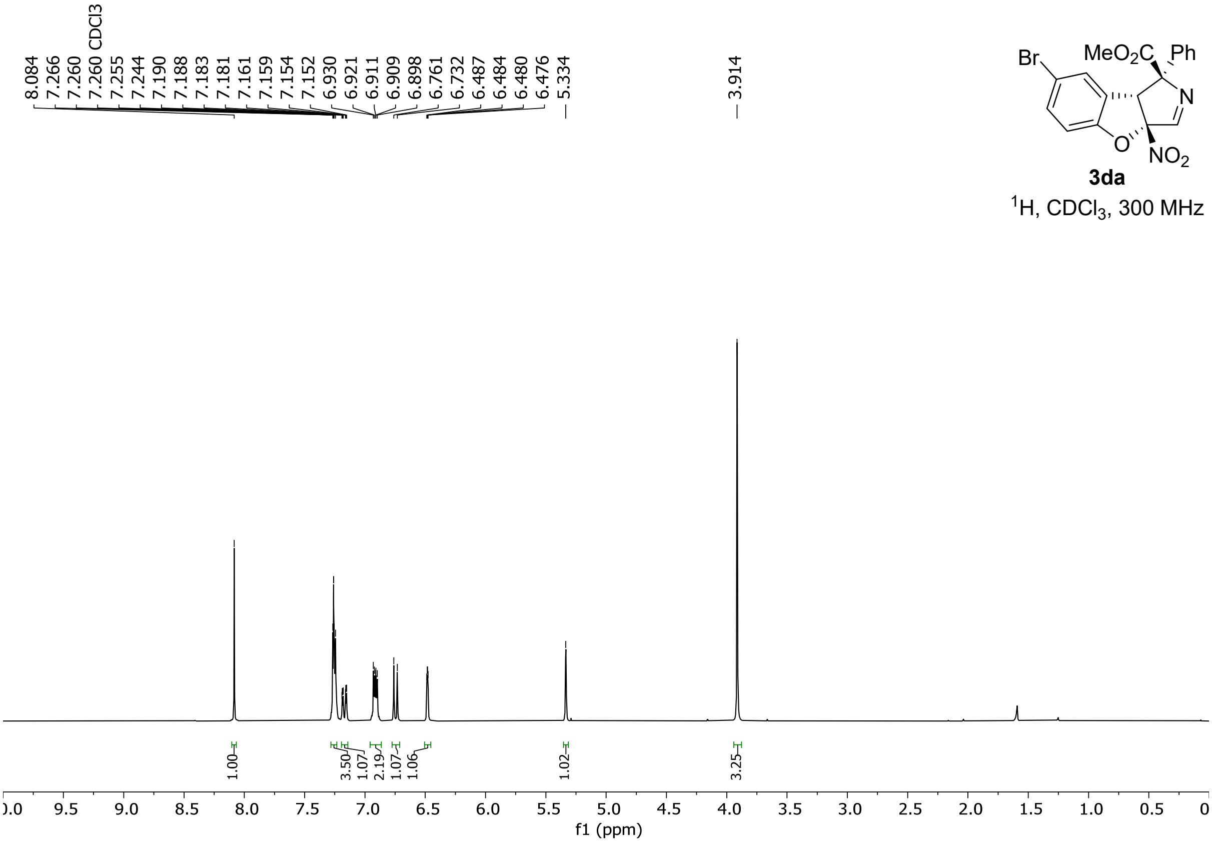
8.084
7.266
7.260
7.260 CDCl3
7.255
7.244
7.190
7.188
7.183
7.181
7.161
7.159
7.154
7.152
6.930
6.921
6.911
6.909
6.898
6.761
6.732
6.487
6.484
6.480
6.476
5.334
3.914
Br
MeO2C
Ph
N
O
NO2
3da
1H, CDCl3, 300 MHz
1.00
3.50
1.07
2.19
1.07
1.06
1.02
3.25
f1 (ppm)

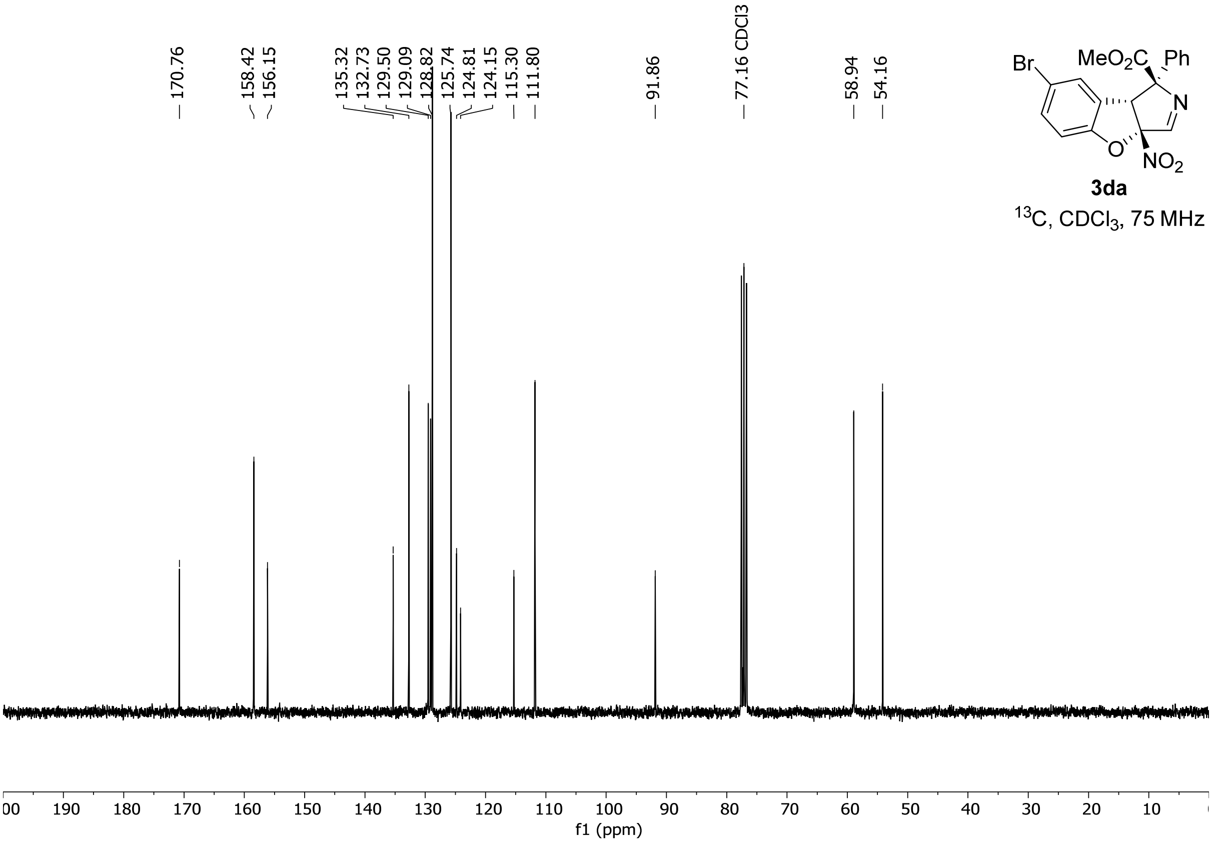
170.76
158.42
156.15
135.32
132.73
129.50
129.09
128.82
125.74
124.81
124.15
115.30
111.80
91.86
77.16 CDCl3
58.94
54.16
Br
MeO2C
Ph
N
O
NO2
3da
13C, CDCl3, 75 MHz
f1 (ppm)

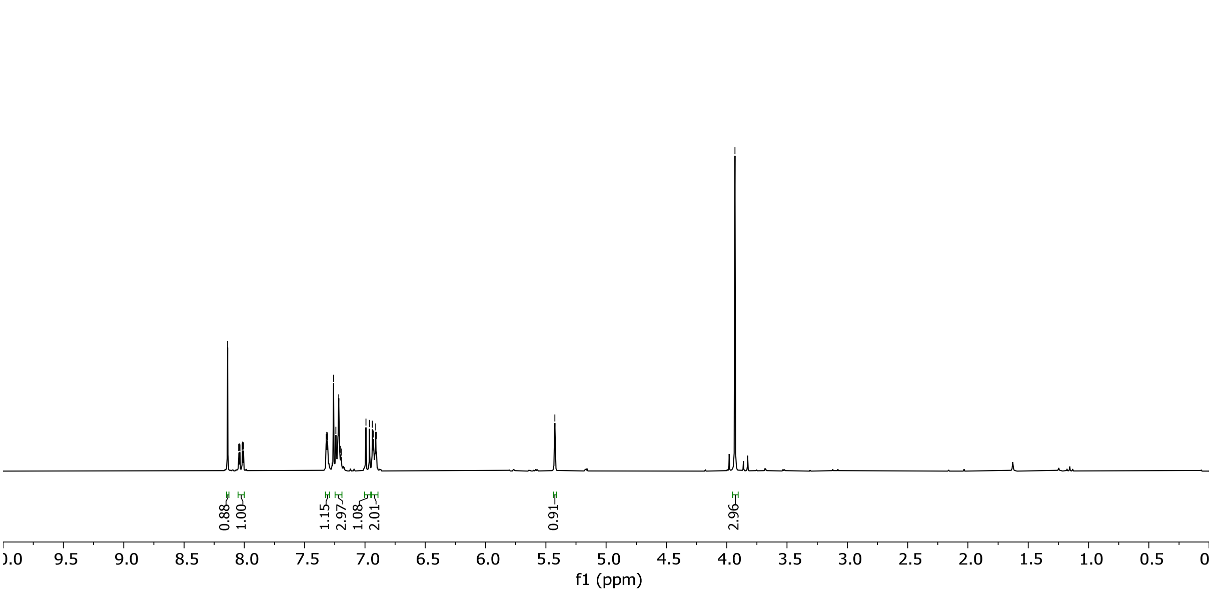

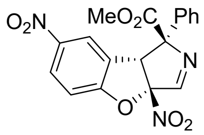

3ea

^{1}H, $CDCl_3$, 300 MHz

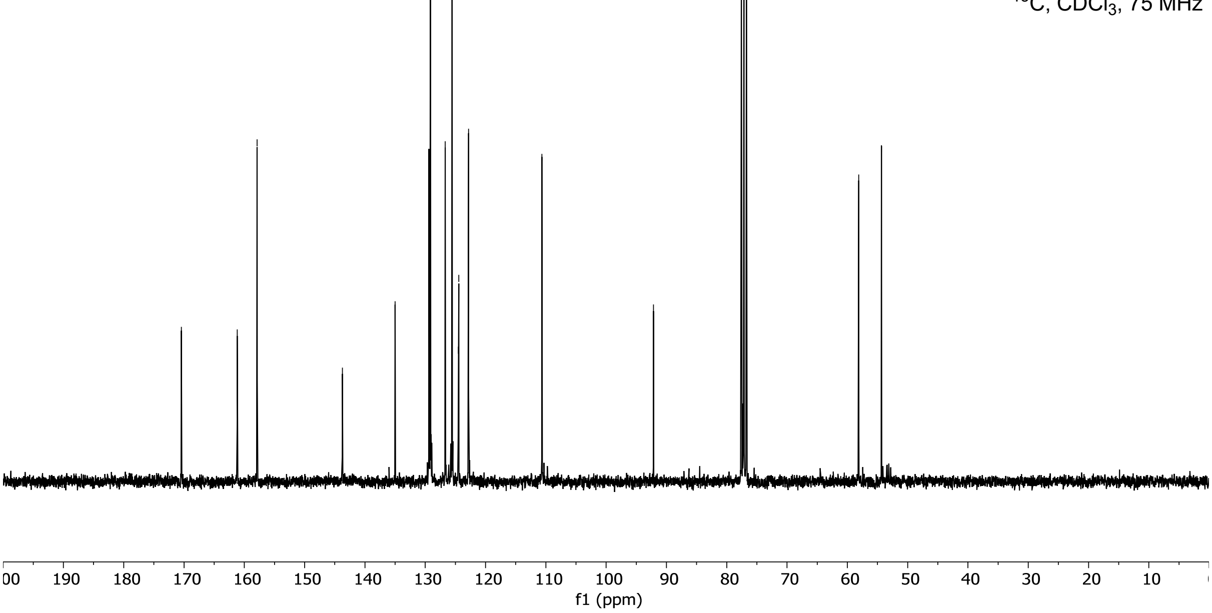

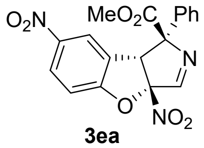

3ea

^{13}C, $CDCl_3$, 75 MHz

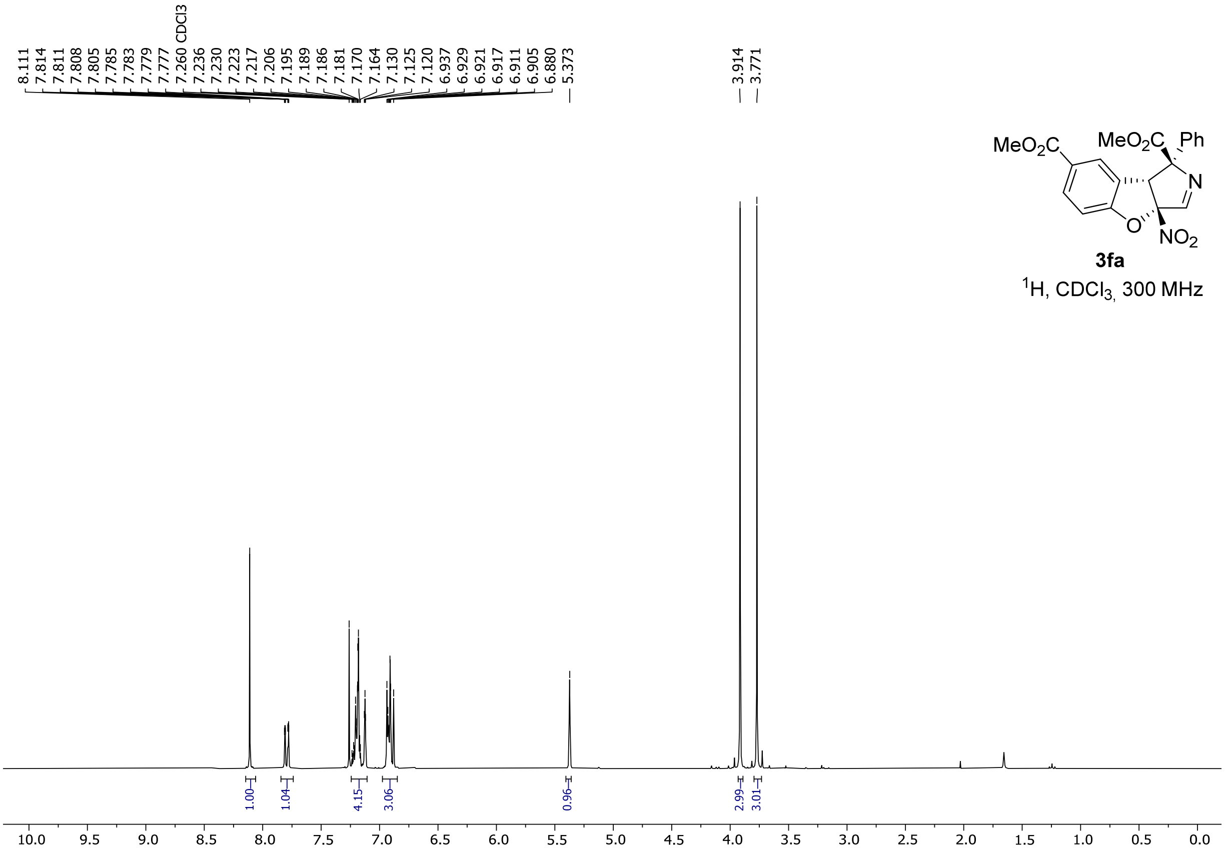
8.111
7.814
7.811
7.808
7.805
7.785
7.783
7.779
7.777
7.260 CDCl3
7.236
7.230
7.223
7.217
7.206
7.195
7.189
7.186
7.181
7.170
7.164
7.130
7.125
7.120
6.937
6.929
6.921
6.917
6.911
6.905
6.880
5.373
3.914
3.771
MeO2C
MeO2C
Ph
N
O
NO2
3fa
1H, CDCl3, 300 MHz
1.00
1.04
4.15
3.06
0.96
2.99
3.01
10.0 9.5 9.0 8.5 8.0 7.5 7.0 6.5 6.0 5.5 5.0 4.5 4.0 3.5 3.0 2.5 2.0 1.5 1.0 0.5 0.0

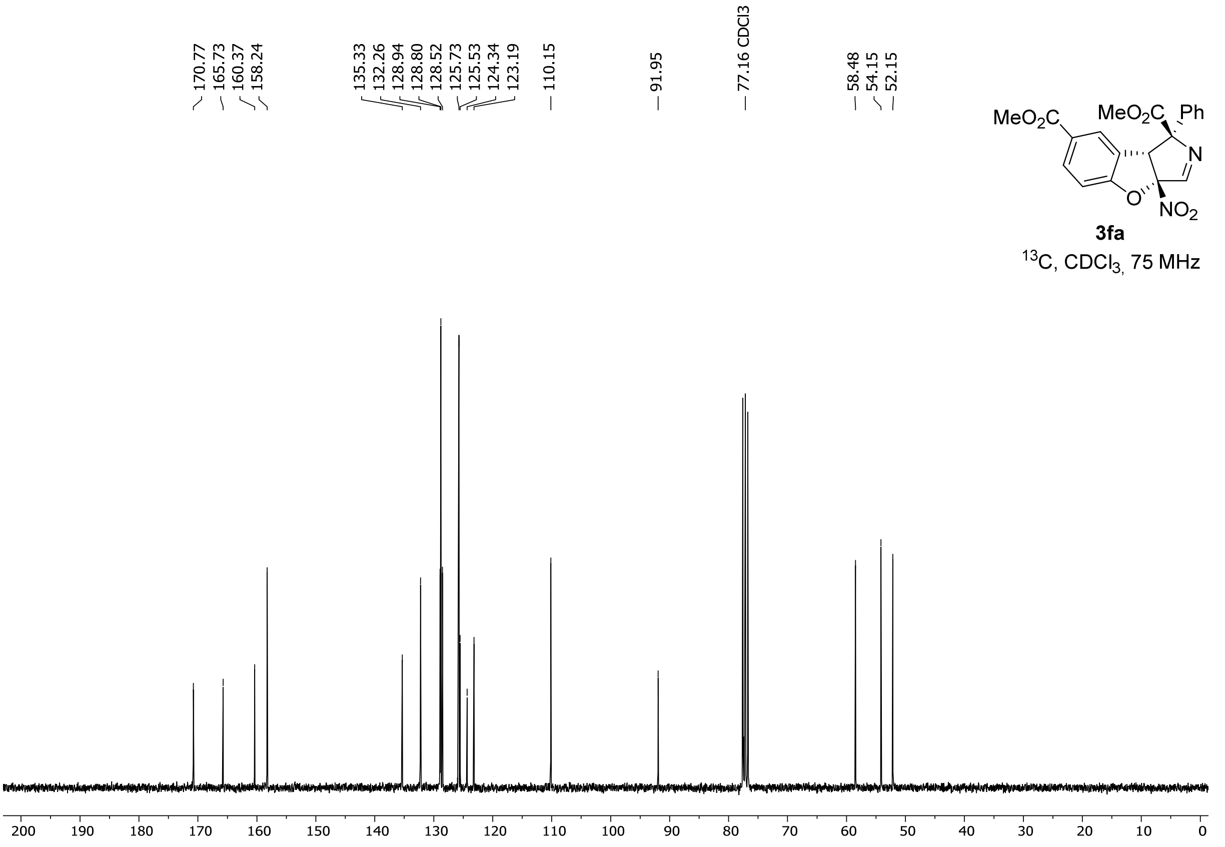
170.77
165.73
160.37
158.24
135.33
132.26
128.94
128.80
128.52
125.73
125.53
124.34
123.19
110.15
91.95
77.16 CDCl3
58.48
54.15
52.15
MeO2C
MeO2C
Ph
N
O
NO2
3fa
13C, CDCl3, 75 MHz
200 190 180 170 160 150 140 130 120 110 100 90 80 70 60 50 40 30 20 10 0

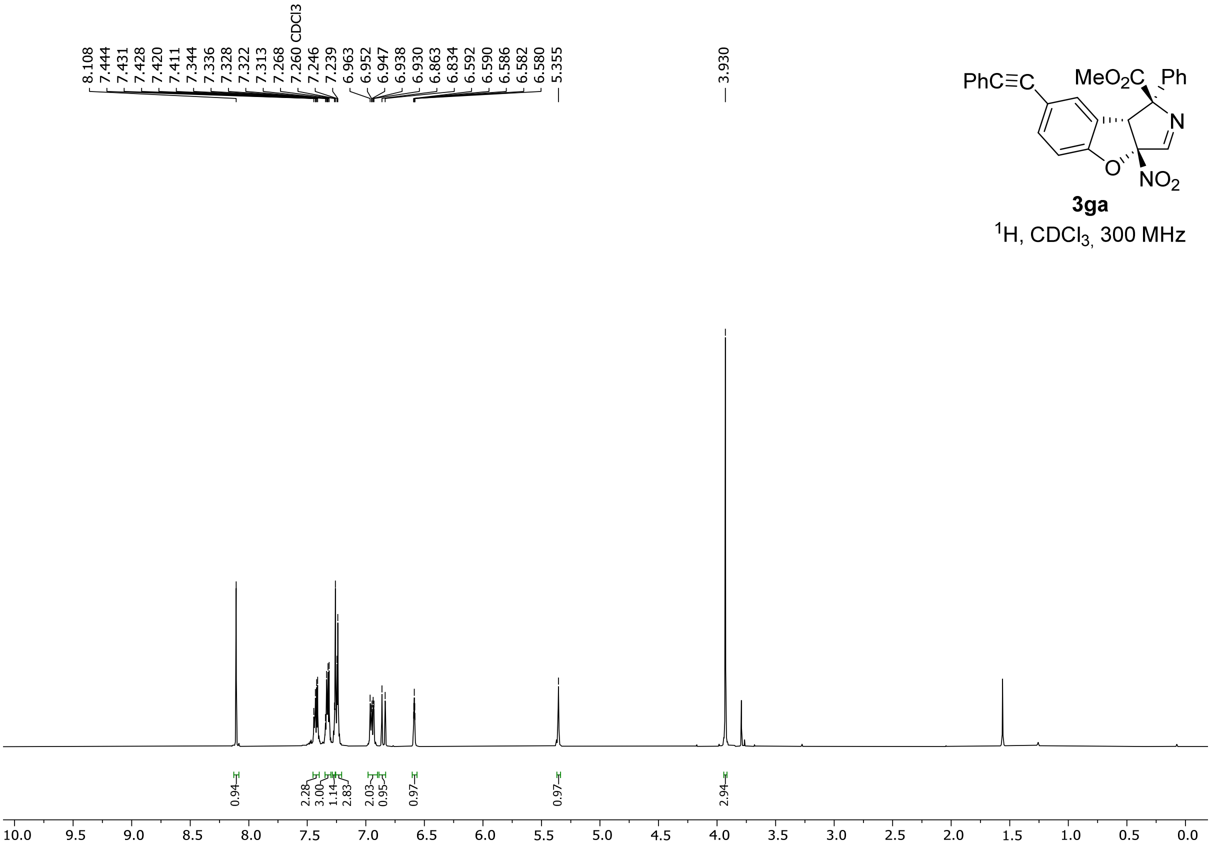

3ga

^{1}H, $CDCl_3$, 300 MHz

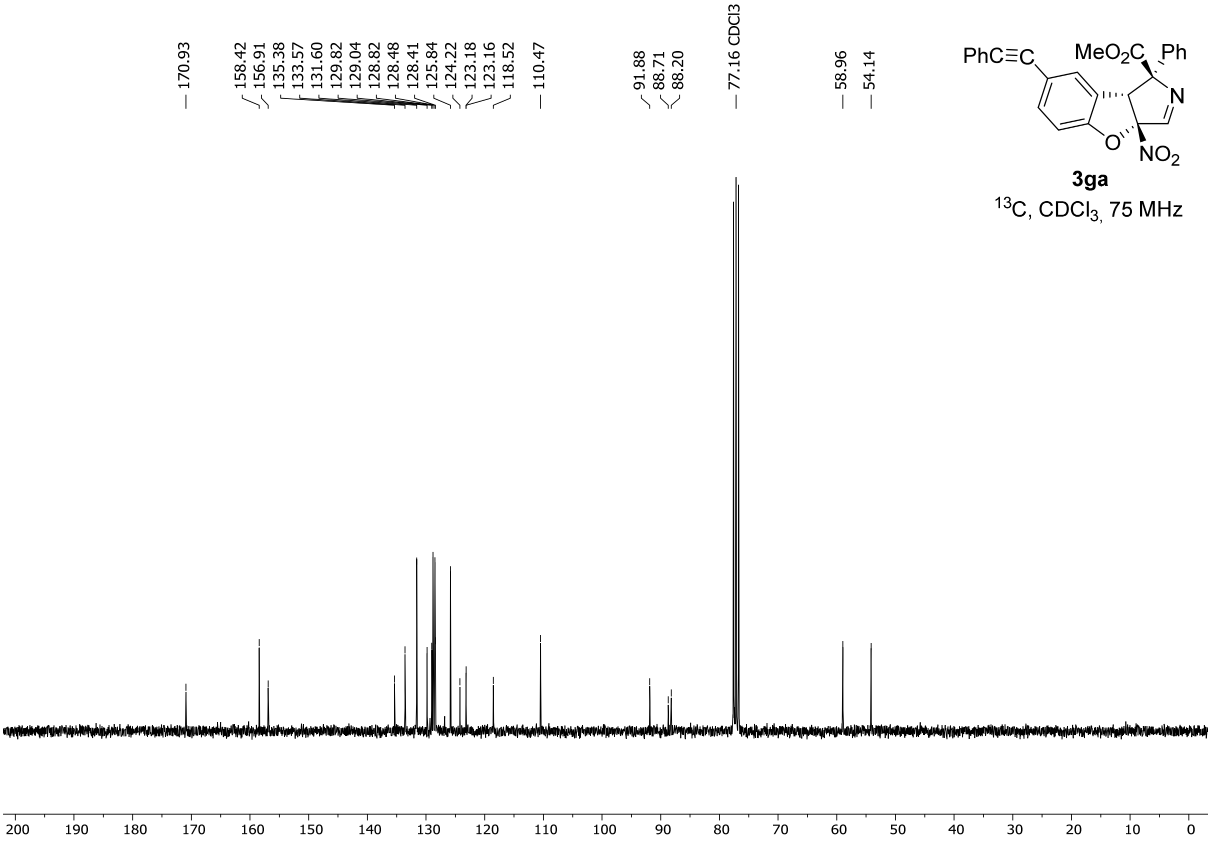

3ga

^{13}C, $CDCl_3$, 75 MHz

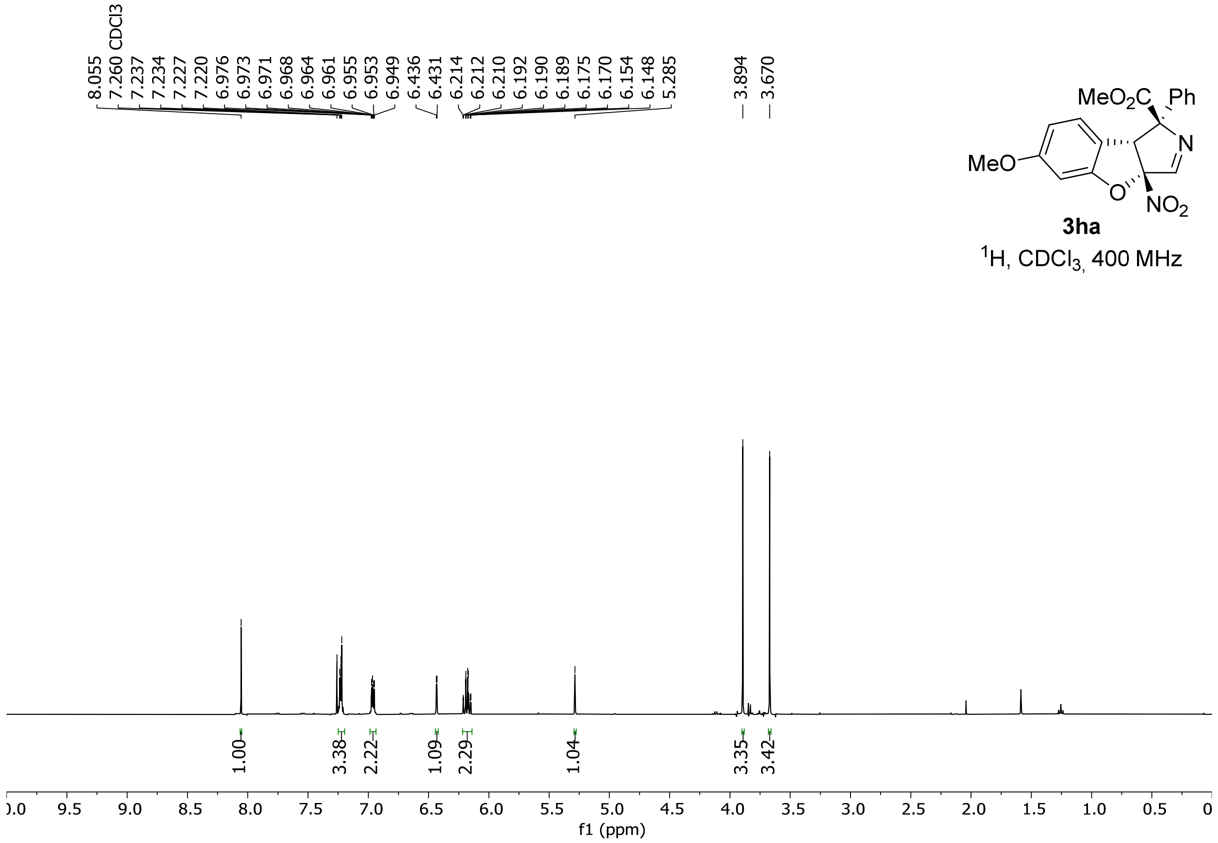
8.055
7.260 CDCl3
7.237
7.234
7.227
7.220
6.976
6.973
6.971
6.968
6.964
6.961
6.955
6.953
6.949
6.436
6.431
6.214
6.212
6.210
6.192
6.190
6.189
6.175
6.170
6.154
6.148
5.285
3.894
3.670
MeO$_2$C
Ph
N
MeO
O
NO$_2$
3ha
^{1}H, CDCl$_3$, 400 MHz
1.00
3.38
2.22
1.09
2.29
1.04
3.35
3.42
f1 (ppm)

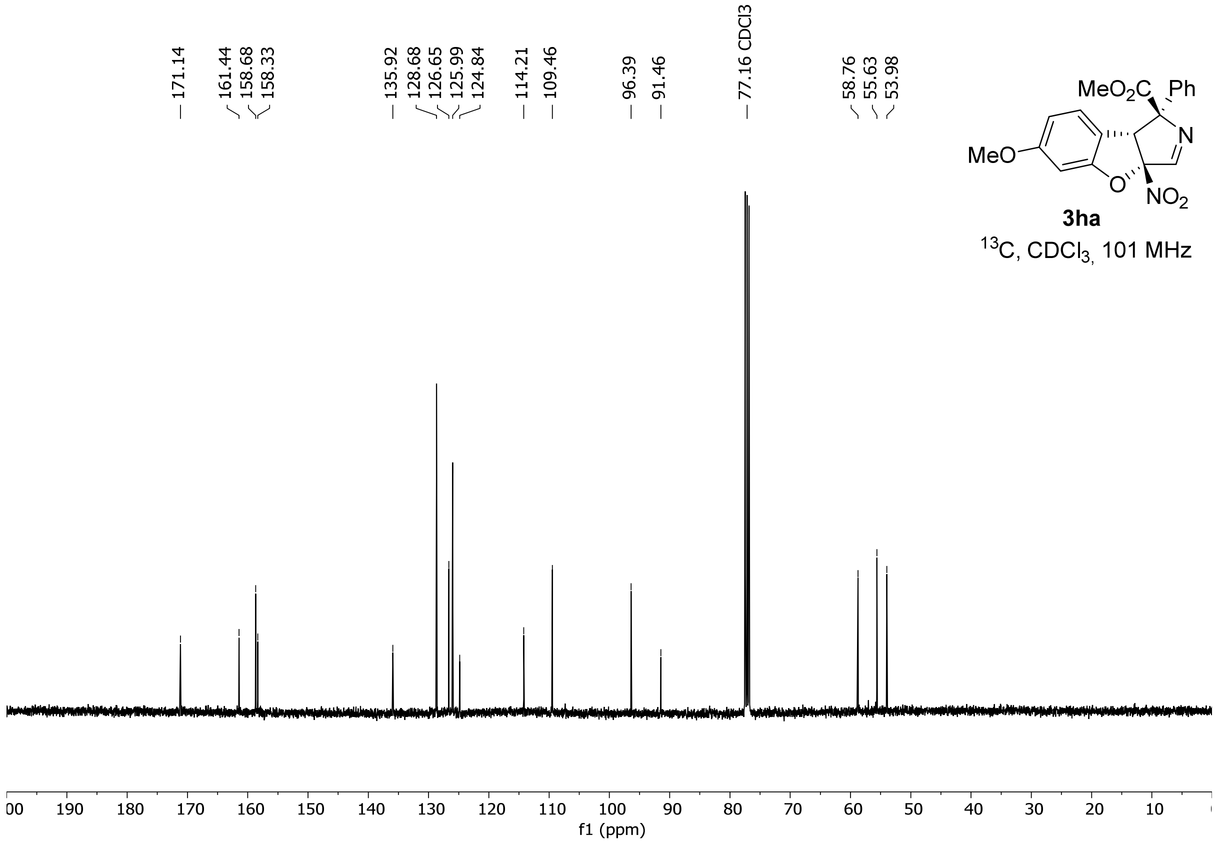
171.14
161.44
158.68
158.33
135.92
128.68
126.65
125.99
124.84
114.21
109.46
96.39
91.46
77.16 CDCl3
58.76
55.63
53.98
MeO$_2$C
Ph
N
MeO
O
NO$_2$
3ha
^{13}C, CDCl$_3$, 101 MHz
f1 (ppm)

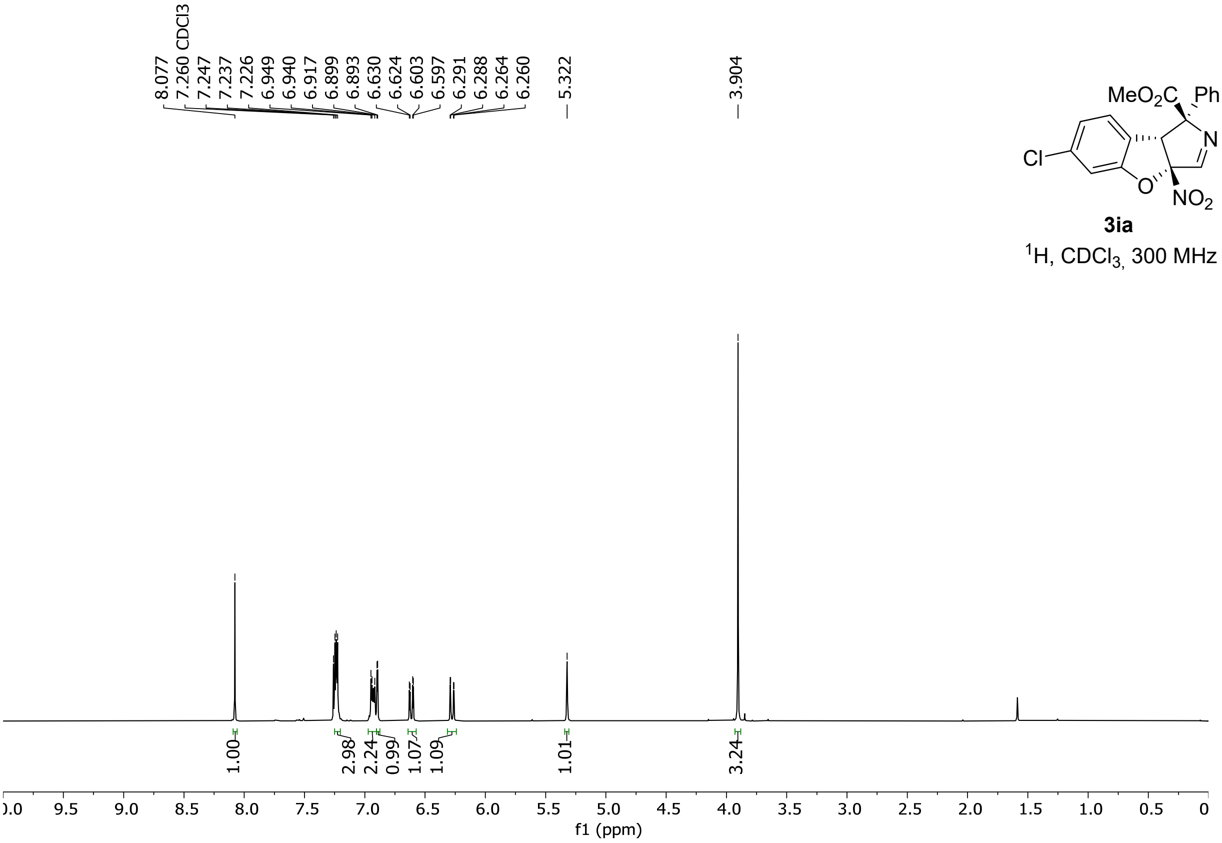

3ia

^{1}H, $CDCl_3$, 300 MHz

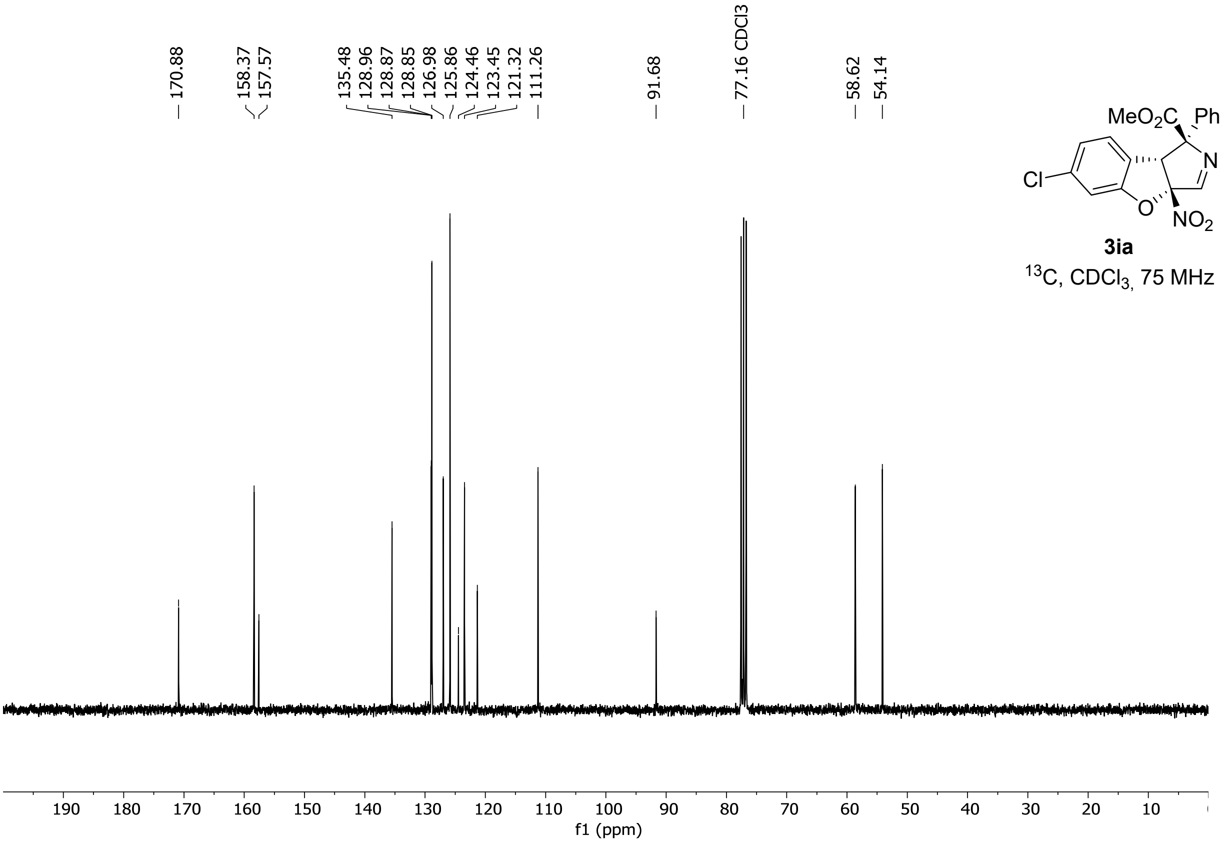

3ia

^{13}C, $CDCl_3$, 75 MHz

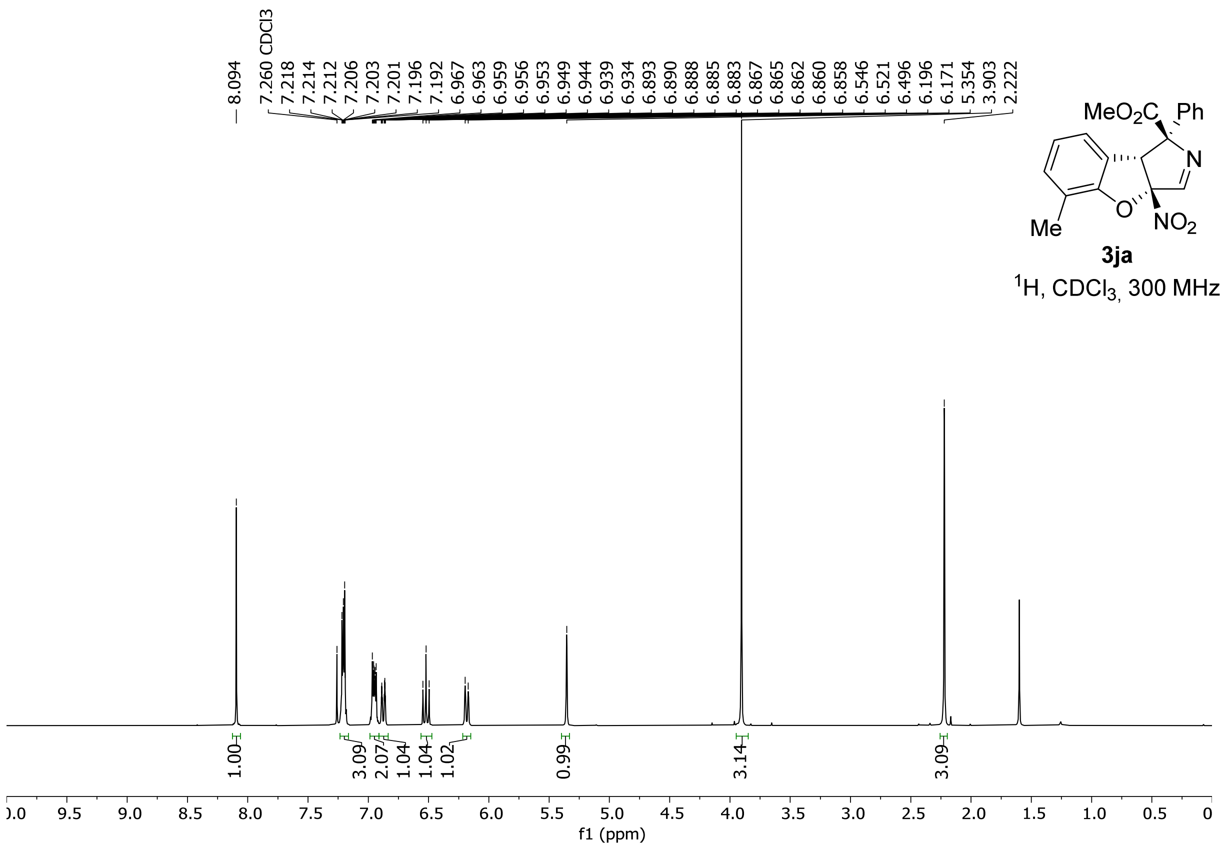

8.094
7.260 CDCl3
7.218
7.214
7.212
7.206
7.203
7.201
7.196
7.192
6.967
6.963
6.959
6.956
6.953
6.949
6.944
6.939
6.934
6.893
6.890
6.888
6.885
6.883
6.867
6.865
6.862
6.860
6.858
6.546
6.521
6.496
6.196
6.171
5.354
3.903
2.222
MeO2C
Ph
N
O
NO2
Me
3ja
1H, CDCl3, 300 MHz
1.00
3.09
2.07
1.04
1.04
1.02
0.99
3.14
3.09
f1 (ppm)

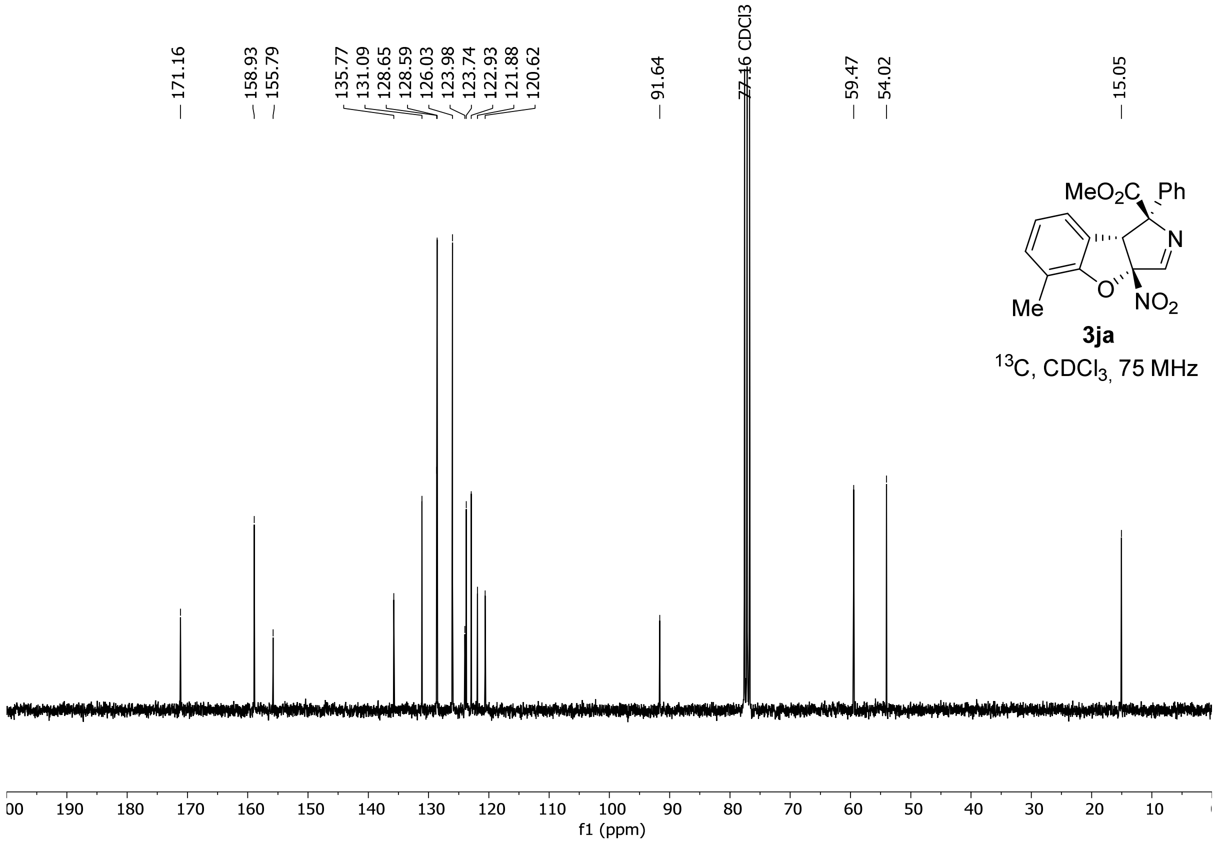

171.16
158.93
155.79
135.77
131.09
128.65
128.59
126.03
123.98
123.74
122.93
121.88
120.62
91.64
77.16 CDCl3
59.47
54.02
15.05
MeO2C
Ph
N
O
NO2
Me
3ja
13C, CDCl3, 75 MHz
f1 (ppm)

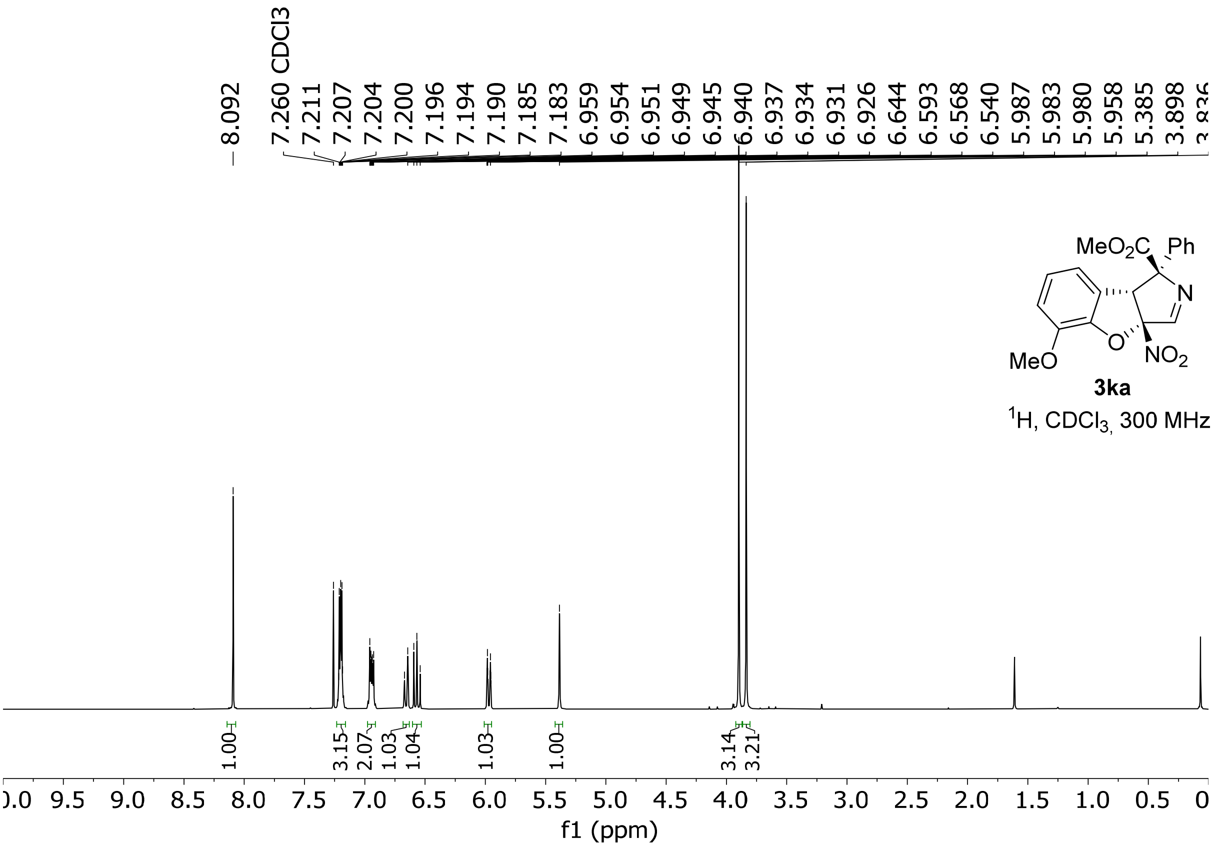
8.092
7.260 CDCl3
7.211
7.207
7.204
7.200
7.196
7.194
7.190
7.185
7.183
6.959
6.954
6.951
6.949
6.945
6.940
6.937
6.934
6.931
6.926
6.644
6.593
6.568
6.540
5.987
5.983
5.980
5.958
5.385
3.898
MeO2C
Ph
N
MeO
O
NO2
3ka
1H, CDCl3, 300 MHz
1.00
3.15
2.07
1.03
1.04
1.03
1.00
3.14
3.21
f1 (ppm)

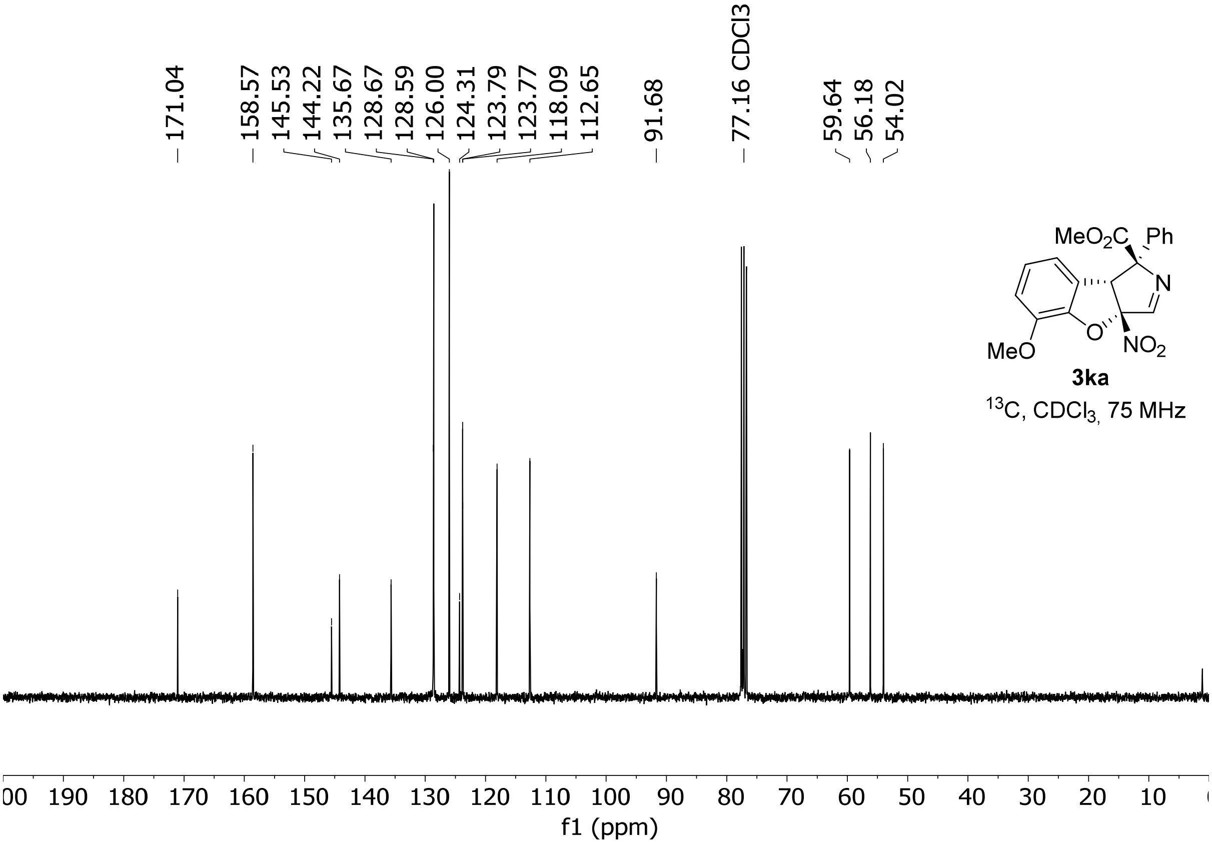
171.04
158.57
145.53
144.22
135.67
128.67
128.59
126.00
124.31
123.79
123.77
118.09
112.65
91.68
77.16 CDCl3
59.64
56.18
54.02
MeO2C
Ph
N
MeO
O
NO2
3ka
13C, CDCl3, 75 MHz
f1 (ppm)

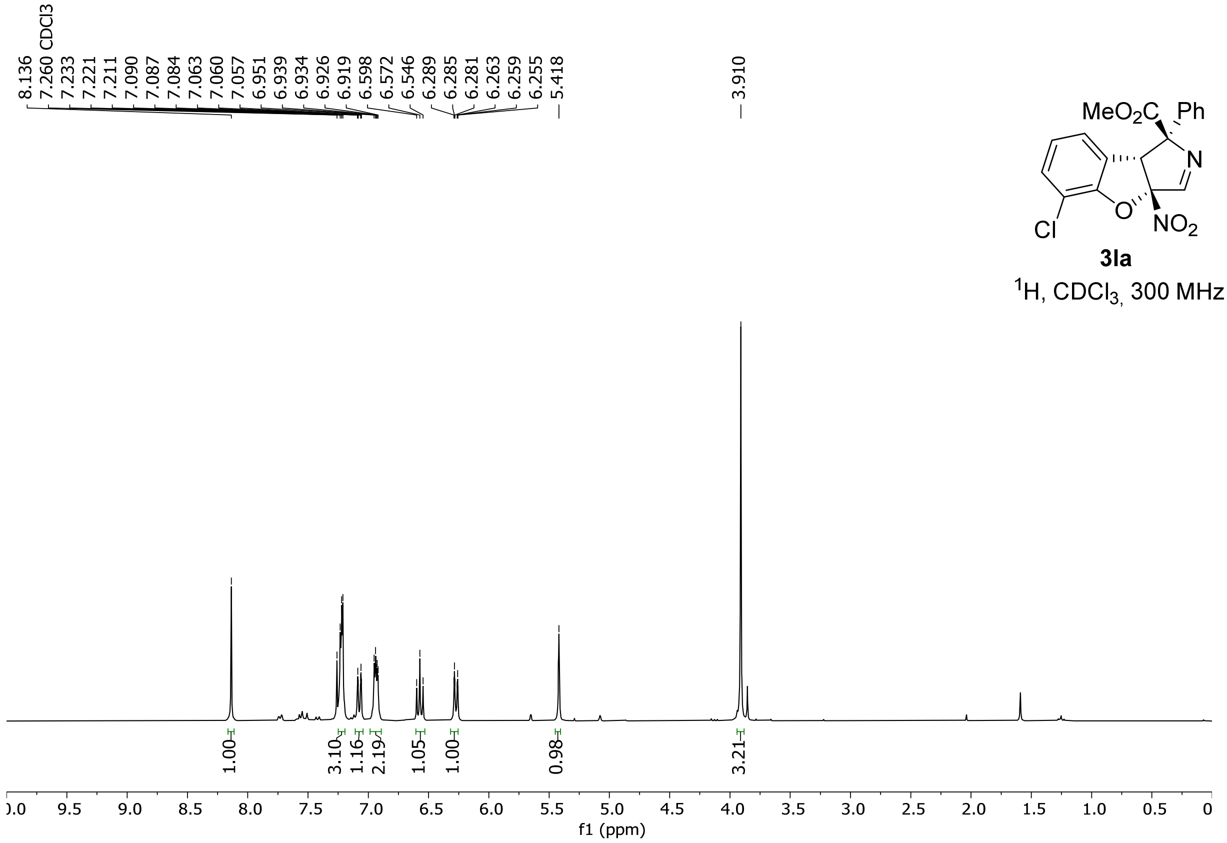

3la

^{1}H, $CDCl_3$, 300 MHz

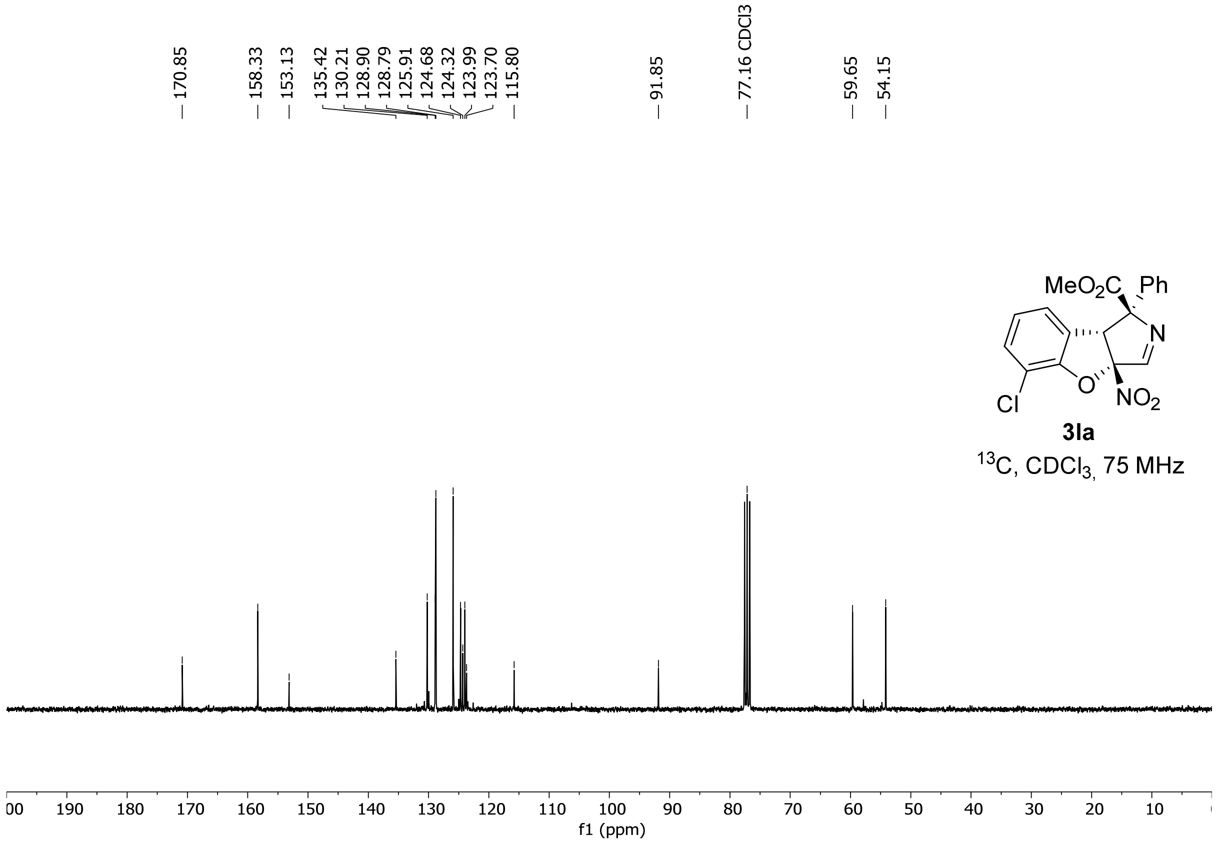

3la

^{13}C, $CDCl_3$, 75 MHz

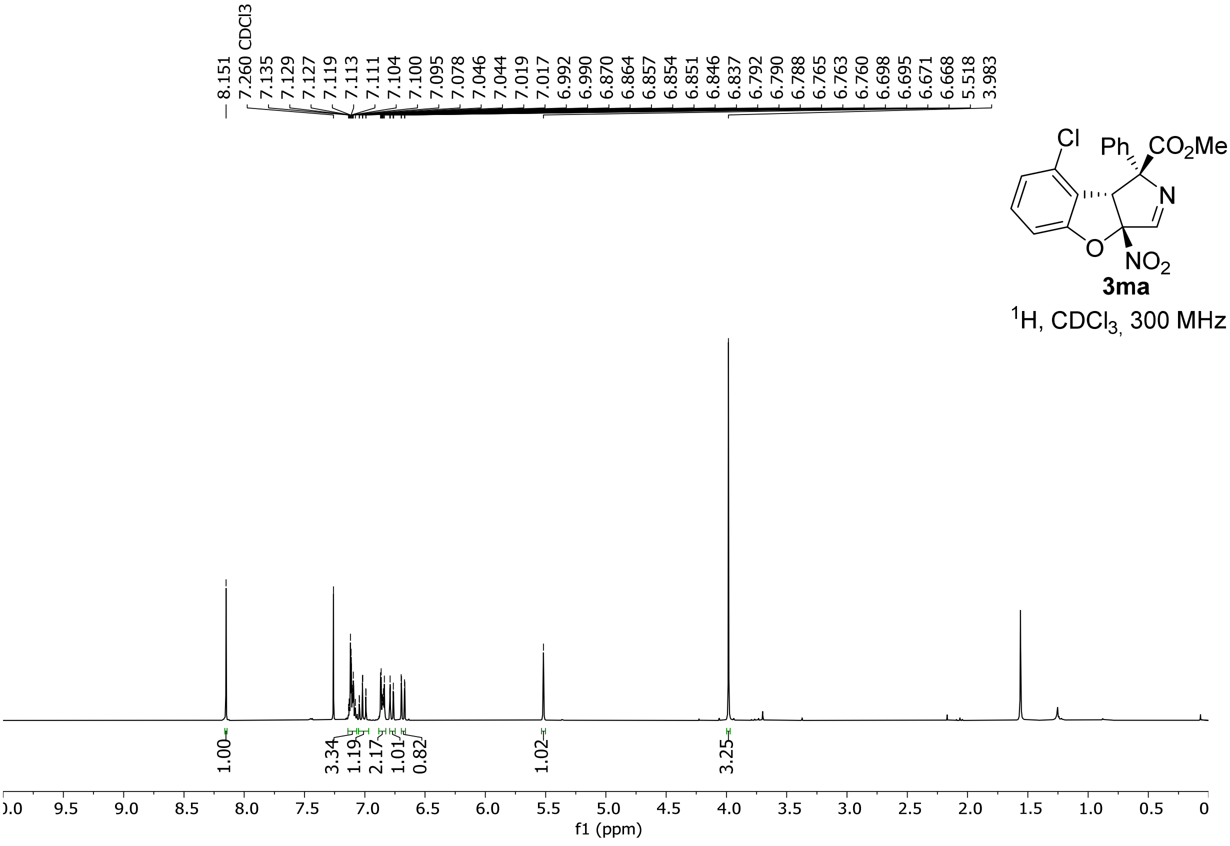
8.151
7.260 CDCl3
7.135
7.129
7.127
7.119
7.113
7.111
7.104
7.100
7.095
7.078
7.046
7.044
7.019
7.017
6.992
6.990
6.870
6.864
6.857
6.854
6.851
6.846
6.837
6.792
6.790
6.788
6.765
6.763
6.760
6.698
6.695
6.671
6.668
5.518
3.983
Cl
Ph
CO2Me
N
O
NO2
3ma
1H, CDCl3, 300 MHz
1.00
3.34
1.19
2.17
1.01
0.82
1.02
3.25
f1 (ppm)

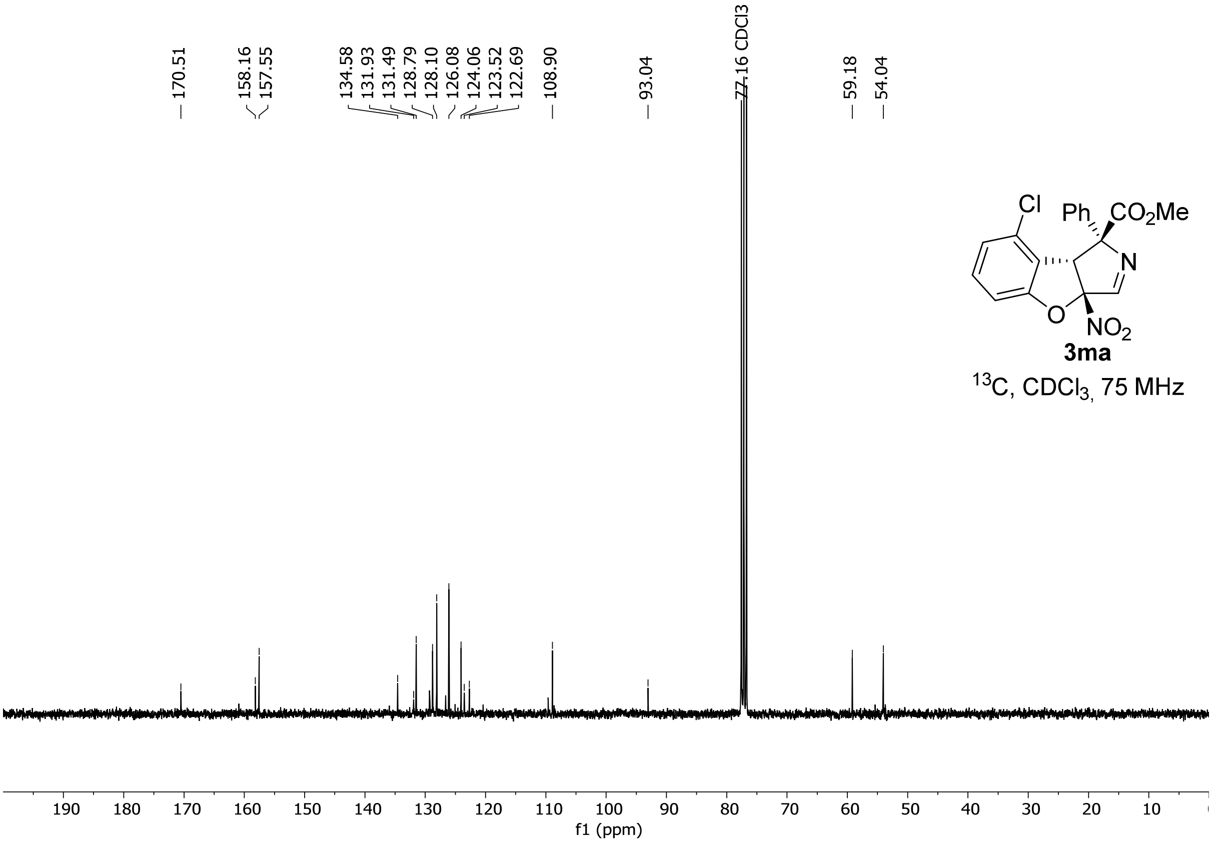
170.51
158.16
157.55
134.58
131.93
131.49
128.79
128.10
126.08
124.06
123.52
122.69
108.90
93.04
77.16 CDCl3
59.18
54.04
Cl
Ph
CO2Me
N
O
NO2
3ma
13C, CDCl3, 75 MHz
f1 (ppm)

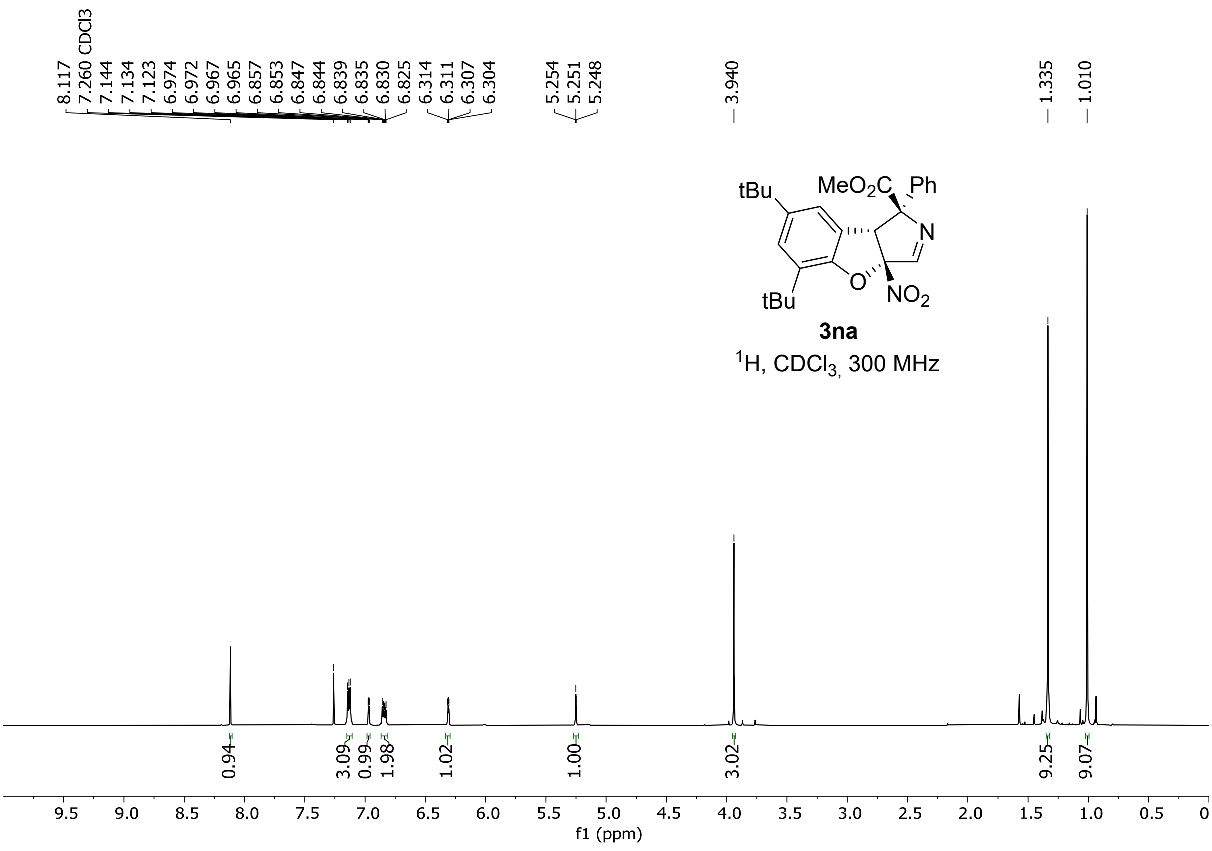

3na

^{1}H, $CDCl_3$, 300 MHz

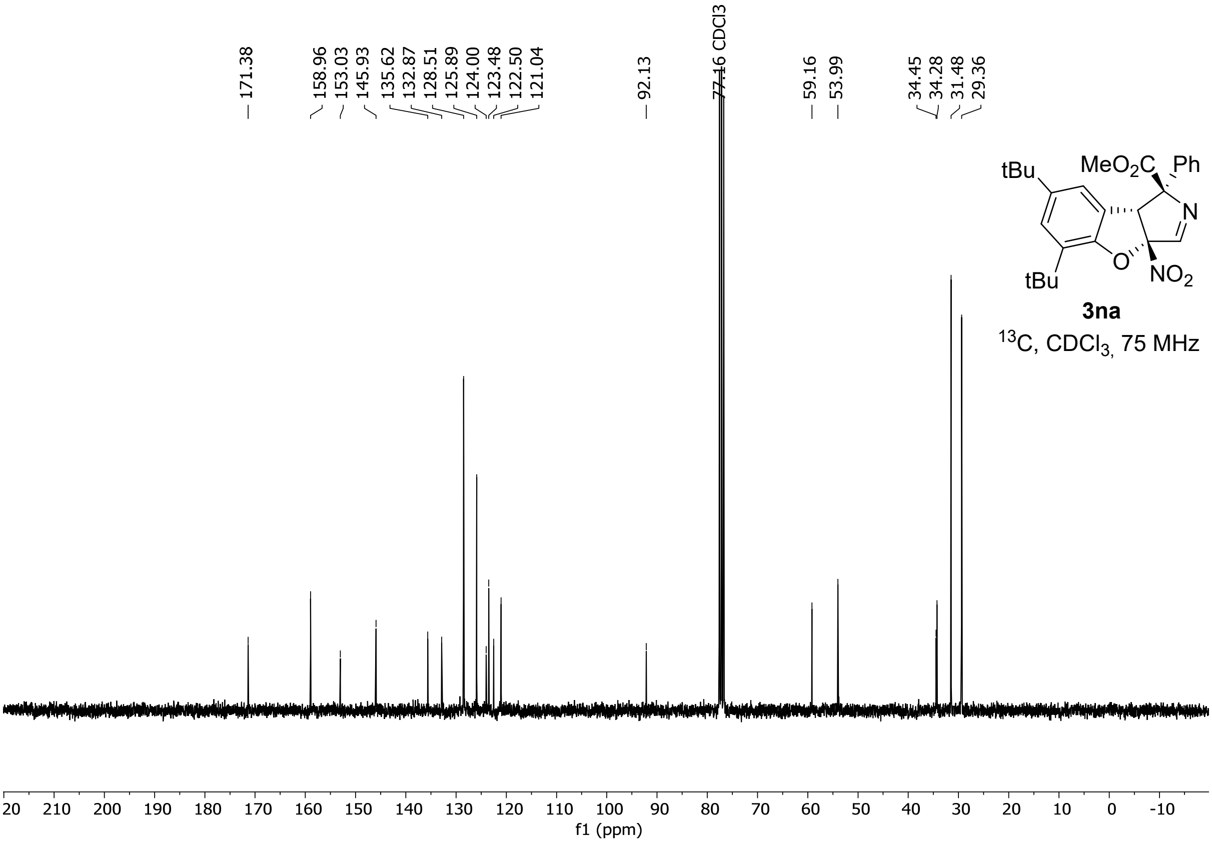

3na

^{13}C, $CDCl_3$, 75 MHz

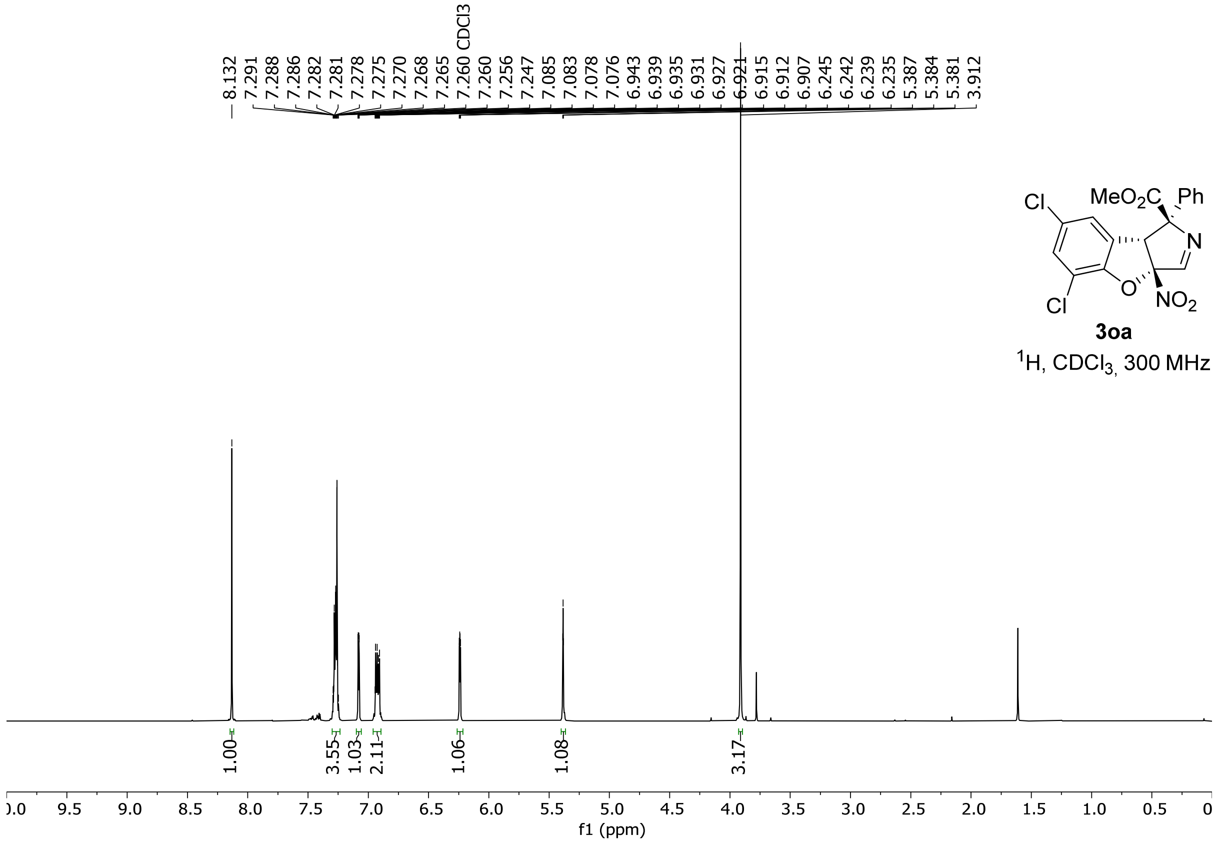
8.132
7.291
7.288
7.286
7.282
7.281
7.278
7.275
7.270
7.268
7.265
7.260 CDCl3
7.260
7.256
7.247
7.085
7.083
7.078
7.076
6.943
6.939
6.935
6.931
6.927
6.921
6.915
6.912
6.907
6.245
6.242
6.239
6.235
5.387
5.384
5.381
3.912
Cl
MeO2C
Ph
N
O
NO2
Cl
3oa
1H, CDCl3, 300 MHz
1.00
3.55
1.03
2.11
1.06
1.08
3.17
f1 (ppm)

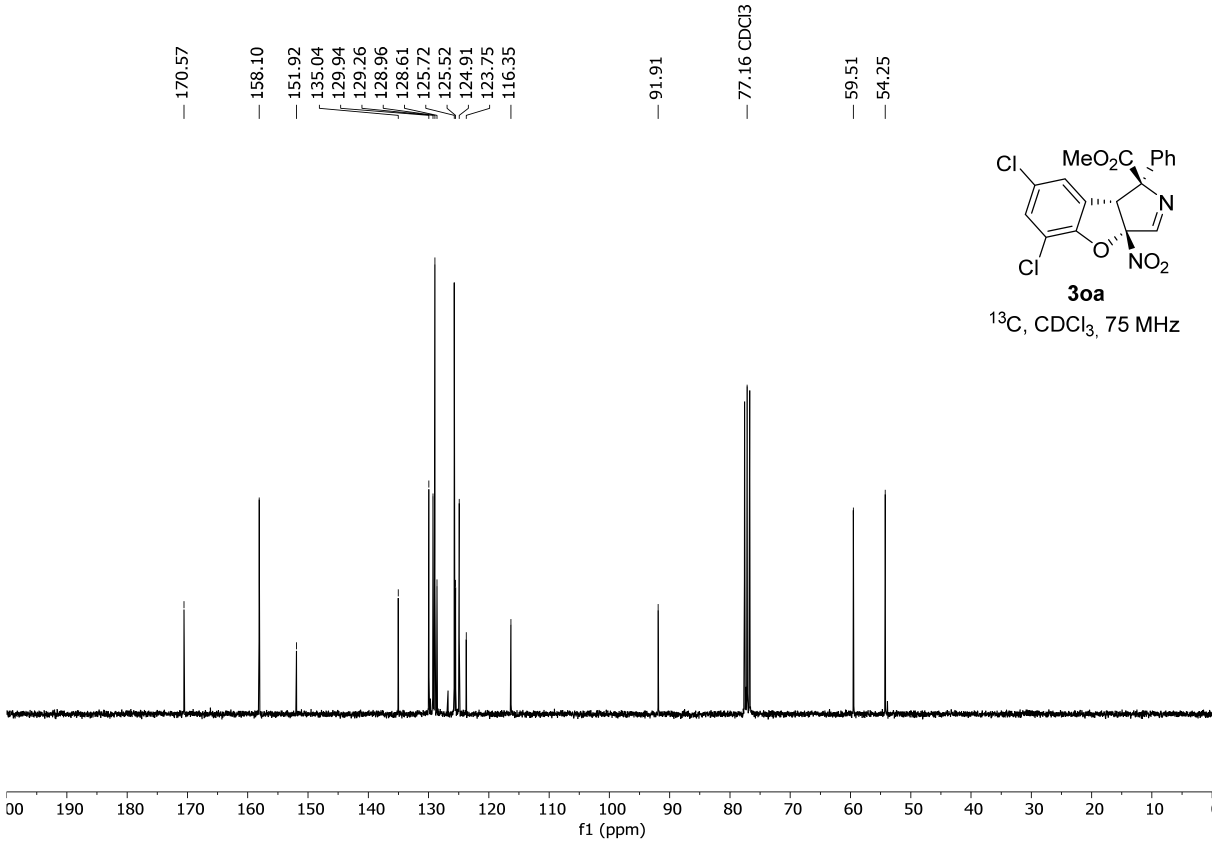
170.57
158.10
151.92
135.04
129.94
129.26
128.96
128.61
125.72
125.52
124.91
123.75
116.35
91.91
77.16 CDCl3
59.51
54.25
Cl
MeO2C
Ph
N
O
NO2
Cl
3oa
13C, CDCl3, 75 MHz
f1 (ppm)

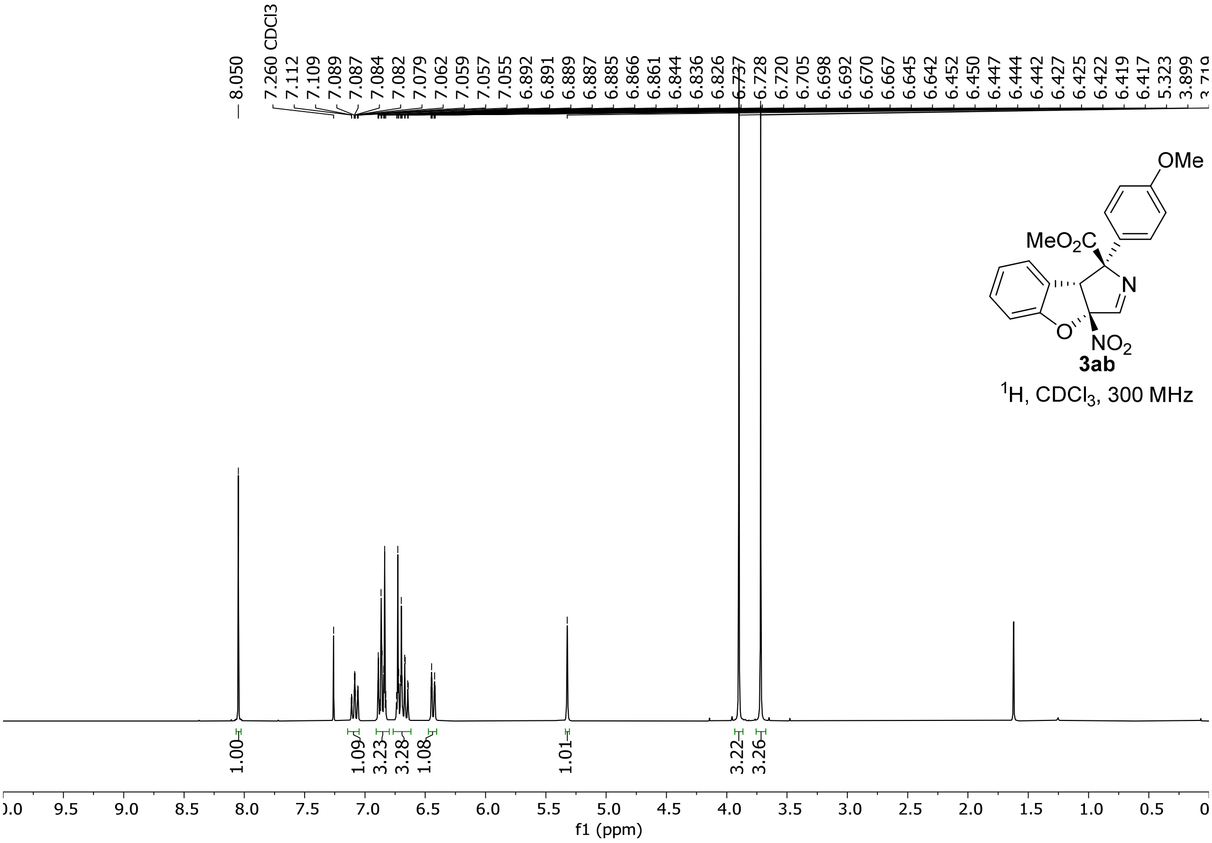

8.050
7.260 CDCl3
7.112
7.109
7.089
7.087
7.084
7.082
7.079
7.062
7.059
7.057
7.055
6.892
6.891
6.889
6.887
6.885
6.866
6.861
6.844
6.836
6.826
6.737
6.728
6.720
6.705
6.698
6.692
6.670
6.667
6.645
6.642
6.452
6.450
6.447
6.444
6.442
6.427
6.425
6.422
6.419
6.417
5.323
3.899
OMe
MeO2C
N
O
NO2
3ab
1H, CDCl3, 300 MHz
1.00
1.09
3.23
3.28
1.08
1.01
3.22
3.26
9.5
9.0
8.5
8.0
7.5
7.0
6.5
6.0
5.5
5.0
4.5
4.0
3.5
3.0
2.5
2.0
1.5
1.0
0.5
f1 (ppm)

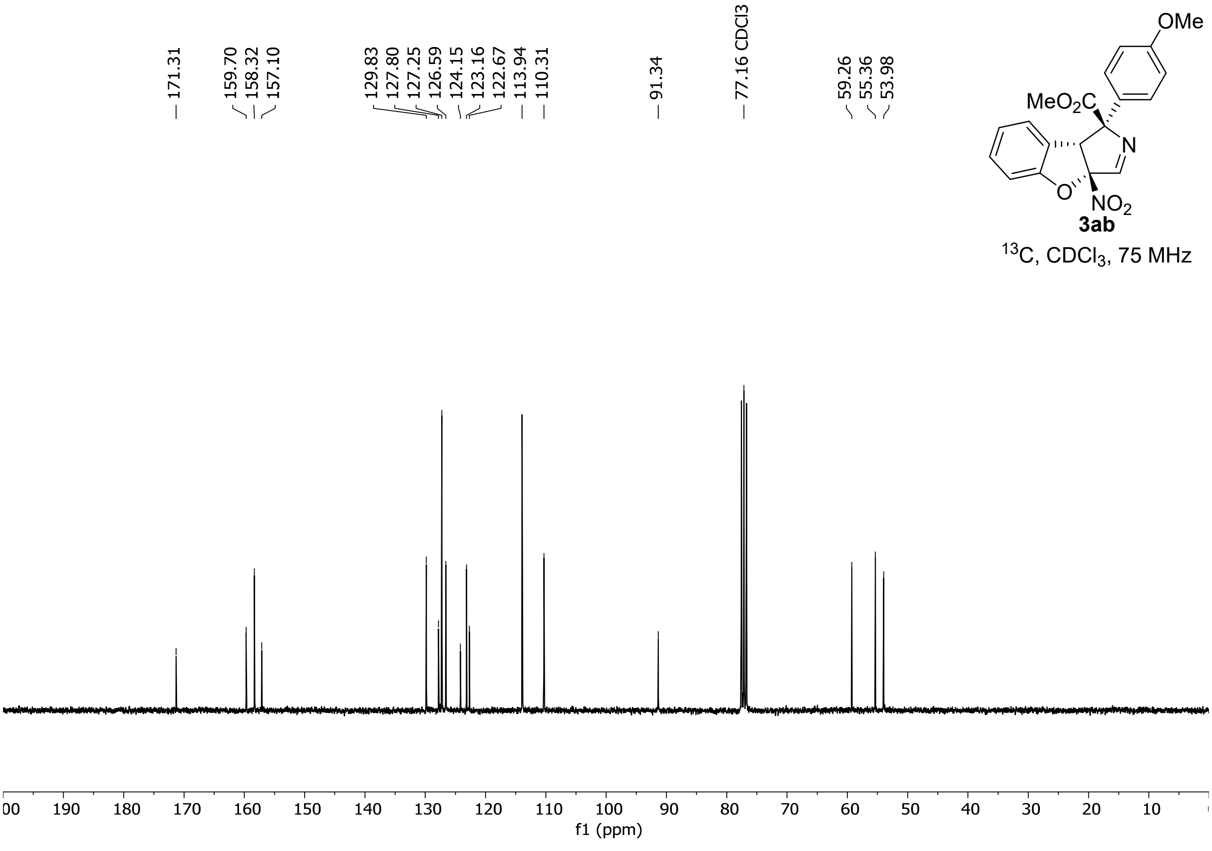

171.31
159.70
158.32
157.10
129.83
127.80
127.25
126.59
124.15
123.16
122.67
113.94
110.31
91.34
77.16 CDCl3
59.26
55.36
53.98
OMe
MeO2C
N
O
NO2
3ab
13C, CDCl3, 75 MHz
190
180
170
160
150
140
130
120
110
100
90
80
70
60
50
40
30
20
10
f1 (ppm)

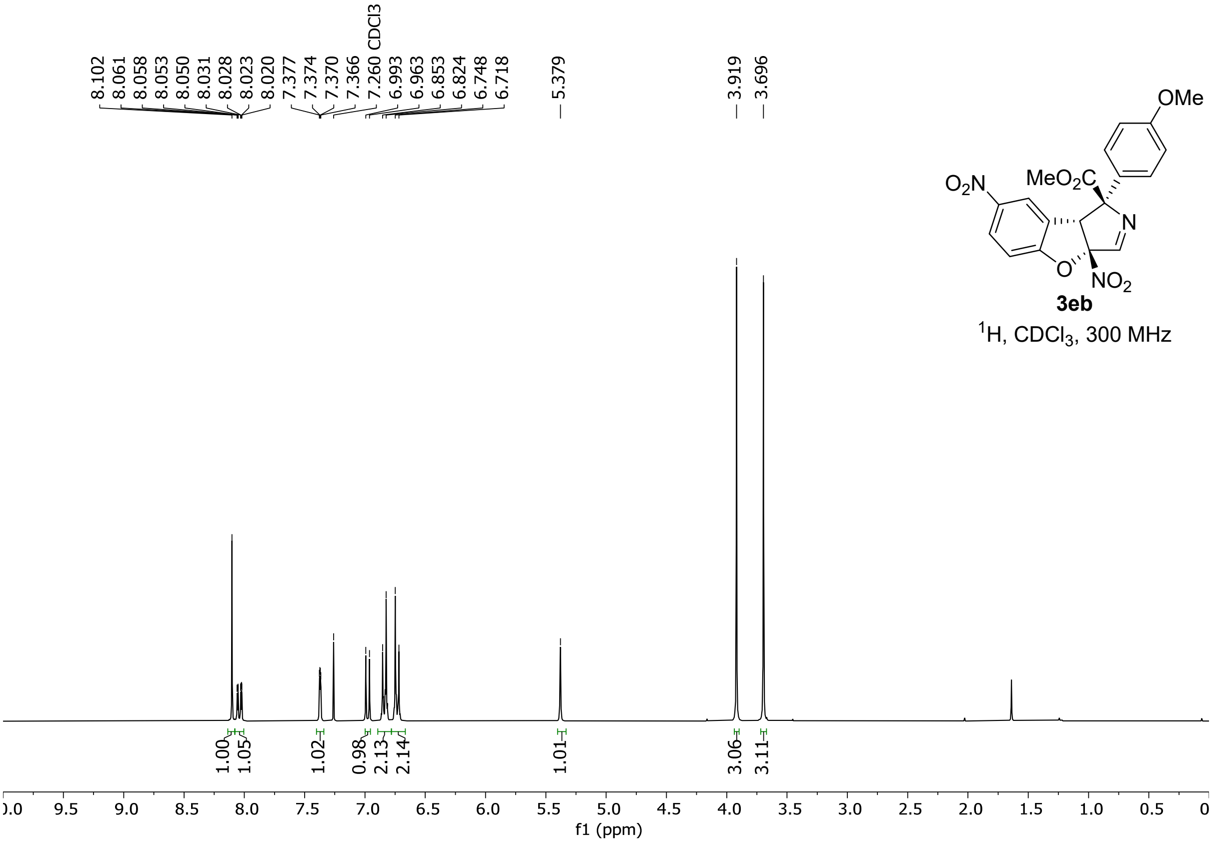

3eb

^1H, $CDCl_3$, 300 MHz

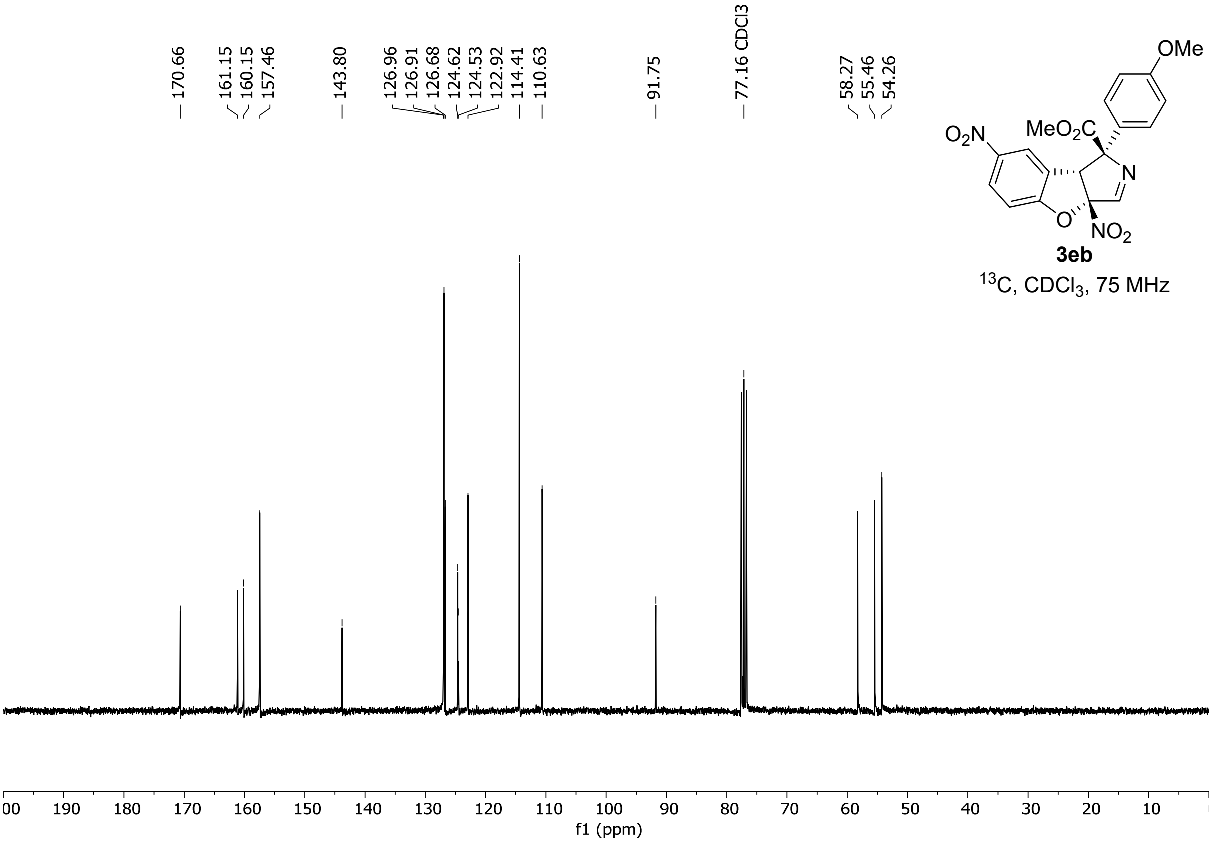

3eb

^{13}C, $CDCl_3$, 75 MHz

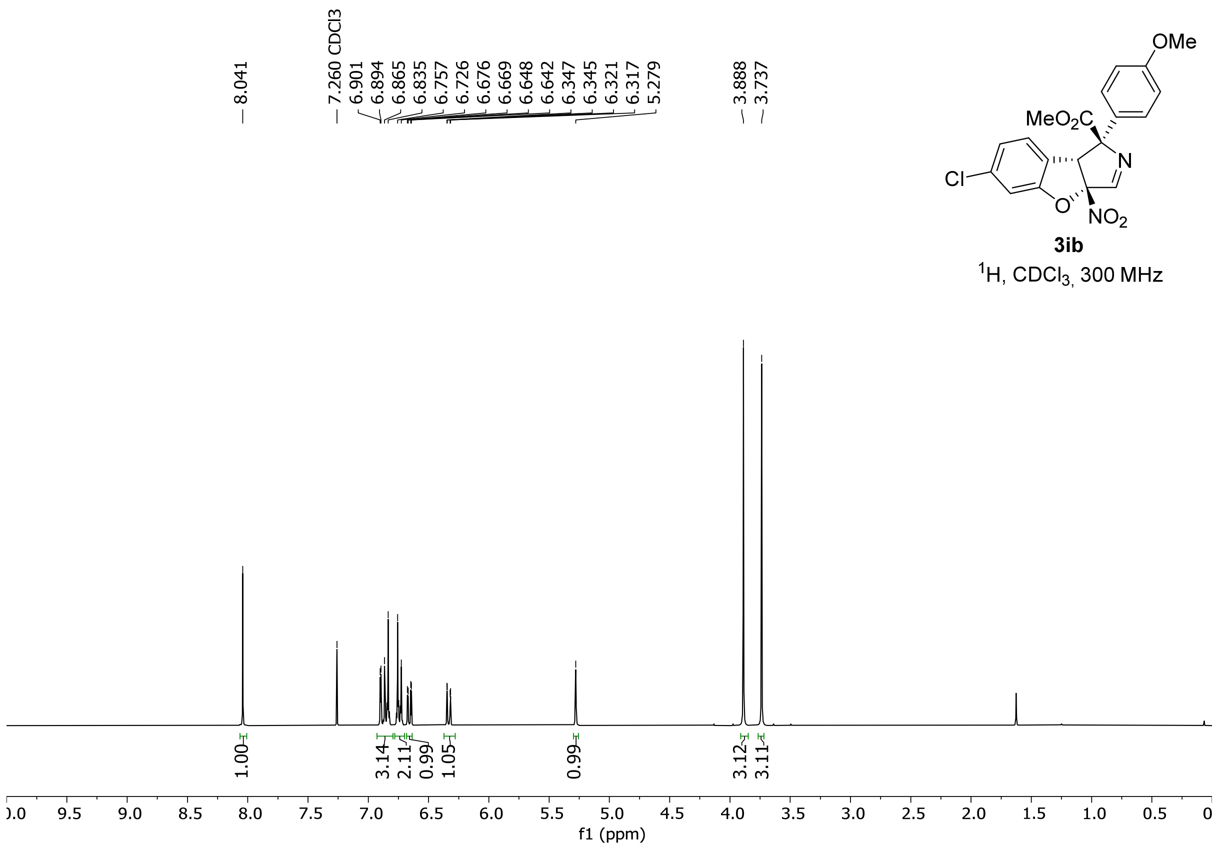

3ib
^1H, $CDCl_3$, 300 MHz

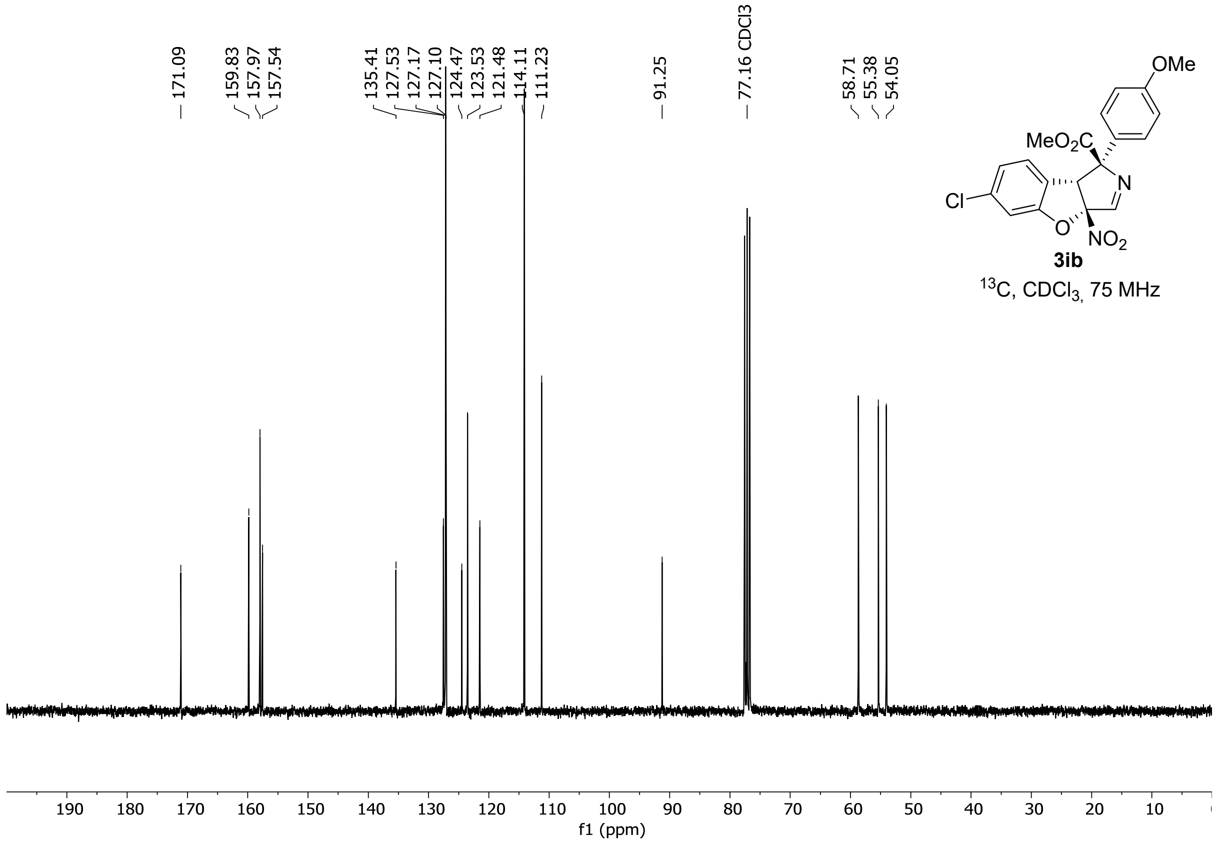

3ib
^{13}C, $CDCl_3$, 75 MHz

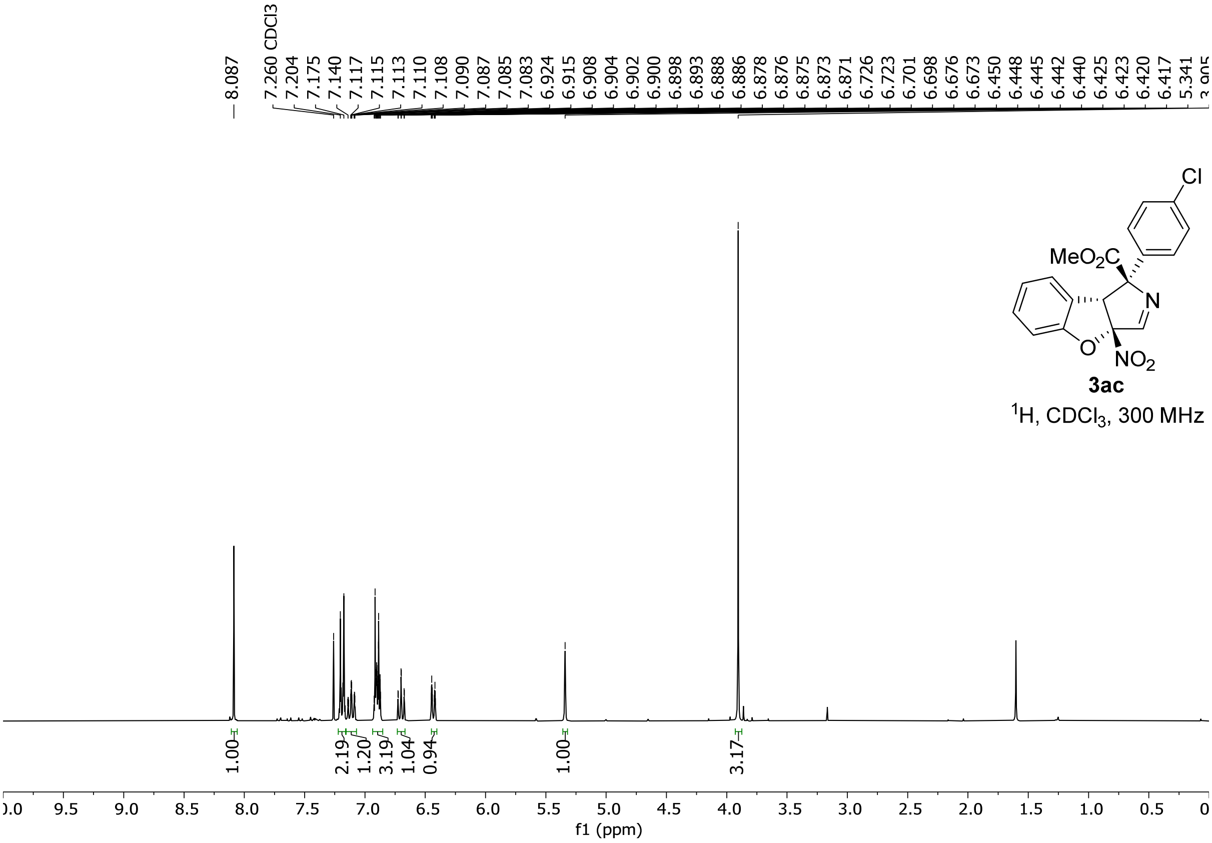
8.087
7.260 CDCl3
7.204
7.175
7.140
7.117
7.115
7.113
7.110
7.108
7.090
7.087
7.085
7.083
6.924
6.915
6.908
6.904
6.902
6.900
6.898
6.893
6.888
6.886
6.878
6.876
6.875
6.873
6.871
6.726
6.723
6.701
6.698
6.676
6.673
6.450
6.448
6.445
6.442
6.440
6.425
6.423
6.420
6.417
5.341
Cl
MeO2C
N
O
NO2
3ac
1H, CDCl3, 300 MHz
1.00
2.19
1.20
3.19
1.04
0.94
1.00
3.17
f1 (ppm)

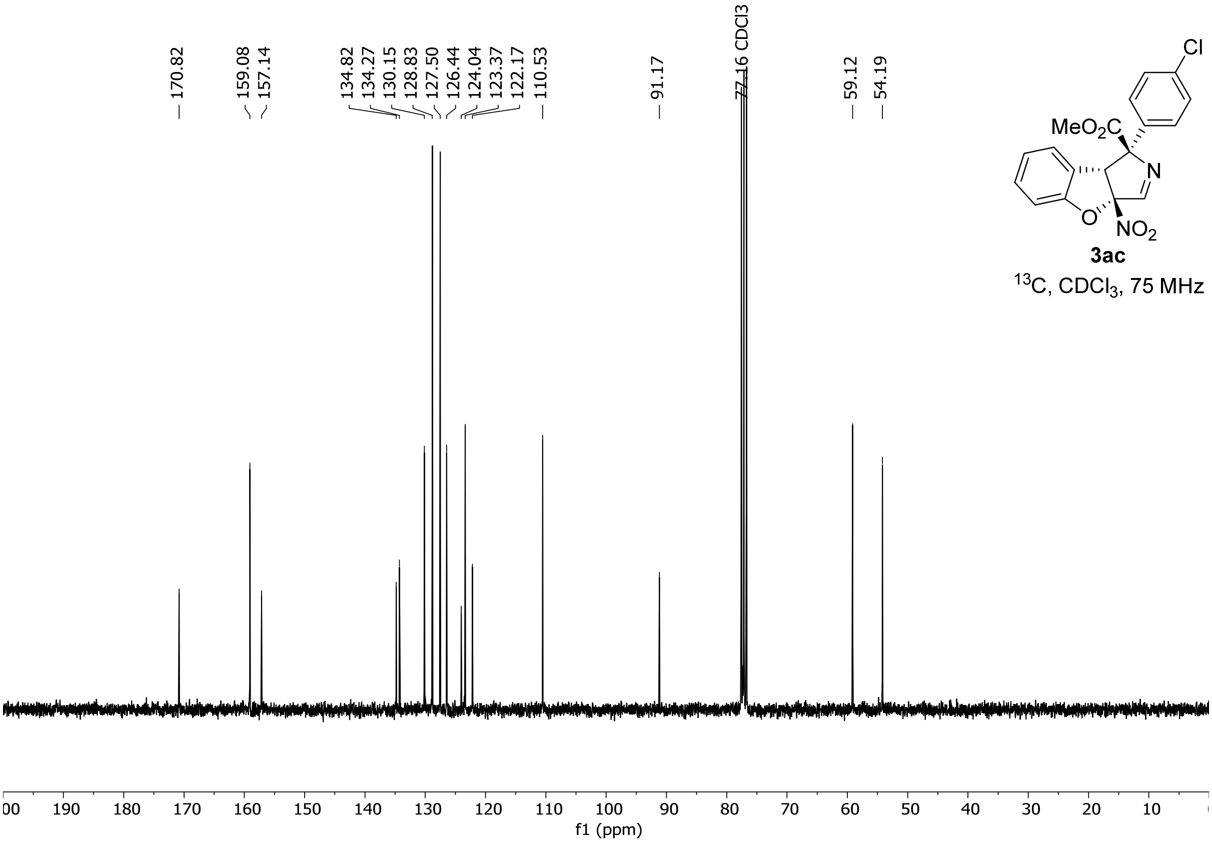
170.82
159.08
157.14
134.82
134.27
130.15
128.83
127.50
126.44
124.04
123.37
122.17
110.53
91.17
77.16 CDCl3
59.12
54.19
Cl
MeO2C
N
O
NO2
3ac
13C, CDCl3, 75 MHz
f1 (ppm)

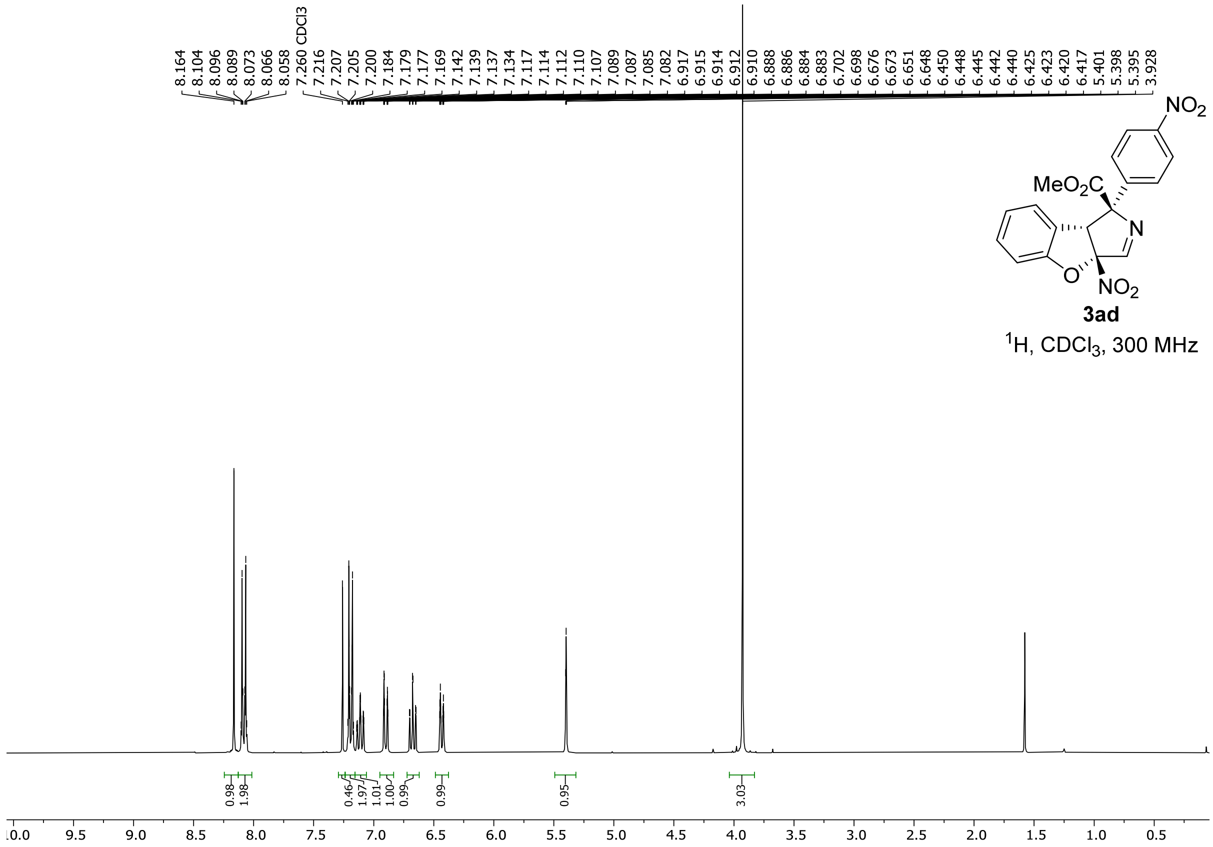
8.164
8.104
8.096
8.089
8.073
8.066
8.058
7.260 CDCl3
7.216
7.207
7.205
7.200
7.184
7.179
7.177
7.169
7.142
7.139
7.137
7.134
7.117
7.114
7.112
7.110
7.107
7.089
7.087
7.085
7.082
6.917
6.915
6.914
6.912
6.910
6.888
6.886
6.884
6.883
6.702
6.698
6.676
6.673
6.651
6.648
6.450
6.448
6.445
6.442
6.440
6.425
6.423
6.420
6.417
5.401
5.398
5.395
3.928
NO2
MeO2C
N
O
NO2
3ad
1H, CDCl3, 300 MHz
0.98
1.98
0.46
1.97
1.01
1.00
0.99
0.99
0.95
3.03
9.5
9.0
8.5
8.0
7.5
7.0
6.5
6.0
5.5
5.0
4.5
4.0
3.5
3.0
2.5
2.0
1.5
1.0
0.5

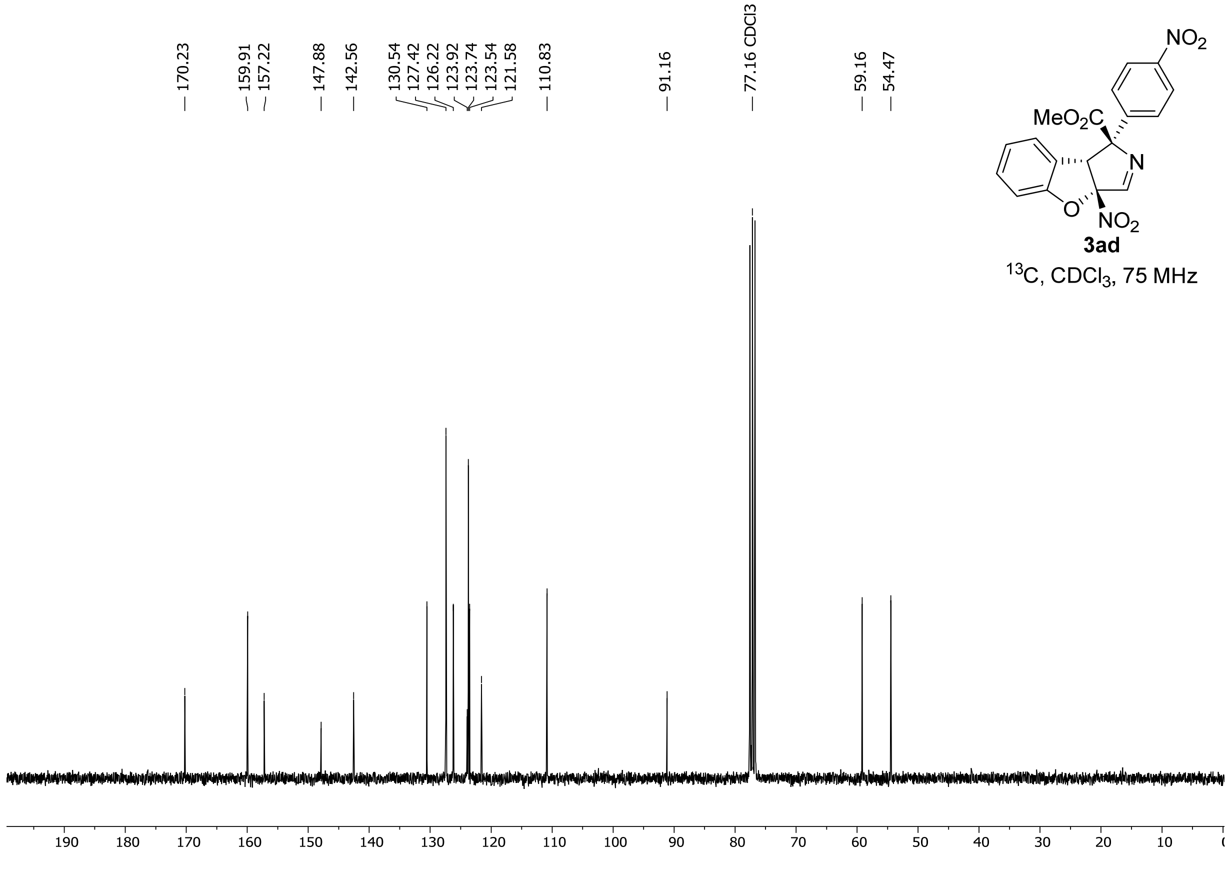
170.23
159.91
157.22
147.88
142.56
130.54
127.42
126.22
123.92
123.74
123.54
121.58
110.83
91.16
77.16 CDCl3
59.16
54.47
NO2
MeO2C
N
O
NO2
3ad
13C, CDCl3, 75 MHz
190
180
170
160
150
140
130
120
110
100
90
80
70
60
50
40
30
20
10

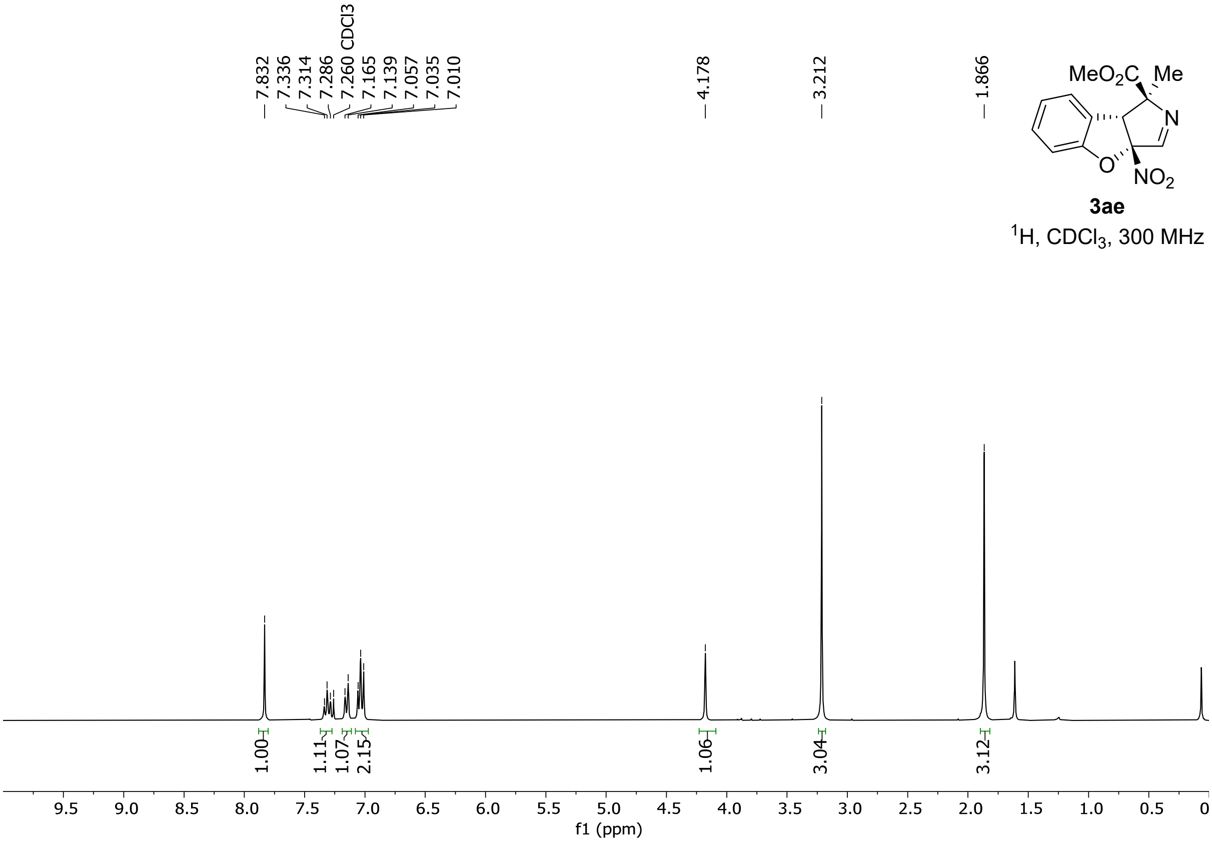

3ae
^{1}H, $CDCl_3$, 300 MHz

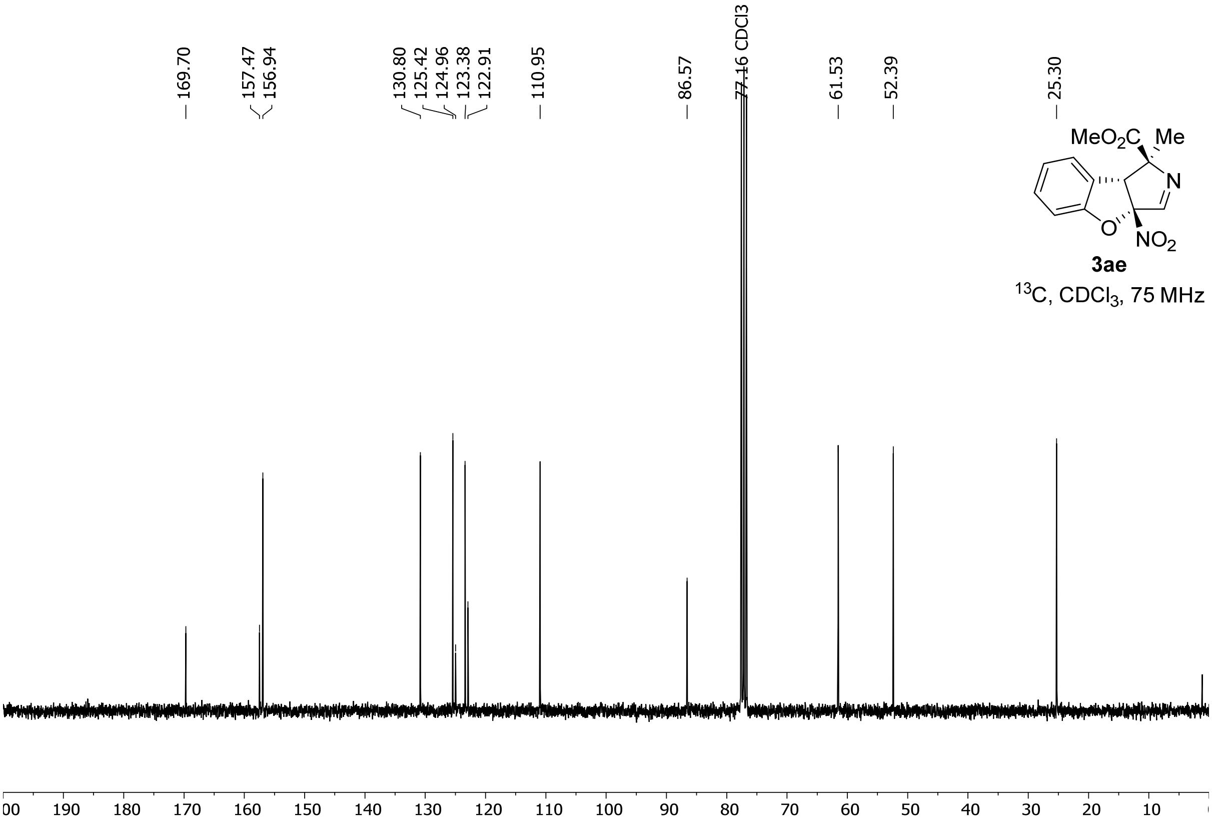

3ae
^{13}C, $CDCl_3$, 75 MHz

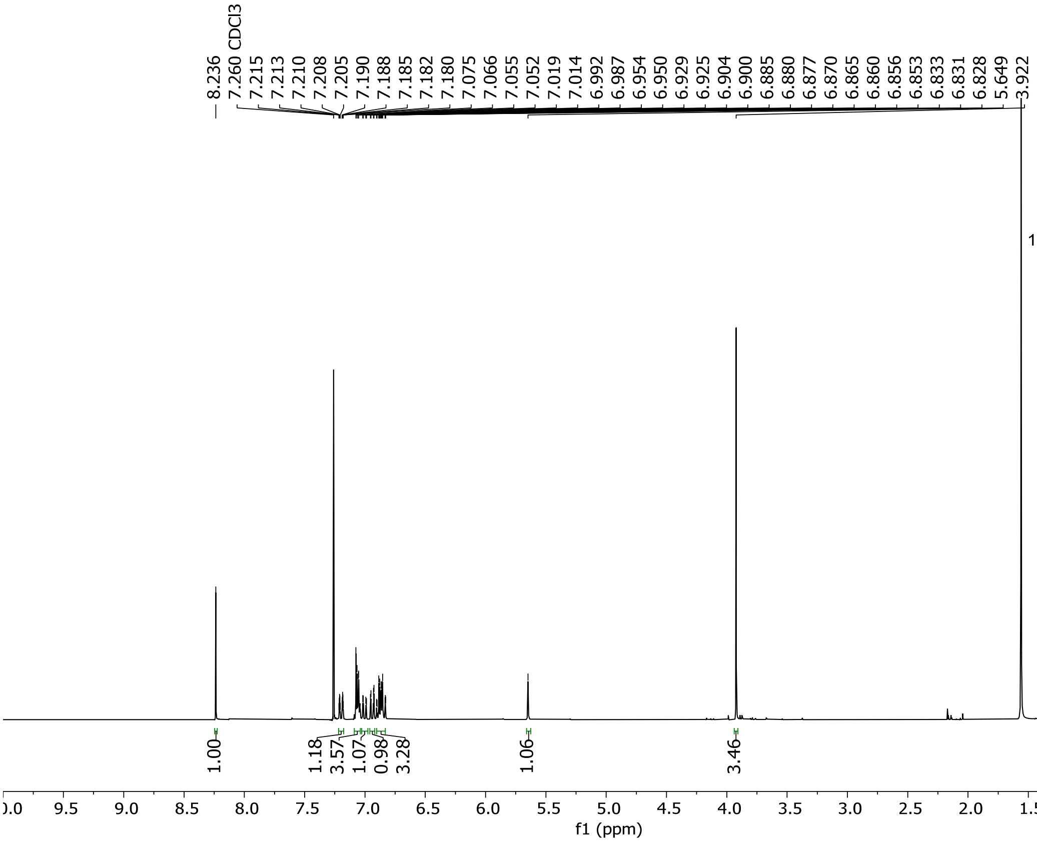

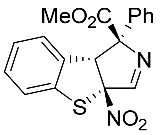

4

^{1}H, $CDCl_3$, 300 MHz

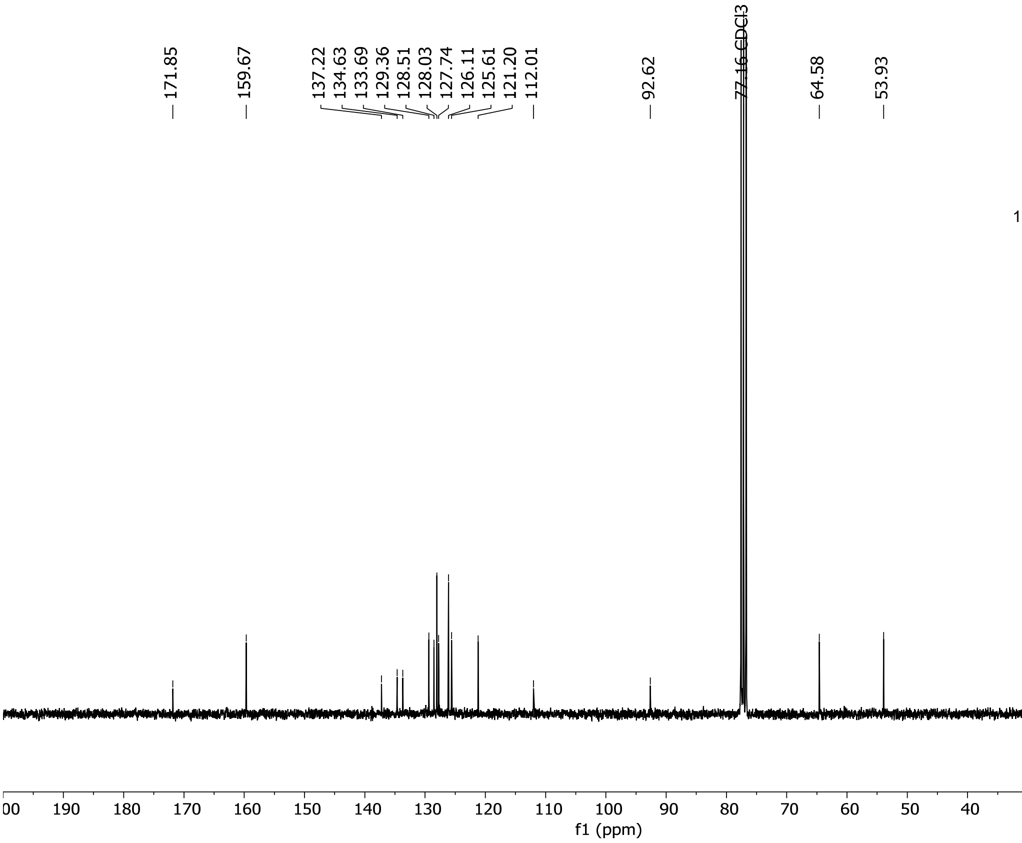

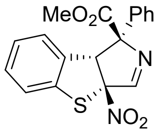

4

^{13}C, $CDCl_3$, 75 MHz

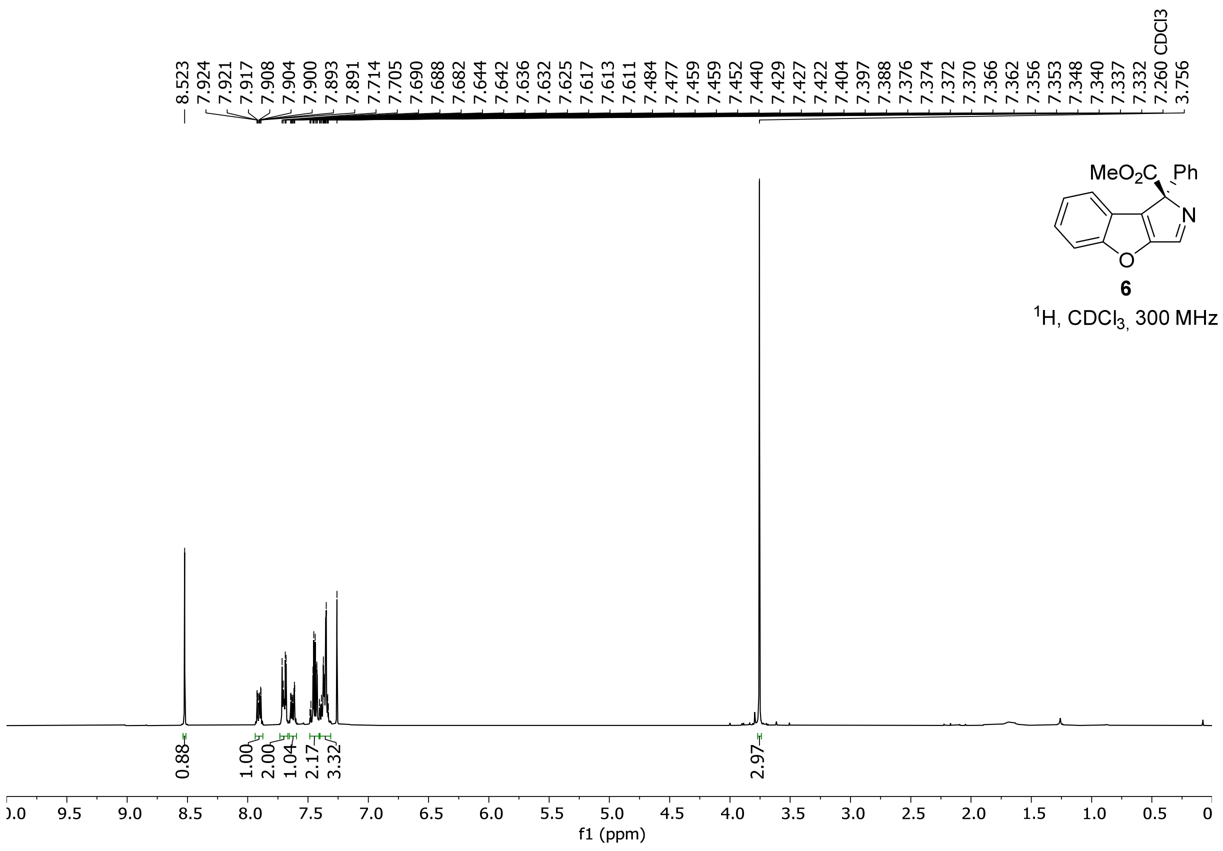
8.523 7.924 7.921 7.917 7.908 7.904 7.900 7.893 7.891 7.714 7.705 7.690 7.688 7.682 7.644 7.642 7.636 7.632 7.625 7.617 7.613 7.611 7.484 7.477 7.459 7.459 7.452 7.440 7.429 7.427 7.422 7.404 7.397 7.388 7.376 7.374 7.372 7.370 7.366 7.362 7.356 7.353 7.348 7.340 7.337 7.332 7.260 CDCl3 3.756
MeO_2C Ph
N
O
6
^1H, $CDCl_3$, 300 MHz
0.88 1.00 2.00 1.04 2.17 3.32 2.97
f1 (ppm)

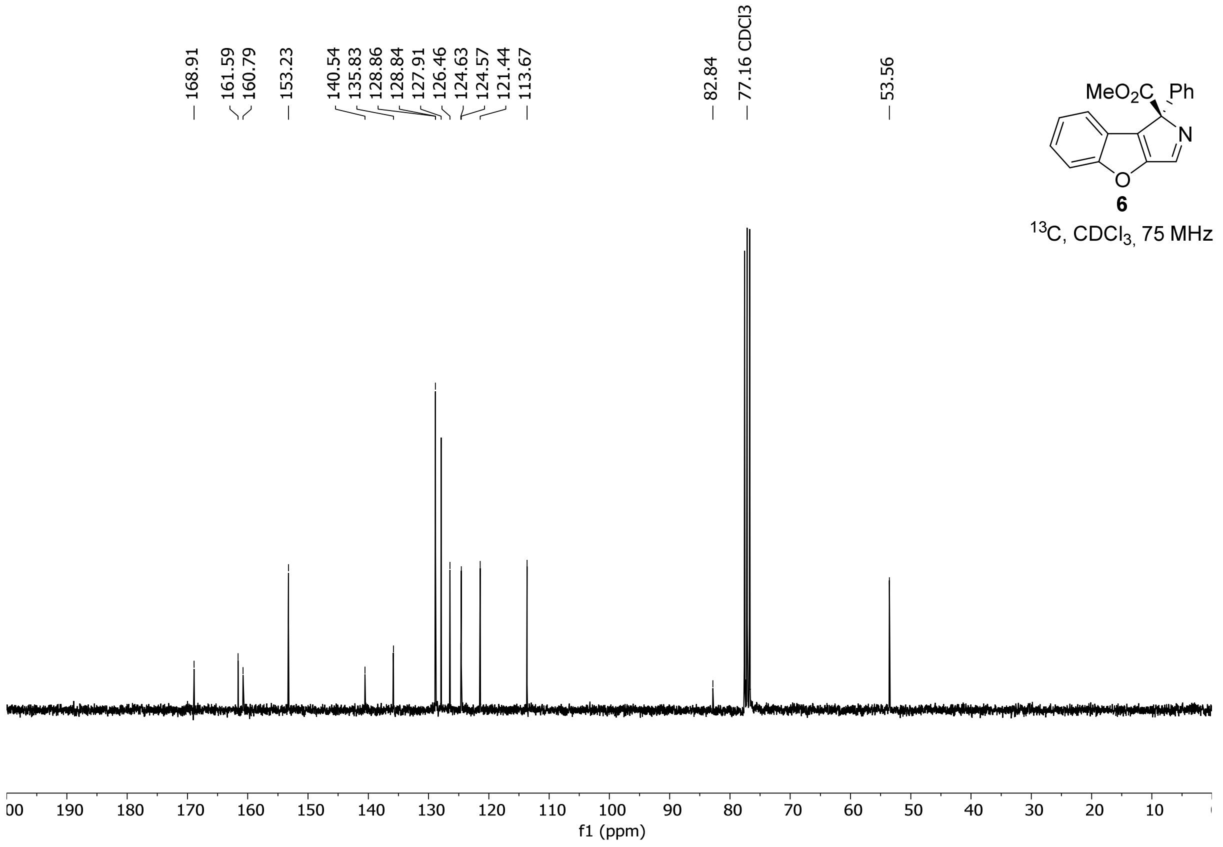
168.91 161.59 160.79 153.23 140.54 135.83 128.86 128.84 127.91 126.46 124.63 124.57 121.44 113.67 82.84 77.16 CDCl3 53.56
MeO_2C Ph
N
O
6
^{13}C, $CDCl_3$, 75 MHz
f1 (ppm)

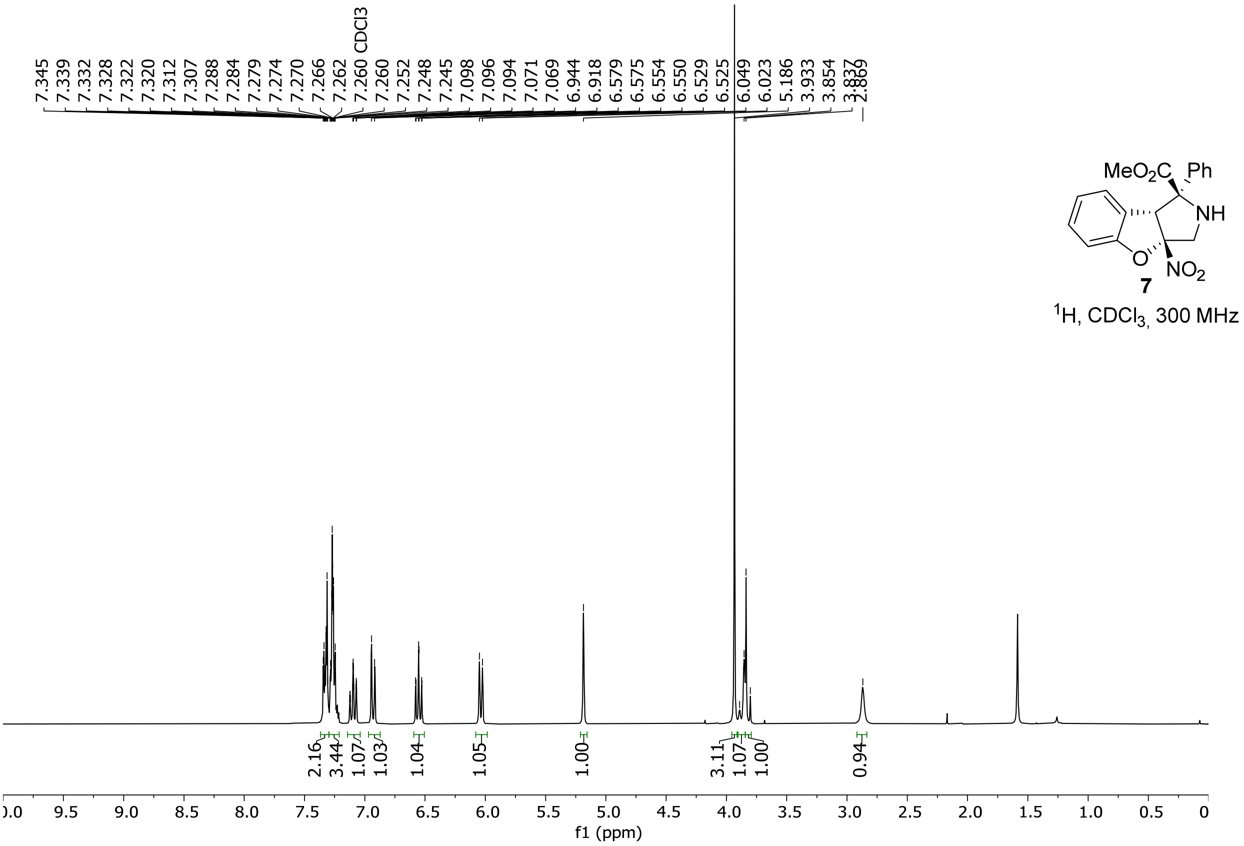
7.345 7.339 7.332 7.328 7.322 7.320 7.312 7.307 7.288 7.284 7.279 7.274 7.270 7.266 7.262 7.260 CDCl3 7.260 7.252 7.248 7.245 7.098 7.096 7.094 7.071 7.069 6.944 6.918 6.579 6.575 6.554 6.550 6.529 6.525 6.049 6.023 5.186 3.933 3.854 3.837 2.869
MeO2C Ph
NH
O
NO2
7
1H, CDCl3, 300 MHz
2.16 3.44 1.07 1.03 1.04 1.05 1.00 3.11 1.07 1.00 0.94
f1 (ppm)

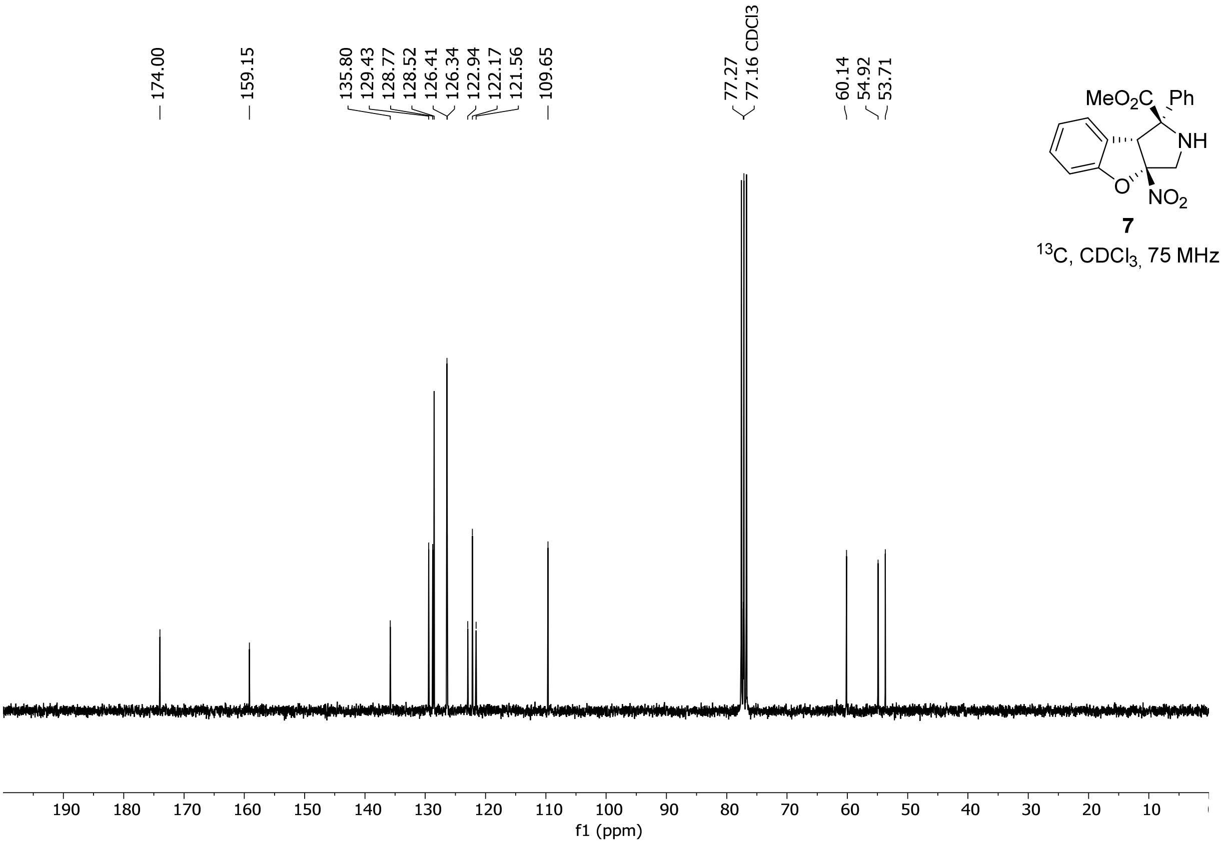
174.00
159.15
135.80 129.43 128.77 128.52 126.41 126.34 122.94 122.17 121.56
109.65
77.27 77.16 CDCl3
60.14 54.92 53.71
MeO2C Ph
NH
O
NO2
7
13C, CDCl3, 75 MHz
f1 (ppm)

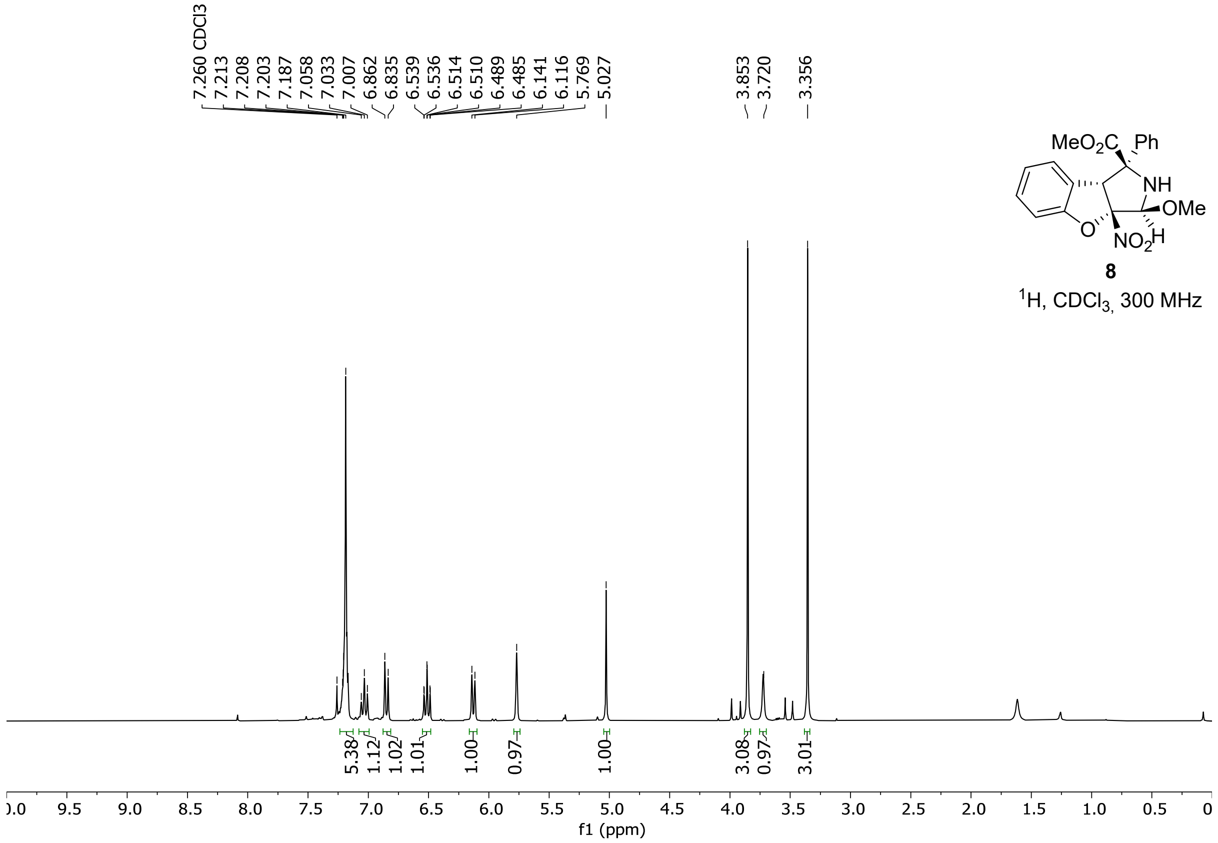
7.260 CDCl3
7.213
7.208
7.203
7.187
7.058
7.033
7.007
6.862
6.835
6.539
6.536
6.514
6.510
6.489
6.485
6.141
6.116
5.769
5.027
3.853
3.720
3.356
MeO2C
Ph
NH
OMe
O
NO2
H
8
1H, CDCl3, 300 MHz
5.38
1.12
1.02
1.01
1.00
0.97
1.00
3.08
0.97
3.01
f1 (ppm)

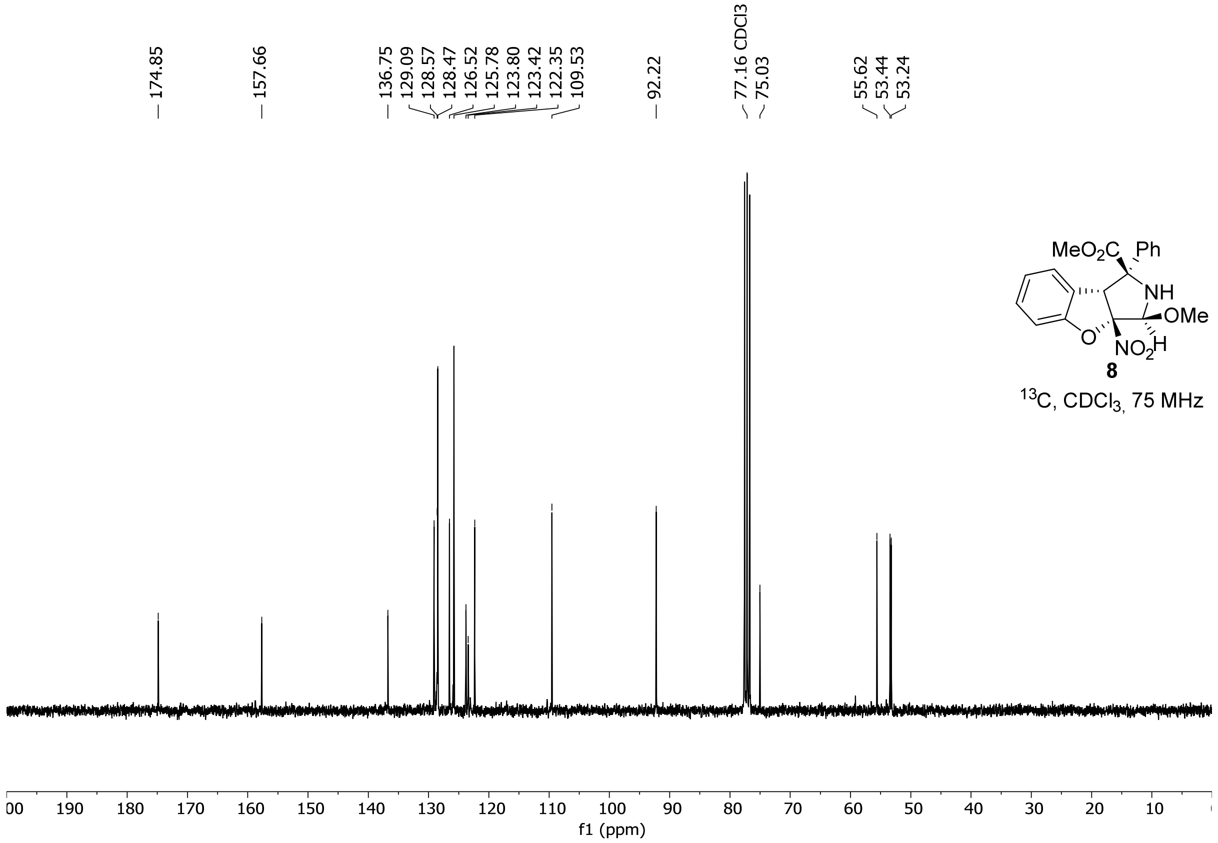
174.85
157.66
136.75
129.09
128.57
128.47
126.52
125.78
123.80
123.42
122.35
109.53
92.22
77.16 CDCl3
75.03
55.62
53.44
53.24
MeO2C
Ph
NH
OMe
O
NO2
H
8
13C, CDCl3, 75 MHz
f1 (ppm)

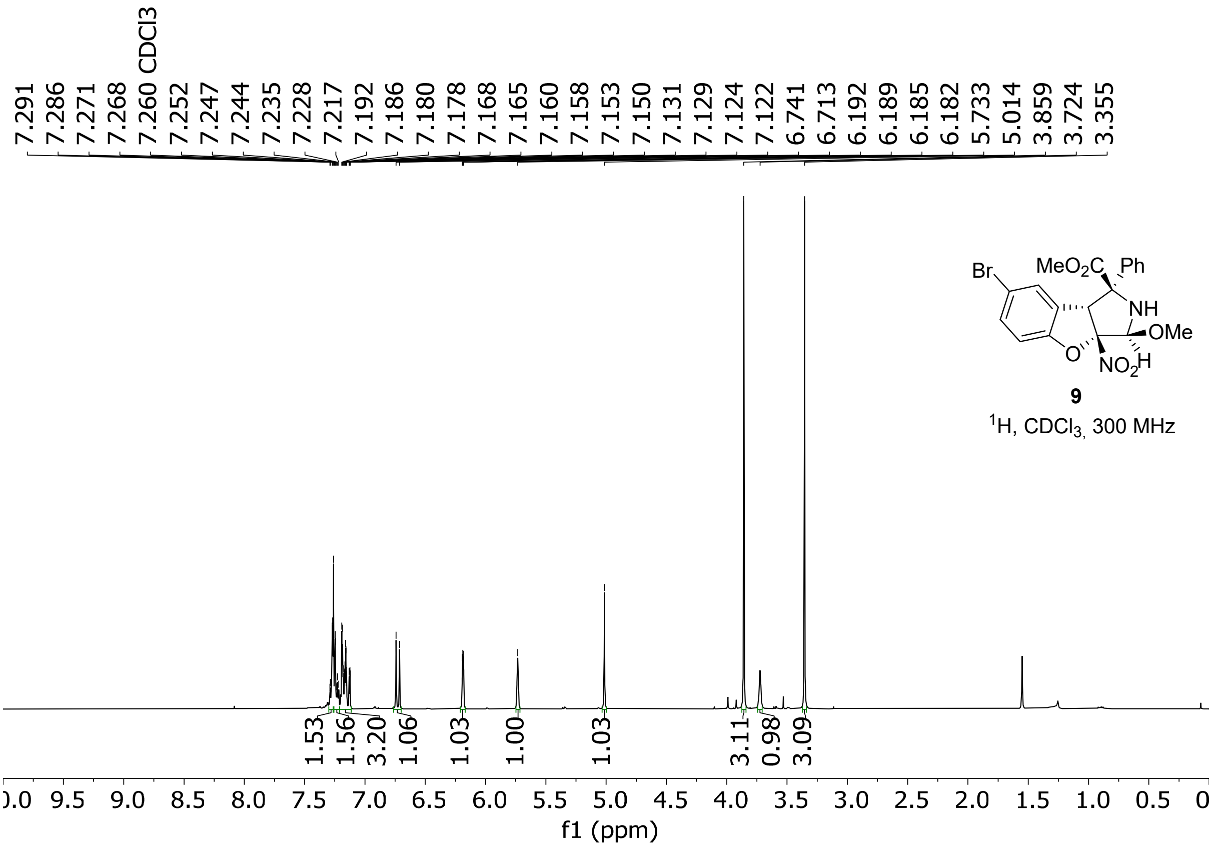
7.291
7.286
7.271
7.268
7.260 CDCl3
7.252
7.247
7.244
7.235
7.228
7.217
7.192
7.186
7.180
7.178
7.168
7.165
7.160
7.158
7.153
7.150
7.131
7.129
7.124
7.122
6.741
6.713
6.192
6.189
6.185
6.182
5.733
5.014
3.859
3.724
3.355
Br
MeO2C
Ph
NH
OMe
O
NO2
H
9
1H, CDCl3, 300 MHz
1.53
1.56
3.20
1.06
1.03
1.00
1.03
3.11
0.98
3.09
f1 (ppm)

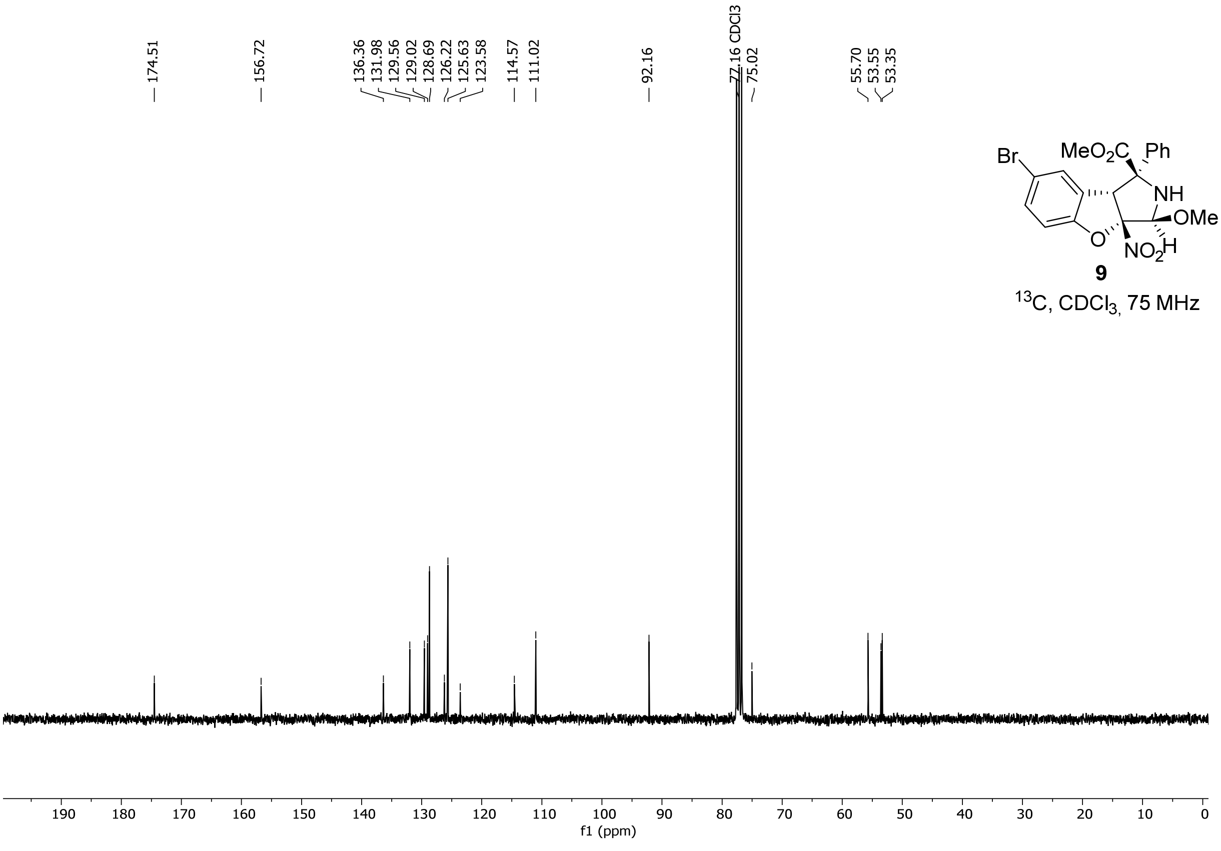
174.51
156.72
136.36
131.98
129.56
129.02
128.69
126.22
125.63
123.58
114.57
111.02
92.16
77.16 CDCl3
75.02
55.70
53.55
53.35
Br
MeO2C
Ph
NH
OMe
O
NO2
H
9
13C, CDCl3, 75 MHz
f1 (ppm)

HPLC spectra

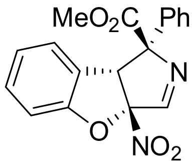

3aa

Racemic product (diastereomeric mixture)

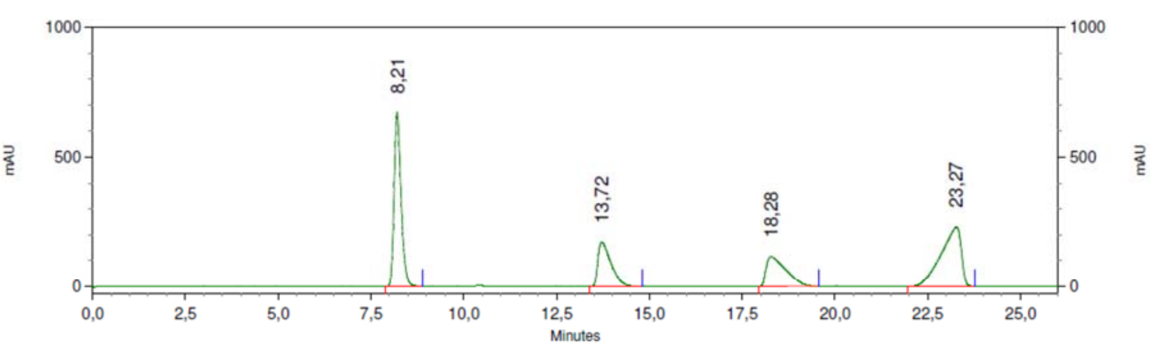

29: 274 nm, 4 nm
Results

Retention Time	Area	Area Percent
8,21	34950134	33,688
13,72	16827736	16,220
18,28	17022510	16,408
23,27	34946519	33,684

Enantioenriched product

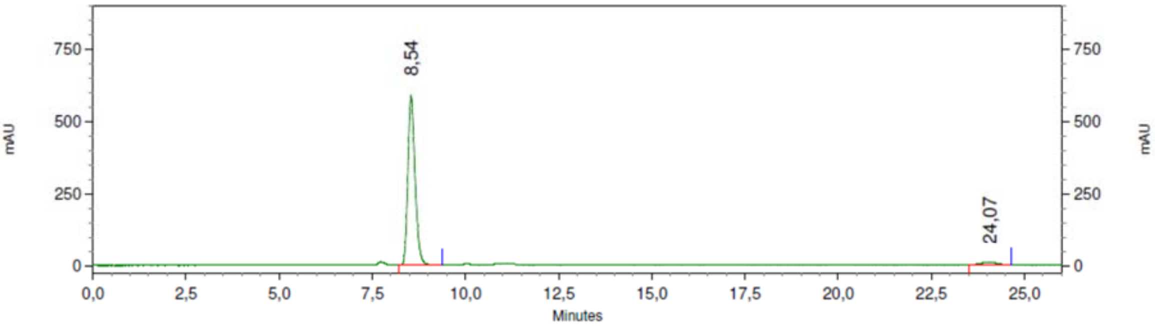

29: 274 nm, 4 nm
Results

Retention Time	Area	Area Percent
8,54	31575867	96,296
24,07	1214637	3,704

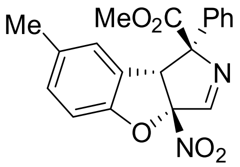

3ba

Racemic product

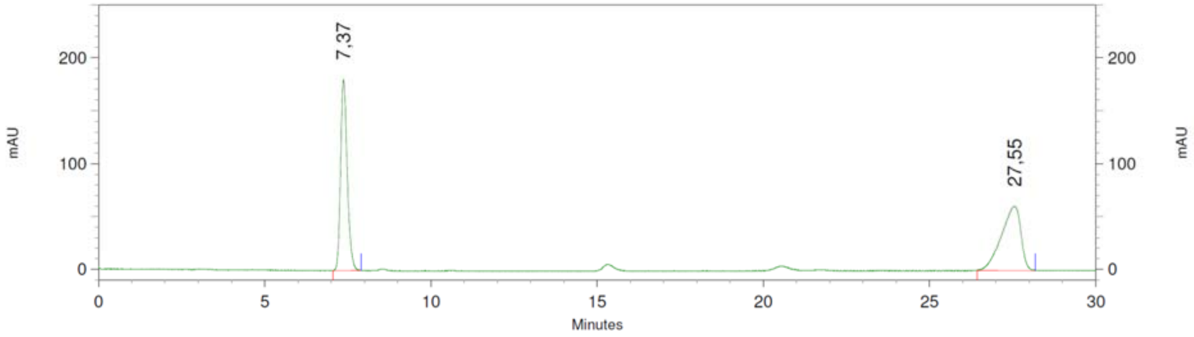

48: 283 nm, 4 nm
Results

Retention Time	Area	Area Percent
7,37	10013749	50,051
27,55	9993312	49,949

Enantioenriched product

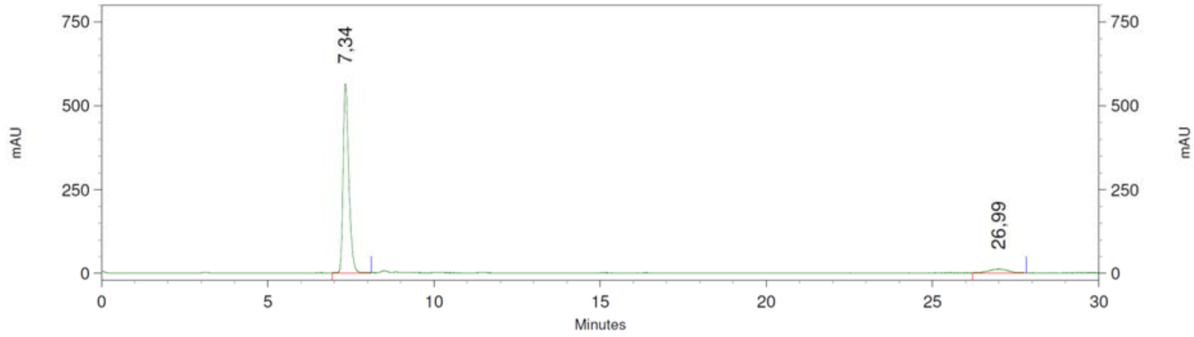

48: 283 nm, 4 nm
Results

Retention Time	Area	Area Percent
7,34	26972470	93,985
26,99	1726270	6,015

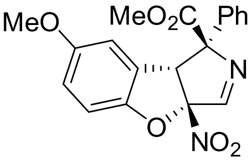

3ca

Racemic product (diastereomeric mixture)

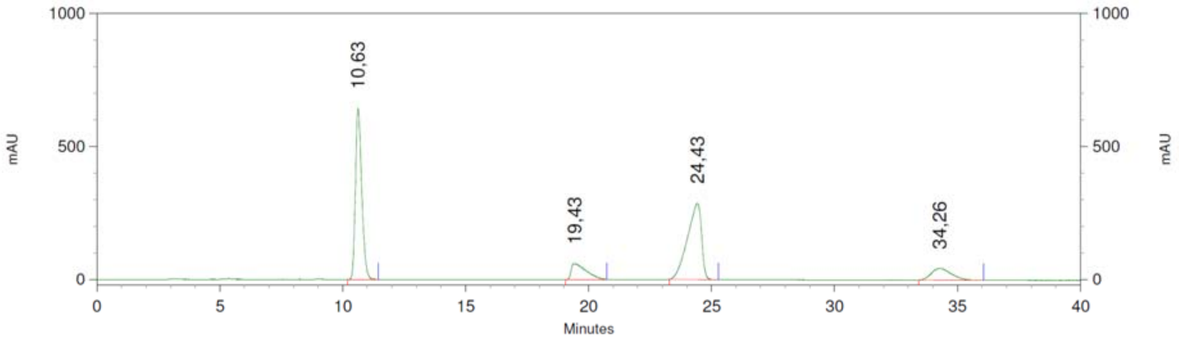

44: 296 nm, 4 nm
Results

Retention Time	Area	Area Percent
10,63	45907309	41,184
19,43	10073935	9,037
24,43	45589124	40,899
34,26	9898188	8,880

Enantioenriched product

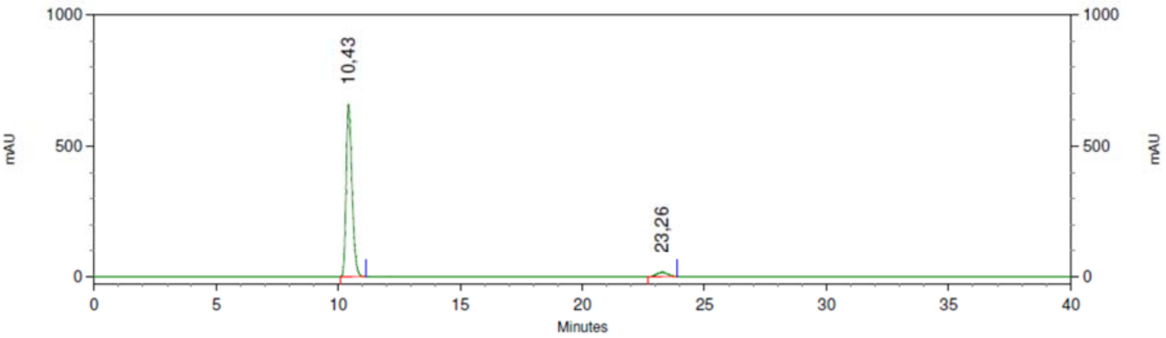

64: 296 nm, 4 nm
Results

Retention Time	Area	Area Percent
10,43	43937407	95,686
23,26	1980949	4,314

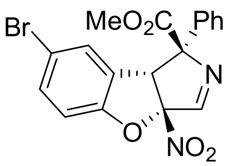

3da

Racemic product (diastereomeric mixture)

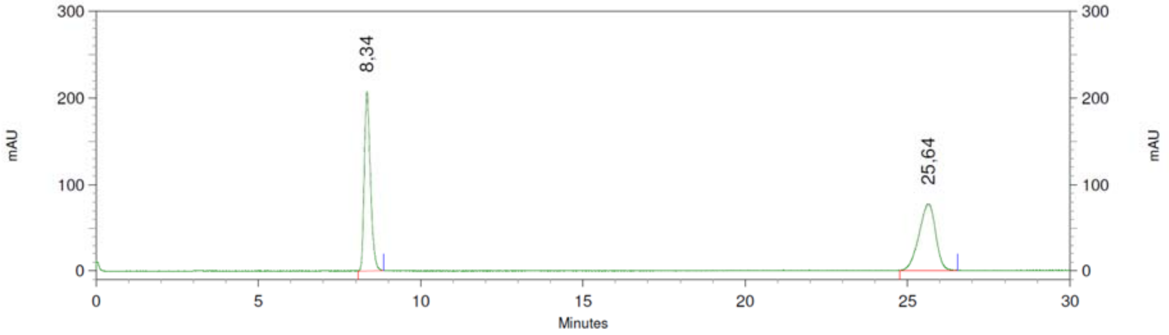

53: 286 nm, 4 nm
Results

Retention Time	Area	Area Percent
8,34	10974299	49,875
25,64	11029433	50,125

Enantioenriched product

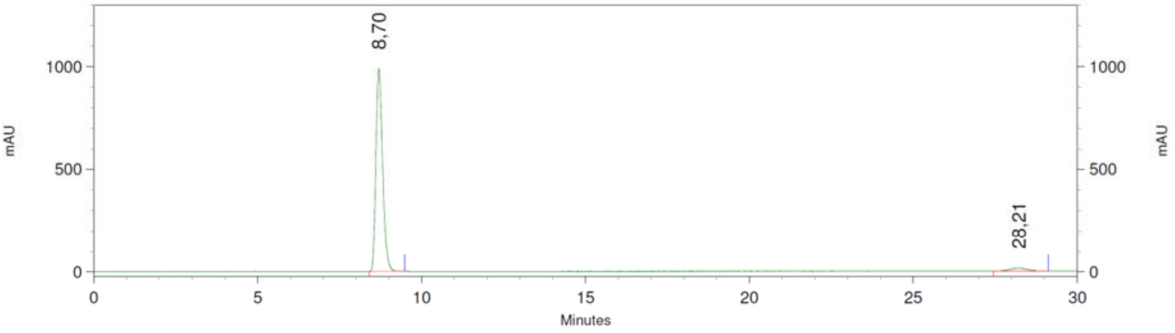

3: 286 nm, 4 nm Results

Retention Time	Area	Area Percent
8,70	56013362	95,875
28,21	2410145	4,125

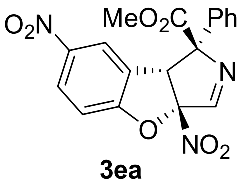

3ea

Racemic product (diastereomeric mixture)

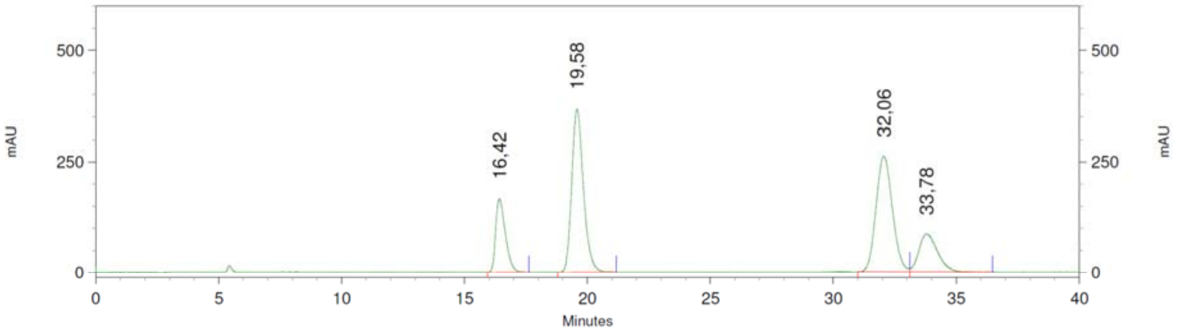

48: 283 nm, 4 nm
Results

Retention Time	Area	Area Percent
16,42	18255926	13,651
19,58	48713093	36,425
32,06	48447137	36,226
33,78	18319249	13,698

Enantioenriched product

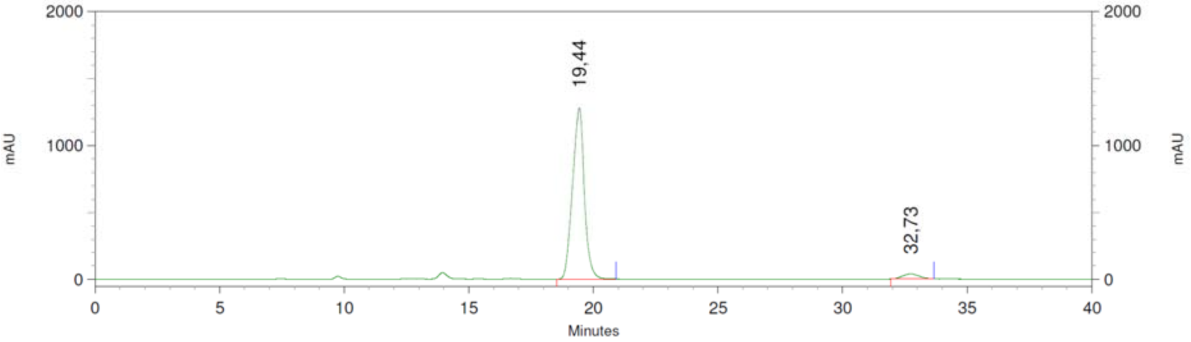

48: 283 nm, 4 nm
Results

Retention Time	Area	Area Percent
19,44	168757541	96,095
32,73	6857555	3,905

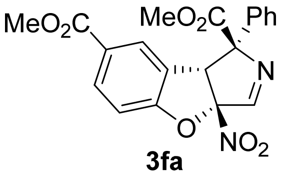

3fa

Racemic product (diastereomeric mixture)

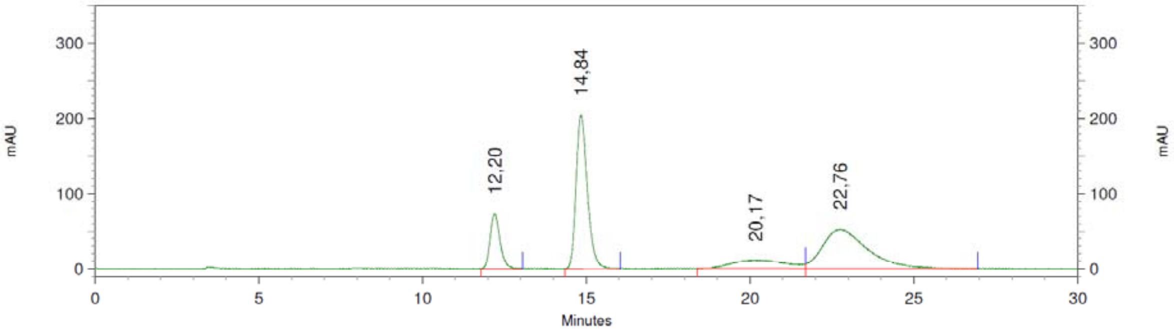

8: 246 nm, 4 nm Results

Retention Time	Area	Area Percent
12,20	6134384	12,089
14,84	19639812	38,704
20,17	5005361	9,864
22,76	19963611	39,342

Enantioenriched product

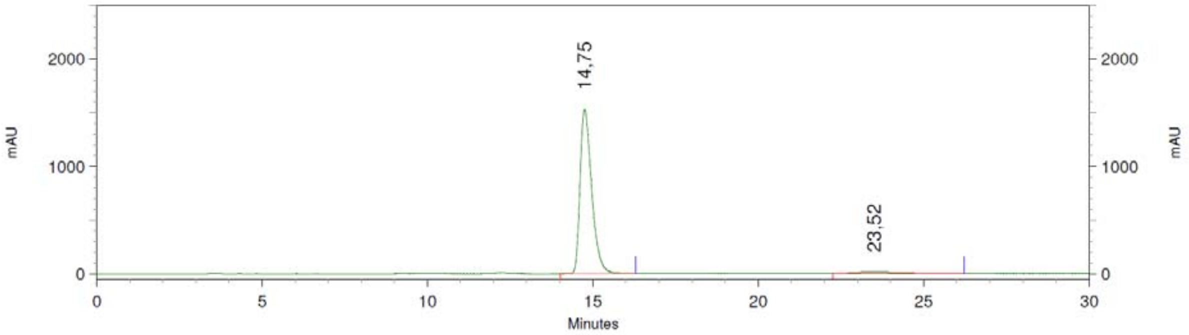

8: 246 nm, 4 nm Results

Retention Time	Area	Area Percent
14,75	149651491	95,748
23,52	6645687	4,252

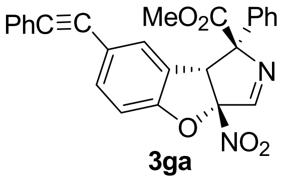

3ga

Racemic product (diastereomeric mixture)

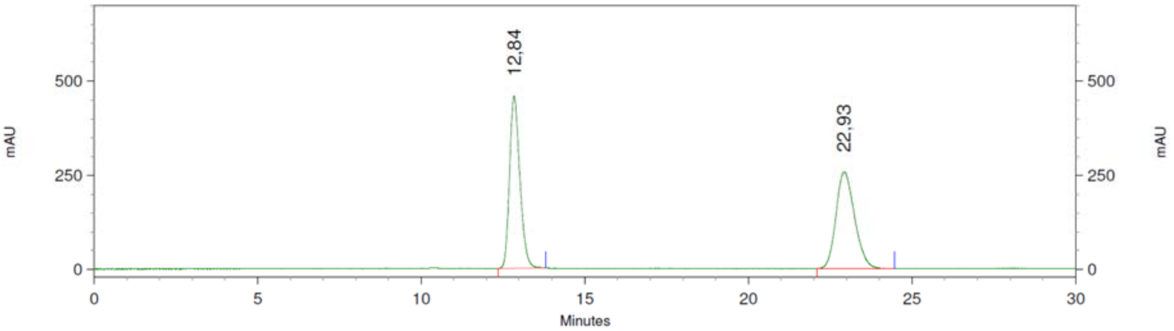

3: 286 nm, 4 nm Results

Retention Time	Area	Area Percent
12,84	40545156	49,804
22,93	40864909	50,196

Enantioenriched product

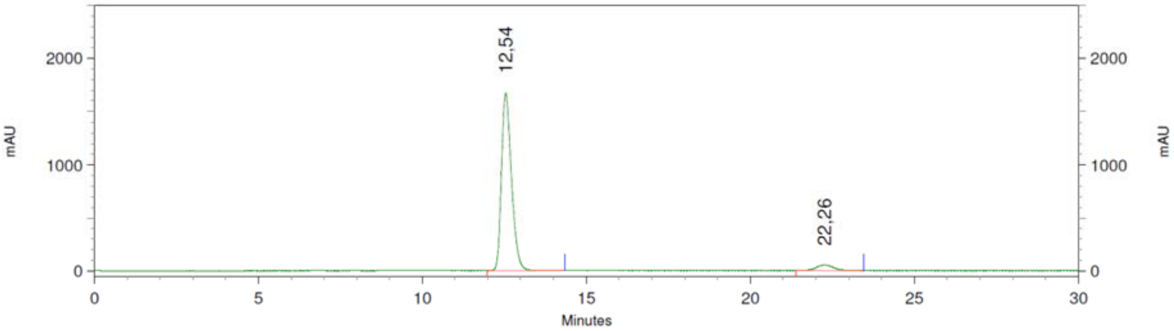

3: 286 nm, 4 nm Results

Retention Time	Area	Area Percent
12,54	141996711	94,652
22,26	8023383	5,348

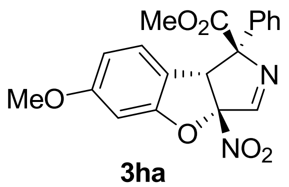

3ha

Racemic product (diastereomeric mixture)

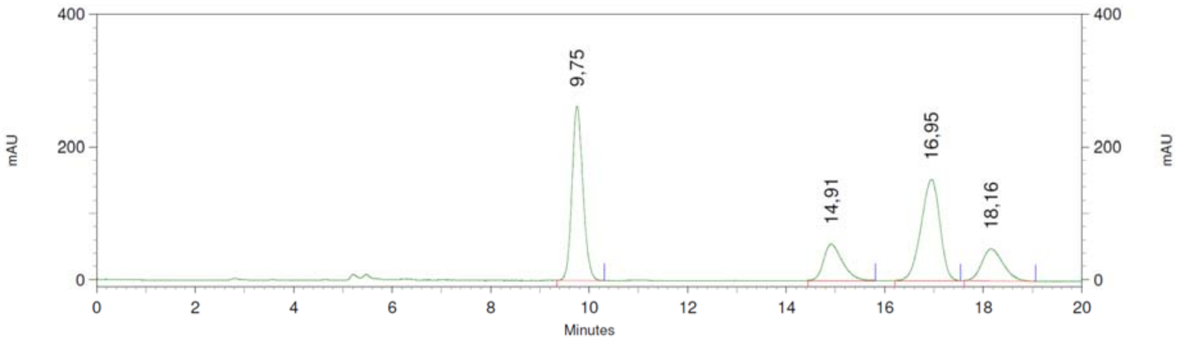

52: 281 nm, 4 nm
Results

Retention Time	Area	Area Percent
9,75	16266524	36,848
14,91	5831753	13,211
16,95	16228268	36,762
18,16	5817963	13,179

Enantioenriched product

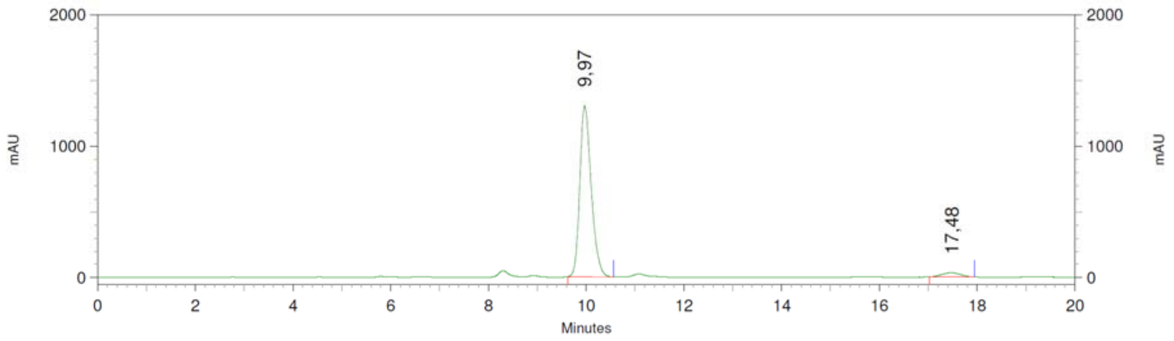

52: 281 nm, 4 nm
Results

Retention Time	Area	Area Percent
9,97	84862168	96,104
17,48	3439836	3,896

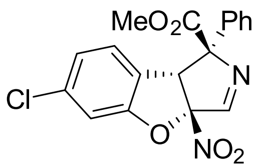

3ia

Racemic product

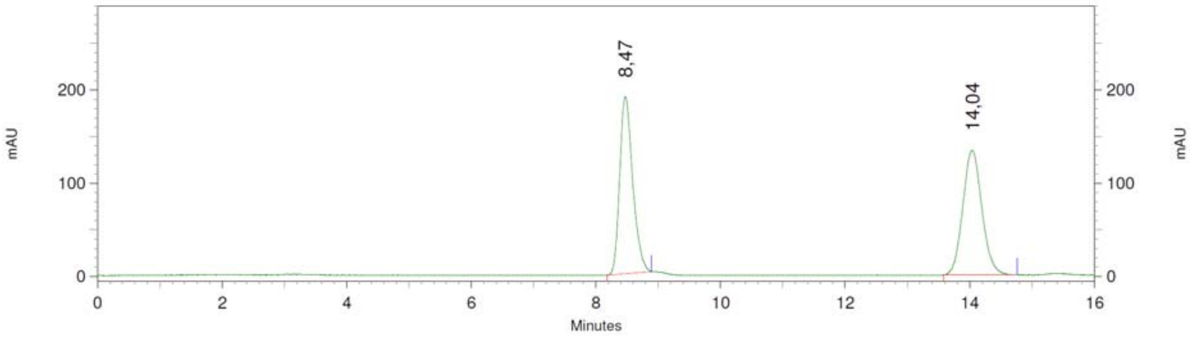

42: 279 nm, 4 nm
Results

Retention Time	Area	Area Percent
8,47	11037512	49,142
14,04	11422726	50,858

Enantioenriched product

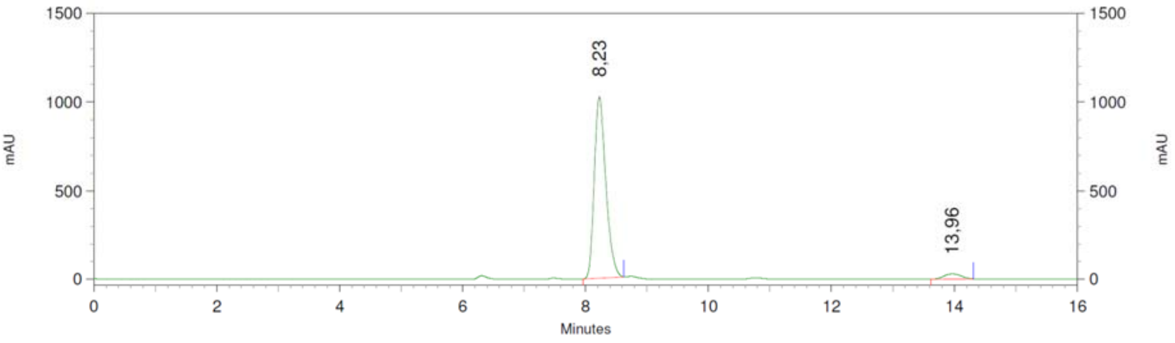

42: 279 nm, 4 nm
Results

Retention Time	Area	Area Percent
8,23	53748924	95,981
13,96	2250432	4,019

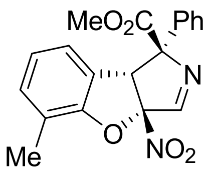

3ja

Racemic product (diastereomeric mixture)

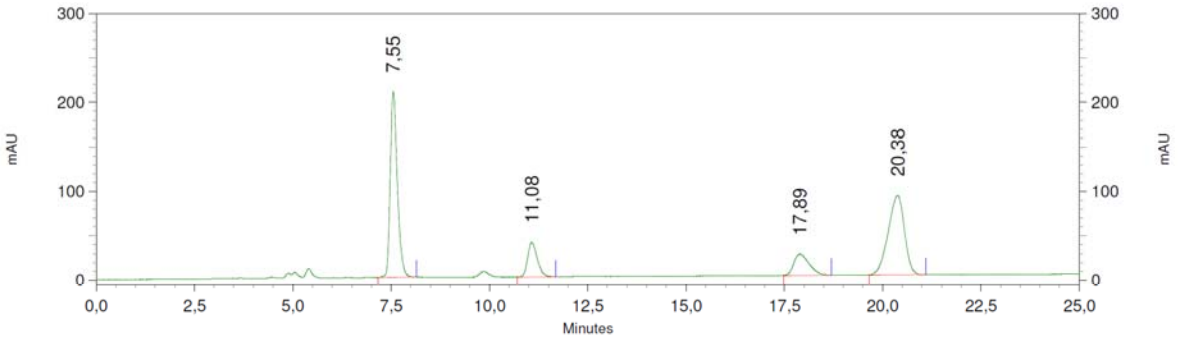

29: 274 nm, 4 nm
Results

Retention Time	Area	Area Percent
7,55	10451419	40,028
11,08	2689876	10,302
17,89	2647034	10,138
20,38	10322111	39,533

Enantioenriched product

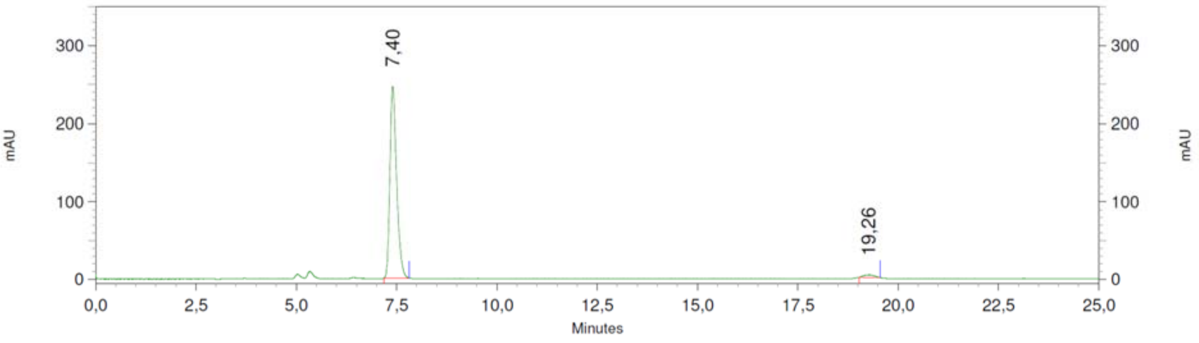

29: 274 nm, 4 nm
Results

Retention Time	Area	Area Percent
7,40	11471625	97,940
19,26	241242	2,060

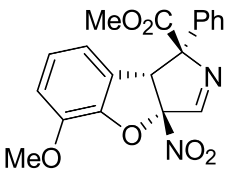

3ka

Racemic product (diastereomeric mixture)

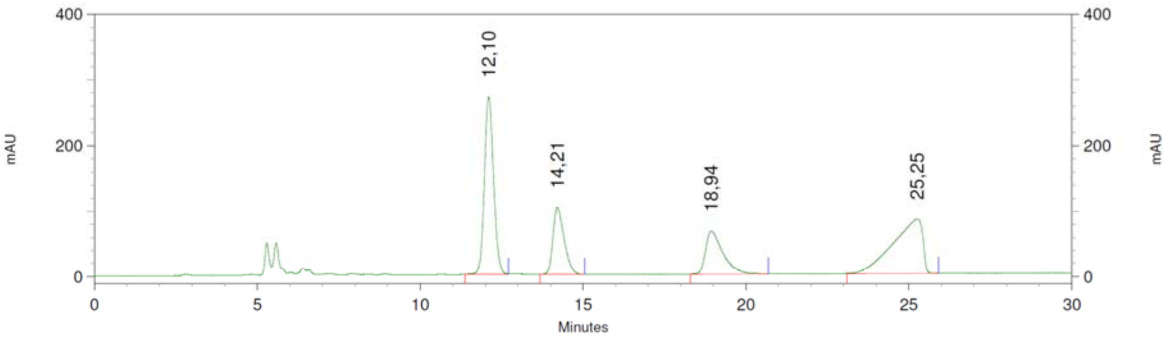


```
51: 279 nm, 4 nm
Results
```

Retention Time	Area	Area Percent
12,10	21179029	34,075
14,21	9883000	15,901
18,94	9824686	15,807
25,25	21266977	34,217

Enantioenriched product

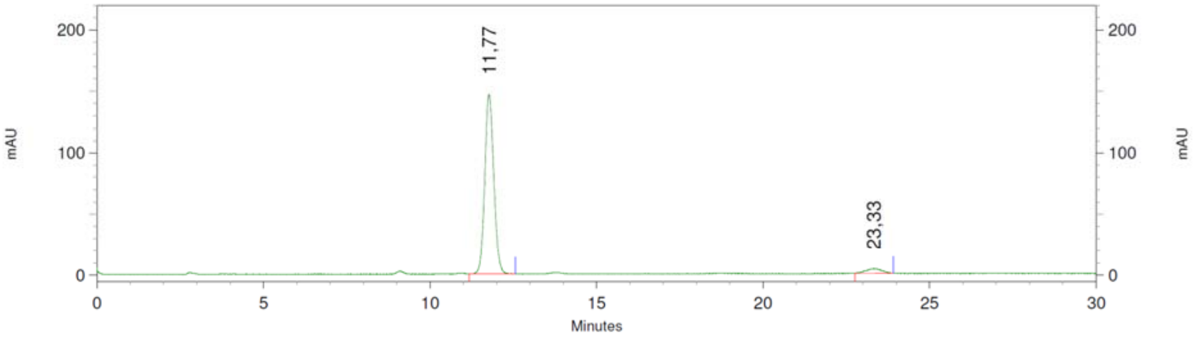


```
51: 279 nm, 4 nm
Results
```

Retention Time	Area	Area Percent
11,77	11047301	95,646
23,33	502912	4,354

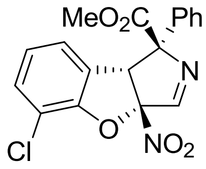

3la

Racemic product (diastereomeric mixture)

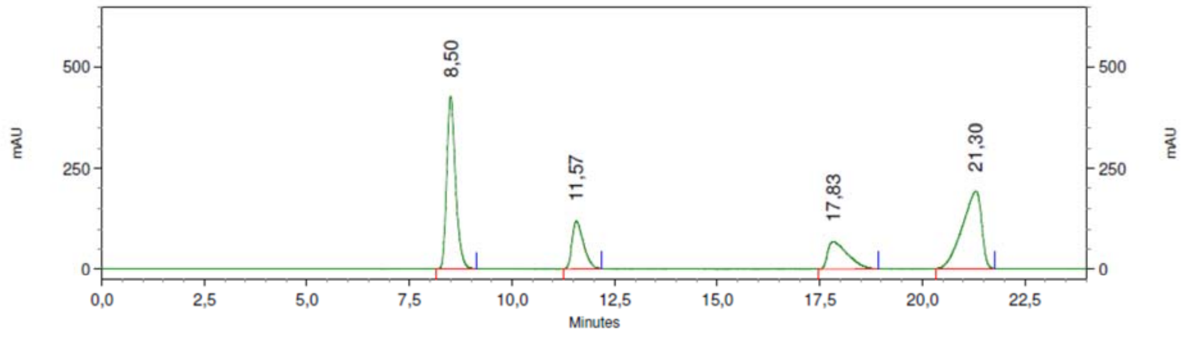

47: 276 nm, 4 nm
Results

Retention Time	Area	Area Percent
8,50	25576383	36,874
11,57	9241689	13,324
17,83	9234741	13,314
21,30	25307992	36,487

Enantioenriched product

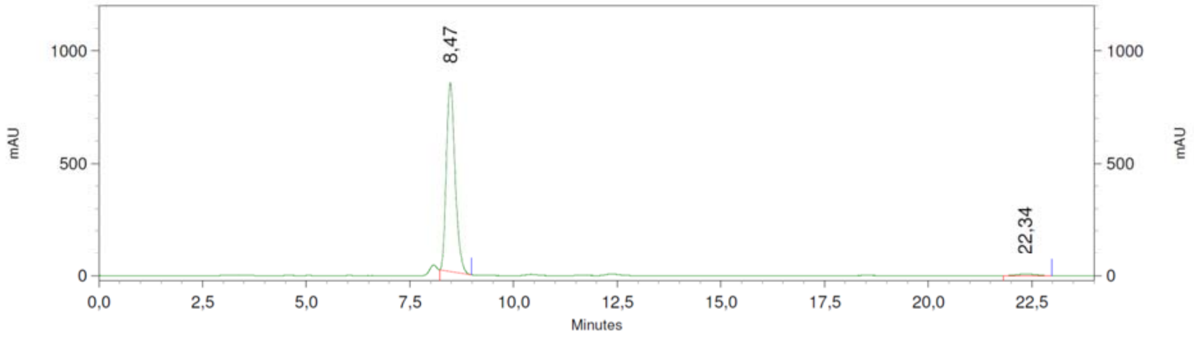

47: 276 nm, 4 nm
Results

Retention Time	Area	Area Percent
8,47	49047148	98,024
22,34	988488	1,976

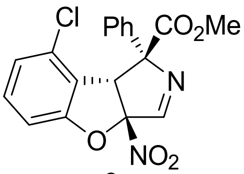

3ma

Racemic product (diastereomeric mixture)

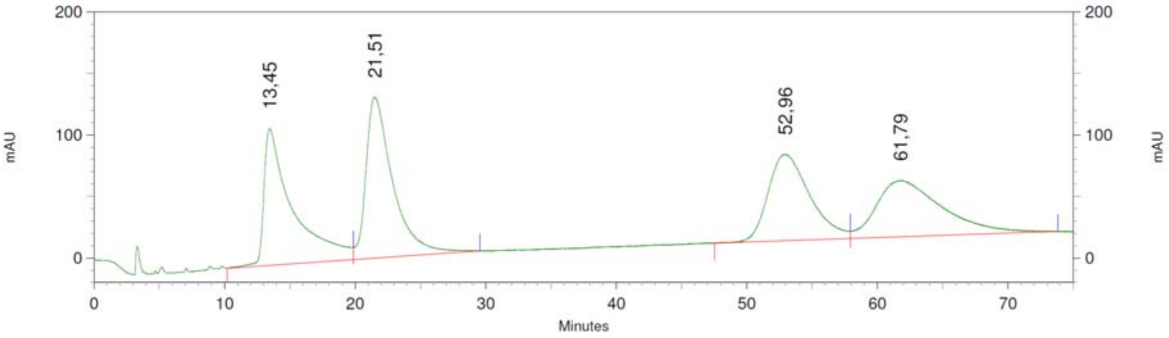

27: 222 nm, 4 nm
Results

Retention Time	Area	Area Percent
13,45	67913068	25,225
21,51	74739023	27,760
52,96	62334930	23,153
61,79	64245117	23,862

Enantioenriched product

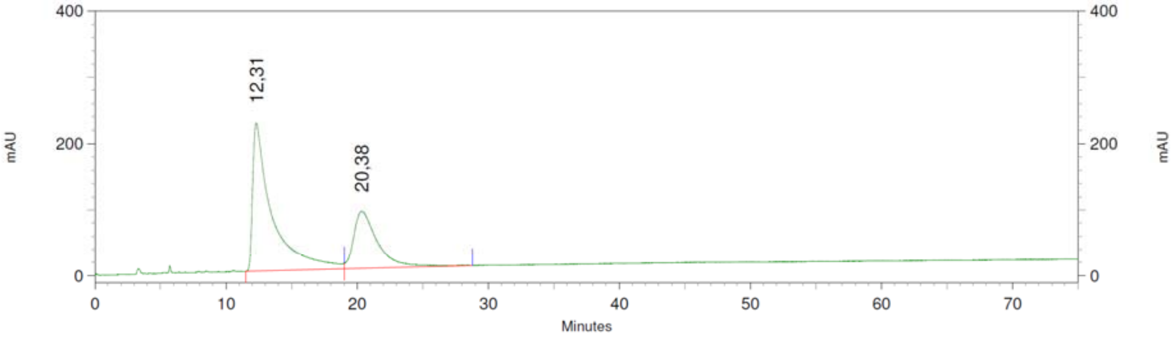

37: 222 nm, 4 nm
Results

Retention Time	Area	Area Percent
12,31	92290295	67,780
20,38	43872033	32,220

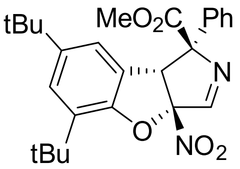

3na

Racemic product

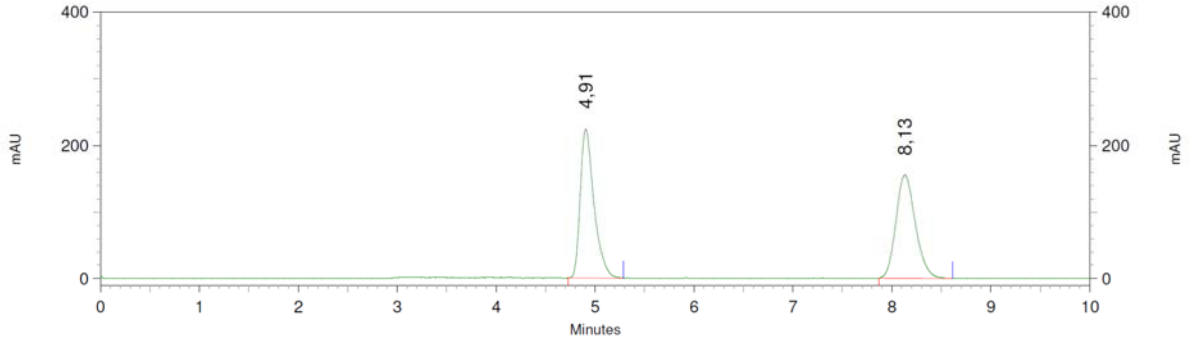

51: 279 nm, 4 nm
Results

Retention Time	Area	Area Percent
4,91	8669960	50,420
8,13	8525539	49,580

Enantioenriched product

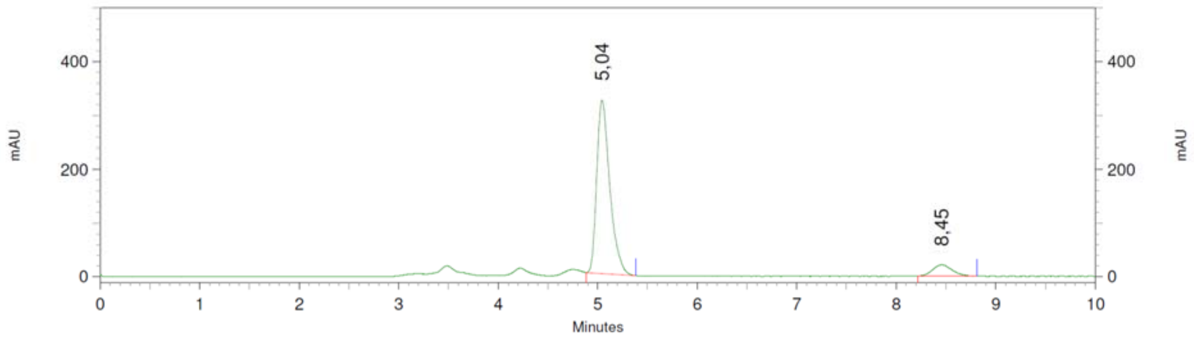

51: 279 nm, 4 nm
Results

Retention Time	Area	Area Percent
5,04	11783221	90,946
8,45	1173040	9,054

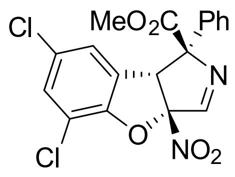

3oa

Racemic product

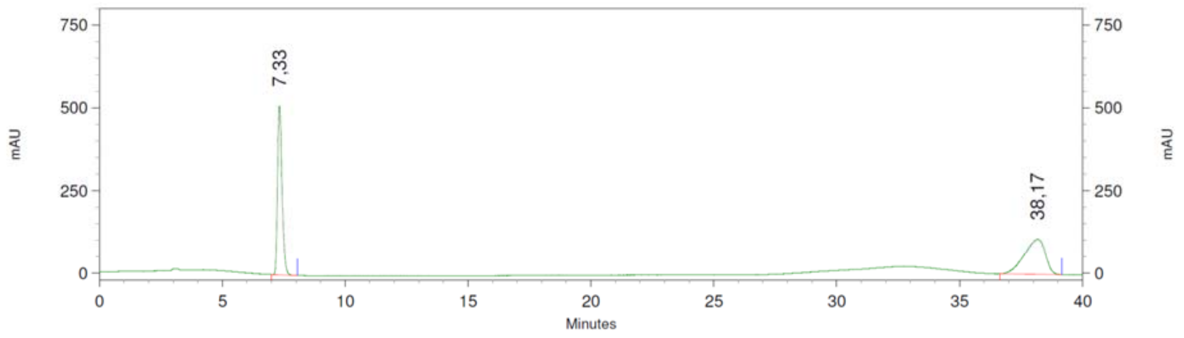

34: 288 nm, 4 nm
Results

Retention Time	Area	Area Percent
7,33	25978567	50,516
38,17	25447934	49,484

Enantioenriched product

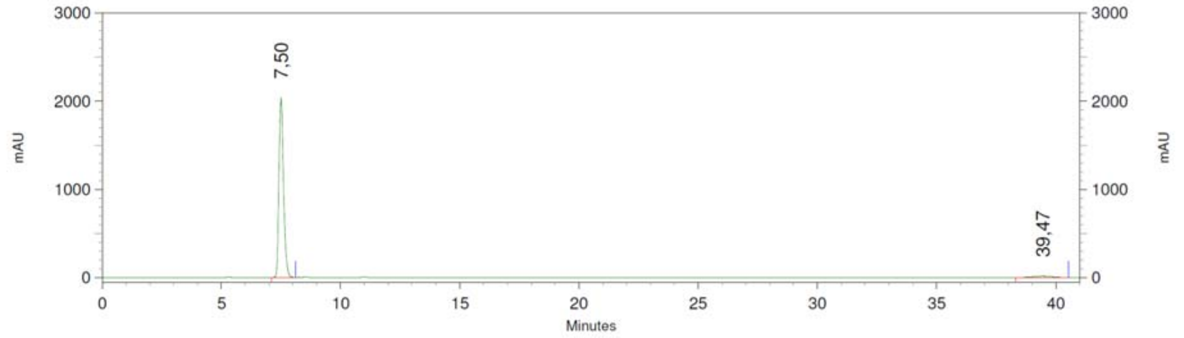

34: 288 nm, 4 nm
Results

Retention Time	Area	Area Percent
7,50	108140163	97,017
39,47	3324692	2,983

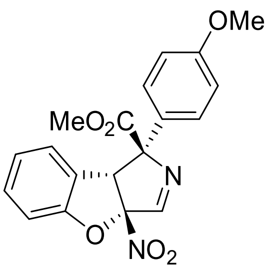

3ab

Racemic product

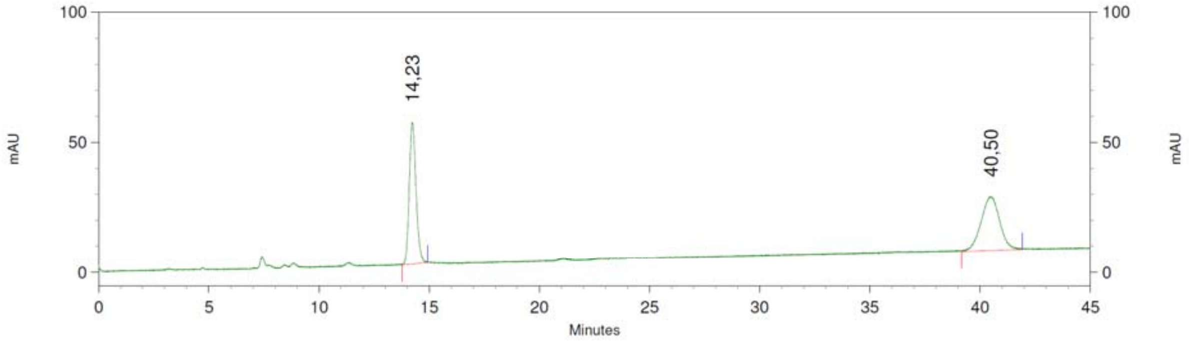

62: 273 nm, 4 nm
Results

Retention Time	Area	Area Percent
14,23	4644233	48,871
40,50	4858764	51,129

Enantioenriched product

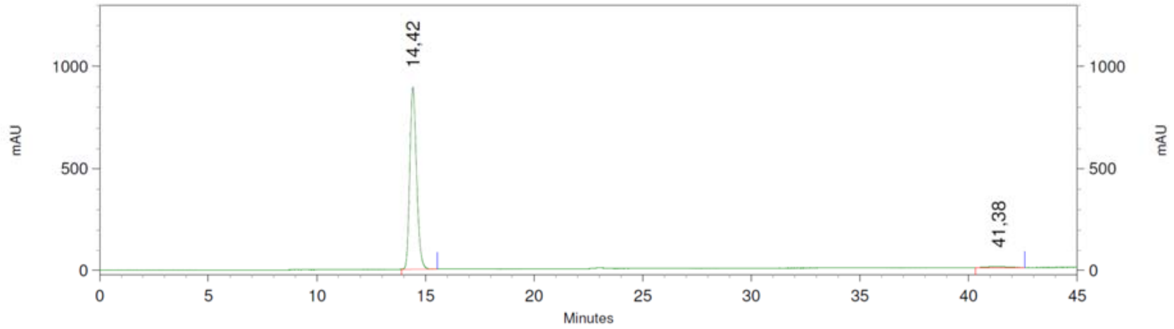

62: 273 nm, 4 nm
Results

Retention Time	Area	Area Percent
14,42	77989354	98,367
41,38	1294550	1,633

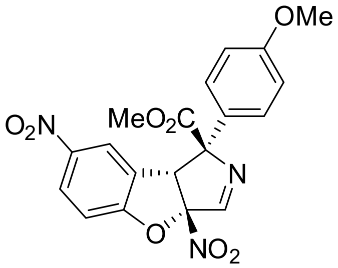

3eb

Racemic product

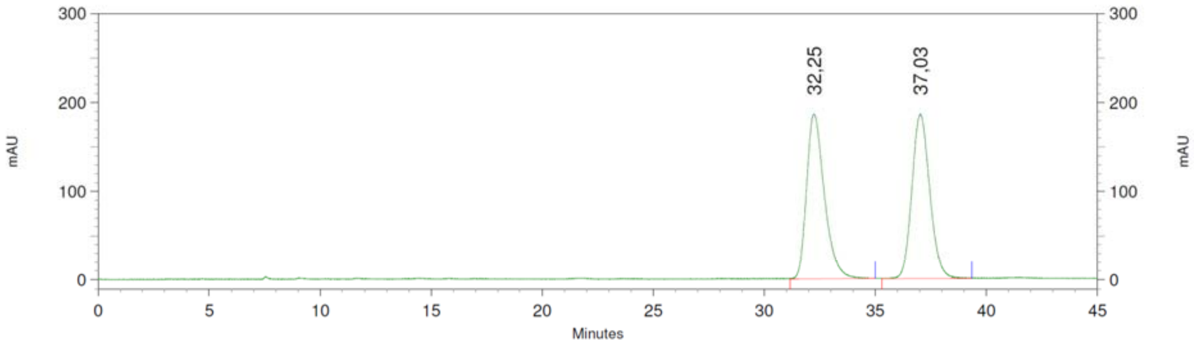

51: 279 nm, 4 nm
Results

Retention Time	Area	Area Percent
32,25	41638223	50,053
37,03	41550339	49,947

Enantioenriched product

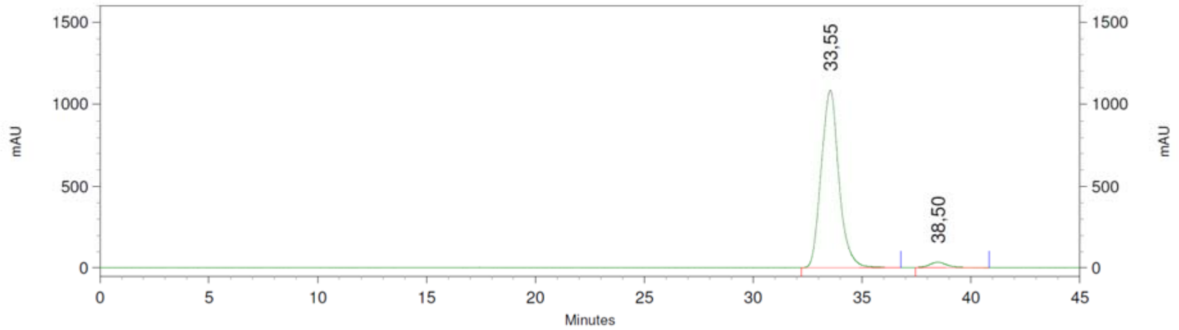

51: 279 nm, 4 nm
Results

Retention Time	Area	Area Percent
33,55	245342031	97,015
38,50	7548866	2,985

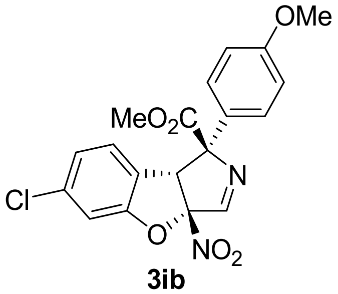

3ib

Racemic product

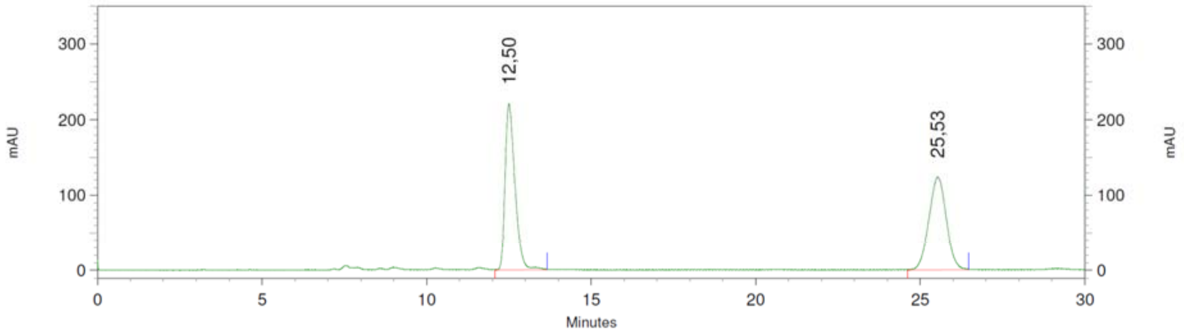

51: 279 nm, 4 nm
Results

Retention Time	Area	Area Percent
12,50	18397464	50,351
25,53	18141151	49,649

Enantioenriched product

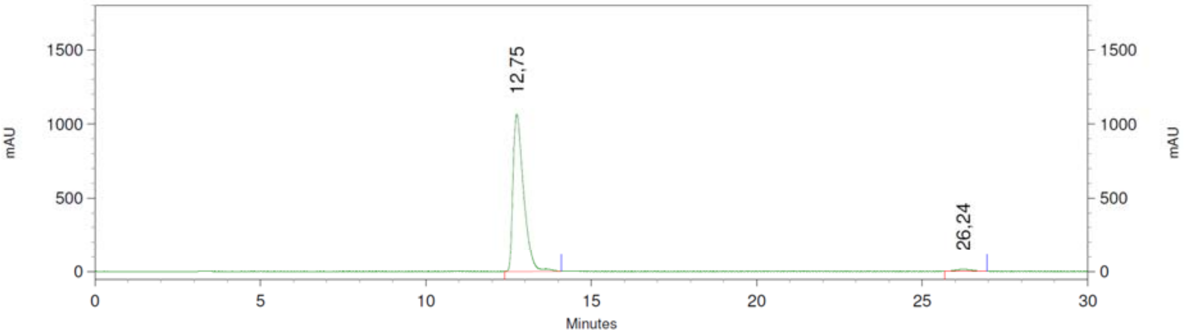

51: 279 nm, 4 nm
Results

Retention Time	Area	Area Percent
12,75	95232669	98,033
26,24	1910385	1,967

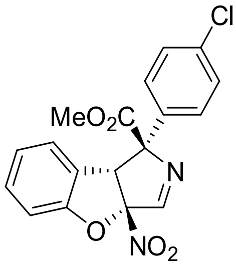

3ac

Racemic product (diastereomeric mixture)

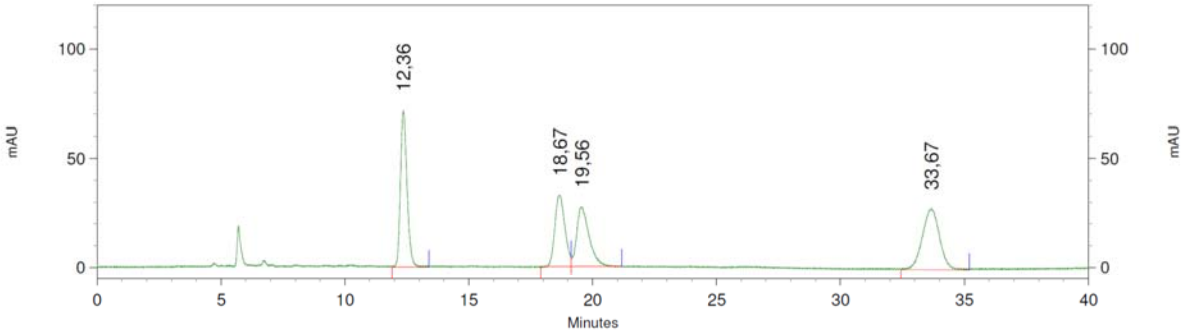

62: 273 nm, 4 nm
Results

Retention Time	Area	Area Percent
12,36	5290478	29,074
18,67	3673650	20,188
19,56	3786009	20,806
33,67	5446618	29,932

Enantioenriched product

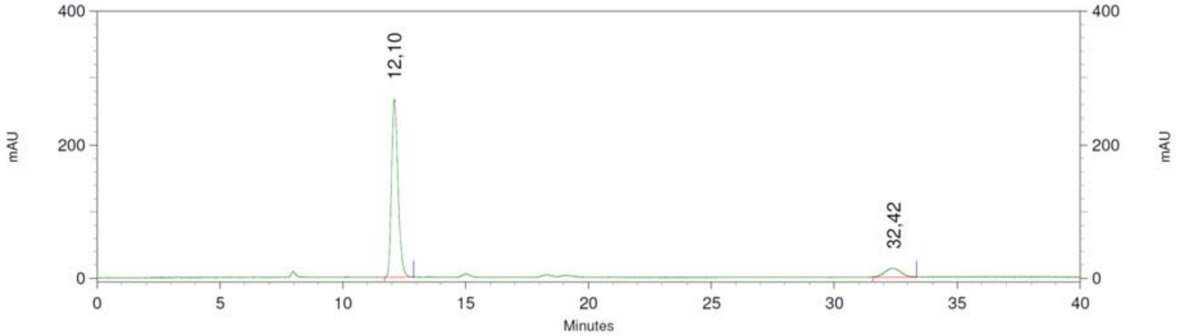

62: 273 nm, 4 nm
Results

Retention Time	Area	Area Percent
12,10	19531125	88,883
32,42	2442868	11,117

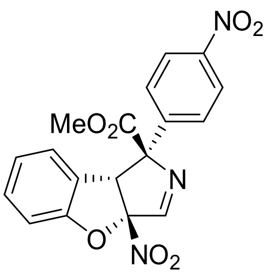

3ad

Racemic product

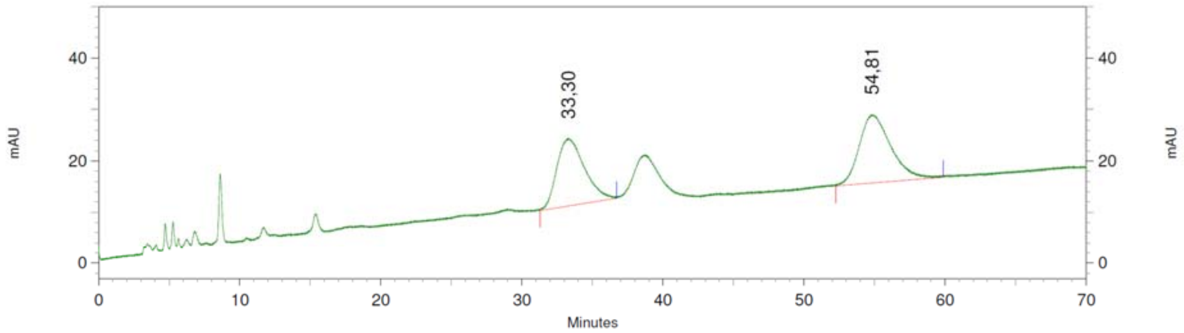

61: 261 nm, 4 nm
Results

Retention Time	Area	Area Percent
33,30	7199988	46,356
54,81	8331876	53,644

Enantioenriched product

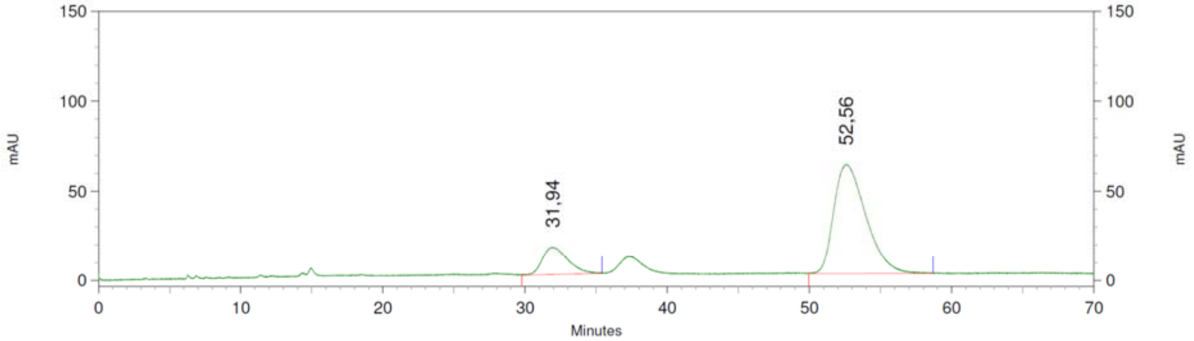

61: 261 nm, 4 nm
Results

Retention Time	Area	Area Percent
31,94	7639373	16,590
52,56	38407833	83,410

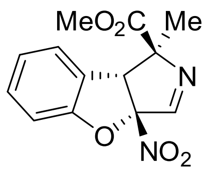

3ae

Racemic product

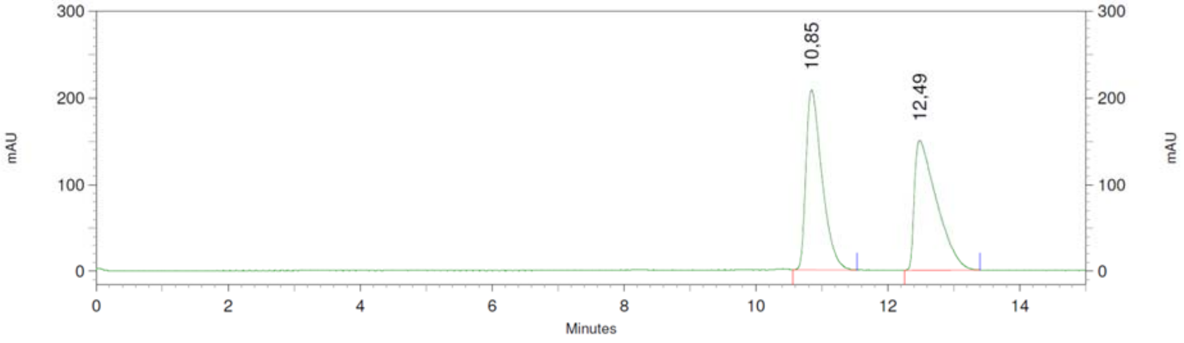

51: 279 nm, 4 nm
Results

Retention Time	Area	Area Percent
10,85	14071177	49,863
12,49	14148376	50,137

Enantioenriched product

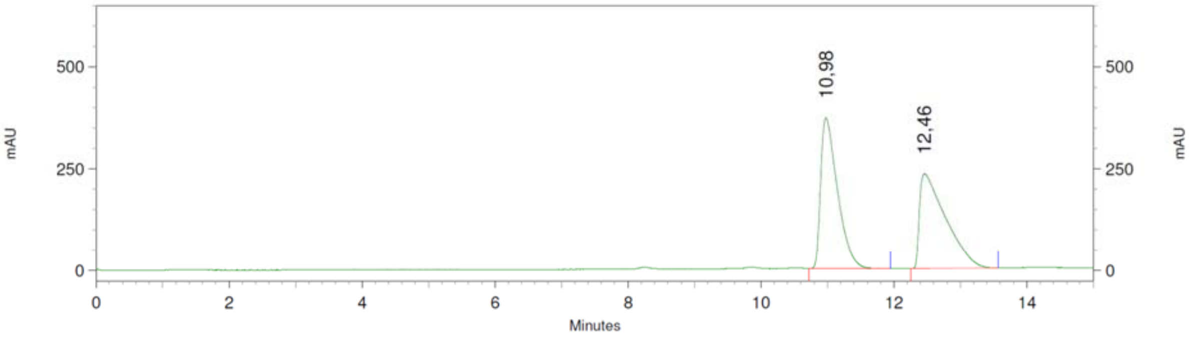

51: 279 nm, 4 nm
Results

Retention Time	Area	Area Percent
10,98	26113573	50,560
12,46	25535372	49,440

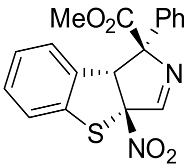

4

Racemic product

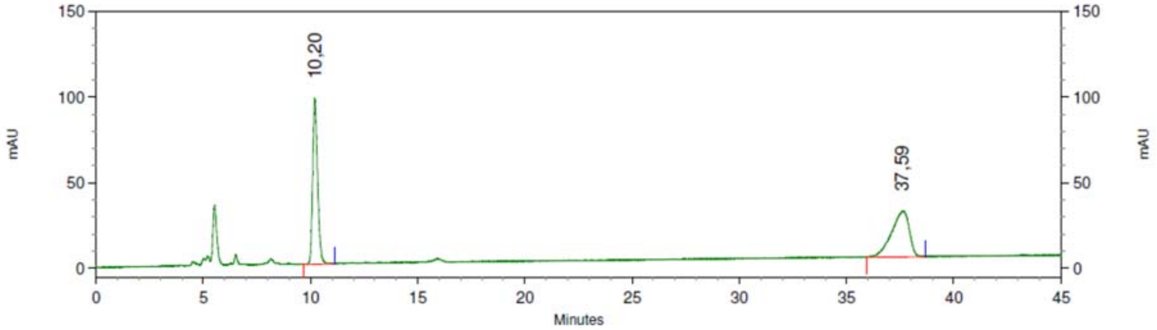

51: 279 nm, 4 nm
Results

Retention Time	Area	Area Percent
10,20	6248643	49,896
37,59	6274698	50,104

Enantioenriched product

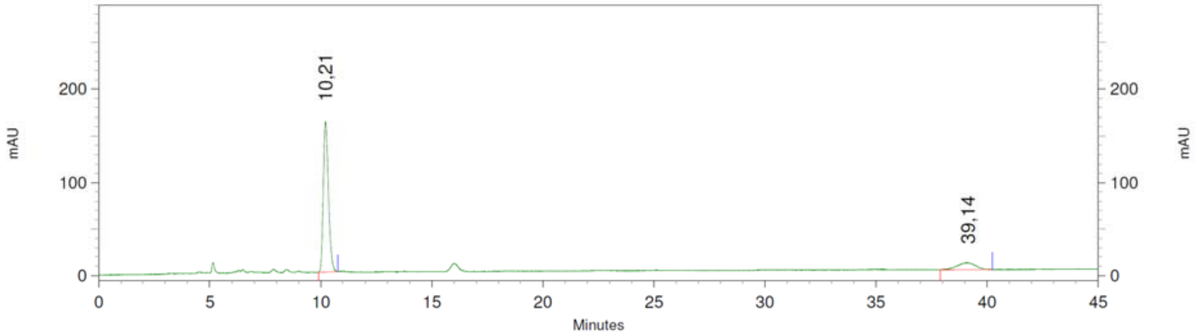

51: 279 nm, 4 nm
Results

Retention Time	Area	Area Percent
10,21	10383407	85,818
39,14	1715968	14,182

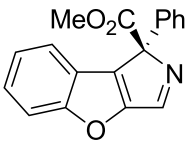

6

Racemic product

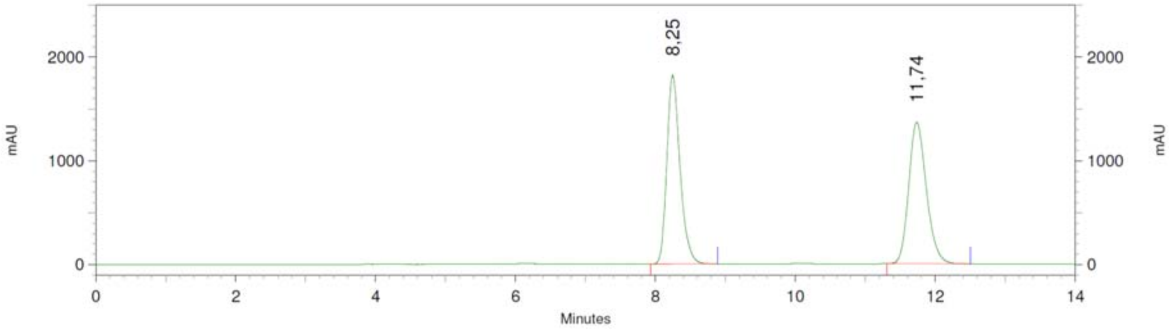

9: 306 nm, 4 nm Results

Retention Time	Area	Area Percent
8,25	95512315	49,940
11,74	95739909	50,060

Enantioenriched product

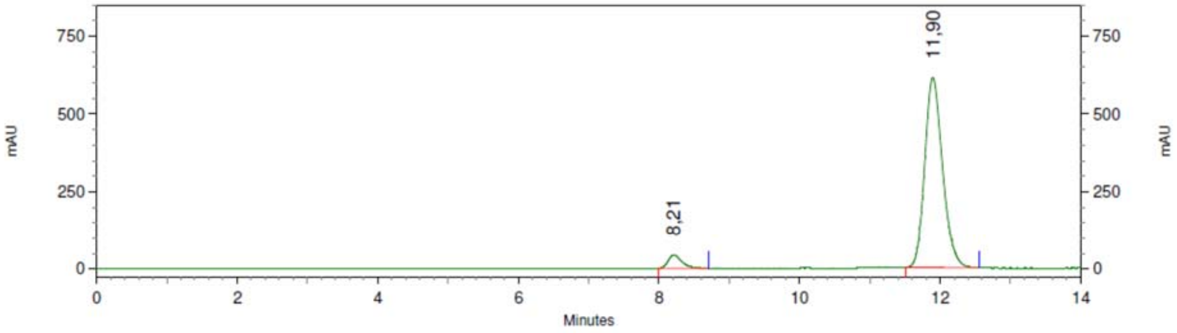

9: 306 nm, 4 nm Results

Retention Time	Area	Area Percent
8,21	2310412	5,052
11,90	43417824	94,948

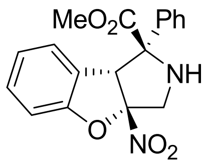

7

Racemic product (diastereomeric mixture)

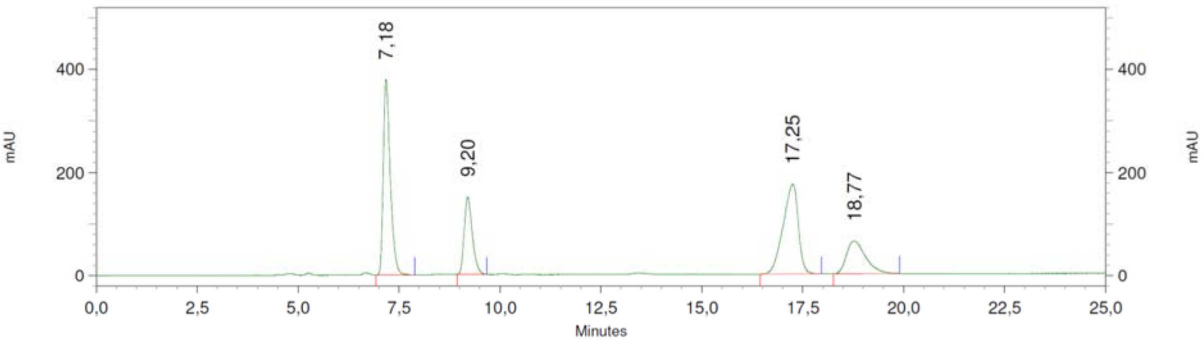

52: 281 nm, 4 nm
Results

Retention Time	Area	Area Percent
7,18	18226971	34,153
9,20	8436177	15,807
17,25	18308201	34,305
18,77	8397522	15,735

Enantioenriched product

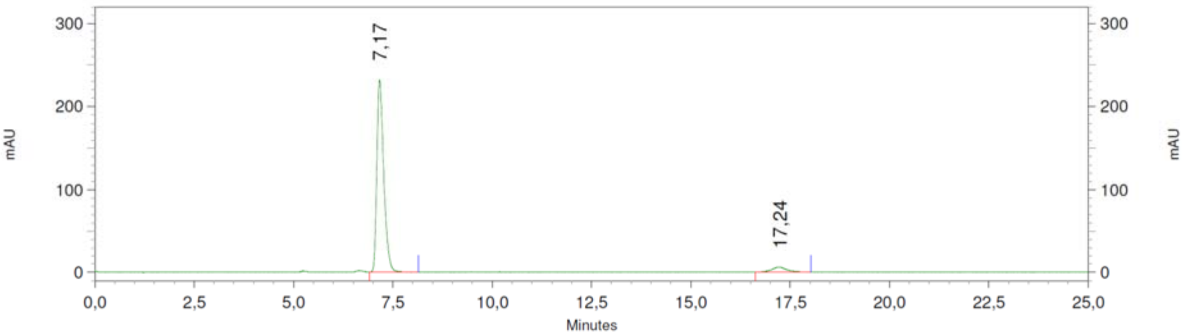

52: 281 nm, 4 nm
Results

Retention Time	Area	Area Percent
7,17	11269996	94,535
17,24	651512	5,465

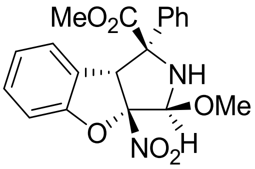

8

Racemic product (diastereomeric mixture)

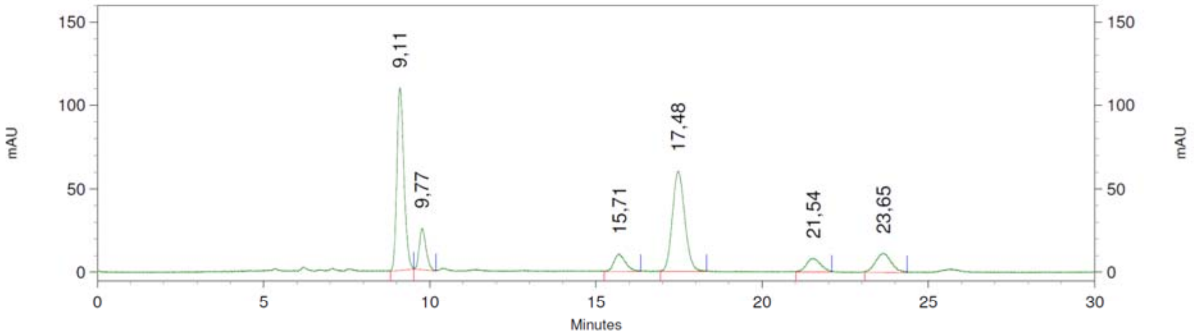

51: 279 nm, 4 nm
Results

Retention Time	Area	Area Percent
9,11	6442861	36,566
9,77	1408402	7,993
15,71	1029924	5,845
17,48	6273730	35,606
21,54	956174	5,427
23,65	1508629	8,562

Enantioenriched product

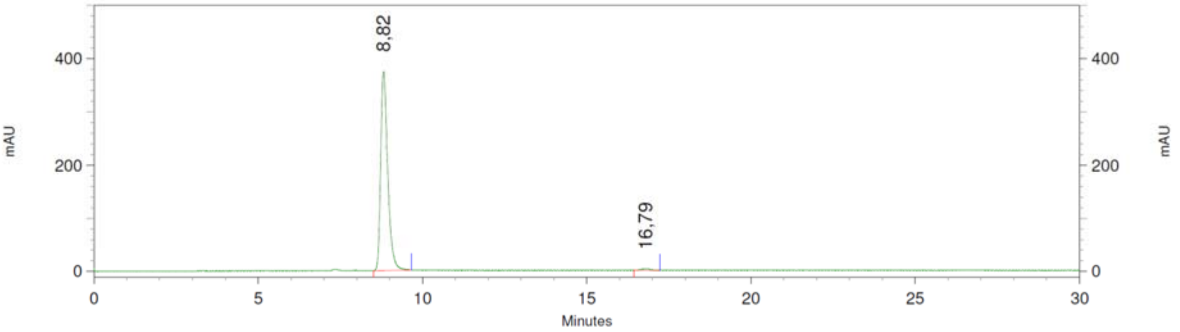

51: 279 nm, 4 nm
Results

Retention Time	Area	Area Percent
8,82	22372993	98,885
16,79	252331	1,115

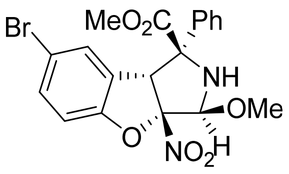

9

Racemic product

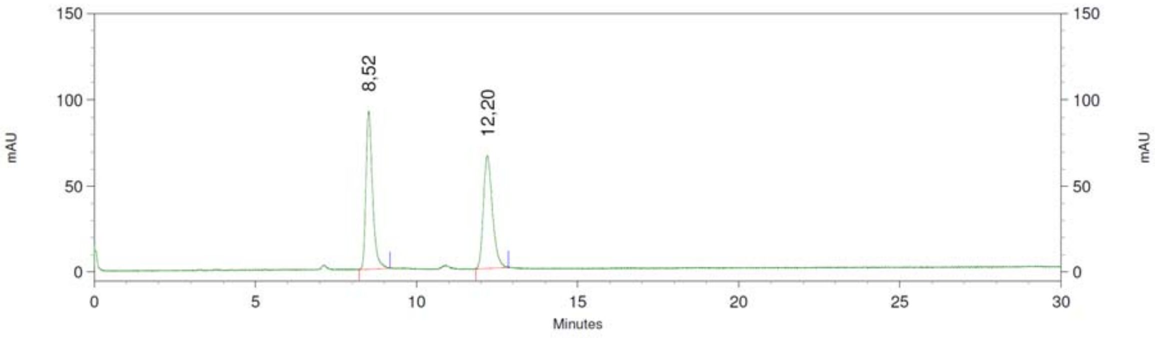

3: 286 nm, 4 nm Results

Retention Time	Area	Area Percent
8,52	5248038	50,578
12,20	5128117	49,422

Enantioenriched product

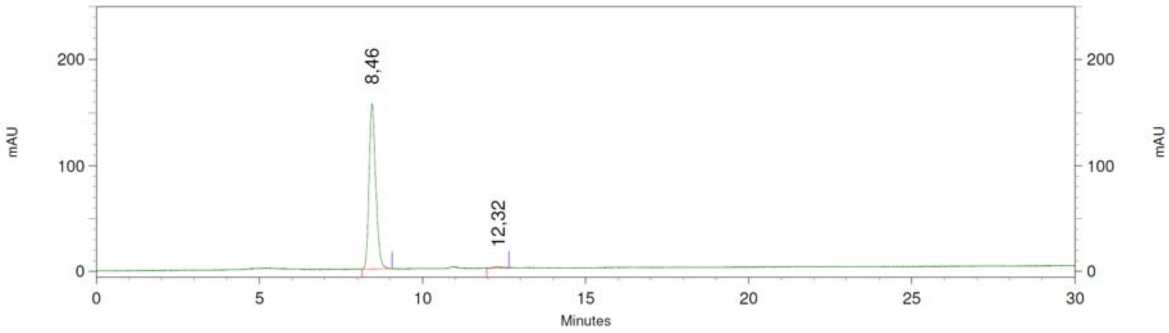

3: 286 nm, 4 nm Results

Retention Time	Area	Area Percent
8,46	8909263	98,984
12,32	91481	1,016

Figure S-1. Ortep plot for the X-ray structure of compound **9**. Thermal ellipsoids drawn at the 50% probability level. Flack parameter 0.005(15)

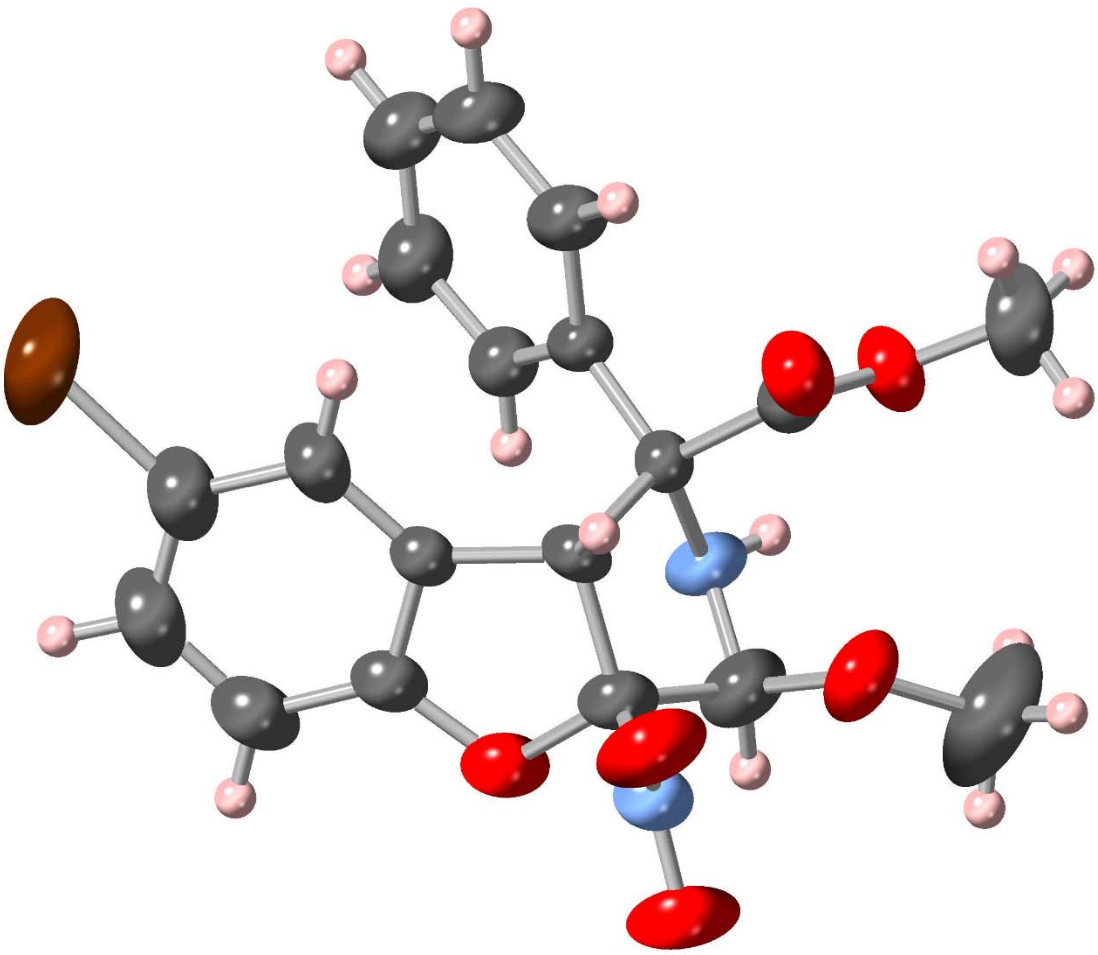

Optimization of the reaction conditions. Further experiments

Table 1. Optimization of the catalytic system.[a]

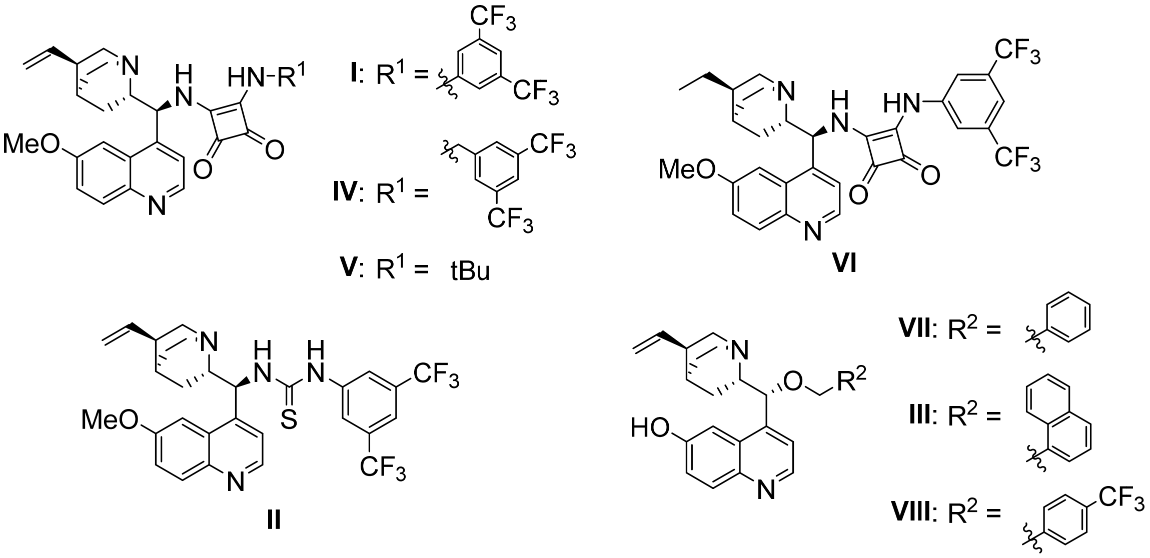

Entry	Catalyst	Yield (%)	dr	*ee* (%)
1	**I**	99	50:50	35/37
2[b]	**I**	100	61:39	10/30
3	**IV**	65	10:90	59/39
4	**V**	58	32:68	90/23
5	**VI**	93	39:61	56/39
6	**II**	62	17:83	80/6
7	**VII**	52	4:96	19/77
8	**III**	73	3:97	9/73
9[b]	**III**	100	76:24	11/38
10	**VIII**	91	12:88	10/63

[a] Reaction conditions: **1a** (16.3 mg, 0.1 mmol), **2a** (19 μL, 0.13 mmol), and catalyst (0.01 mmol) in CH_2Cl_2 (1 mL) at room temperature. [b] Reaction carried out in presence of Ag_2O (1.2 mg, 0.005 mmol).

Table 2. Catalytic loading, concentration and temperature studies.[c]

Entry	Cat. load (mol %)	T (°C)	Conc. of 1a (M)	Yield (%)	dr	*ee* (%)
1	10	r.t.	0.1	73	3:97	9/73
2	15	r.t.	0.1	65	-:100	n.d./76
3	15	0	0.1	48	-:100	n.d./93
4	15	0	0.2	75	-:100	n.d./90
5	10	0	0.2	74	-:100	n.d./90

[c] Reaction conditions: **1a** (16.3 mg, 0.1 mmol), **2a** (19 μL, 0.13 mmol), and **III** in CH_2Cl_2.

Table 3. Solvent analysis.[d]

Entry	Solvent	Yield (%)	dr	*ee* (%)
1	DCM	74	-:100	n.d./90
2	Toluene	40	-:100	n.d./93
3	1,2-DCE	58	-:100	n.d./86
4	MTBE	46	-:100	n.d./74
5	$CHCl_3$	79	-:100	n.d./93

[d] Reaction conditions: **1a** (16.3 mg, 0.1 mmol), **2a** (19 μL, 0.13 mmol), and **III** (4.5 mg, 0.01 mmol) in the corresponding solvent (0.5 mL) at 0 °C.
